# Development of an On-DNA Platform Molecule Bearing a Diazidestructure and Its Application to DEL Synthesis

**DOI:** 10.3390/ijms26199501

**Published:** 2025-09-28

**Authors:** Hiroyuki Miyachi, Masaki Koshimizu, Manussada Ratanasak, Yasuteru Shigeta, Masashi Suzuki

**Affiliations:** 1 Lead Exploration Unit, Drug Discovery Initiative, University of Tokyo, 7-3-1 Hongo, Bunkyo, Tokyo 113-0033, Japan; m-koshimizu@g.ecc.u-tokyo.ac.jp (M.K.); suzukimasashi@g.ecc.u-tokyo.ac.jp (M.S.); 2 Center for Computational Sciences, University of Tsukuba, 1-1-1 Tennodai, Tsukuba 305-8577, Ibaraki, Japan; manussada@ccs.tsukuba.ac.jp (M.R.); shigeta@ccs.tsukuba.ac.jp (Y.S.)

**Keywords:** DEL, chemical space, on-DNA diazide platform, DAP, chemoselective transformation

## Abstract

Expanding the chemical space of DNA-encoded libraries (DELs) is desirable for identifying novel bioactive compounds and enhancing hit quality in affinity-based screening. In this study, we designed and synthesized a new on-DNA diazide platform (DAP) molecule that incorporates both aromatic and aliphatic azido groups within a single scaffold. These orthogonal azides exhibit distinct reactivity profiles, enabling a stepwise warhead construction strategy through chemoselective transformations. This approach facilitates greater structural diversity and efficient incorporation of diverse building blocks. A virtual DEL was generated based on this DAP scaffold, and its chemical space was compared with that of bioactive compounds in the ChEMBL database. The analysis revealed that this virtual library occupied a distinct and previously unexplored region of chemical space, highlighting the potential of this DAP-based strategy for discovering structurally novel DEL members with biological relevance.

## 1. Introduction

DNA-encoded libraries (DELs), first proposed by Professor Richard A. Lerner and Professor Sydney Brenner in 1992 [1], have rapidly emerged as a powerful discovery technology for drug targets and an innovative platform for high-throughput screening [2]. This technology enables the covalent attachment of small molecules (warheads) to DNA tags, allowing the structural information of each compound to be encoded in a specific DNA sequence. Consequently, the construction and screening of extremely large libraries become feasible [3].

To fully leverage the potential of DEL technology, it is essential to expand the chemical space of the library and to explore previously uncharted regions within this space [4]. Chemical space refers to the theoretical set of all possible chemical structures. The extent to which a DEL occupies this space directly impacts both the diversity and the quality of the hit compounds obtained from screening [5].

In this context, the strategic use of platform molecules has recently attracted considerable attention [6]. Platform molecules are core scaffolds bearing multiple functional groups or reactive handles that allow for diverse structural elaboration through the incorporation of various building blocks (BBs). The use of platform molecules in DEL design enables flexible and efficient synthetic transformations, facilitating the generation of structurally unique compound libraries. Moreover, the rational design of platform molecules promotes modular library construction, enhances synthetic reproducibility, and improves the acquisition of structure–activity relationship data [7]. These attributes can contribute to the overall efficiency of the hit-to-lead optimization process.

The exploration and coverage of uncharted chemical space in DEL synthesis are critical not only for increasing the probability of hit identification but also for enabling access to innovative and previously intractable drug targets—making DELs a strategically vital element of modern drug discovery.

In the present study, we report on the design and synthesis of platform molecules incorporating diazide structures as synthetic entry points for warhead elaboration [8], along with their application to DEL synthesis.

## 2. Results and Discussion

### 2.1. Organocatalyzed [3+2] Cycloaddition Reactions of On-DNA Organic Azides

The azido group (N_3_) is a highly versatile functional group widely utilized in drug discovery and chemical biology because of its high reactivity and compact molecular size, which enables a broad range of chemical transformations. In particular, the azido group plays a central role in copper-catalyzed azide–alkyne cycloaddition (CuAAC), a well-known “click chemistry” reaction that allows for the selective introduction of functional moieties into target molecules [9,10,11]. Additionally, the azido group can be readily reduced to an amino group, functioning as a latent amine [12]. Amino groups serve as valuable sites for further derivatization, such as amide or sulfonamide formation, facilitating the structural diversification of small molecules.

CuAAC reactions have been extensively employed in the synthesis of DELs. However, this methodology predominantly yields 1,4-disubstituted 1,2,3-triazoles. In contrast, 1,4,5-trisubstituted 1,2,3-triazoles possess greater structural complexity and are thus more useful for chemical space expansion. Therefore, it is highly desirable to develop DNA-compatible chemical reactions capable of constructing such triazole architectures [13].

Recently, an organocatalyzed [3+2] cycloaddition reaction between enolates and on-DNA azides has been reported as a method for synthesizing 1,4,5-trisubstituted 1,2,3-triazoles [12]. In the present study, we focused on this organocatalytic [3+2] cycloaddition as a means to expand the DEL chemical space.

**Figure 1:**
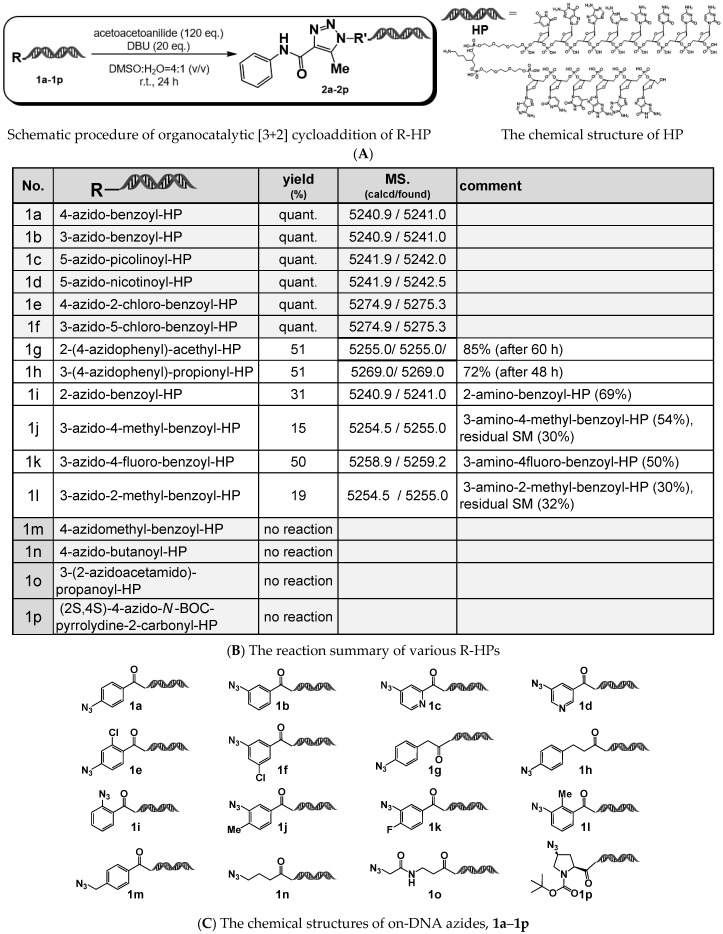
On-DNA organocatalyzed [3+2] cycloaddition reactions. (**A**) Representative reaction scheme of the [3+2] cycloaddition between azide-functionalized headpiece (HP) and acetoacetoanilide in the presence of DBU. The structure of the HP is shown. (**B**) Summary of the results obtained from the DBU-mediated [3+2] cycloaddition. (**C**) Chemical structures of the azide-functionalized HPs.

As a model reaction, we investigated the reactivity of various organic azides attached to a Head Piece (HP) (sequence: 5′-/5Phos/GAGTCA/iSp9/iUniAmM/iSp9/TGACTCCC-3′), using 1,8-diazabicyclo [5.4.0] undec-7-ene (DBU) as the organocatalyst. Acetoacetanilide was employed as the active methylene compound. The results of the organocatalyzed [3+2] cycloaddition are shown in Figure 1.

Consistent with previous reports, aryl azide–HPs generally showed reactivity under these conditions (**1a**–**1l**), whereas alkyl azide–HPs (both primary and secondary azides) did not exhibit reactivity (**1m**–**1p**). Among the aryl azide–HPs, 4- (or 3-) azidobenzamide–HPs afforded the corresponding 1,4,5-trisubstituted 1,2,3-triazole–HPs quantitatively after 24 h at room temperature (**1a** and **1b**). In contrast, 2-(4-azidophenyl)acetamide–HP and 3-(4-azidophenyl)propionamide–HP showed slower reaction kinetics, with yields of approximately 50% after 24 h (**1g** and **1h**).

When using on-DNA aryl azide–HPs bearing substituents at the ortho position relative to the azide group, low product yields (15–50%) were observed regardless of whether the substituent was electron-donating or electron-withdrawing (**1i**–**1l**). In these cases, the major products were suspected to be the corresponding amines, resulting from the reduction of the azide group.

Notably, the observed reactivity of the ortho-substituted aryl azide–HPs differed from the previously reported behavior [14], although the underlying cause of this discrepancy remains unclear at present.

### 2.2. Computational Analysis of Reactivity in the Organocatalyzed [3+2] Cycloaddition of On-DNA Organic Azides

The reactivity of on-DNA organic azides in organocatalyzed [3+2] cycloaddition reactions was categorized into four distinct groups based on the performance characteristics:

Group 1 (G1): High reactivity with excellent yields

Group 2 (G2): Reactive but with slow reaction kinetics

Group 3 (G3): Reactive but with low yields

Group 4 (G4): No observable reaction

To achieve a molecular-level understanding of the observed differences in reactivity, we performed a computational analysis based on frontier molecular orbital theory, with particular emphasis on the following two key energy parameters.

ΔE_1_: Represents the energy gap between the highest occupied molecular orbital (HOMO) and the lowest unoccupied molecular orbital (LUMO) of the azide group, defined as:

ΔE_1_ = E_HOMO_ (azide) − E_LUMO_ (azide)

ΔE_2_: Denotes the energy difference between the HOMO of the active methylene (AM) compound and the LUMO of the azide group, which serves as an indicator of the nucleophilic attack feasibility:

ΔE_2_ = E_HOMO_ (AM) − E_LUMO_ (azide)

In general, smaller values of ΔE_1_ and ΔE_2_ correlate with higher reactivity.

All quantum chemical calculations were conducted using Gaussian16 (Revision C.02) [15] employing the M06-2X functional [16] with the 6-31+G(d,p) basis set [17]. Solvent effects were incorporated via the SMD continuum solvation model [18], with dimethyl sulfoxide (DMSO; dielectric constant, ε = 46.826) as the solvent environment. To optimize computational efficiency, the DNA-conjugated moiety (HP) was represented by a simplified methylamide model.

**Figure 2:**
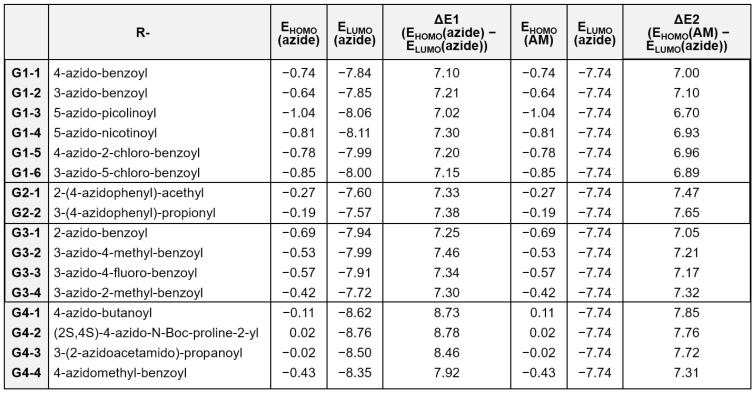
Computational analysis of the reactivity of on-DNA organic azides in the organocatalyzed [3+2] cycloaddition reaction.

Figure 2 presents the calculated ΔE_1_ and ΔE_2_ values for representative compounds across distinct reactivity groups. A comparative analysis between the highly reactive G1 group and the unreactive G4 group revealed that compounds within G1 exhibited significantly smaller ΔE_1_ and ΔE_2_ values, whereas those in G4 displayed larger energy gaps, indicative of energetically unfavorable conditions for the cycloaddition reaction. Specifically, the average ΔE_1_ and ΔE_2_ values for G1 were 7.16 and 6.93 eV, respectively, compared with 8.48 and 7.66 eV for G4.

In the G3 group, which is characterized by low reaction yields, the average ΔE_1_ value (7.34 eV) was comparable to that of G1; however, the ΔE_2_ values were higher (average: 7.19 eV) than the values in G1, suggesting that suboptimal orbital interactions may contribute to the diminished yields. The G2 group, which is associated with slow reaction kinetics, exhibited a high average ΔE_2_ value (7.56 eV), comparable to that of the unreactive G4 group. This result suggested that a large ΔE_2_ value is the primary factor contributing to the reduced reaction rate (average ΔE_1_ for G2 = 7.36 eV).

Collectively, these findings demonstrated that both ΔE_1_ and ΔE_2_ are valuable predictive parameters for selecting suitable organic azides in the rational design of DELs utilizing organocatalyzed [3+2] cycloaddition chemistry.

### 2.3. Design of the On-DNA Platform Molecule: N-(4-Azidobenzoyl)-azidohomoalanine-HP (homoAla-Based DAP)

Aromatic azide–HP conjugates lacking ortho-substituents were shown to undergo the organocatalyzed [3+2] cycloaddition efficiently, yielding the corresponding 1,4,5-trisubstituted 1,2,3-triazoles in high yields. In contrast, aliphatic azide–HP conjugates exhibited no reactivity in this transformation.

**Figure 3:**
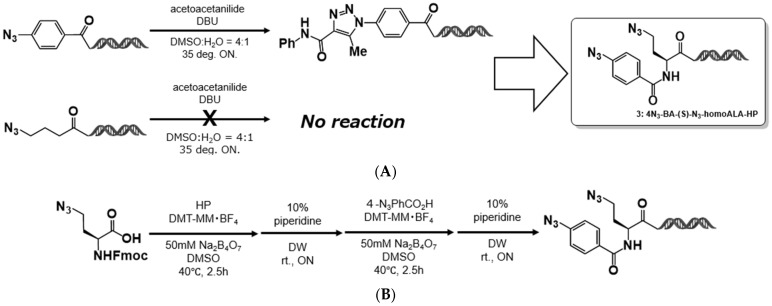
On-DNA platform molecule. (**A**) Design of a representative on-DNA platform molecule, 4N_3_-BA-(*S*)-N_3_-homoAla-HP. (**B**) Synthetic route for 4N_3_-BA-(*S*)-N_3_-homoAla-HP.

From this observation, we hypothesized that in an on-DNA azide bearing both aromatic and aliphatic azido groups within the same molecule, only the aromatic azide would undergo the organocatalyzed [3+2] cycloaddition, while the aliphatic azide would remain intact. The unreacted aliphatic azide could then be used in subsequent transformations, such as CuAAC, or as a latent amino group after reduction. This strategy offers a promising route to develop a class of multifunctional on-DNA platform molecules amenable to sequential modification (Figure 3A) [19,20,21].

To test this concept, we designed a model compound, (*S*)-4-azido-2-(4-azidobenzamido)butanoic acid–HP (4N_3_-BA-(*S*)-N_3_-homoAla–HP). This molecule was synthesized at the nanomole scale by coupling commercially available (*S*)-N_3_-homoAla–OH with the HP using 4-(4,6-dimethoxy-1,3,5-triazin-2-yl)-4-methylmorpholinium tetrafluoroborate (DMT-MM BF_4_) as a coupling reagent, followed by piperidine treatment to yield (*S*)-N_3_-homoAla–HP. A second coupling with 4-azidobenzoic acid using DMT-MM BF_4_ and subsequent piperidine treatment afforded the desired product in an overall yield of 64% (4-step yield) (Figure 3B). The resulting compound was used in subsequent reactions without further purification.

**Figure 4:**
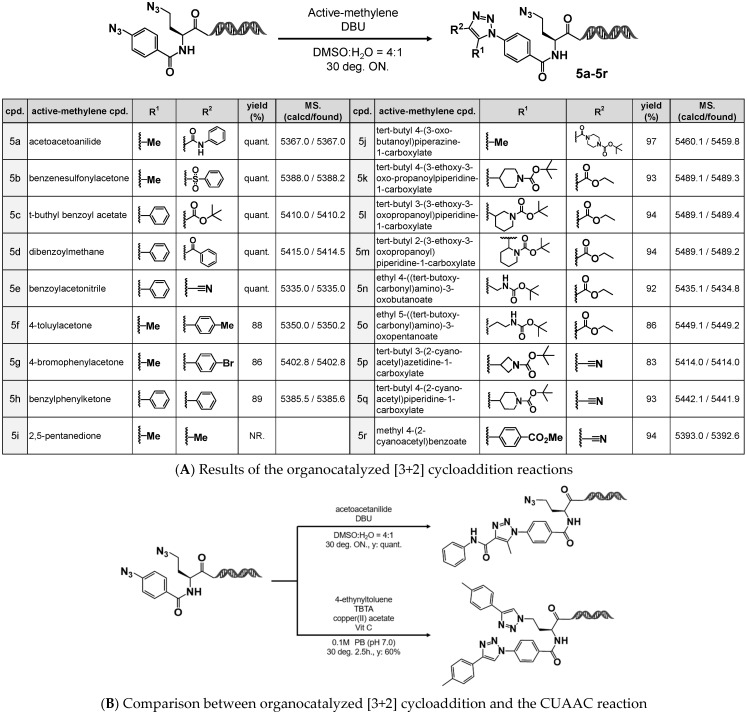
Cycloaddition reactions of 4N_3_-BA-(*S*)-N_3_-homoAla-HP. (**A**) Organocatalyzed [3+2] cycloaddition reactions of 4N_3_-BA-(*S*)-N_3_-homoAla-HP with various active methylene compounds. (**B**) Comparison between organocatalyzed [3+2] cycloaddition and copper-catalyzed cycloaddition of 4N_3_-BA-(*S*)-N_3_-homoAla-HP.

The synthesized 4N_3_-BA-(*S*)-N_3_-homoAla–HP was then subjected to organocatalyzed [3+2] cycloaddition reactions with various active methylene compounds. As shown in Figure 4, the reaction proceeded quantitatively with all tested active methylene partners, including the β-ketoanilide (**5a**) used during model compound synthesis, as well as β-ketosulfone (**5b**), β-ketoester (**5c**), 1,3-diketone (**5d**), and α-ketonitrile (**5e**). Monoketones containing aromatic rings also yielded the desired 1,4,5-trisubstituted triazoles in yields greater than 80% (**5f**–**5h**).

Compounds **5j**–**5r** are examples of active methylene compounds bearing functional handles at the R^1^ and R^2^ positions, designed to enable expansion of the structural diversity. Notably, the use of these compounds as starting materials afforded 1,4,5-trisubstituted 1,2,3-triazoles in high yields, thereby demonstrating the applicability of this class of scaffolds in the reaction system.

In contrast, the reaction did not proceed with an aliphatic monoketone (**5i**), likely because of the lower acidity of the α-hydrogen adjacent to the carbonyl group, which would impede enolate formation under the reaction conditions.

Product formation was confirmed using quadrupole time-of-flight mass spectrometry (QTOF-MS) in negative ion mode. In all cases, the detected molecular ion peaks indicated that only one azide moiety had participated in the organocatalyzed [3+2] cycloaddition, with no evidence for double triazole formation involving both azides.

Conversely, when 4N_3_-BA-(*S*)-N_3_-homoAla–HP was subjected to CuAAC with 4-ethynyltoluene, both azides reacted non-selectively to afford the bis-triazole product in approximately 60% yield. No mono-triazole intermediates were observed under these conditions (Figure 4B).

### 2.4. Stepwise Construction of On-DNA Compounds Using 4N_3_-BA-(S)-N_3_-homoAla–HP (Double-Click Strategy)

To evaluate the utility of 4N_3_-BA-(*S*)-N_3_-homoAla–HP as an on-DNA platform molecule bearing both aromatic and aliphatic azides within the same structure, we carried out a stepwise click reaction sequence (double clicked DEL).

**Figure 5:**
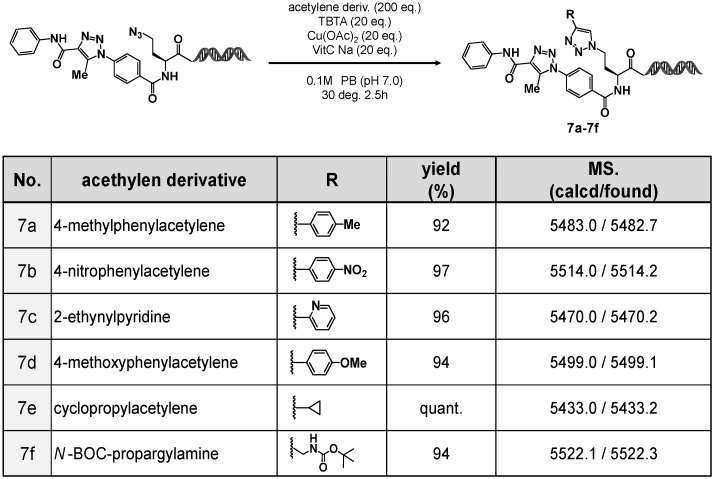
Copper-catalyzed cycloaddition leading to 1,4,5-trisubstituted 1,2,3-triazole-containing (*S*)-N_3_-homoAla-HP compounds.

As shown in Figure 5, various alkynes, aromatic alkynes (**7a**, **7b**, and **7d**), a heteroaromatic alkyne (**7c**), and aliphatic alkynes (**7e** and **7f**), were employed in the CuAAC reactions. In all cases, the corresponding 1,4-disubstituted 1,2,3-triazoles were obtained in high yields.

These results demonstrated the effectiveness of 4N_3_-BA-(*S*)-N_3_-homoAla–HP as a new on-DNA platform molecule. By taking advantage of the orthogonal reactivity of the two azides (aromatic and aliphatic), it is possible to perform sequential organocatalyzed [3+2] cycloaddition and CuAAC reactions, thus enabling the stepwise construction of on-DNA compound libraries containing two 1,2,3-triazole motifs with distinct substitution patterns.

### 2.5. Development of On-DNA Azide–Amine Platform Molecules: 4N_3_-BA-(S)-N_3_-Lys–HP and (2S,4S)-4N_3_-BA-(S)-N_3_-Pro–HP

**Figure 6:**
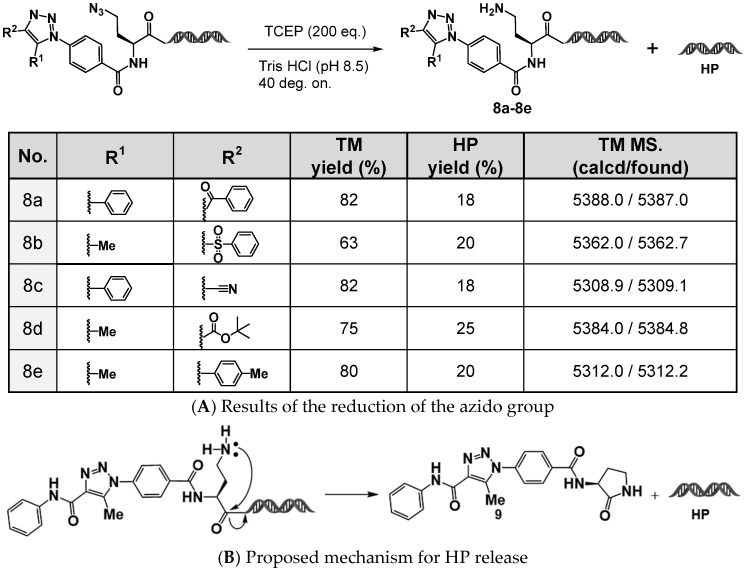
Reduction of (S)-N_3_-homoAla-HP derivatives. (**A**) TCEP-mediated reduction of the azide group in 1,4,5-trisubstituted 1,2,3-triazole-containing (*S*)-N_3_-homoAla-HP derivatives. (**B**) Proposed mechanism for headpiece (HP) release following TCEP reduction.

Azides, which are readily reducible to primary amines, can act as latent amines that can be further utilized in capping reactions, such as amidation or sulfonamidation. This amine functionality contributes to the ability for structural diversification and is particularly valuable in DEL synthesis. Using this concept, we investigated the reduction of the aromatic azide in 4N_3_-BA-(*S*)-N_3_-homoAla–HP (Figure 6A).

The target compounds, (*S*)-NH_2_-homoAla–HP derivatives, were successfully obtained in 60–80% yields using tris(2-carboxyethyl)phosphine (TCEP) as a reductant. However, for all the substrates tested, QTOF-MS analysis revealed the formation of a byproduct eluting at approximately 2.05 min, with a molecular mass of 4936.8, suggesting that cleavage had occurred to liberate the HP moiety. This side reaction is likely caused by the nucleophilic attack of the generated primary amino group on the adjacent amide carbonyl group, resulting in the intramolecular formation of a γ-lactam (five-membered ring), which in turn releases the HP moiety (Figure 6B).

Such cleavage is undesirable for DEL production, and therefore structural modifications to prevent intramolecular lactamization were considered. Two strategies were explored:

Extension of the length of the carbon side chain to inhibit 5-membered lactam formation (use of a lysine-type amino acid scaffold);

Conformational restriction via side-chain cyclization (use of a proline-type amino acid scaffold). On the basis of these strategies, we designed and synthesized two on-DNA dual azide platform (DAP) molecules:

(*S*)-6-azido-2-(4-azidobenzamido)hexanoic acid–HP (4N_3_-BA-(*S*)-N_3_-Lys–HP)

(*2S*,*4S*)-4-azido-1-(4-azidobenzoyl)proline–HP ((*2S*,*4S*)-4N_3_-BA-(*S*)-N_3_-Pro–HP).

These compounds were successfully synthesized using the same procedures as for 4N_3_-BA-(*S*)-N_3_-homoAla–HP.

**Figure 7:**
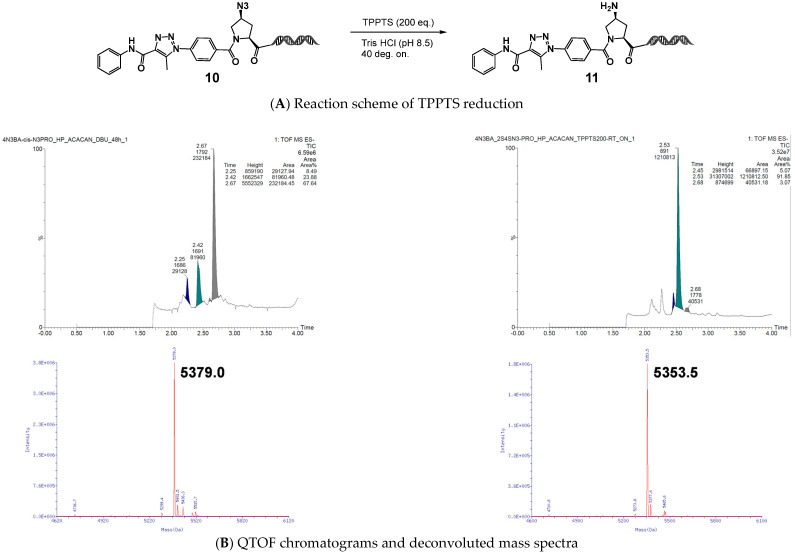
Reduction of the diazide platform molecule, (2*S*,4*S*)-4N_3_-BA-(*S*)-N_3_-Pro-HP. QTOF chromatograms and deconvoluted mass spectra of the starting material and the TPPTS-reduced product.

We next evaluated (*2S*,*4S*)-4N_3_-BA-(*S*)-N_3_-Pro–HP as a model compound. After reaction with acetoacetanilide under DBU-mediated organocatalyzed [3+2] cycloaddition conditions, the resulting 1,2,3-triazole was subjected to reduction using the trisodium salt of triphenylphosphine-3,3′,3″-trisulfonic acid (TPPTS). The desired amino proline–HP was obtained in nearly quantitative yield, and QTOF MS analysis confirmed the absence of HP cleavage (Figure 7).

Similarly, no HP detachment was observed when using the lysine-type on-DNA DAP (4N_3_-BA-(*S*)-N_3_-Lys–HP). Thus, we concluded that the partial cleavage of HP observed with the reduction of 4N_3_-BA-(*S*)-N_3_-homoAla–HP was because of intramolecular lactam formation, and demonstrated that this issue can be circumvented through rational structural modifications, either by extending or cyclizing the side chain. The newly developed DAPs, 4N_3_-BA-(*S*)-N_3_-Lys–HP and (*2S*,*4S*)-4N_3_-BA-(*S*)-N_3_-Pro–HP, effectively eliminated this problem. These findings pave the way for utilizing the primary amines obtained via reduction as functional handles for diversification in DEL construction.

### 2.6. DEL Synthesis Using 4N_3_-BA-(2S,4S)-N_3_-Pro–HP: On-DNA Di-Azide and Azide–Amine Platforms

**Figure 8:**
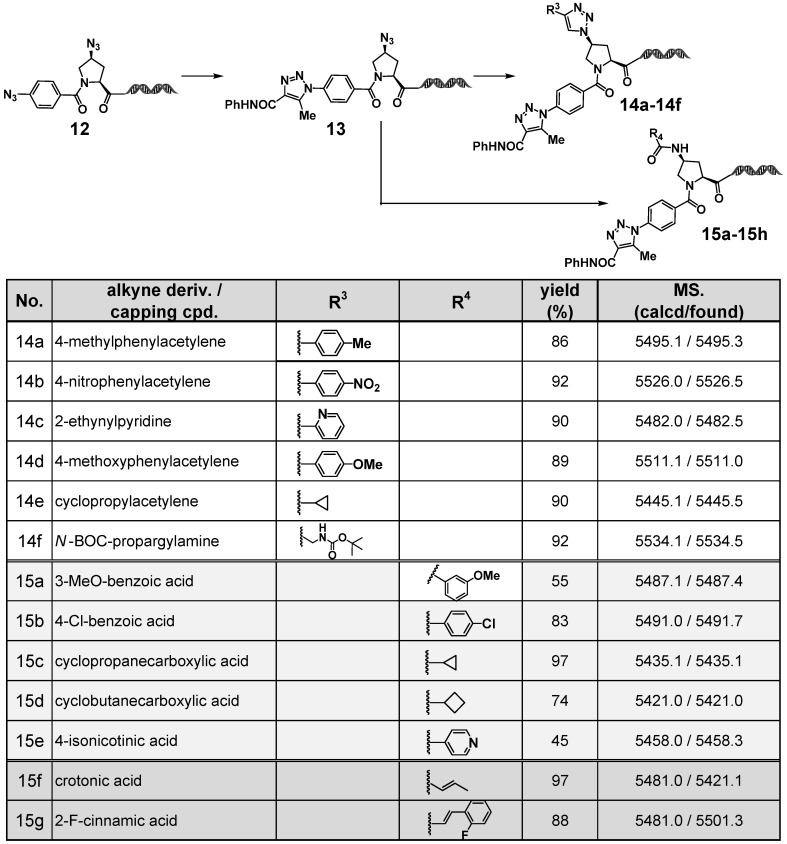
Sequential transformations of (2*S*,4*S*)-4N_3_-BA-(*S*)-N_3_-Pro-HP via double click reactions followed by amidation.

To explore the utility of a more synthetically practical azide-bearing latent amine platform molecule, we employed the newly developed 4N_3_-BA-(*2S*,*4S*)-N_3_-Pro–HP and investigated the stepwise application of organocatalyzed [3+2] cycloaddition and CuAAC reactions to the two azides with differing reactivity. In addition, we evaluated nitrogen capping reactions of the amino group generated by the reduction of the aliphatic azide. The results are summarized in Figure 8.

As observed previously with the 4N_3_-BA-(*S*)-N_3_-homoAla–HP platform, the stepwise reactions using aryl alkynes, a heteroaryl alkyne, and aliphatic alkynes all proceeded efficiently, affording the desired 1,4-disubstituted 1,2,3-triazoles in high yields (**14a**–**14f**). These results demonstrated that 4N_3_-BA-(*2S*,*4S*)-N_3_-Pro–HP can enable the construction of on-DNA compound libraries containing two distinct 1,2,3-triazole moieties with different substitution patterns by sequential application of the organocatalyzed [3+2] cycloaddition and CuAAC reactions to the two orthogonally reactive azides.

The aliphatic azido group could be successfully reduced using TPPTS to generate a primary amine, which served as a functional handle for nitrogen capping (amidation). As shown in Figure 8, condensation with aryl carboxylic acids, heteroaryl carboxylic acids, and aliphatic carboxylic acids proceeded in good to excellent yields using *O*-(7-azabenzotriazol-1-yl)-*N*,*N*,*N*′,*N*′-tetramethyluronium hexafluorophosphate (HATU) as the coupling agent and *N*,*N*-diisopropylethylamine (DIPEA) as the base (**15a**–**15f**).

The resulting amine handle can serve not only for derivatization with simple carboxylic acid-based BBs, but also for the incorporation of functional modules. As an example, we explored on-DNA synthesis aimed at the discovery of covalent binders. Covalent ligands bearing electrophilic functional groups are particularly effective for targeting “undruggable” proteins, which often exhibit weak or transient interactions with non-covalent ligands [22]. By forming selective and durable covalent bonds, such ligands improve the binding specificity and stability, enabling highly sensitive screening and expanding the utility of DEL technology in drug discovery [23].

In compounds **15g** and **15f**, the α,β-unsaturated carboxylic acids, crotonic acid and 2-fluorocinnamic acid, were employed as the electrophilic functional groups. These acids, similar to the previously tested BBs, successfully underwent amidation with the reduced amine handle under HATU–DIPEA conditions, affording the desired amide products in excellent yields.

### 2.7. Validation of the Practical Utility of On-DNA DAP via Mock DNA-Encoded Pool Synthesis

Using the platform molecule 4N_3_-BA-(*2S*,*4S*)-N_3_-Pro-HP, we demonstrated that sequential click reactions targeting the two azides with different reactivities could enable the construction of on-DNA compounds bearing two 1,2,3-triazole rings with distinct substitution patterns. Furthermore, we showed that the amino group generated by the reduction of the aliphatic azide after the organocatalyzed [3+2] cycloaddition can serve as a handle for nitrogen capping with various BBs and functional modules. These findings were based on a two-step reaction scheme performed on a single substrate.

To evaluate the applicability of this platform under conditions more representative of actual DEL synthesis, we conducted a mock DNA-encoded pool synthesis starting from 4N_3_-BA-(*2S*,*4S*)-N_3_-Pro-HP. Creation of this mock pool is a critical step prior toward full-scale DEL construction, allowing verification of the compatibility of the synthetic methods with DNA tagging [24].

**Figure 9:**
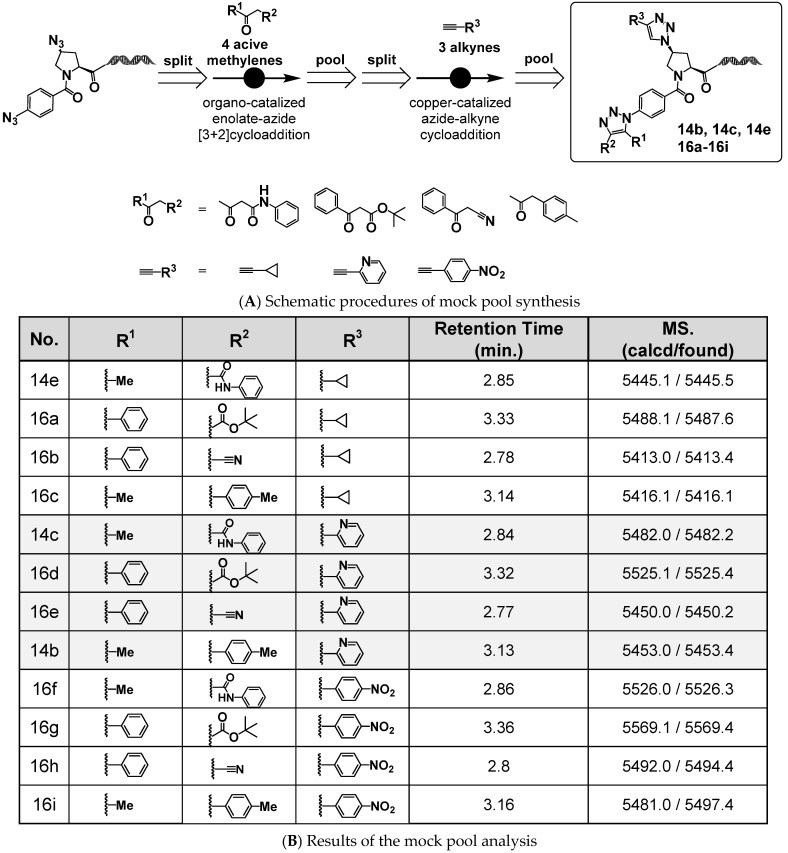
Mock DNA-encoded library synthesis via double click reactions of (2*S*,4*S*)-4N_3_-BA-(*S*)-N_3_-Pro-HP.

An aqueous solution of 4N_3_-BA-(*2S*,*4S*)-N_3_-Pro-HP was first divided into four portions, each portion being subjected to an organocatalyzed [3+2] cycloaddition with different active methylene compounds. The resulting products were combined, precipitated by ethanol, and lyophilized to obtain intermediate mixtures. These mixtures were redissolved in water, split into three portions, and then reacted with various alkyne derivatives via CuAAC. A portion of the reaction products was analyzed individually (Reaction Analysis 1), while the remainder was pooled, re-precipitated, re-lyophilized, re-dissolved, and analyzed collectively (Reaction Analysis 2) (Figure 9A).

**Figure 10:**
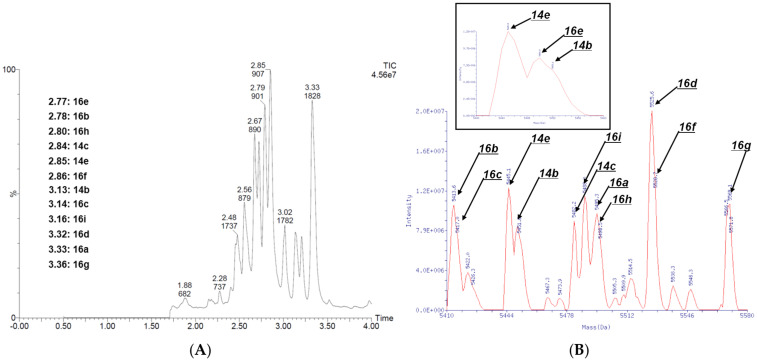
Results of mock DNA-encoded library synthesis via double click reactions of (2*S*,4*S*)-4N_3_-BA-(*S*)-N_3_-Pro-HP. (**A**) QTOF chromatogram of a mixture of 12 double click reaction products. (**B**) Deconvoluted mass spectrum of the mixture in (**A**).

Figure 9B shows the QTOF measurements from Reaction Analysis 1 for each of the 4 × 3 compound mixtures. Figure 10 shows the QTOF chromatograms and mass spectra of the 12 compound mixtures analyzed in Reaction Analysis 2.

These data indicate that the CuAAC reactions proceeded successfully even when starting from the mixtures of four 1,4,5-trisubstituted 1,2,3-triazoles derived from the organocatalyzed [3+2] cycloadditions. However, the presence of some unreacted starting materials indicated that there is a need for further optimization of the CuAAC reaction conditions.

Importantly, all 12 target compounds were detected via QTOF chromatographic and mass spectrometric analysis. These results strongly support the feasibility of employing 4N_3_-BA-(*2S*,*4S*)-N_3_-Pro-HP as a platform molecule for DEL synthesis and indicate its potential applicability in constructing screening libraries for practical drug discovery campaigns.

### 2.8. Assessment of DNA Damage During the Full-Length On-DNA Synthesis of DAP Compounds

As part of an evaluation of the practical applicability of DEL synthesis employing 4N_3_-BA-(2*S*,4*S*)-N_3_-Pro-HP as a novel platform molecule, we undertook the continuous synthesis of a single DEL. In this system, barcode DNAs were enzymatically ligated to stepwise click-reaction intermediates derived from the platform molecule, and the resulting full-length DEL was subjected to quantitative PCR (qPCR) to assess the impact of the reaction sequence on DNA amplification [25,26,27].

For this purpose, two distinct full-length DELs were designed and synthesized: Control-HP-AOP-P-TAG1-TAG2-CP and DAP-HP-AOP-P-TAG1-TAG2-CP. The AOP moiety corresponds to a 15-amino-4,7,10,13-tetrapentaoxadecanoyl group functioning as a spacer, whereas P and CP denote the double-stranded opening and closing primers, respectively. TAG1 and TAG2 represent double-stranded DNA tag elements.

Control-HP-AOP-P-TAG1-TAG2-CP was synthesized as a positive control for qPCR. In this construct, 3-methoxybenzoic acid served as the warhead. Following condensation with HP-AOP-P using DMTMM·BF_4_ as a coupling reagent, TAG1, TAG2, and CP were sequentially ligated by T4 DNA ligase. The construct was thus obtained in a total of four steps: one chemical transformation and three enzymatic ligation steps.

**Figure 11:**
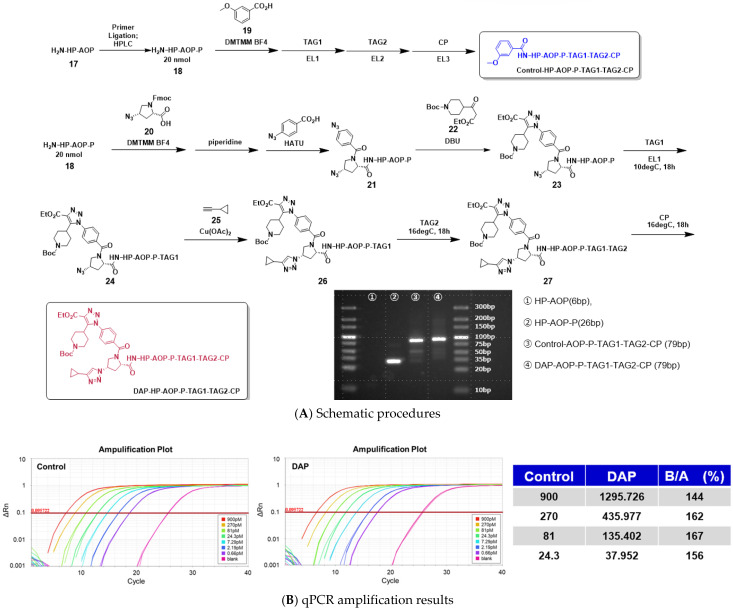
DNA damage analysis following double click reactions of (2*S*,4*S*)-4N_3_-BA-(*S*)-N_3_-PRO-HP. (**A**) Schematic overview of the synthetic procedure for the tested product (Control-AOP-P-TAG1-TAG2-CP (blue), and DAP-AOP-P-TAG1-TAG2-CP (red)), and an agarose gel analysis from a DEL involving two tagging cycles. (**B**) qPCR amplification of Control-AOP-P-TAG1-TAG2-CP, and DAP-AOP-P-TAG1-TAG2-CP, and the quantitative data comparison of the amplification of Control-AOP-P-TAG1-TAG2-CP, and DAP-AOP-P-TAG1-TAG2-CP.

DAP-HP-AOP-P-TAG1-TAG2-CP was prepared as the test compound. Initially, (2*S*,4*S*)-1-(((9*H*-fluoren-9-yl)methoxy)carbonyl)-4-azapyrrolidine-2-carboxylic acid was coupled with HP-AOP-P using DMTMM·BF_4_, followed by piperidine-mediated Fmoc deprotection. Subsequently, 4-azidobenzoic acid was coupled to the N1 atom of proline employing HATU. An organocatalyzed [3+2] cycloaddition with tert-butyl 4-(3-ethoxy-3-oxopropanoyl)piperidine-1-carboxylate was then performed, effecting selective conversion of the aromatic azide. Thereafter, TAG1 was enzymatically ligated by T4 DNA ligase, followed by a CuAAC reaction between the aliphatic azide and cyclopropylacetylene to construct the warhead, tert-butyl 4-(1-(4-((2*S*,4*S*)-2-carbamoyl-4-(4-cyclopropyl-1*H*-1,2,3-triazol-1-yl)pyrrolidin-1-yl)carbonyl)phenyl)-4-(ethoxycarbonyl)-1*H*-1,2,3-triazol-5-yl)piperidine-1-carboxylate. Finally, TAG2 and CP were enzymatically ligated with T4 DNA ligase. The overall synthesis was completed in eight steps, comprising five chemical transformations and three enzymatic ligation steps (Figure 11A).

The outcomes of agarose gel electrophoresis, qPCR amplification curves, and quantitative analyses for both Control-HP-AOP-P-TAG1-TAG2-CP and DAP-HP-AOP-P-TAG1-TAG2-CP are presented in Figure 11B. Electrophoresis revealed distinct single bands at the expected molecular weights for both DEL constructs. Furthermore, both libraries exhibited efficient amplification, with quantitative analysis demonstrating that DAP-HP-AOP-P-TAG1-TAG2-CP achieved amplification comparable to the control. Collectively, these results indicate that the stepwise click-reaction sequence employed herein—comprising organocatalyzed [3+2] cycloaddition and CuAAC—induces negligible, if any, DNA damage during DEL synthesis.

### 2.9. Chemical Space Analysis of a Virtual DEL Originating from 4N_3_-BA-(2S,4S)-N_3_-Pro-HP

To characterize the chemical space of a virtual DEL originating from 4N_3_-BA-(2*S*,4*S*)-N_3_-Pro-HP, we performed a comparative analysis with compounds from the ChEMBL database [28]. Chemical space analysis was carried out using a custom Conda environment, built on the KNIME Analytics Platform (v5.4.3), incorporating Python 3.11.6, RDKit v2024.09.2 [29], and Marvin Extensions 4.7.0 based on Marvin 25.1.3 [30] [MarvinSketch was used for generating and editing chemical structures, primarily for SMARTS pattern creation within KNIME workflows, ChemAxon (https://www.chemaxon.com, accessed on 22 September 2025)]. The BBs used to construct the virtual library were obtained from the Enamine catalog and were selected with consideration of structural diversity [31]. Specifically, after desalting, BBs were transformed into Morgan fingerprints (1024 bits, radius = 2) [32], followed by K-means clustering [33]. The compounds nearest to the centroids of each cluster were selected as representative BBs. Ultimately, 500 active methylene compounds and 500 terminal alkyne derivatives were selected, along with three azidobenzoic acids and four stereochemical isomers of azidoproline.

By systematically combining these selected BBs, a virtual library of approximately six million compounds (referred to as DAP compound sets: Scaffold A and Scaffold B) was generated. For representative compound selection, MiniBatch K-means clustering (number of clusters: 5000) was applied to each of Scaffold A, Scaffold B, and ChEMBL_35 (molecular weight 100–1000, desalted and deduplicated), based on Morgan fingerprints (512 bits, radius = 2) [34]. Structures located near the cluster centroids were extracted as representative compounds. In total, 5000 representative compounds were selected from each of Scaffolds A and B and 90,227 from ChEMBL. Dimensionality reduction was performed using uniform manifold approximation and projection (UMAP) on the combined set of 100,277 (5000 + 5000 + 90,227) compounds with the following parameters: n_neighbors = 100; n_components = 2; and random_state = 42 [35]. The resulting two-dimensional coordinates were used to compare the chemical space coverage of each library. To evaluate the density distribution of each library quantitatively, kernel density estimation (KDE) was performed using gaussian_kde from SciPy [36]. A 500 × 500 grid was defined over the UMAP coordinate space, and areas with density values above the threshold of 0.01 were calculated [37].

**Figure 12:**
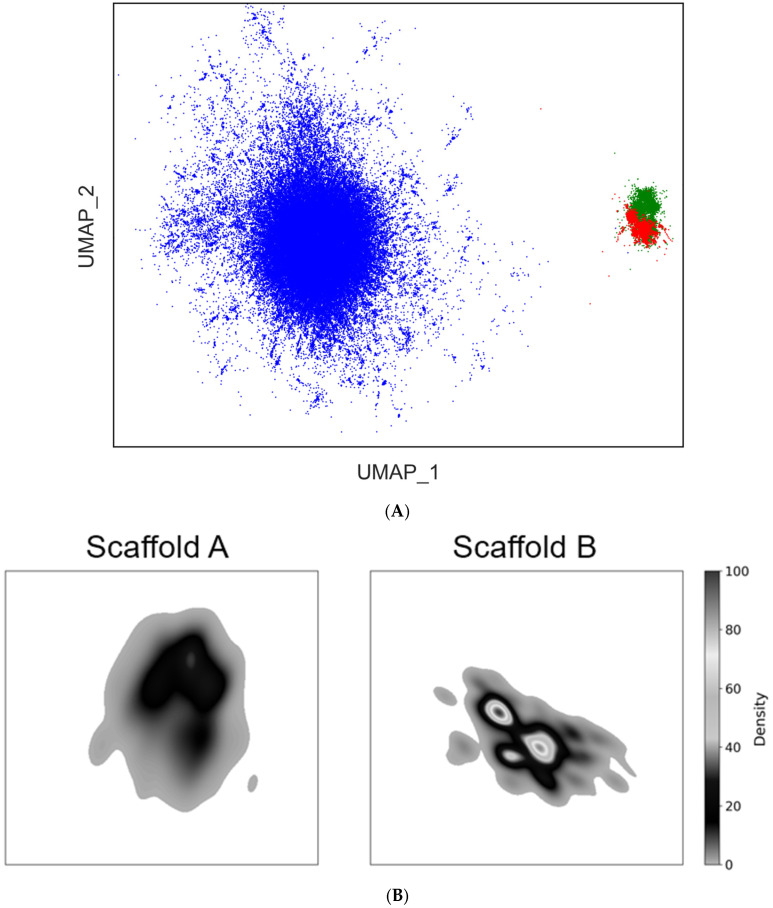
Chemical space analysis of a virtual DEL derived from (2*S*,4*S*)-4N_3_-BA-(*S*)-N_3_-Pro-HP. (**A**) Two-dimensional UMAP of compounds based on 1024-bit Morgan fingerprints (radius = 2). Compounds were categorized into three distinct scaffolds (**A**,**B**), and ChEMBL, shown in green, red, and blue, respectively). The UMAP representation highlights the distribution and clustering of chemical structures in reduced dimensional space, enabling a visual comparison of scaffold-specific chemical space coverage. The fingerprint descriptors were computed to capture structural features relevant to scaffold classification and were subsequently embedded using UMAP for visualization. (**B**) The text shows the kernel density estimation (KDE) of UMAP embeddings for Scaffold A and Scaffold B using a threshold of 0.01. The KDE areas were calculated to quantify the spatial distribution of each scaffold in the UMAP space. Using the KDE area of Scaffold B as 100%, Scaffold A exhibited a relative KDE area of 162%, indicating a broader spatial distribution in the embedded space. The density bars were normalized and scaled between 0 and 100.

As a result, the chemical space of the virtual DEL constructed from the 4N_3_-BA-(2*S*,4*S*)-N_3_-Pro-HP platform, was found to occupy distinct, “uncharted” regions not covered by the ChEMBL-derived library, for both Scaffold A and Scaffold B (Figure 12A). Notably, the significant density region of Scaffold A was approximately twice as large as that of Scaffold B [38]. This result indicated that Scaffold A covers a broader chemical space and supports our initial hypothesis that 1,4,5-trisubstituted 1,2,3-triazole scaffolds, because of their increased structural complexity, would be more advantageous, compared with the simpler 1,4-disubstituted counterparts, in expanding the chemical space (Figure 12B).

## 3. Materials and Methods

### 3.1. General Information

Unless otherwise noted, materials, DNA headpiece (HP-NH2) (5′-/5phos/GAGTCA/iSp9/iUniAmM/iSp9/TGACTCCC-3′, Figure S1) and solvents obtained from commercial suppliers were used without further purification. All on-DNA reactions were performed in 0.2 mL PCR tube or 1.5 mL/2.0 mL micro tubes. On-DNA reactions in the studies of reaction condition optimization and substrate scope extension were analyzed by UPLC-MS. Typically, 1.0 μL samples were dissolved in an appropriate amount of UltraPure^TM^ (Thermo Fisher Scientific Inc., Waltham, MA, USA)distilled water and injected into a reverse-phase chromatography column (Waters XBridge Oligonucleotide BEH C18 column, 1.7 μm, 2.1 × 50 mm) (Waters Inc., Milford, MA, USA) at 60 °C. The elution was carried out as followings: 10–90% solvent B over 4.5 min, 0.4 mL/min, λ = 260 nm; solvent A: water/1,1,1,3,3,3-hexafluoro-2-propanol/triethylamine = 100/2/0.1 (*v*/*v*); solvent B: methanol/1,1,1,3,3,3-hexafluoro-2-propanol/triethylamine/water = 100/2/0.1/2 (*v*/*v*). The effluents were analyzed by a Xevo G2-XS Q-TOF (Waters Inc., Milford, MA, USA) with electrospray ionization source was used for detection.

On DNA reaction yield calculation: Ignoring UV coefficient difference for all on DNA products and assuming 100% of DNA total recovery, the yield of DNA products was determined from total ion chromatography peak area.

### 3.2. General Procedures for the Synthesis of DNA Compounds

#### General Procedure for the Synthesis of DNA-Conjugated Azides

To a solution of DNA headpiece (1 mM in UltraPure™ distilled water) in borate buffer (250 mM, pH 9.5, 20 μL) and UltraPure™ distilled water (80 μL), a mixture consisting of DMTMM·BF_4_ (200 mM in DMSO, 30 μL), azide compound (200 mM in DMSO, 30 μL), and DMSO (40 μL) was added. The resulting mixture was vortexed vigorously, briefly centrifuged, and incubated at 35 °C overnight with shaking. Subsequently, 5 M NaCl (40 μL) and cold ethanol (1.60 mL) were sequentially added. The mixture was vortexed again, centrifuged briefly, and stored at −80 °C for 30 min. After storage, the sample was centrifuged at 16,000× *g* for 30 min at 4 °C to remove the supernatant. The resulting pellet was re-dissolved in UltraPure™ distilled water and used directly in subsequent reactions without further purification.

### 3.3. General Procedure for the On-DNA Enolate–Azide [3+2] Cycloaddition Reaction

To a solution of azido-modified DNA headpiece (500 mM UltraPure™ distilled water solution, 8 μL), DMSO (26.4 μL), an active methylene compound (4.8 μL, 100 mM in DMSO), and DBU (0.8 μL, 100 mM in DMSO) were added sequentially. The resulting mixture was vortexed vigorously, briefly centrifuged, and incubated at 35 °C overnight with continuous shaking. After the reaction, a 1 μL aliquot of the reaction mixture was diluted with UltraPure™ distilled water (150 μL) and subjected to LC-MS analysis.

The conversion of DNA-conjugated products was estimated based on the integrated peak areas in the total ion chromatogram, without correction for differences in UV absorbance coefficients among the products and under the assumption of complete DNA recovery.

### 3.4. General Procedure for the On-DNA CuAAC Reaction

Lyophilized azide-conjugated DNA in UltraPure™ distilled water (500 mM, 10 μL) was added an alkyne derivative (200 mM in DMSO, 4 μL), phosphate buffer (0.1 M, pH 7.0, 2.4 μL), TBTA ligand (25 mM in DMSO, 1.6 μL), Cu(OAc)_2_ (50 mM in UltraPure™ distilled water, 1.6 μL), and sodium ascorbate (50 mM in UltraPure™ distilled water, 1.6 μL). The reaction mixture was incubated at 40 °C for 150 min with shaking. Afterward, sodium diethyldithiocarbamate (Na-DTC, 2000 mM in UltraPure™ distilled water, 2 μL) was added to quench the reaction, followed by incubation for an additional 30 min at room temperature. The mixture was then centrifuged at 12,000 rpm for 5 min at 4 °C. An aliquot of the supernatant (5 μL) was diluted with UltraPure™ distilled water (150 μL) and subjected to LC-MS analysis.

### 3.5. General Procedure for the Reduction of On-DNA Azide

Lyophilized azide-conjugated DNA in UltraPure™ distilled water (500 mM, 20 μL) was added, Tris-HCl buffer (0.1 M, pH 8.0, 42 μL) and TPPTS (200 mM in UltraPure™ distilled water, 8.0 μL) were added sequentially. The resulting mixture was incubated at 40 °C overnight with shaking. Following the reaction, the mixture was centrifuged at 12,000 rpm for 5 min at 4 °C. An aliquot of the supernatant (5 μL) was diluted with UltraPure™ distilled water (150 μL) and analyzed by LC-MS.

### 3.6. General Procedure for T4 Ligase Ligation

Lyophilized linker-conjugated DNA headpiece (500 nmol) was dissolved in UltraPure™ distilled water (740 μL). To this solution were added primer (0.986 mM each of top and bottom primers in distilled water, 507 μL), 10× T4 DNA ligase buffer (140 μL), ATP (100 mM in H_2_O, 4 μL) and T4 DNA ligase (30 units/μL, 10 μL). The mixture was incubated at 21 °C overnight. After the reaction, 5 M NaCl (150 μL) and cold ethanol (3.75 mL) were sequentially added. The mixture was vortexed, briefly centrifuged, and stored at −80 °C for 1 h. The sample was then centrifuged at 10,000× *g* for 30 min at 4 °C to remove the supernatant. The resulting pellet was re-dissolved in UltraPure™ distilled water and purified by column chromatography.

Column: Proteonavi (Osaka Soda) (OSAKA SODA Co., Ltd., Umeda, Osaka, Japan), 5 µm, 20 mm I.D. × 50 mm

Eluent: (A) 50 mM triethylamine-acetic acid buffer, pH 7.5, (B) acetonitrile:H_2_O = 1:1 (*v*/*v*). Gradient: (B) 0–1 min; 0–10%, 1–2 min; 10%, 2–3 min; 10–20%, 3–5 min; 20%, 5–8 min; 20–100%, 8–9 min; 100%. Flow rate: 20 mL/min. Column temperature: 25 °C. Detection: UV 260 nm

### 3.7. qPCR Analysis

Quantitative PCR (qPCR) was performed using the Applied Biosystems QuantStudio™ 7 Flex Real-Time PCR System (Thermo Fisher Scientific Inc., Waltham, MA, USA) with 96-well plates. Each 10 μL reaction mixture consisted of 3 μL of nuclease-free water, 0.8 μL of primer mix (5 μM each of forward and reverse primers), 1 μL of diluted DNA sample, 0.2 μL of ROX reference dye II and 5 μL of TB Green Premix Ex Taq II.

The thermal cycling conditions were as follows: initial denaturation at 94 °C for 30 s, followed by 40 cycles of 95 °C for 10 s, 60 °C for 20 s, and 72 °C for 20 s. Each qPCR reaction was performed in triplicate.

## 4. Conclusions

Traditionally, drug discovery efforts have primarily focused on targets with well-defined binding pockets, such as enzymes and G protein-coupled receptors. However, in recent years, the scope of druggable targets has expanded to include challenging targets, such as proteins involved in protein–protein interactions (PPIs) and those modulated through allosteric mechanisms. These targets often lack clearly defined binding sites, making it difficult to identify high-quality hit compounds using conventional high-throughput screening libraries.

To effectively address such challenging targets, it is necessary to explore a broader and more diverse chemical space, beyond that occupied by conventional small molecules, to efficiently identify potential drug candidates. Targeting PPIs requires compounds with larger molecular weights (often MW > 500) capable of covering flat and extensive interaction interfaces that typically lack well-defined binding pockets. As such, middle molecules (MW > 500) are considered promising for these applications.

In the present study, we designed and synthesized amino acid-derived on-DNA diazide compounds, 4N_3_-BA-(2S)-N_3_-homoAla-HP, 4N_3_-BA-(2*S*)-N_3_-Lys-HP, and 4N_3_-BA-(2*S*,4*S*)-N_3_-Pro-HP, as new platform molecules for DEL construction. Furthermore, a virtual DEL was generated based on 4N_3_-BA-(2*S*,4*S*)-N_3_-Pro-HP, and its chemical space was analyzed. The resulting library was found to occupy unexplored regions of chemical space that are distinct from those covered by existing small-molecule libraries derived from ChEMBL. This coverage suggests the potential of the library to yield novel binders with diverse chemical structures.

The compounds contained within the virtual DEL exhibited an average molecular weight of approximately 650, placing them within the extended Rule-of-5 space that lies between traditional small molecules and middle molecules. Additionally, the compounds had relatively high lipophilicity (clogP = 2.4–4.4) (see Supporting Information), consistent with favorable compatibility with the hydrophobic interfaces that are characteristic of many PPIs. These properties strongly support the potential utility of the DEL in discovering novel binders against challenging targets.

We are currently planning to prepare for the practical synthesis of DELs based on on-DNA diazides, such as 4N_3_-BA-(2*S*,4*S*)-N_3_-Pro-HP, with the aim of realizing their full application in drug discovery.

## Data Availability

Data is contained within the article and Supplementary Materials.

## Supplementary Materials

The following supporting information can be downloaded at: https://www.mdpi.com/article/10.3390/ijms26199501/s1.
