# Supplementary material for: Development of an On-DNA Platform Molecule Bearing a Diazidestructure and Its Application to DEL Synthesis
Source: Int J Mol Sci. 2025 Sep 28;26(19):9501. doi: 10.3390/ijms26199501 (PMC12524918; doi:10.3390/ijms26199501)

SUPPORTING INFORMATION

Hiroyuki Miyachi[1]*, Masaki Koshimizu[1], Manussada Ratanasak[2], Yasuteru Shigeta[2], and Masashi Suzuki[1]

[1] Lead Exploration Unit, Drug Discovery Initiative, University of Tokyo, 3-1 Hongo,Bunkyo, Tokyo 113-0033, Japan

[2] Center for Computational Sciences, University of Tsukuba, 1-1 Tennodai, Tsukuba, Ibaraki 305-8577, Japan

Organocatalyzed [3+2] Cycloaddition Reactions of On-DNA Organic Azides

Design of a Novel On-DNA Platform Molecule: N-(4-Azidobenzoyl)-azidohomoalanine-HP (hALA-based DAP)

Stepwise Construction of On DNA compounds Using 4N_3-BA-(*S*)-N_3-homoALA–HP (Double-Click Strategy)

Development of on-DNA Azide–Amine Platform Molecules: 4N_3-BA-(S)-N_3-LYS–HP and (2S,4S)-4N_3-BA-(S)-N_3-PRO–HP

DEL Synthesis Using 4N_3-BA-(2S,4S)-N_3-PRO–HP: On-DNA Di-Azide and Azide–Amine Platforms

Validation of the Practical Utility of on-DNA DAP via Mock DNA-Encoded Pool Synthesis

Assessment of DNA Damage During the Full-Length on-DNA Synthesis of DAP Compounds

Chemical Space Analysis of a Virtual DEL Originating from 4N_3-BA-(2S,4S)-N_3-PRO-HP

I. General Information

Unless otherwise noted, materials, DNA headpiece (HP-NH2) (5'- / 5phos / GAGTCA / iSp9 / iUniAmM / iSp9 / TGACTCCC-3', Figure S1) and solvents obtained from commercial suppliers were used without further purification. All on-DNA reactions were performed in 0.2 mL PCR tube or 1.5mL / 2.0 mL micro tubes. On-DNA reactions in the studies of reaction condition optimization and substrate scope extension were analyzed by UPLC-MS. Typically, 1.0 uL samples were dissolved in an appropriate amount of UltraPure™ distilled water and injected into a reverse-phase chromatography column (Waters XBridge Oligonucleotide BEH C18 column, 1.7 μm, 2.1 × 50 mm) at 60° C. The elution was carried out as followings: 10–90% solvent B over 4.5 min, 0.4 mL/min, λ = 260 nm; solvent A: water / 1,1,1,3,3,3-hexafluoro-2-propanol / triethylamine = 100 / 2 / 0.1 (v/v); solvent B: methanol / 1,1,1,3,3,3-hexafluoro-2-propanol / triethylamine / water = 100 / 2 / 0.1 / 2 (v/v). The effluents were analyzed by a Xevo G2-XS Q-TOF with electrospray ionization source was used for detection.
On DNA reaction yield calculation: Ignoring UV coefficient difference for all on DNA products and assuming 100% of DNA total recovery, the yield of DNA products was determined from total ion chromatography peak area.

II. Abbreviations

DBU: 1,8-diazabicyclo[5.4.0]undec-7-ene
DIPEA: N,N-diisopropylethylamine
DMSO: dimethyl sulfoxide
DMTMM BF_4: 4-(4,6-Dimethoxy-1,3,5-triazin-2-yl)-4-methylmorpholinium tetrafluoroborate
HATU: 2-(7-Azabenzotriazol-1-yl)-N,N,N',N'-tetramethyluronium hexafluorophosphate
LC-MS: Liquid Chromatography-Mass spectrometry
Na DTC: Sodium diethyldithiocarbamate
TBTA: Tris[(1-benzyl-1*H*-1,2,3-triazol-4-yl)methyl]amine
TCEP: Tris(2-carboxyethyl)phosphine
TPPTS: 3,3′,3″-Phosphanetriyltris(benzenesulfonic acid) trisodium salt
Tris HCl: tris(hydroxymethyl)aminomethane hydrochloride

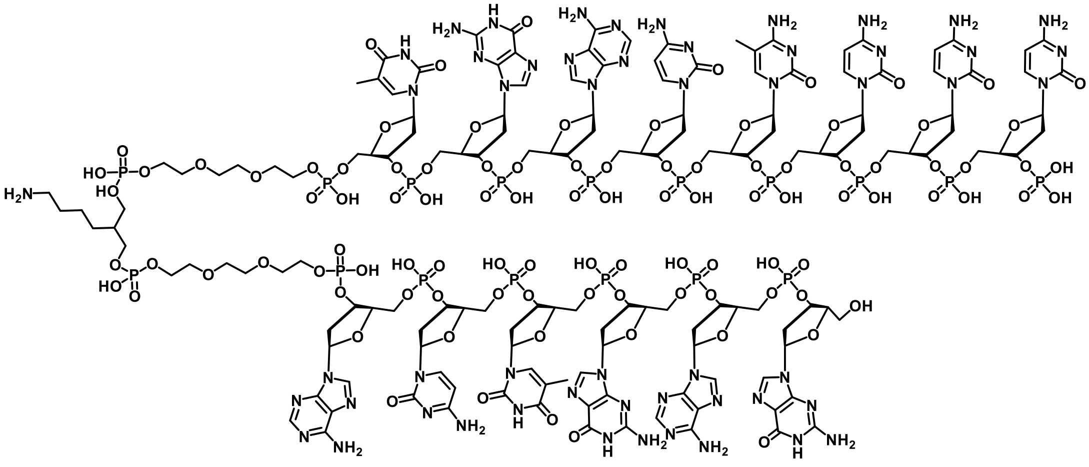

Chemical structure of HP (5'- / 5phos / GAGTCA / iSp9 / iUniAmM / iSp9 / TGACTCCC-3')

III. LC Trace and Mass of on-DNA compounds

Preparation of the starting on DNA-azides

1a

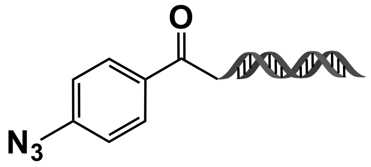

Conversion (Product%) = 100%
Base Peak Mass (Da): 5082.1

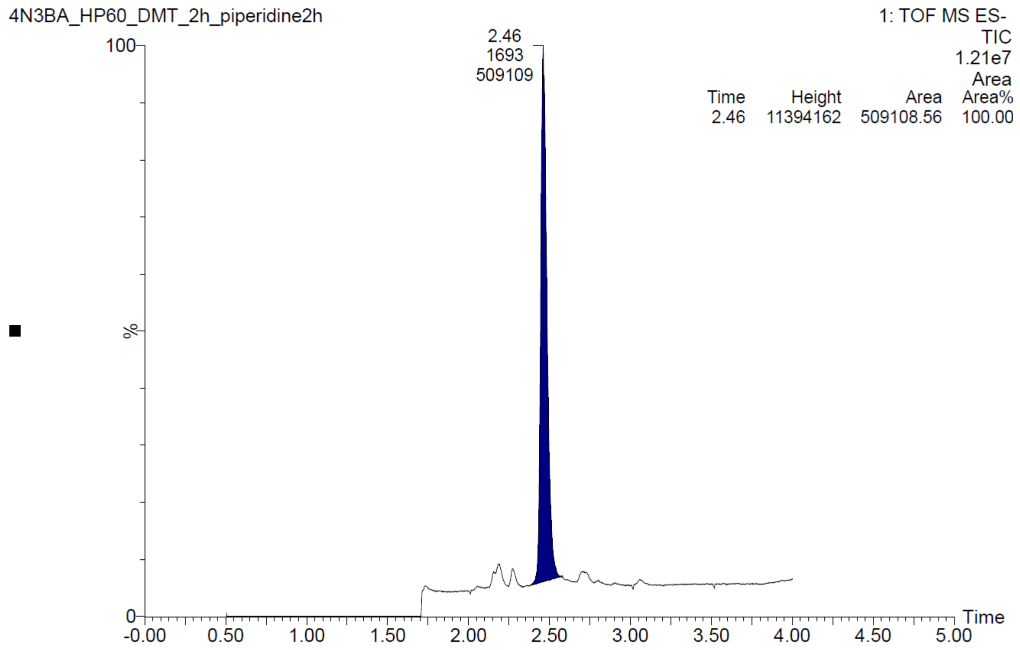

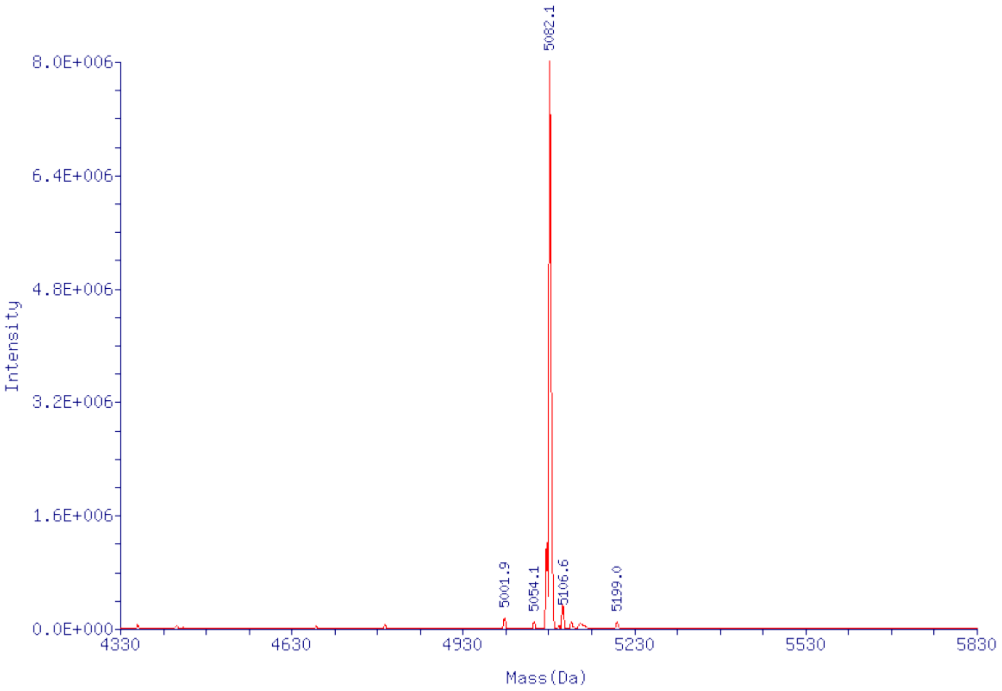

1b

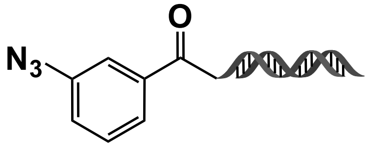

Conversion (Product%) = 100%
Base Peak Mass (Da): 5082.1

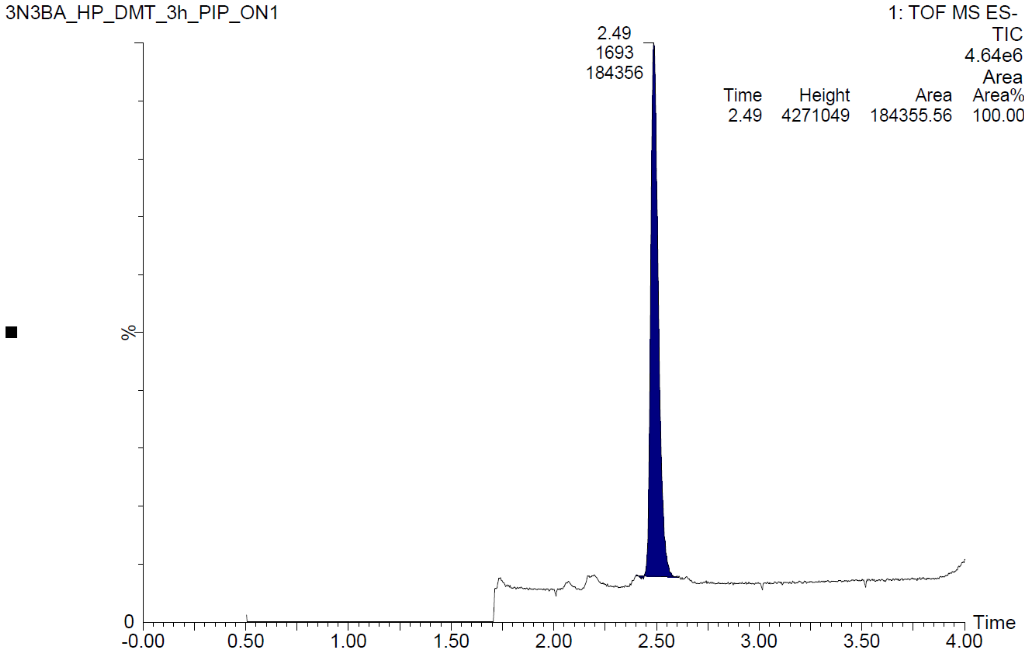

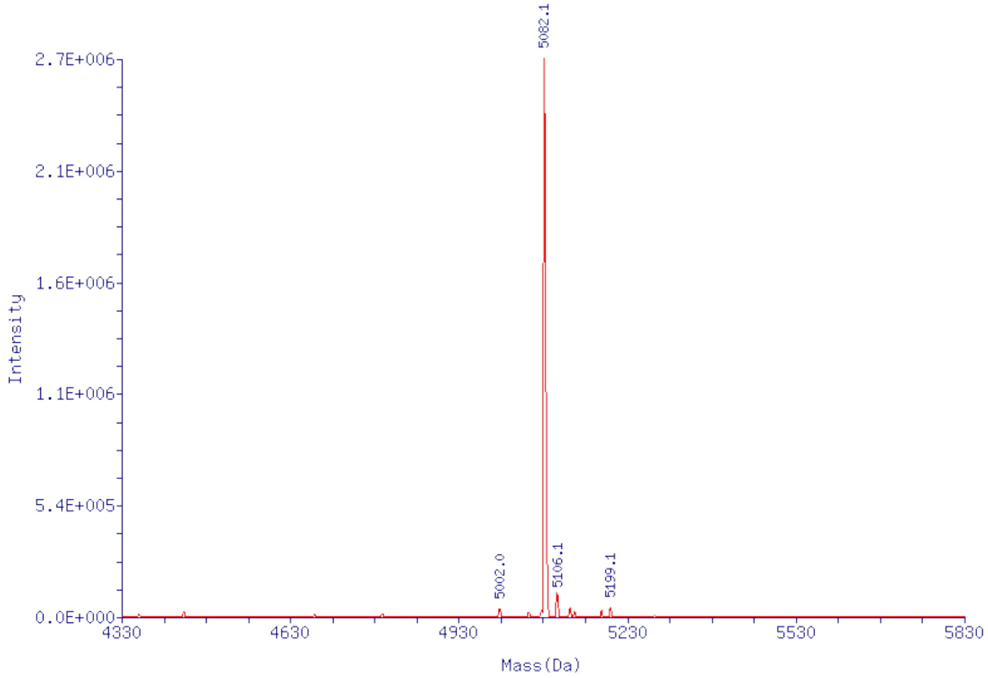

1c

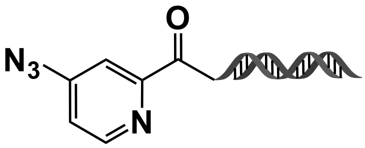

Conversion (Product%) = 100%
Base Peak Mass (Da): 5082.9

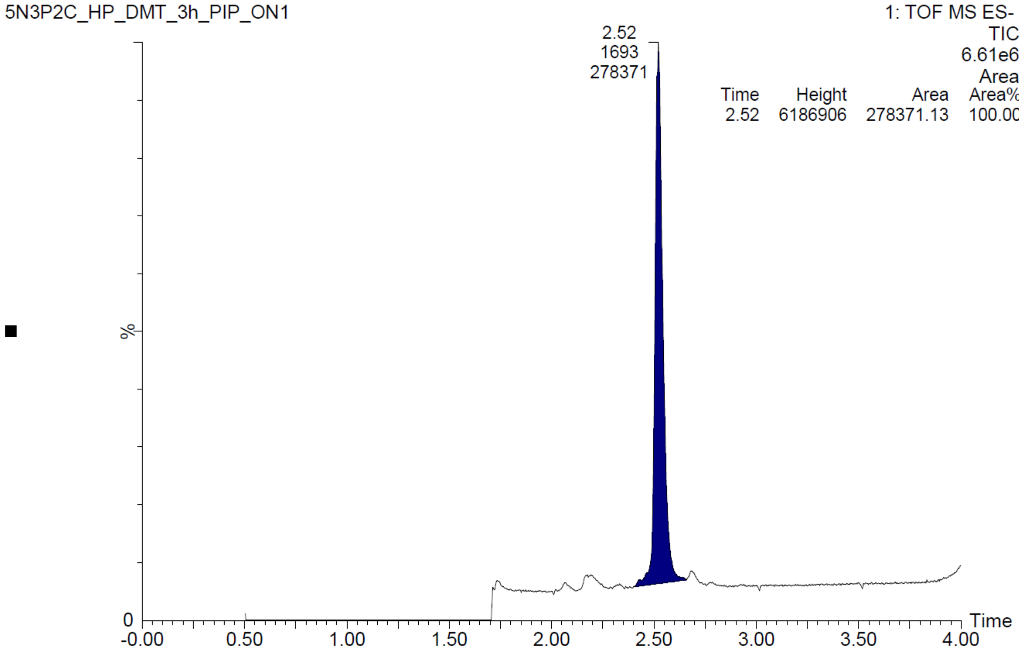

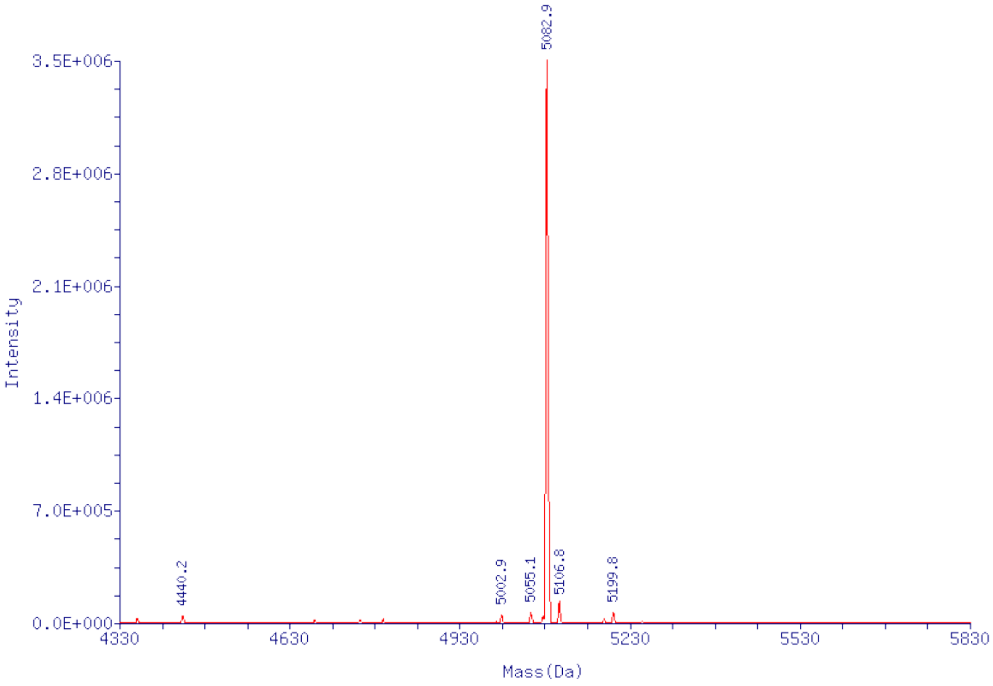

1d

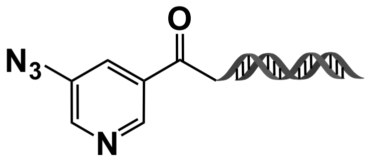

Conversion (Product%) = 100%
Base Peak Mass (Da): 5082.7

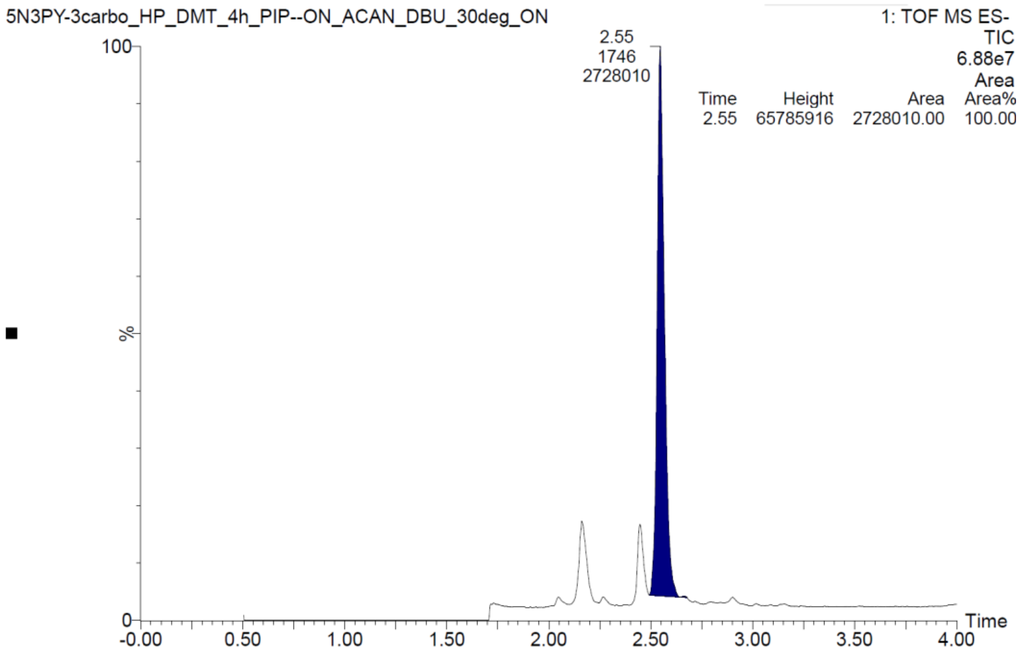

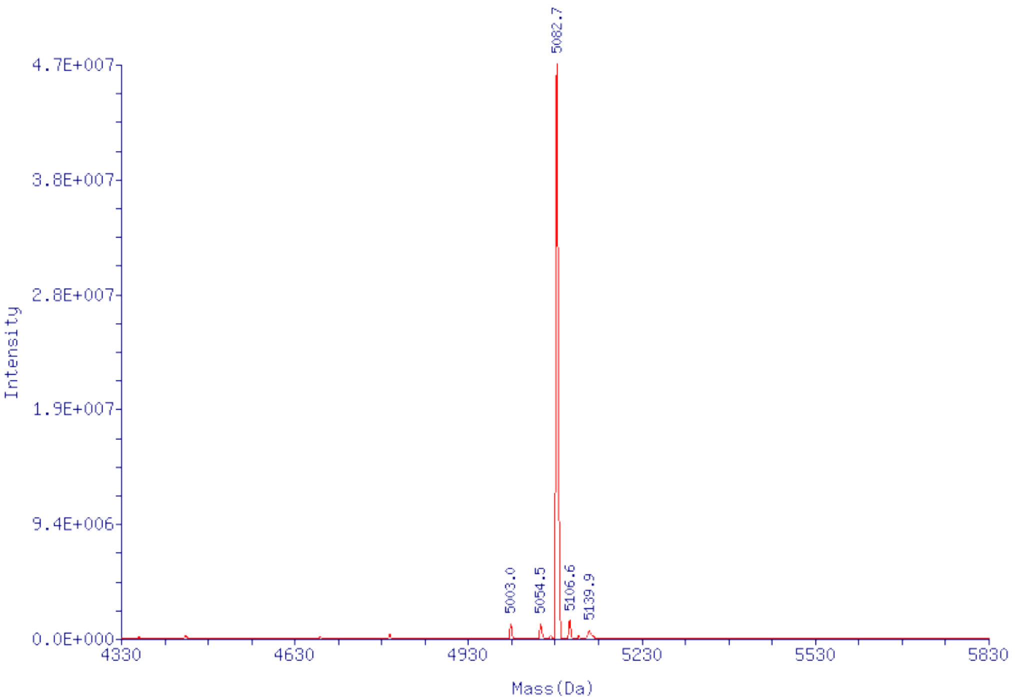

1e

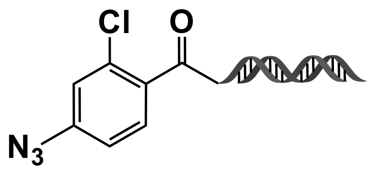

Conversion (Product%) = 96%
Base Peak Mass (Da): 5116.3

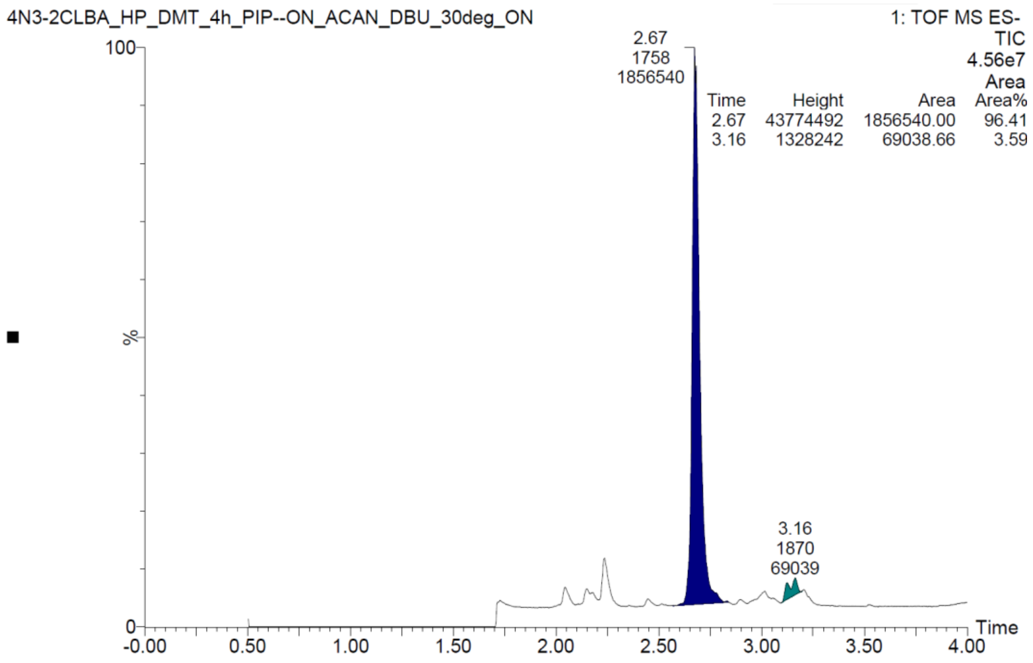

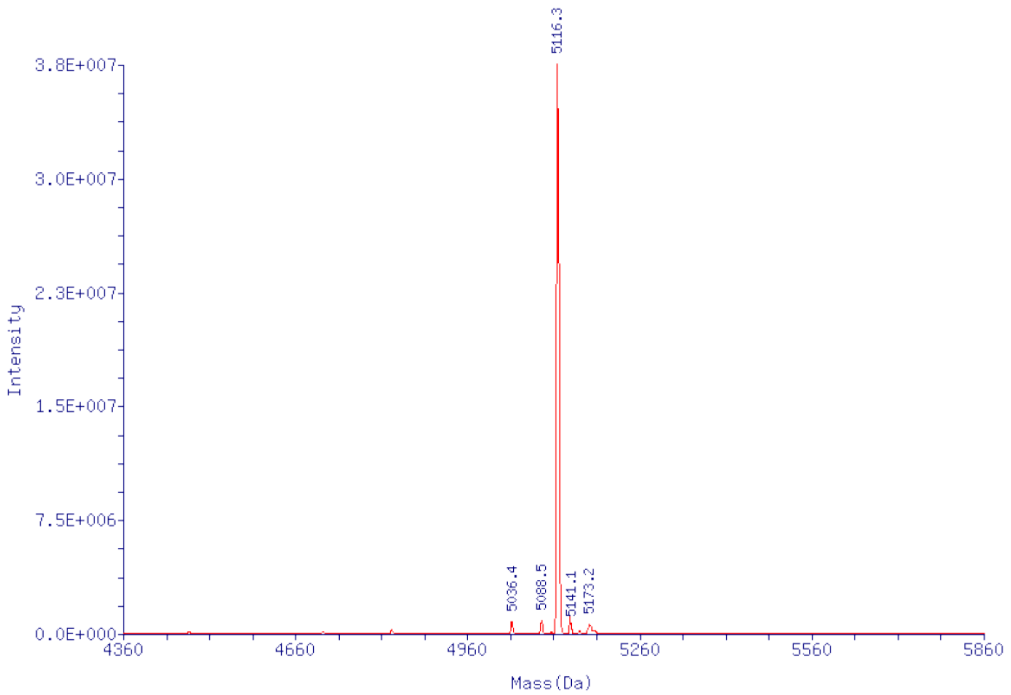

1f

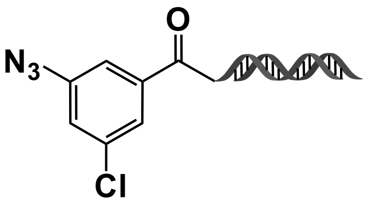

Conversion (Product%) = 100%
Base Peak Mass (Da): 5116.3

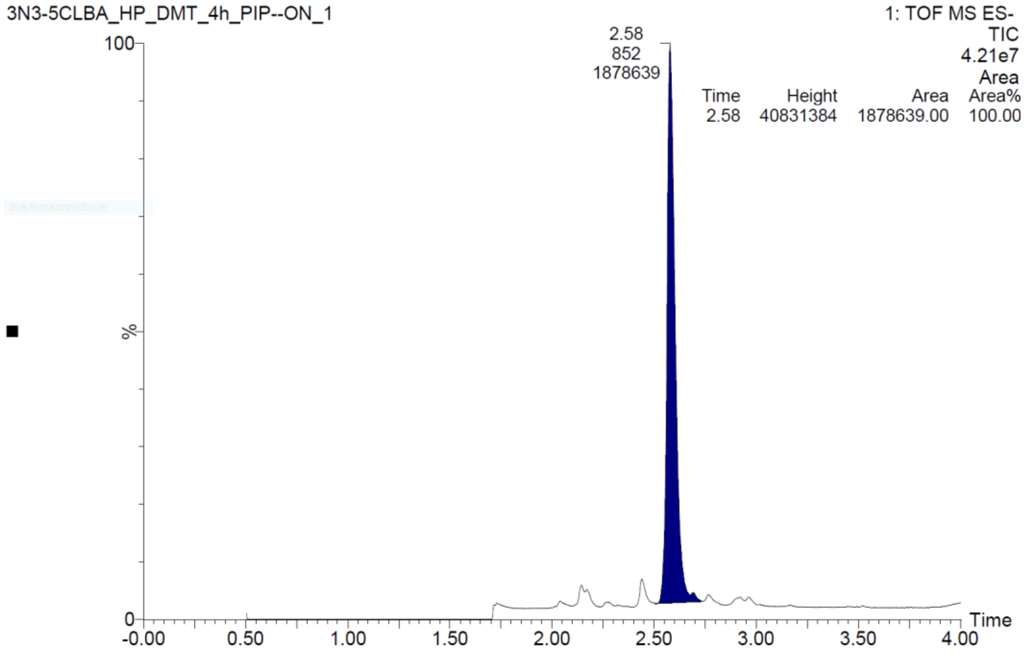

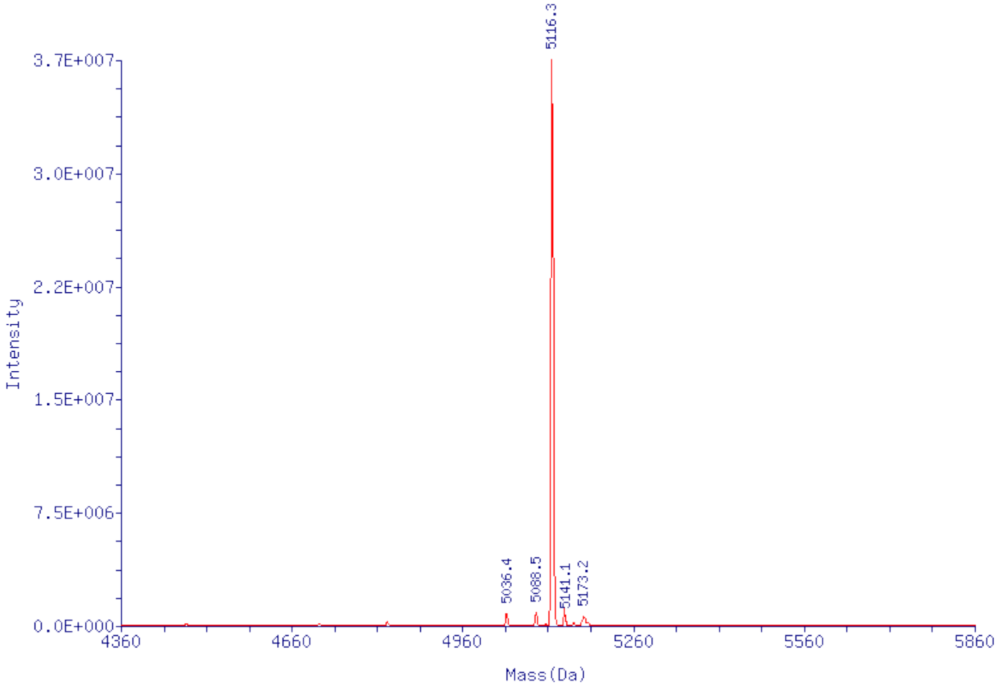

1g

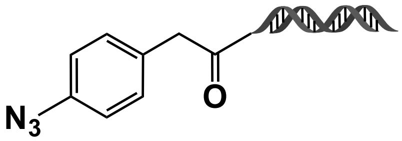

Conversion (Product%) = 59%
Base Peak Mass (Da): 5096.0

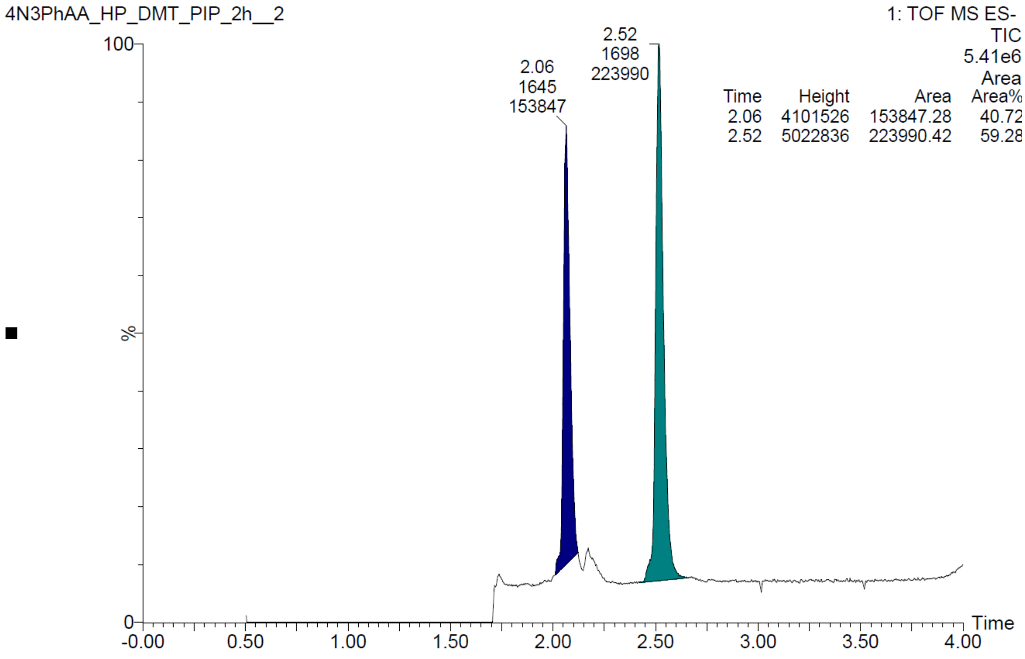

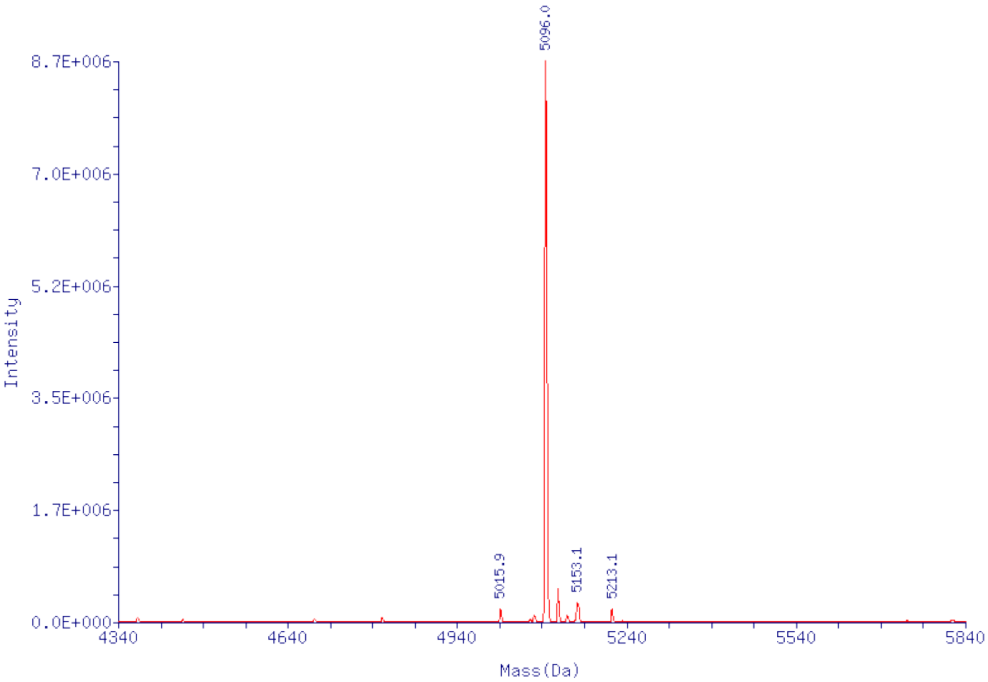

1h

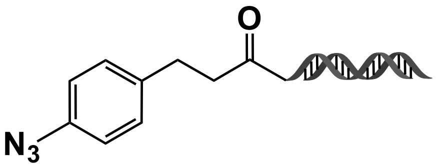

Conversion (Product%) = 100%
Base Peak Mass (Da): 5109.9

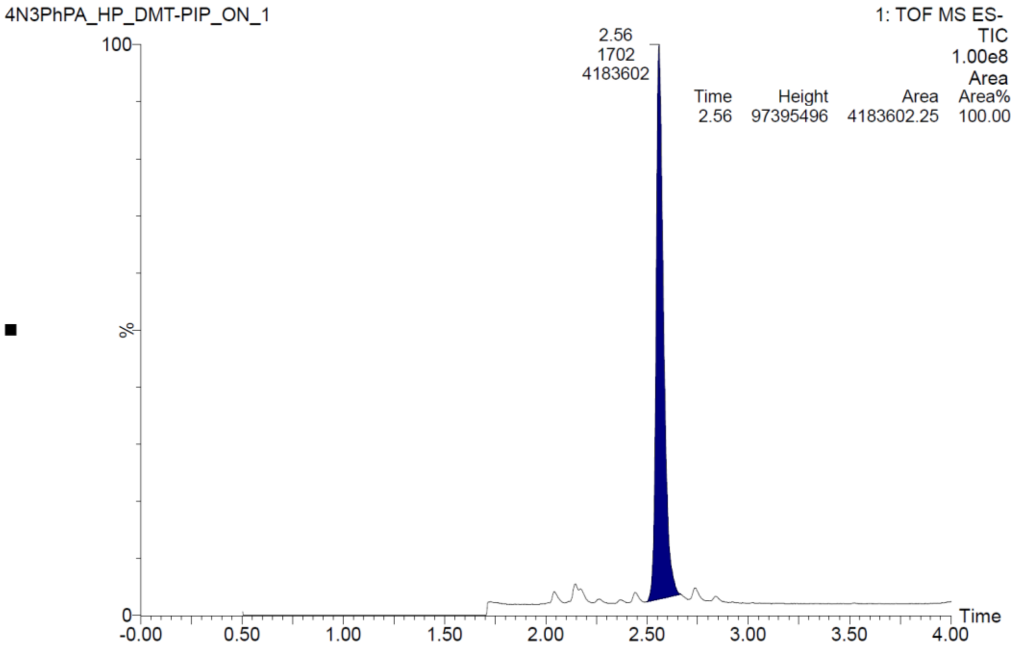

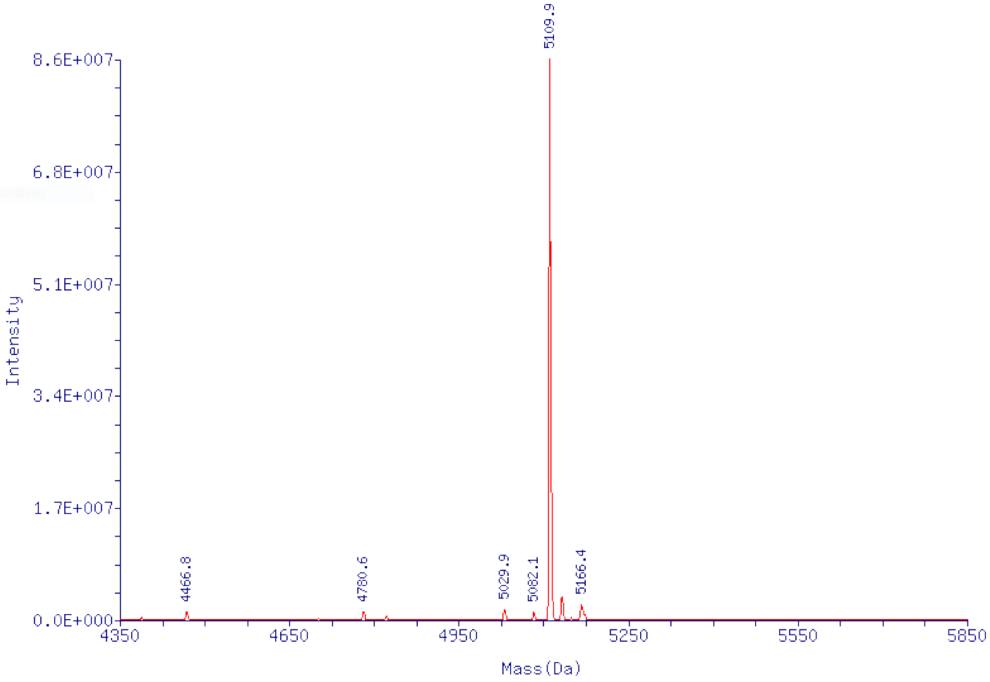

1i

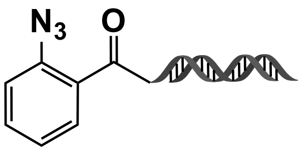

Conversion (Product%) = 89%
Base Peak Mass (Da): 5082.1

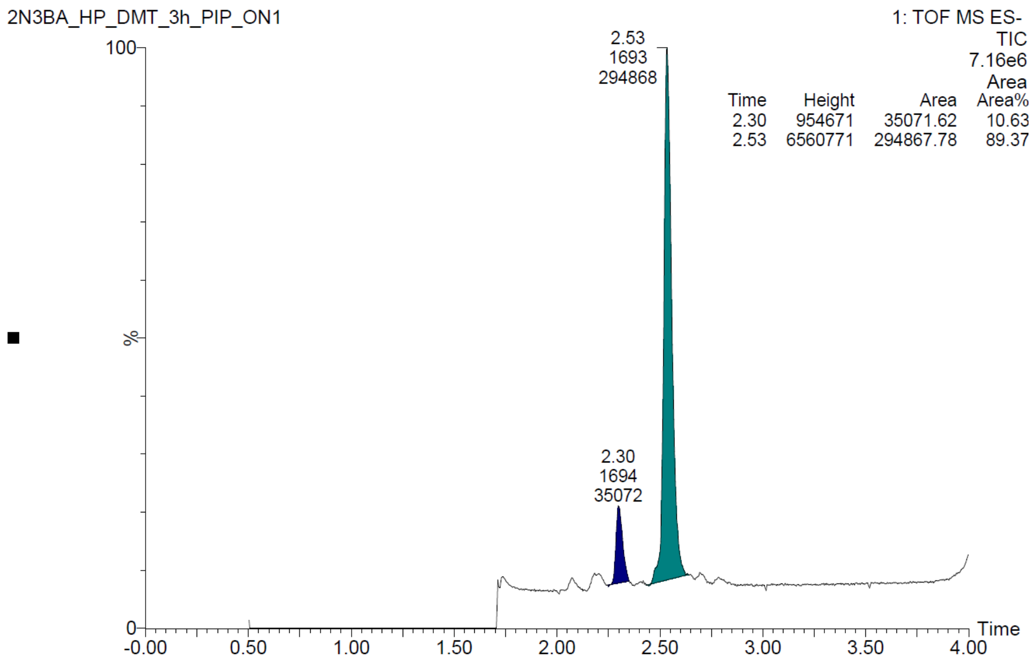

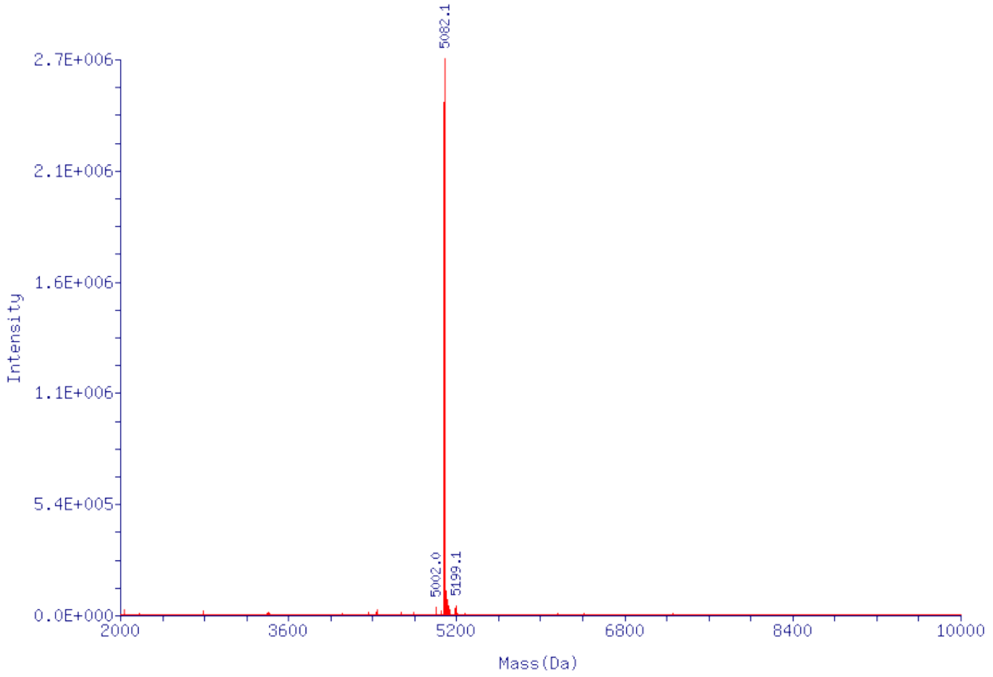

1j

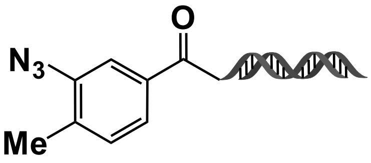

Conversion (Product%) = 93%
Base Peak Mass (Da): 5095.5

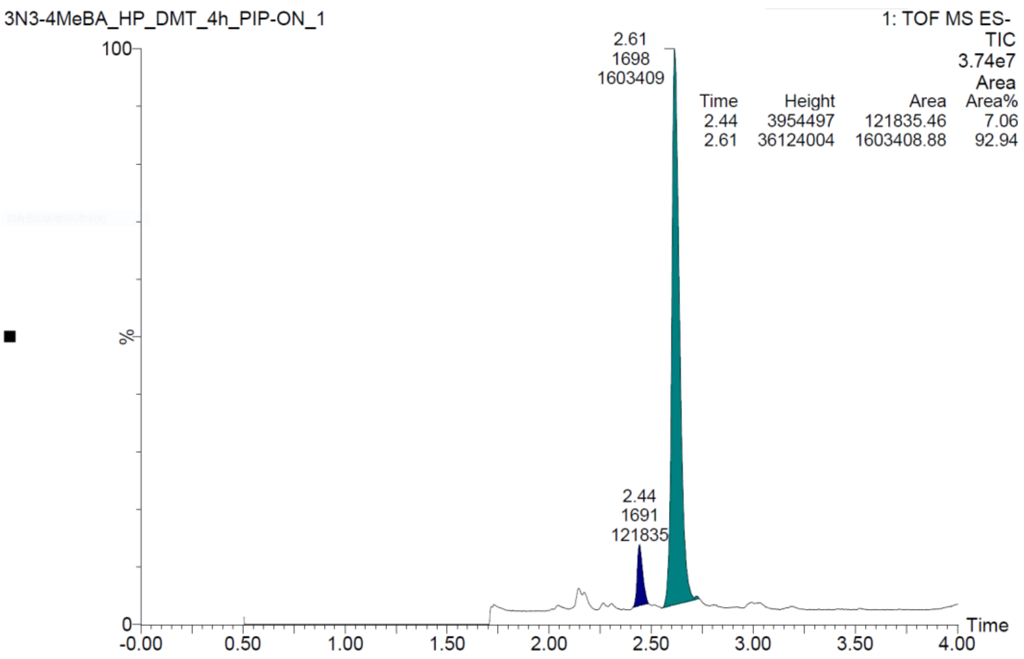

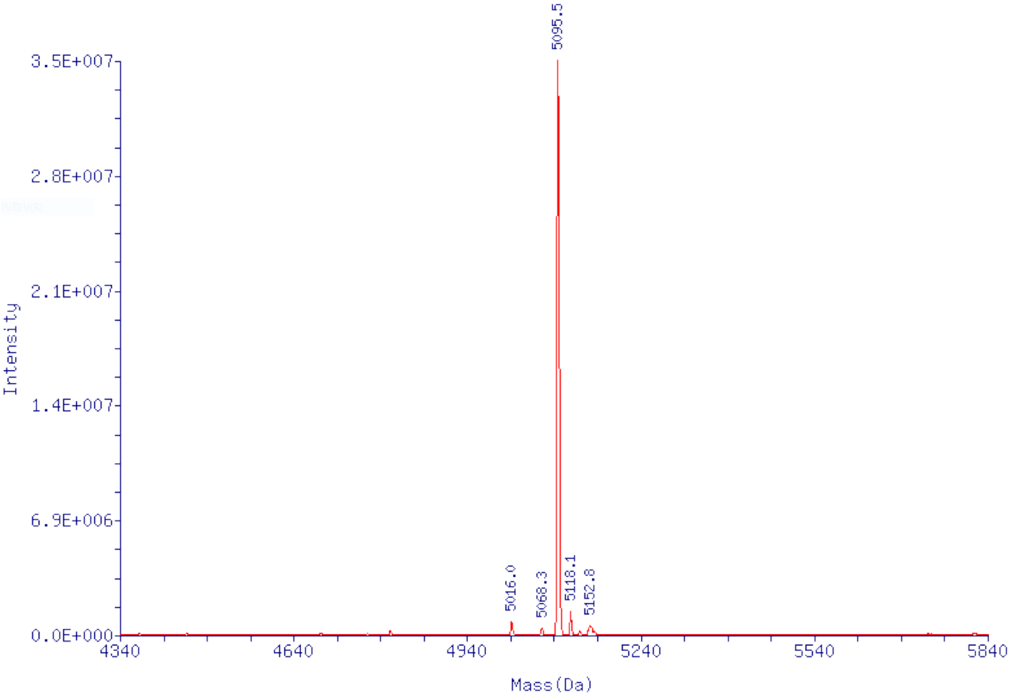

1k

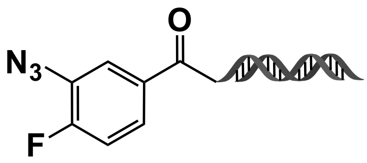

Conversion (Product%) = 95%
Base Peak Mass (Da): 5100.1

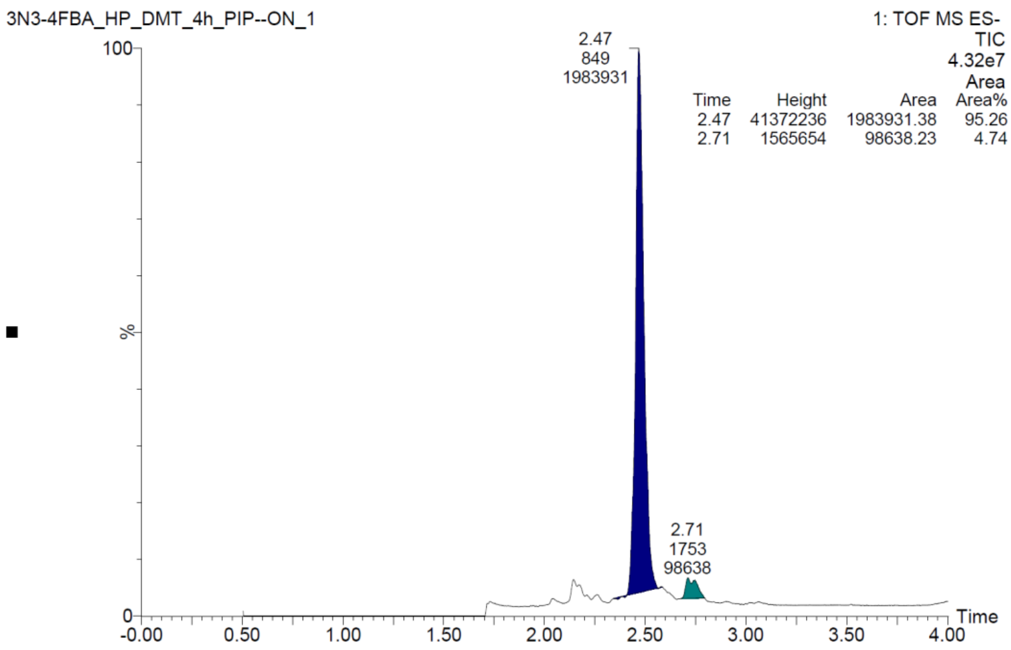

Time	Height	Area	Area%
2.47	41372236	1983931.38	95.26
2.71	1565654	98638.23	4.74

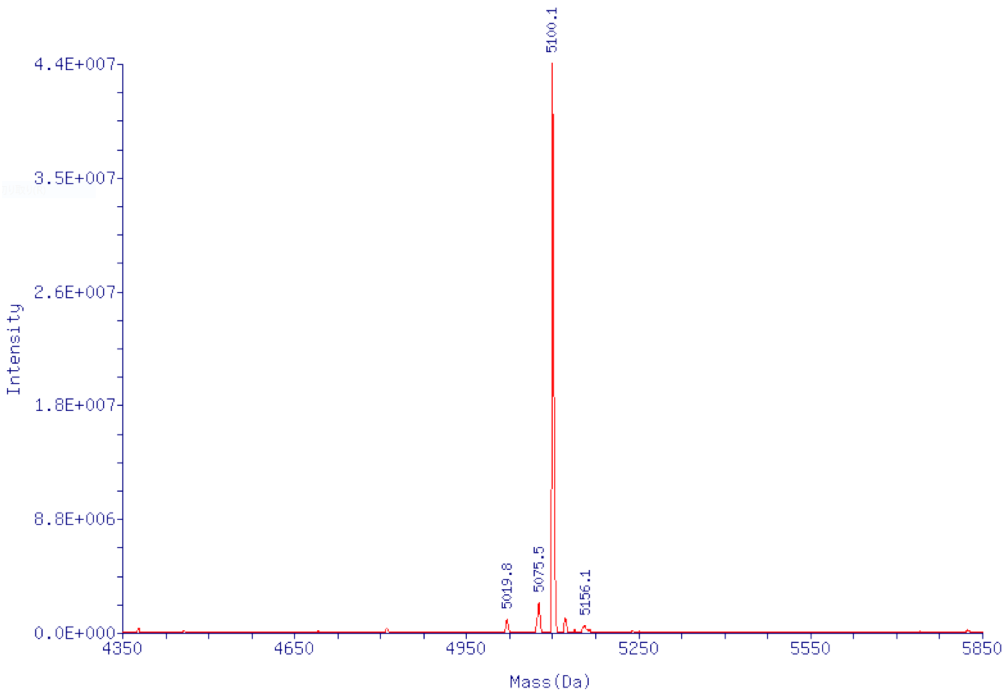

1l

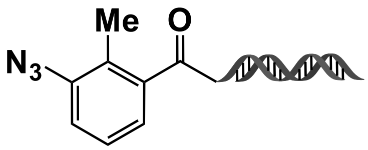

Conversion (Product%) = 79%
Base Peak Mass (Da): 5095.7

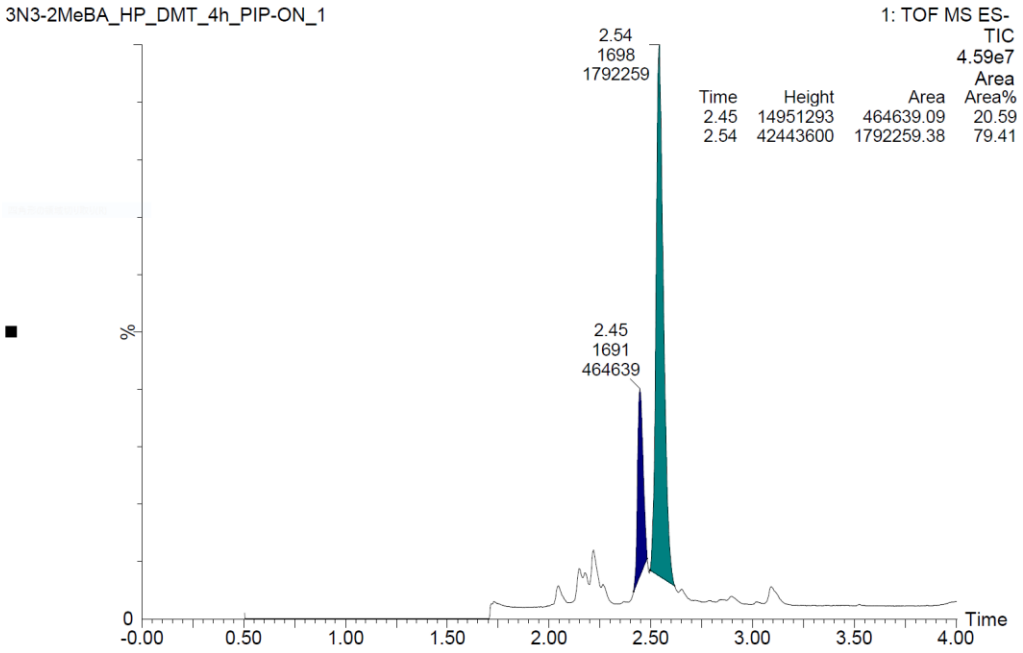

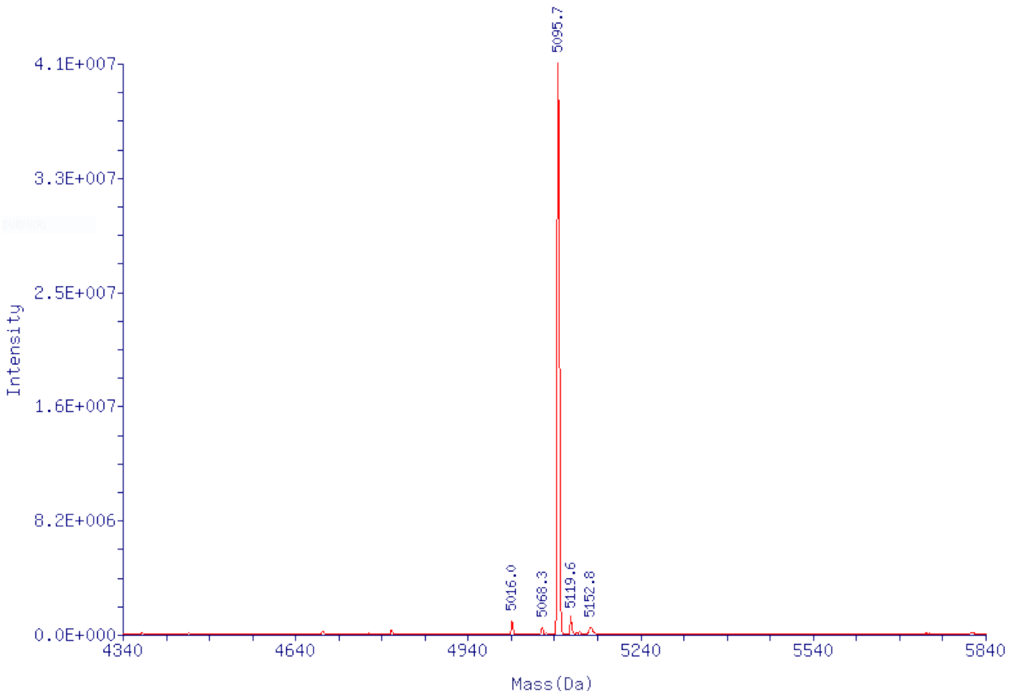

1m

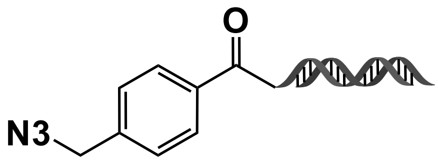

Conversion (Product%) = 100%
Base Peak Mass (Da): 5095.9

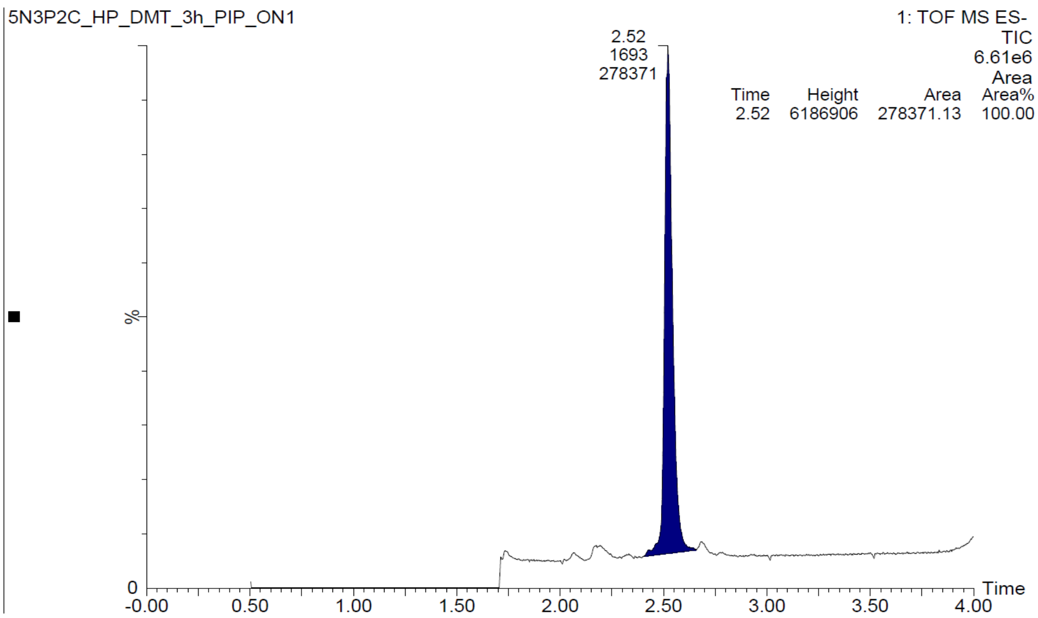

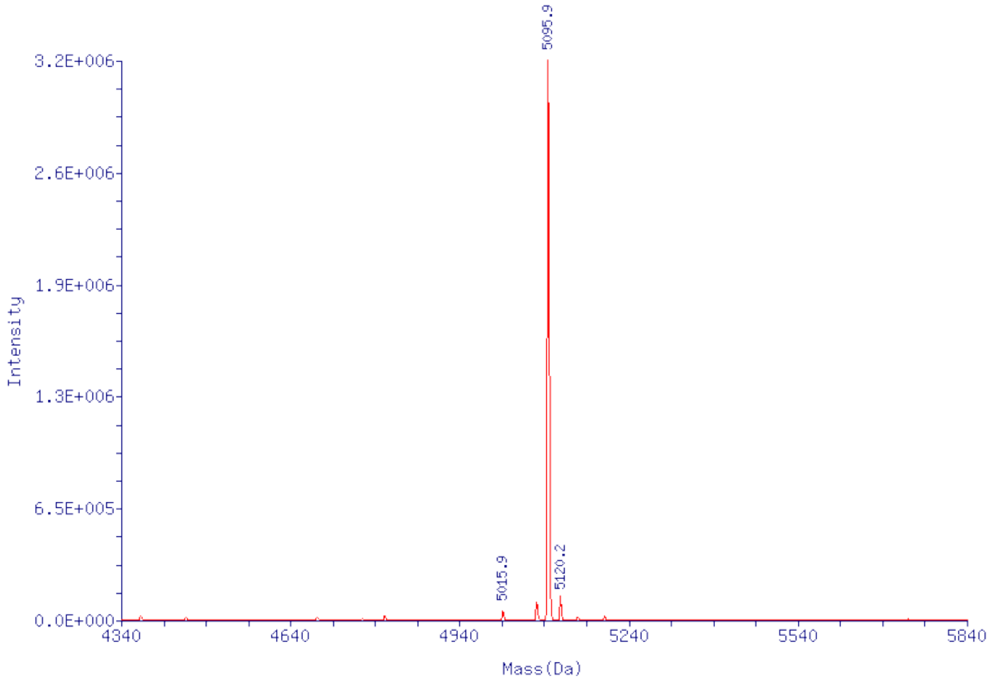

1n

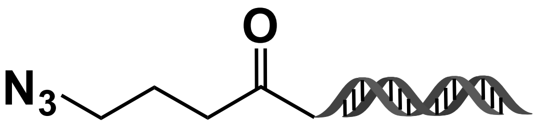

Conversion (Product%) = 100%
Base Peak Mass (Da): 5048.1

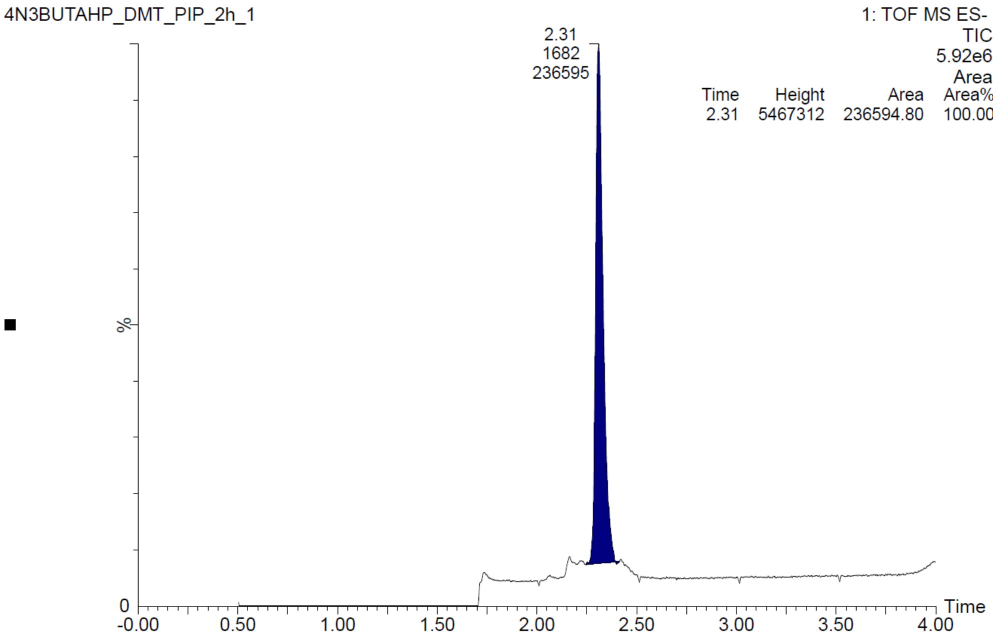

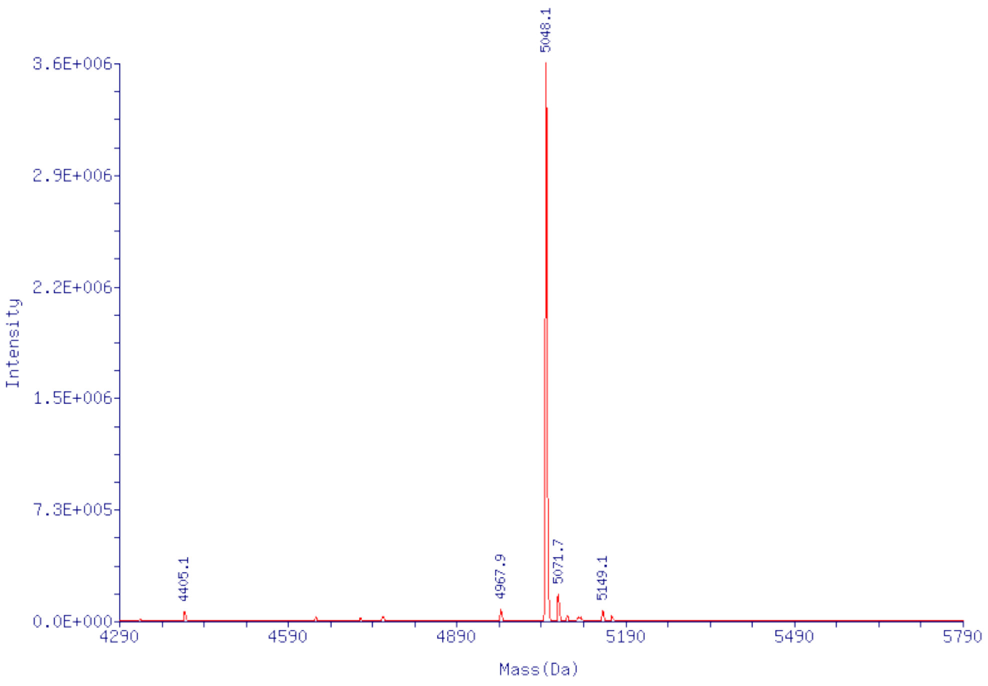

1o

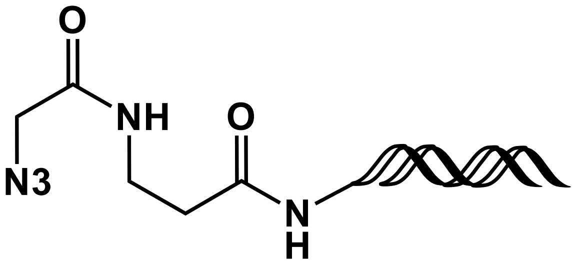

Conversion (Product%) = 100%
Base Peak Mass (Da): 5090.8

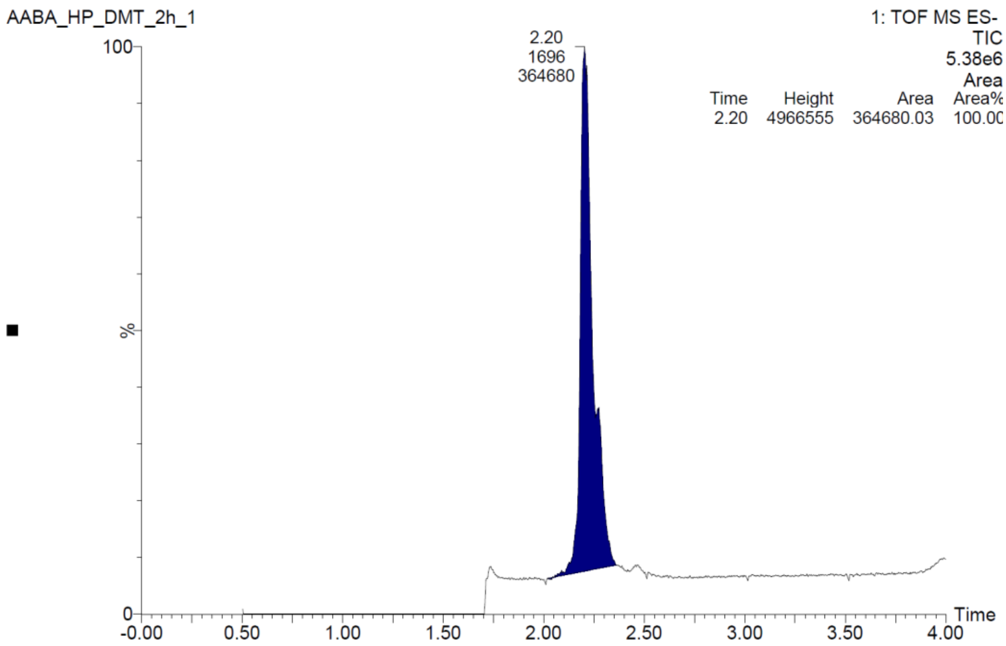

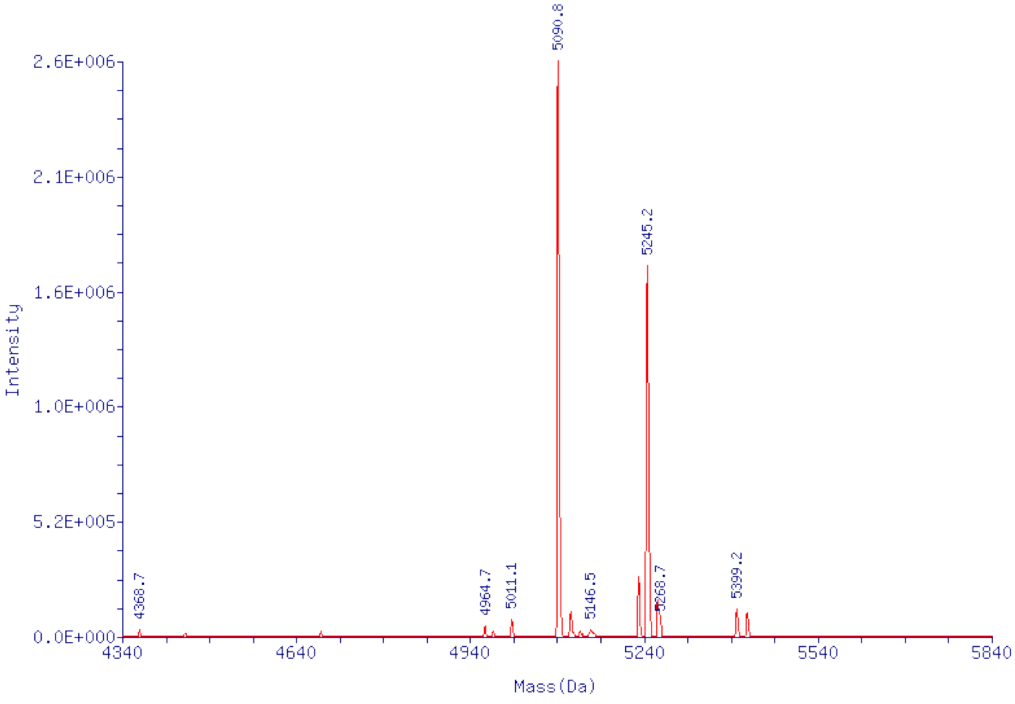

1p

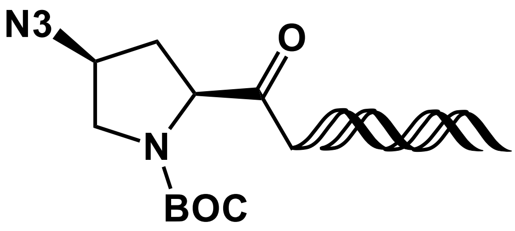

Conversion (Product%) = 100%
Base Peak Mass (Da): 5175.0

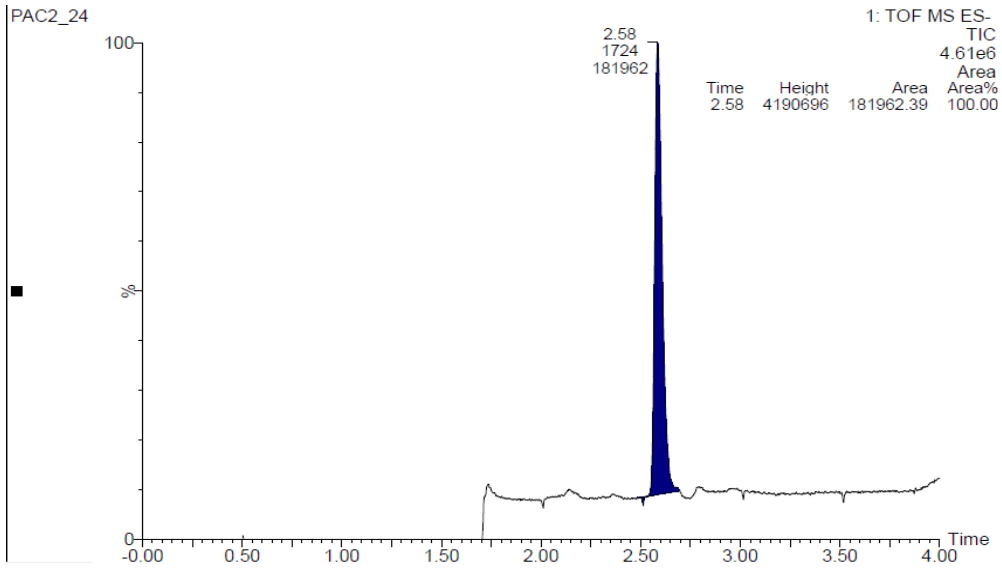

[<<] [Top] [ESI Mass Spectrum] [Deconvolution] [Deconvolution Peak Report] [View Data] [Log File]
Zoom Display Deconvoluted Mass Spectrum:

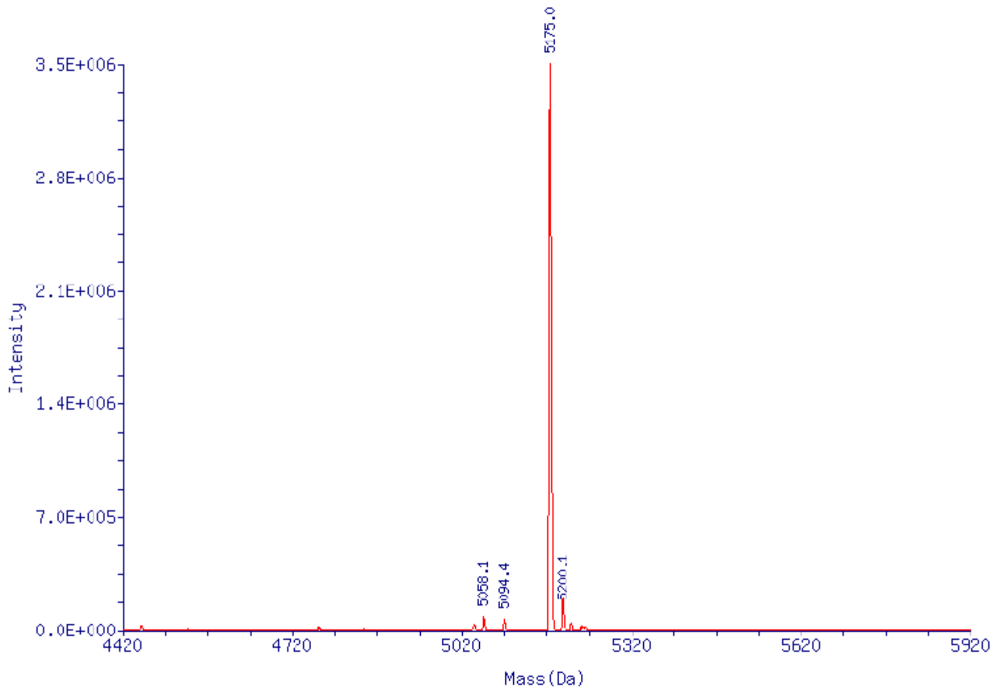

Organocatalyzed [3+2] Cycloaddition Reactions of On-DNA Organic Azides

1a-TM

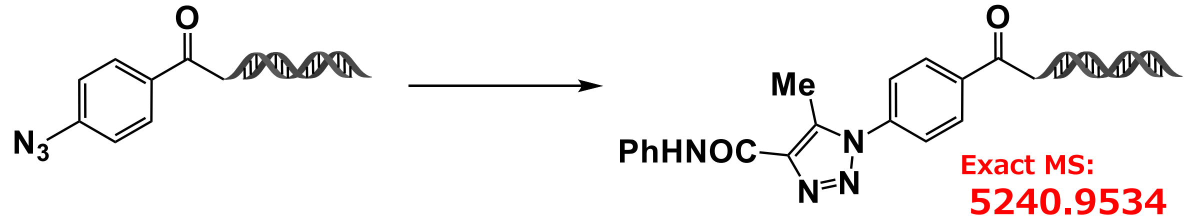

Conversion (Product%) = 88%
Base Peak Mass (Da): 5240.9

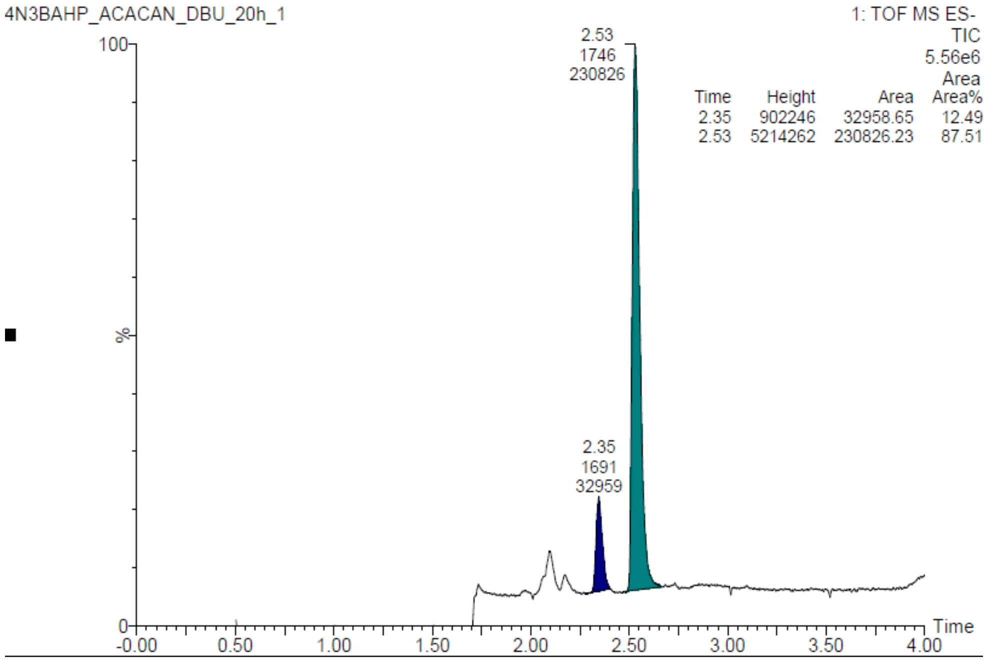

Time	Height	Area	Area%
2.35	902246	32958.65	12.49
2.53	5214262	230826.23	87.51

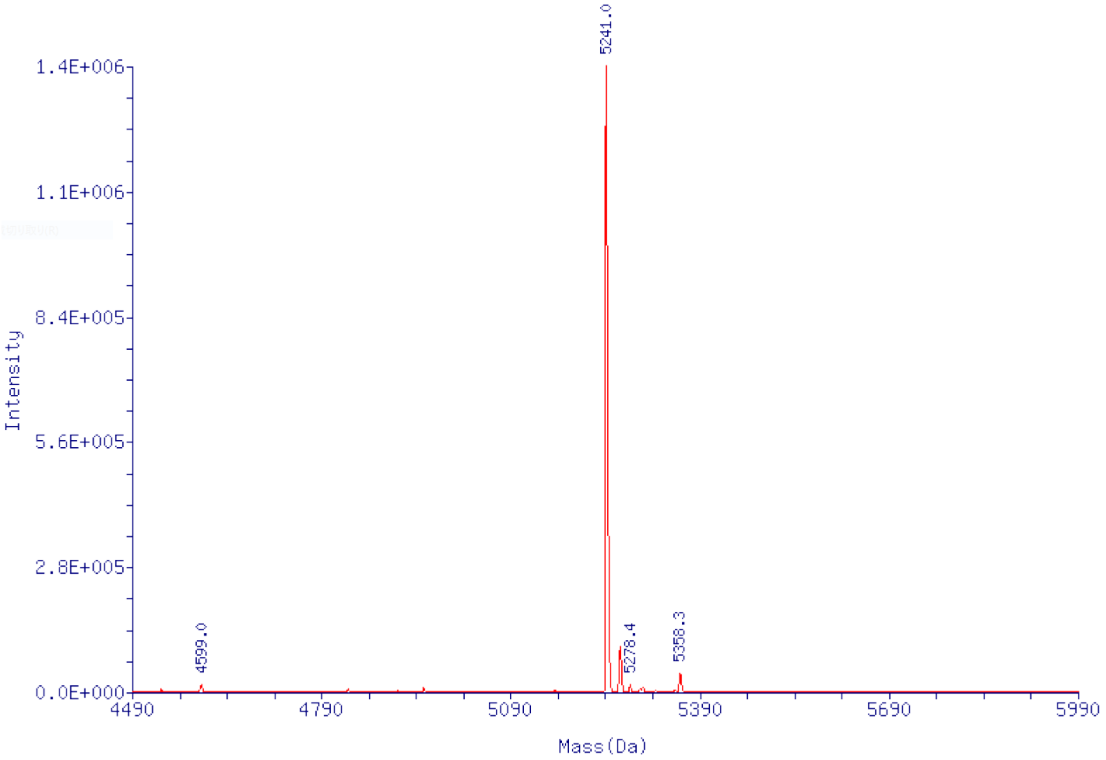

1b-TM

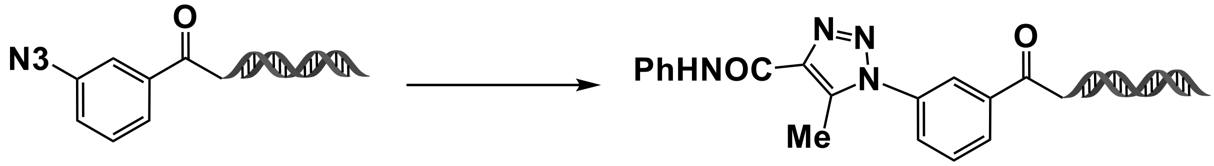

Exact MS:
5240.9534

Conversion (Product%) = 94%
Base Peak Mass (Da): 5240.9

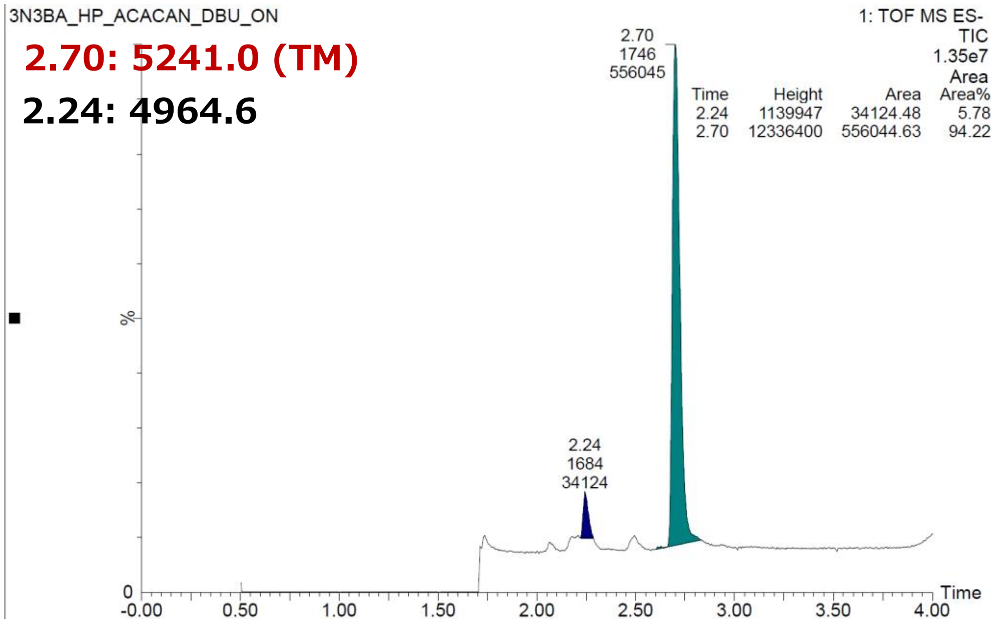

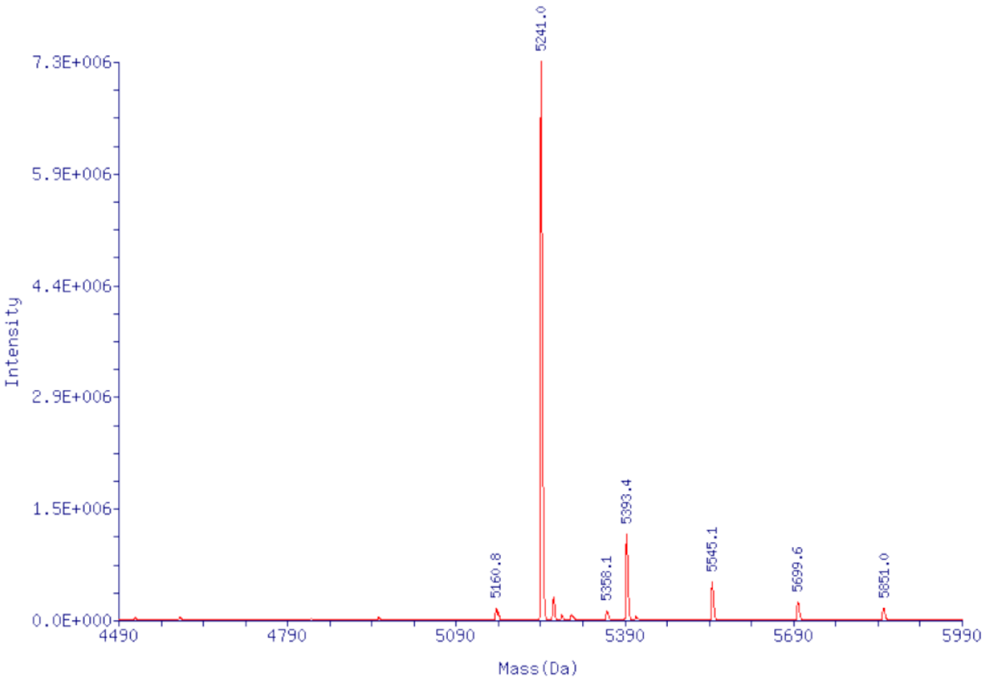

1c-TM

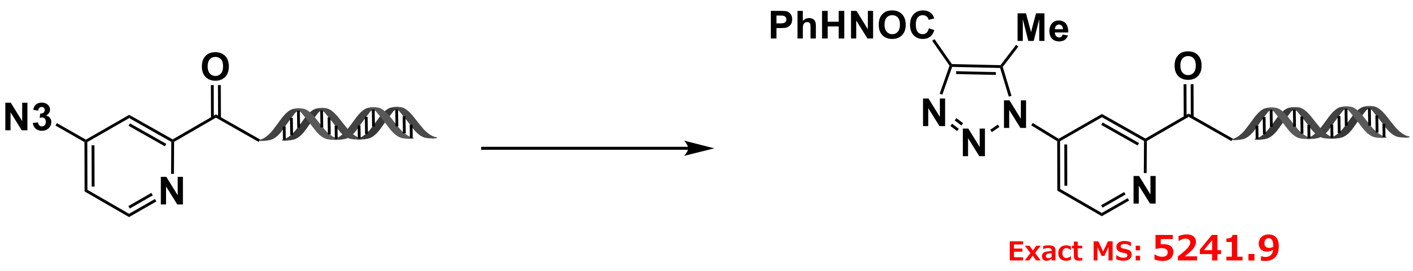

Conversion (Product%) = 97%
Base Peak Mass (Da): 5241.9

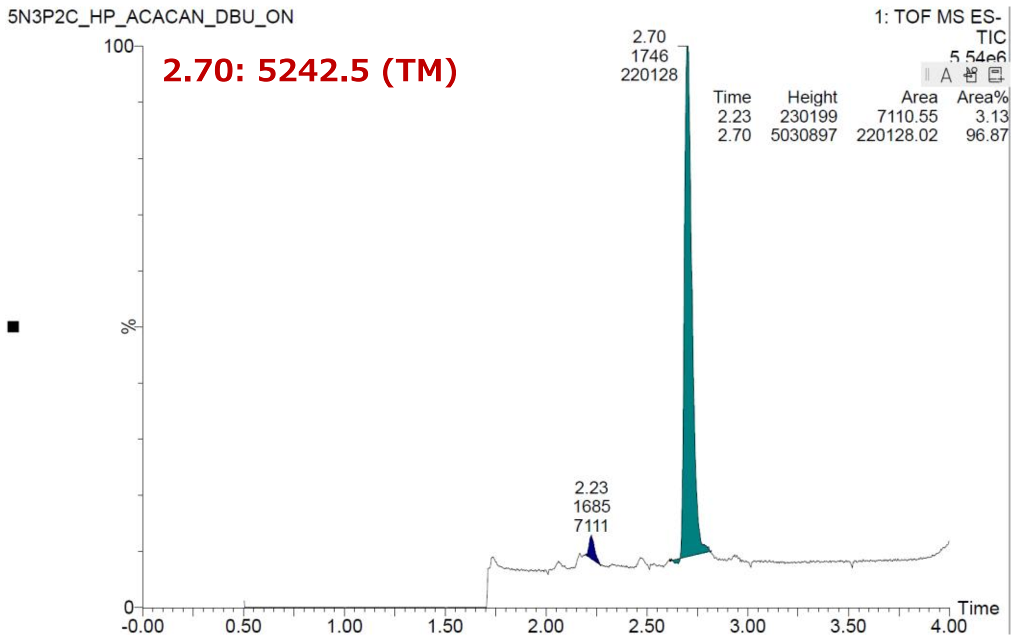

Time	Height	Area	Area%
2.23	230199	7110.55	3.13
2.70	5030897	220128.02	96.87

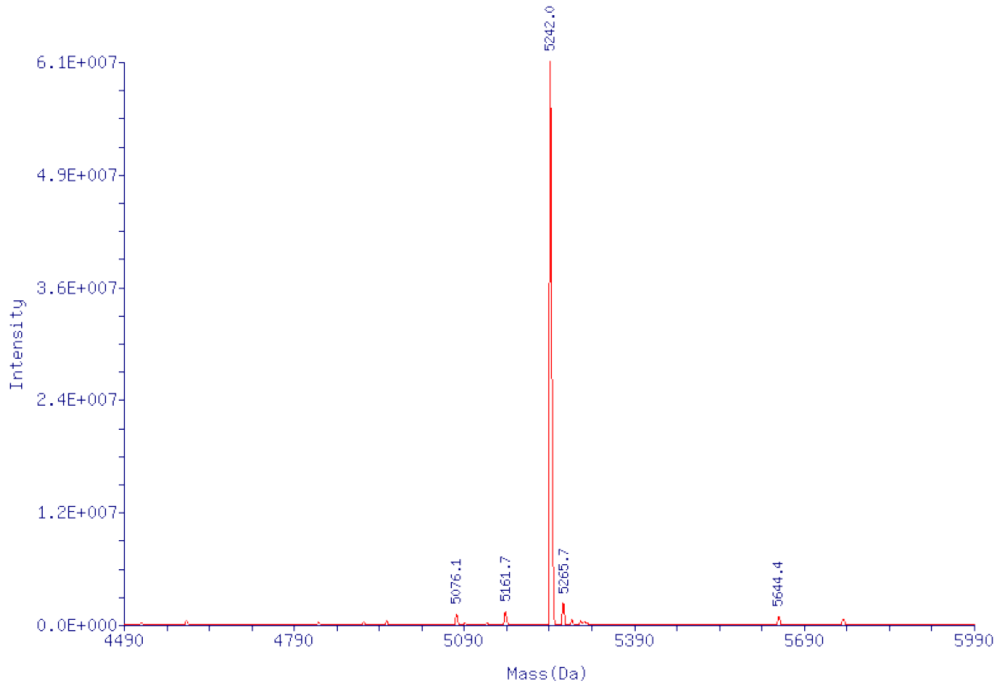

1d-TM

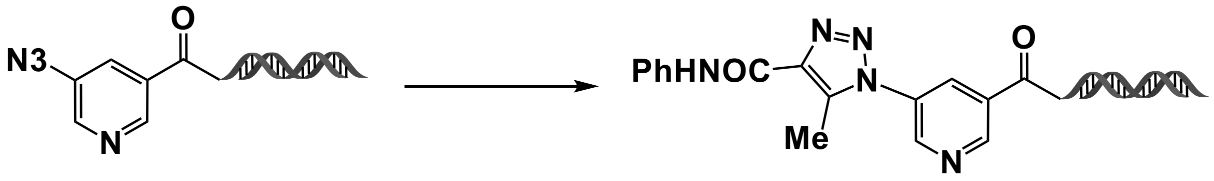

Exact MS:
5241.9486

Conversion (Product%) = 100%
Base Peak Mass (Da): 5242.0

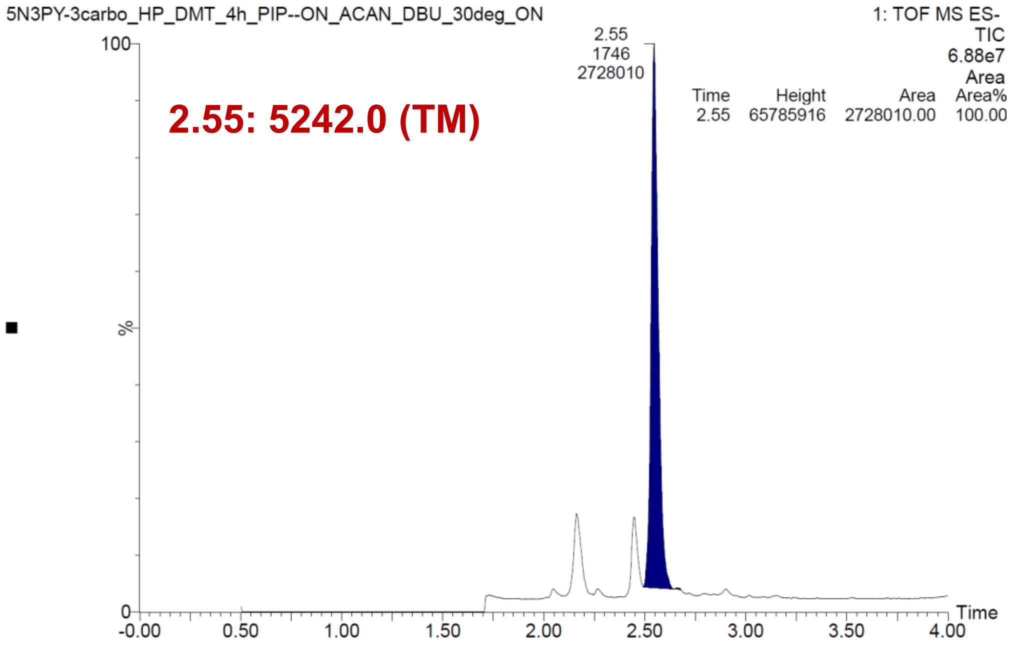

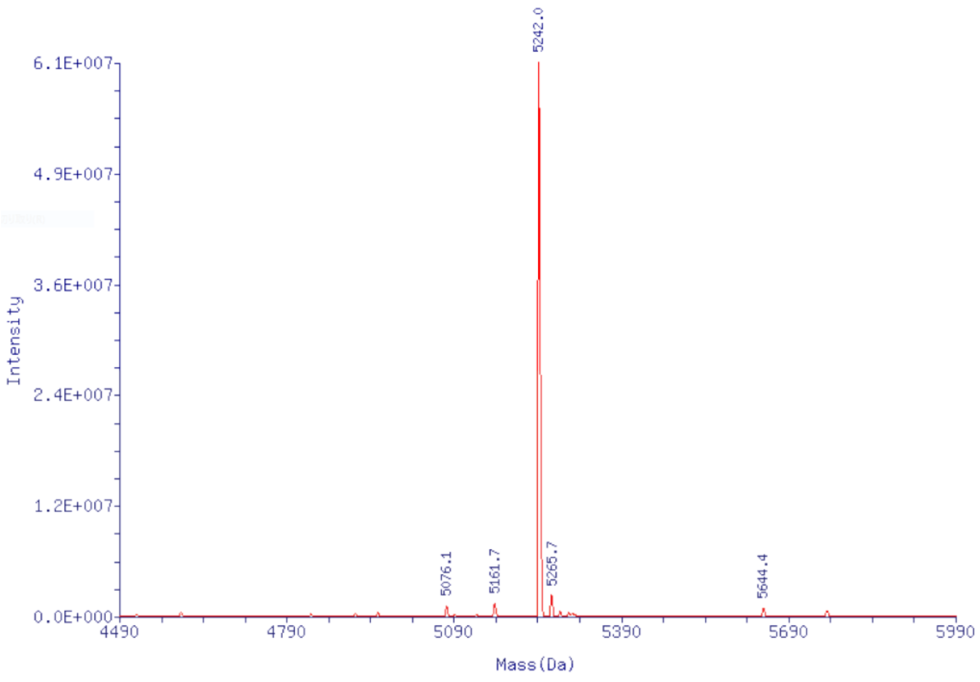

1e-TM

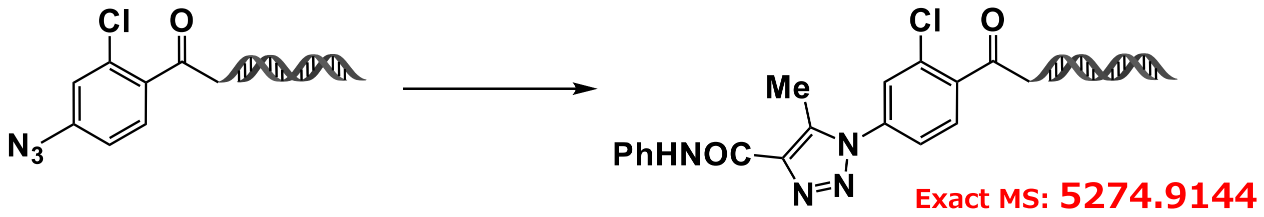

Conversion (Product%) = 96%
Base Peak Mass (Da): 5275.3

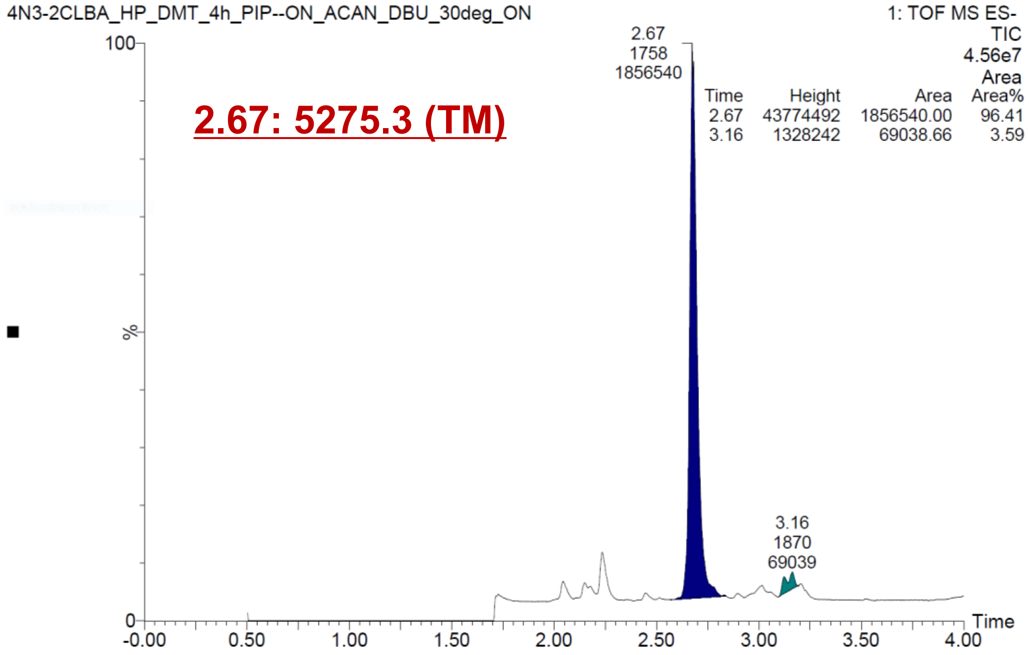

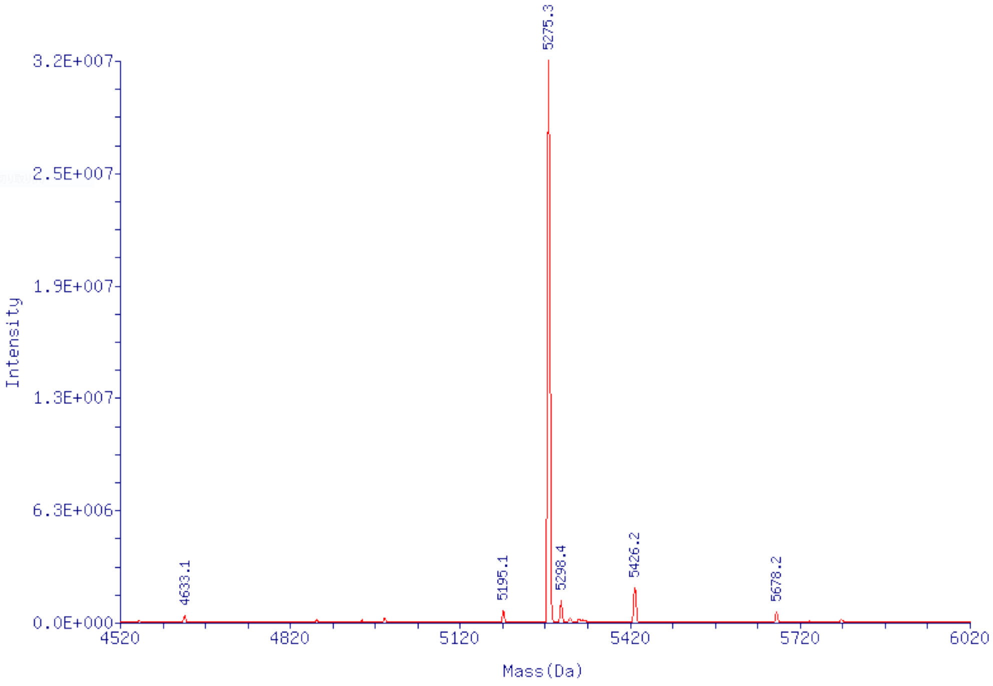

1f-TM

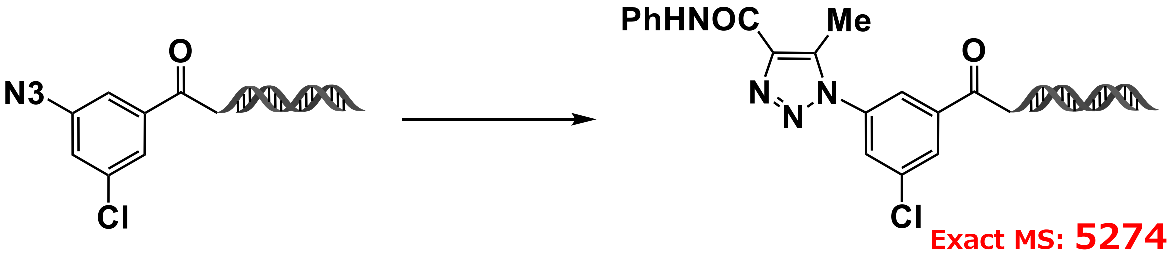

Exact MS: 5274.9144

Conversion (Product%) = 100%
Base Peak Mass (Da): 5275.3

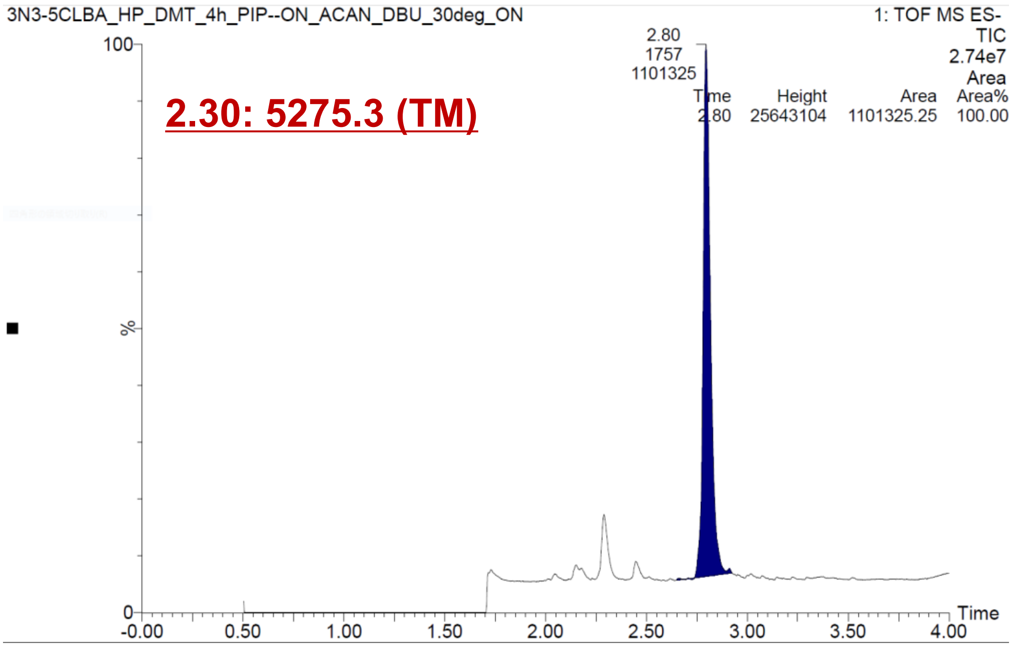

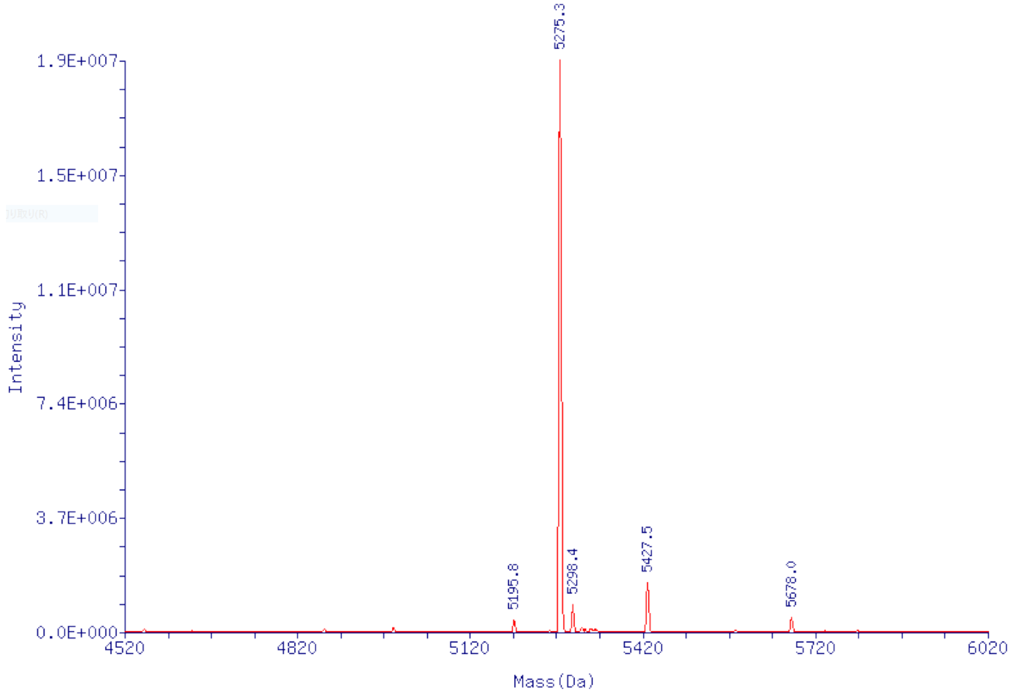

1g-TM

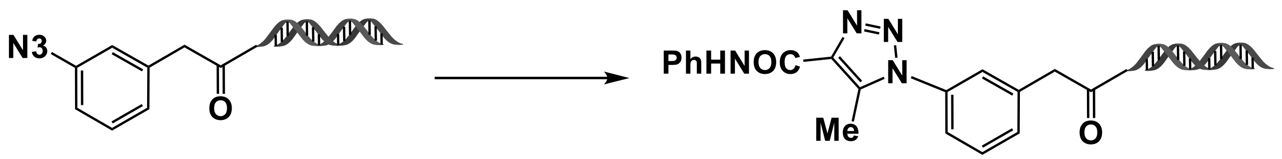

MS: 5254.969

Conversion (Product%) = 51%
Base Peak Mass (Da): 5255.0

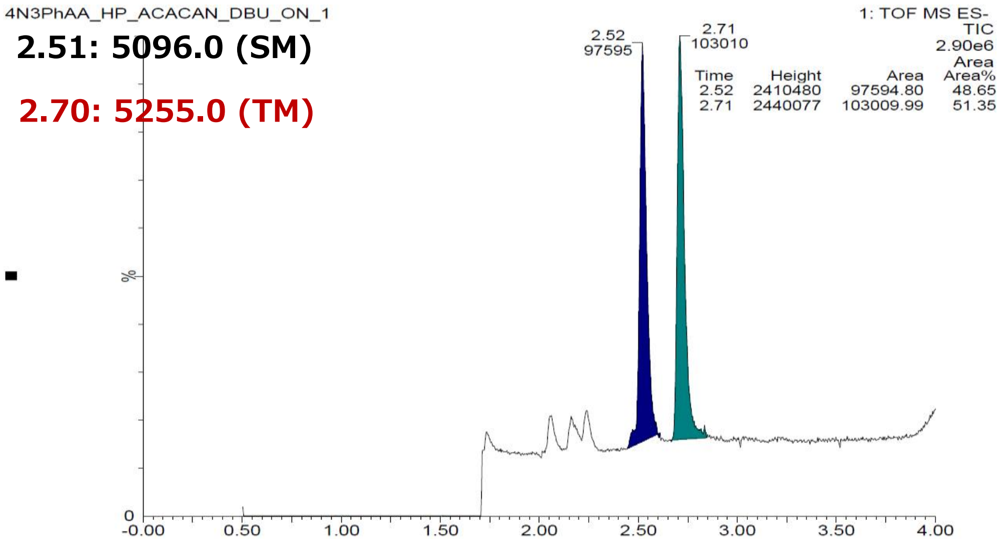

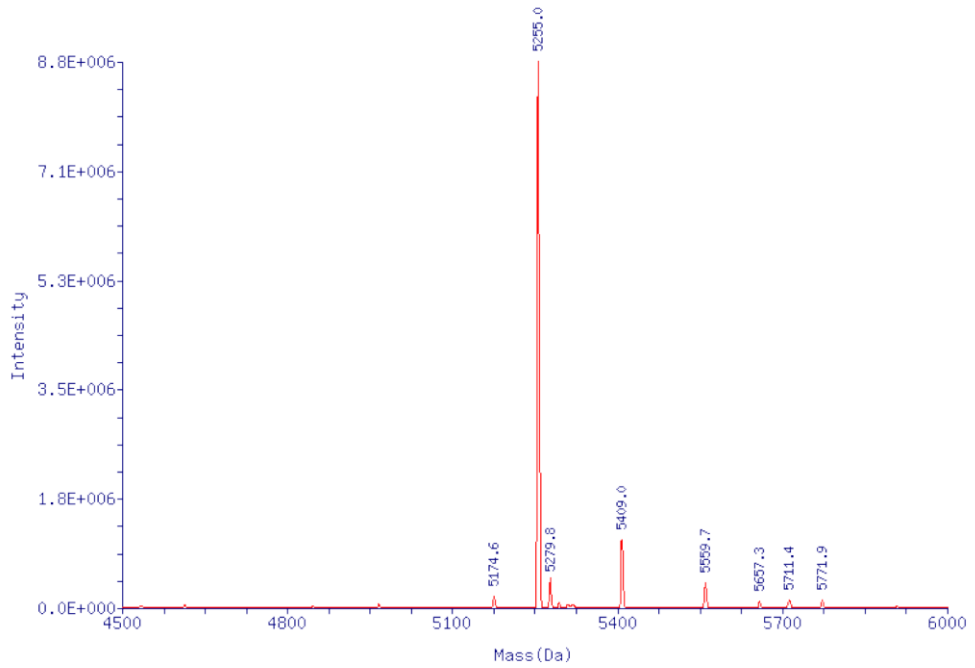

1h-TM

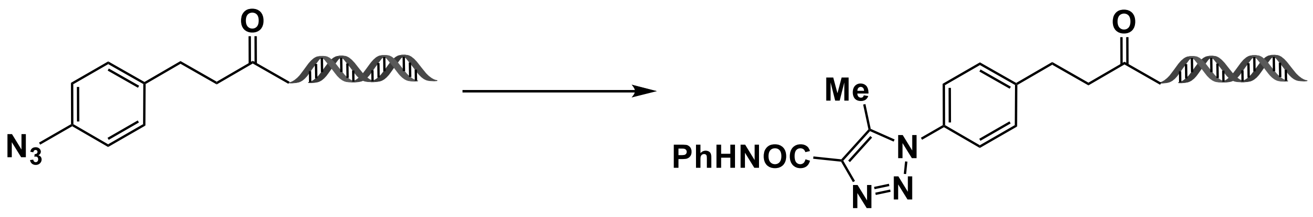

Exact MS: 5268.9847

Conversion (Product%) = 51%
Base Peak Mass (Da): 5269.0

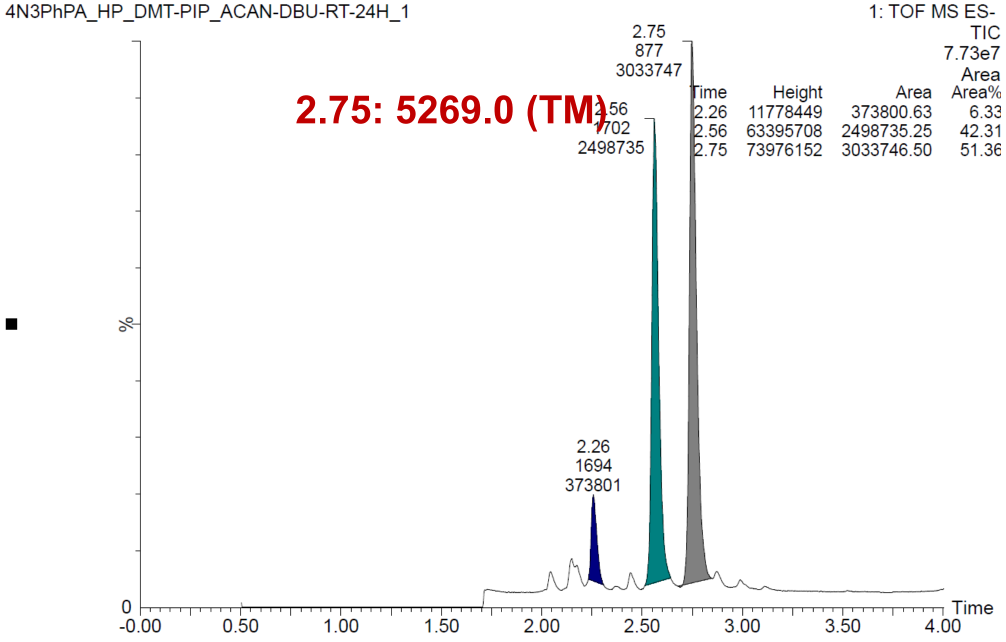

Time	Height	Area	Area%
2.26	11778449	373800.63	6.33
2.56	63395708	2498735.25	42.31
2.75	73976152	3033746.50	51.36

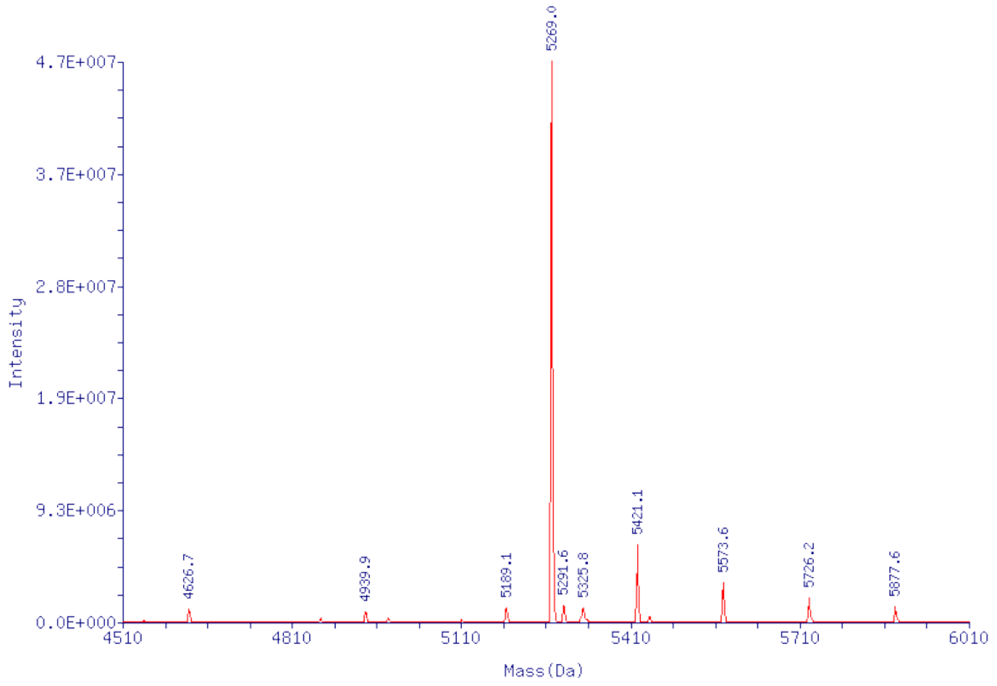

1i-TM

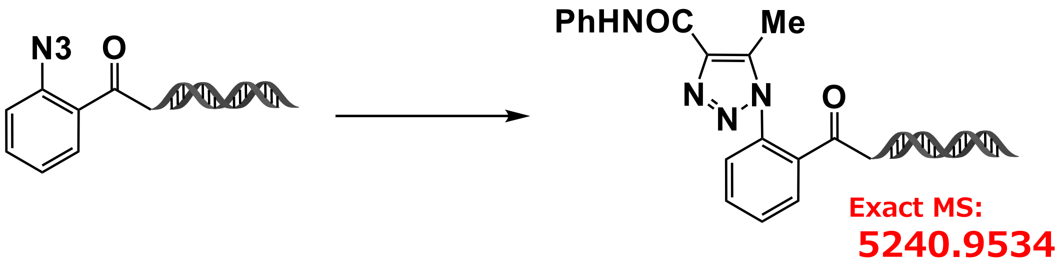

Conversion (Product%) = 31%
Base Peak Mass (Da): 5240.9

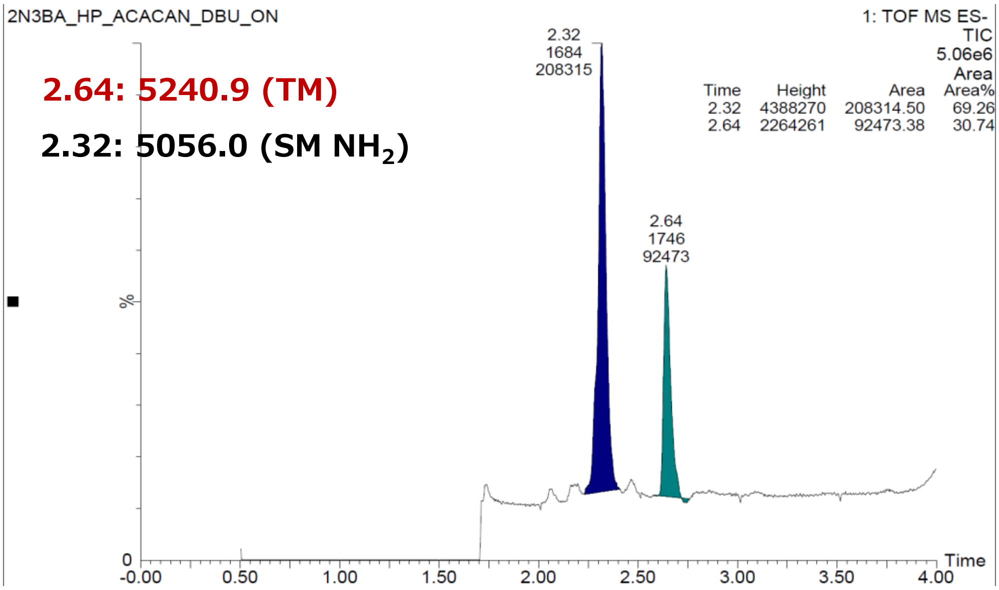

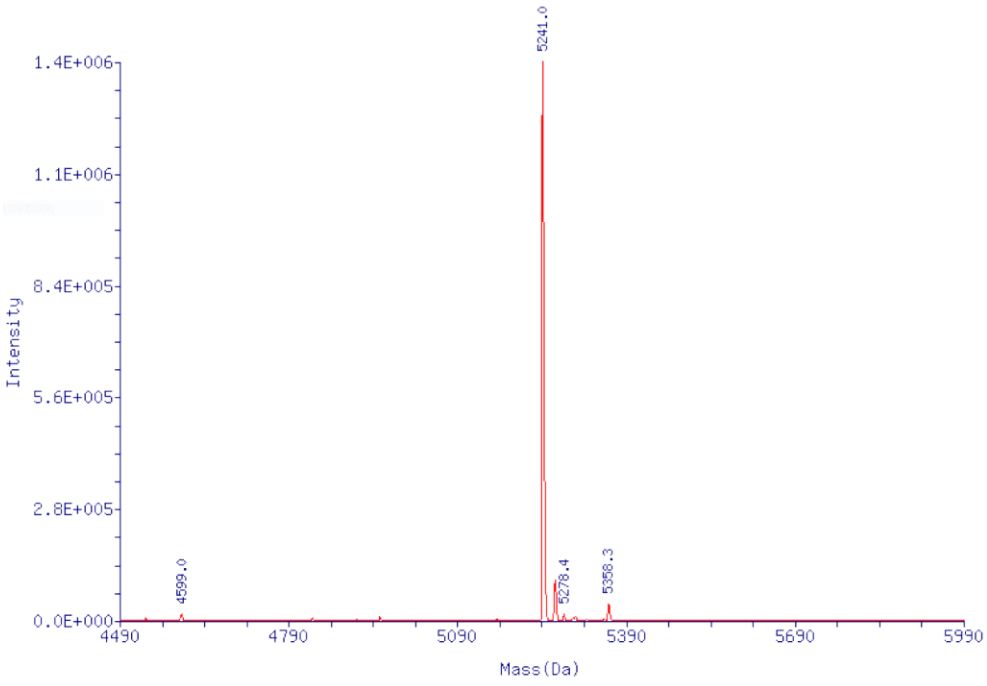

1j-TM

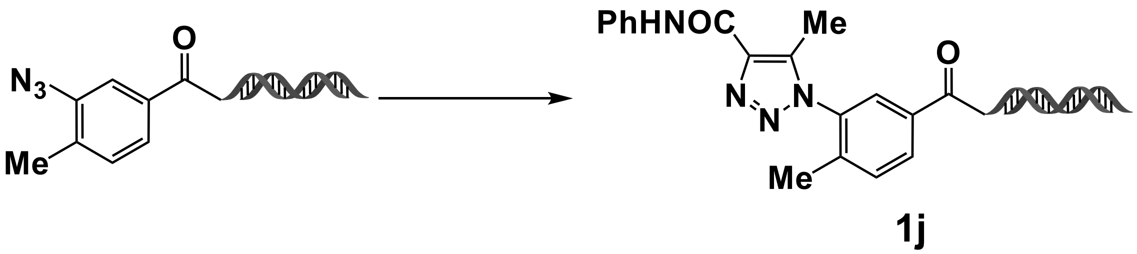

Conversion (Product%) = 54%
Base Peak Mass (Da): 5255.0

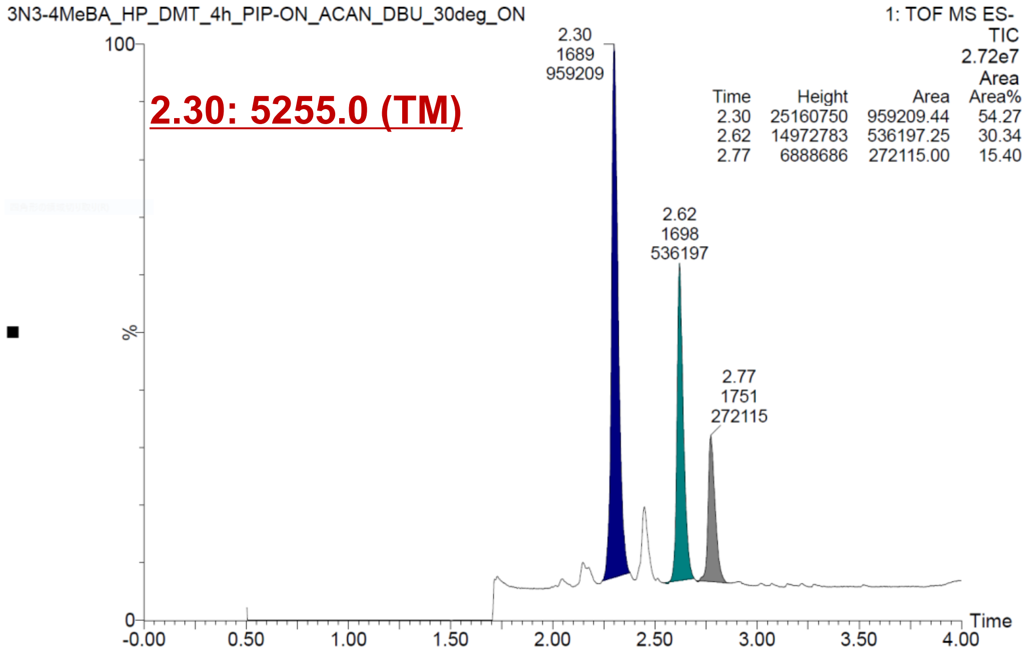

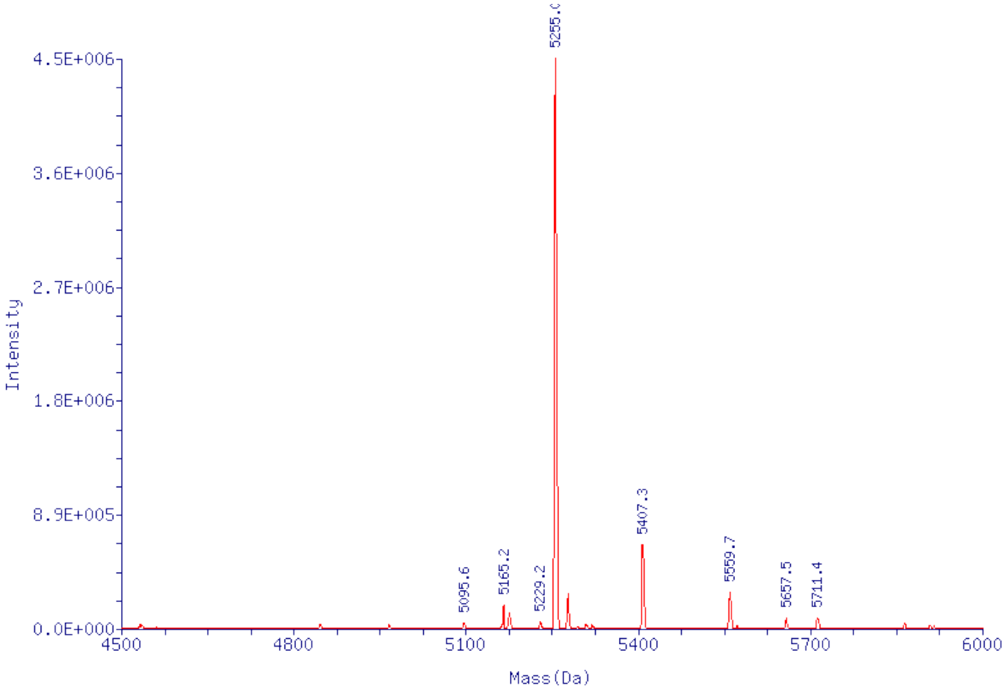

1k-TM

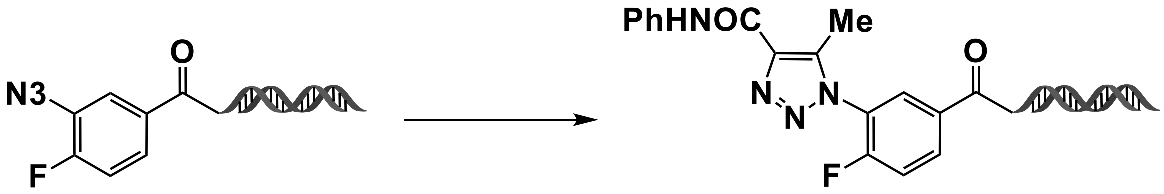

Exact MS: 5258.944

Conversion (Product%) = 50%
Base Peak Mass (Da): 5259.2

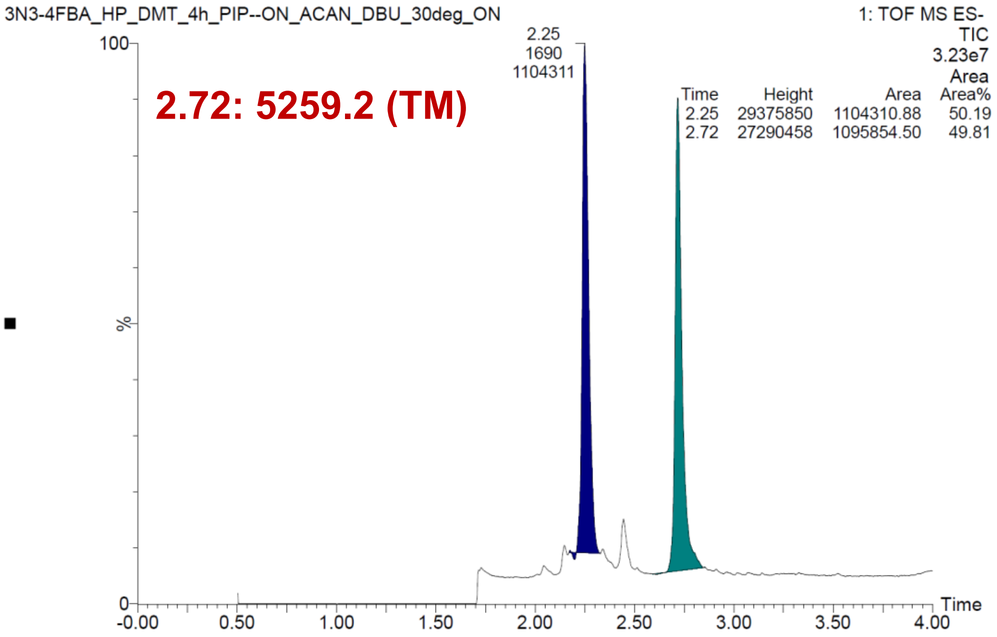

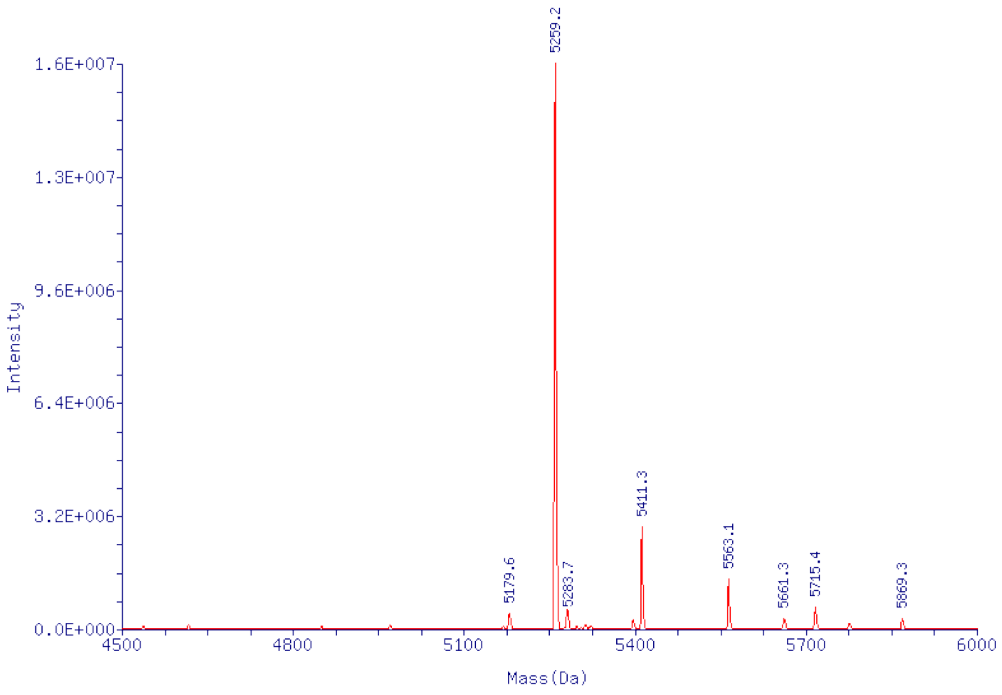

1l-TM

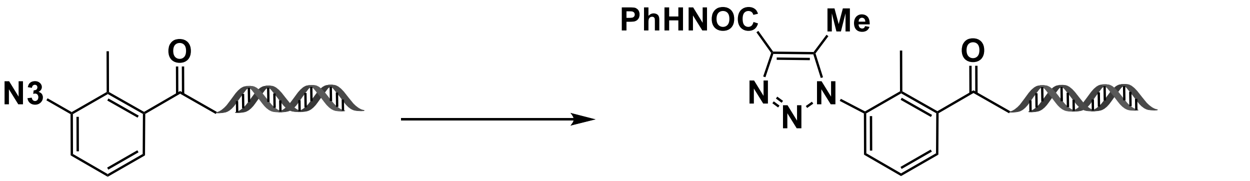

Exact MS: **5258.944**

Conversion (Product%) = 19%
Base Peak Mass (Da): 5255.0

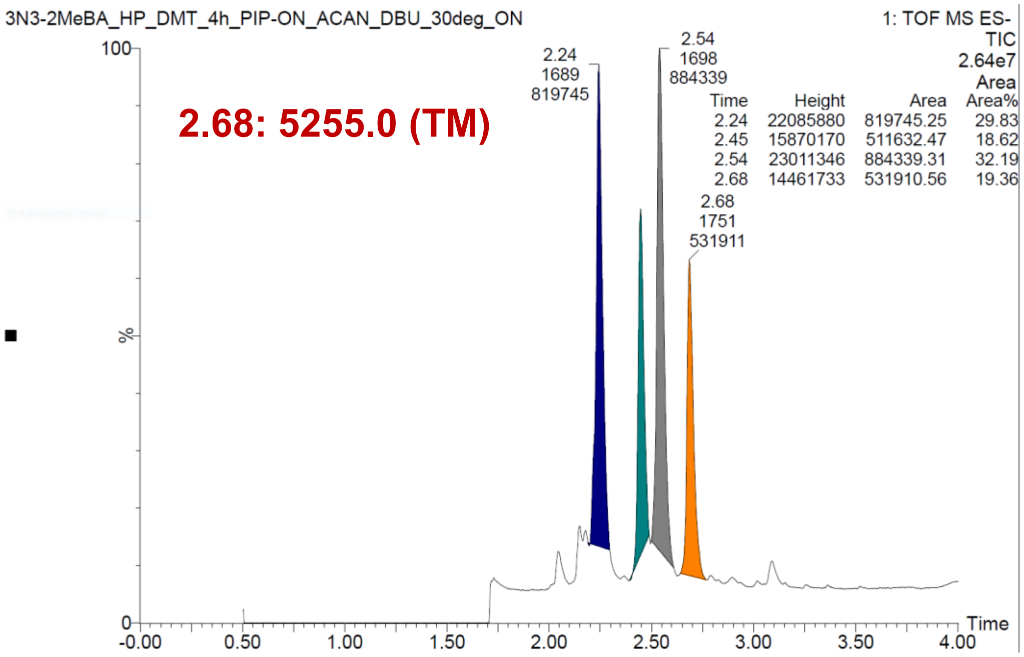

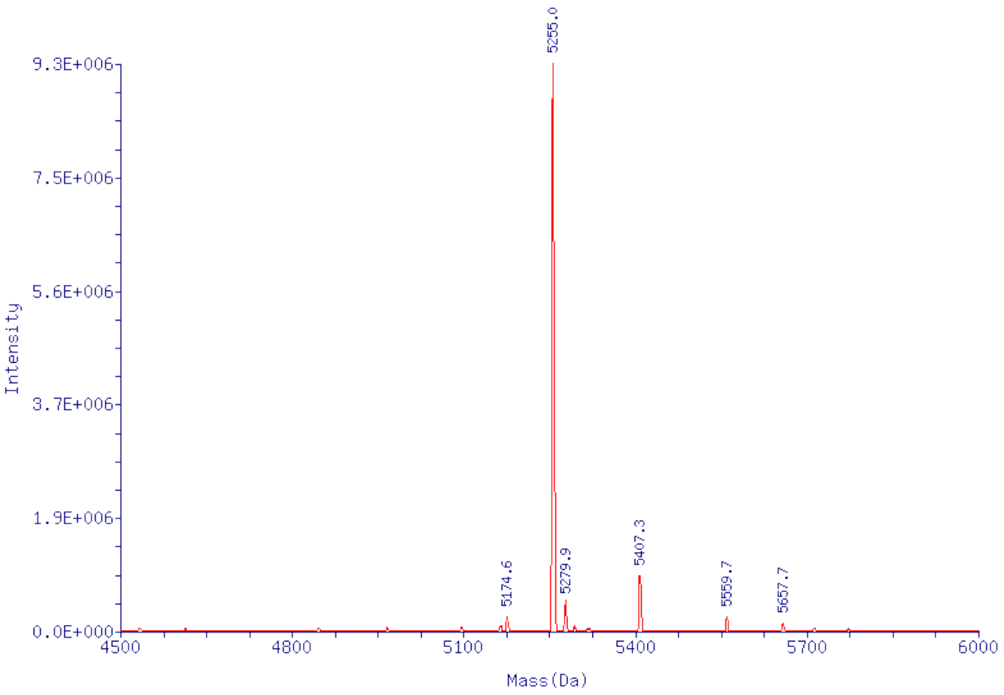

1m-reaction

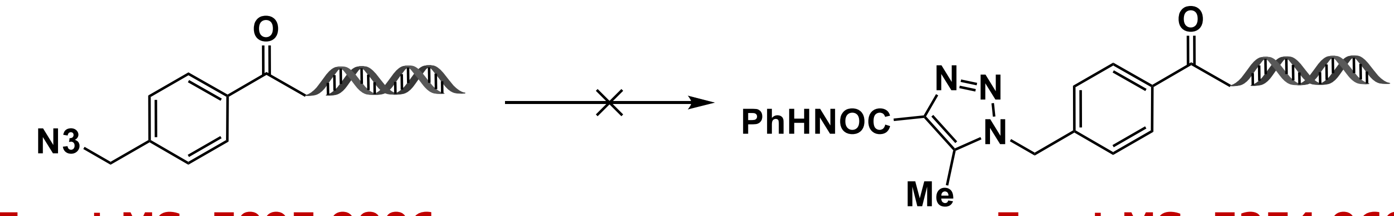

Exact MS: 5095.9006

Exact MS: 5254.969

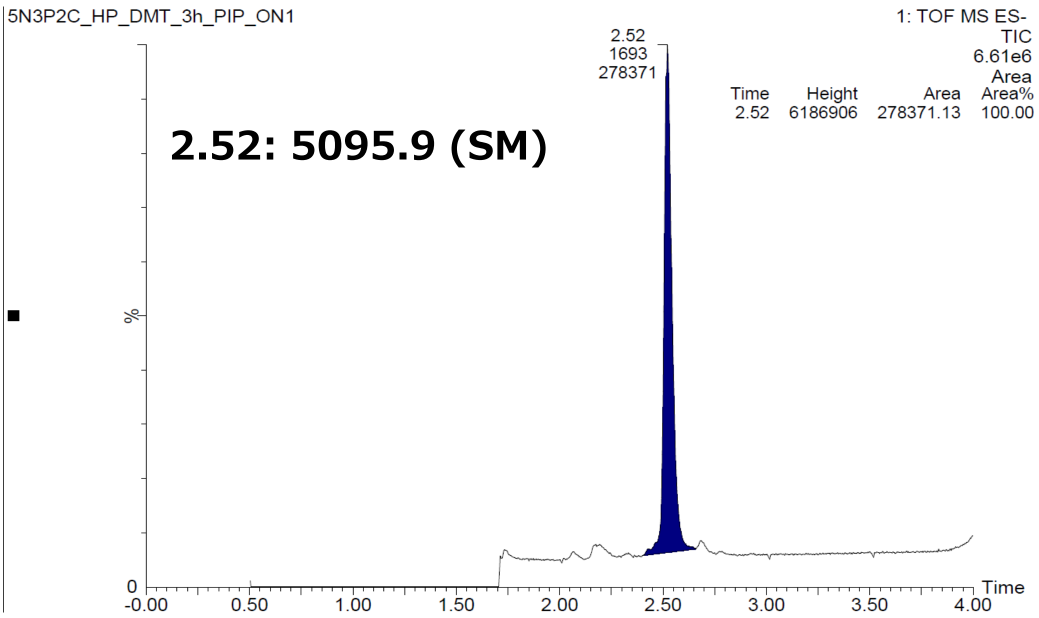

1n-reaction

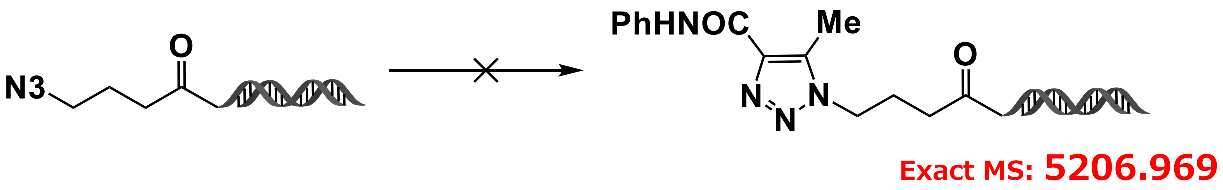
N3
O
PhHNOC
Me
N
N
N
O
Exact MS: 5206.969

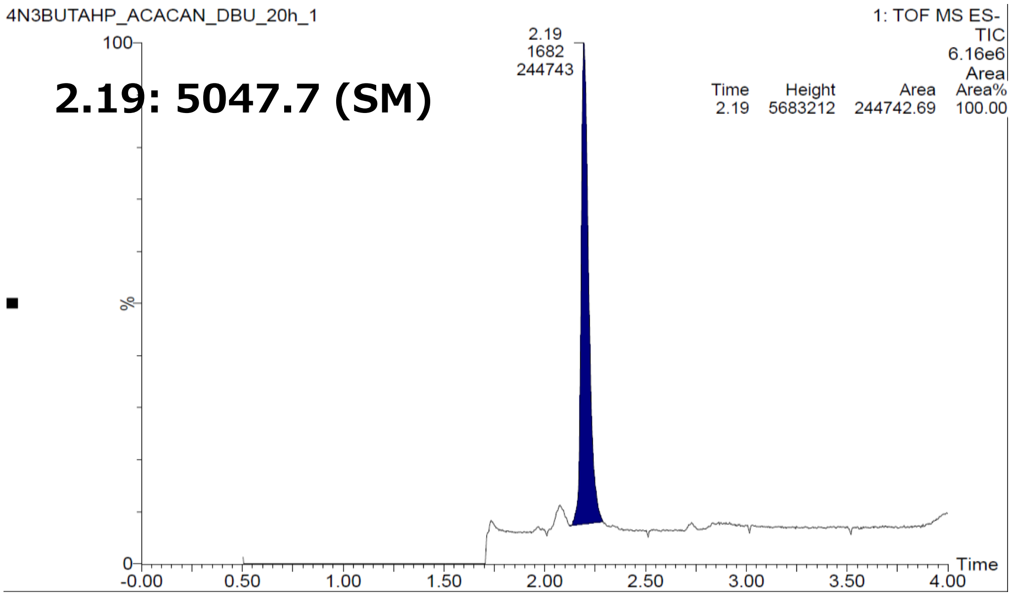
4N3BUTAHP_ACACAN_DBU_20h_1
1: TOF MS ES-
TIC
6.16e6
2.19: 5047.7 (SM)
2.19
1682
244743
Time 2.19
Height 5683212
Area 244742.69
Area Area% 100.00
100
%
0
-0.00
0.50
1.00
1.50
2.00
2.50
3.00
3.50
4.00
Time

1o-reaction

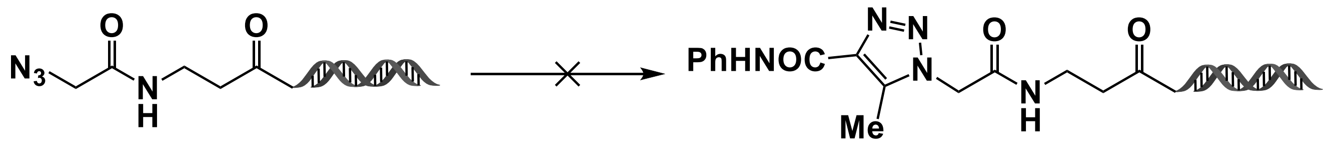

Exact MS: 5249.9749

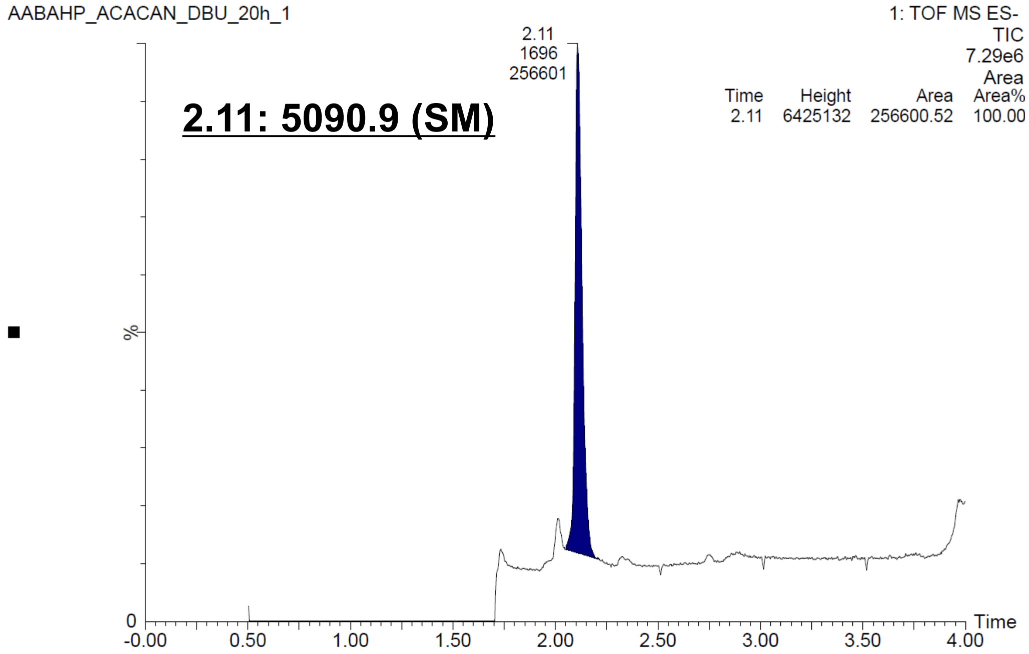

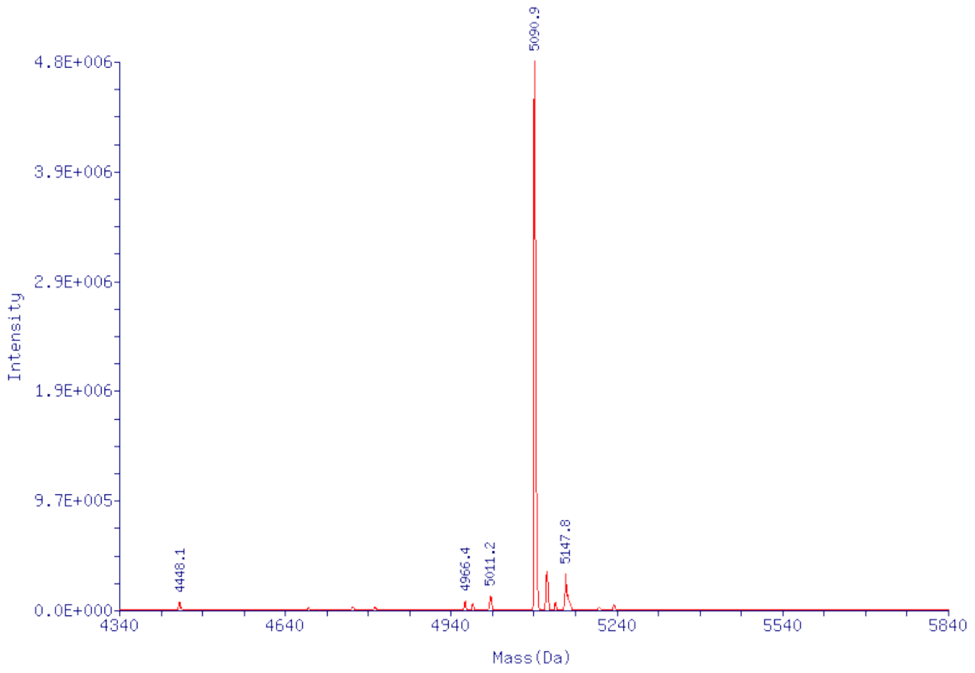

1p-reaction

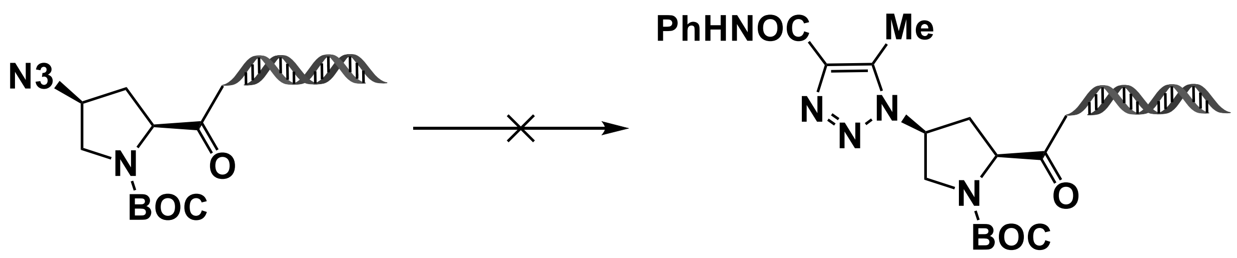

Exact MS: 5174.9640

Exact MS: 5358.0324

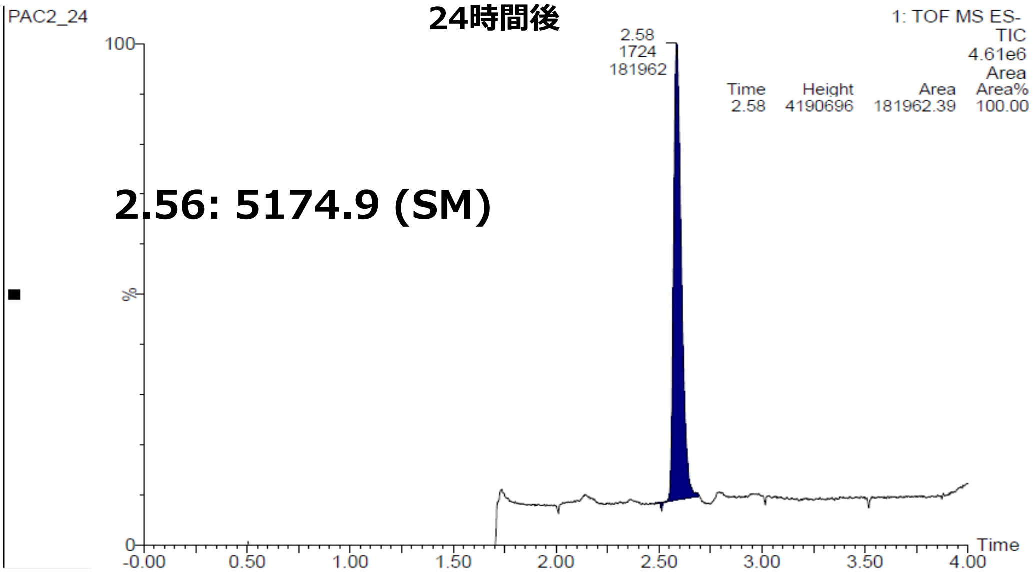

Design of a Novel On-DNA Platform Molecule: N-(4-Azidobenzoyl)-azidohomoalanine-HP (hAla-based DAP)

3

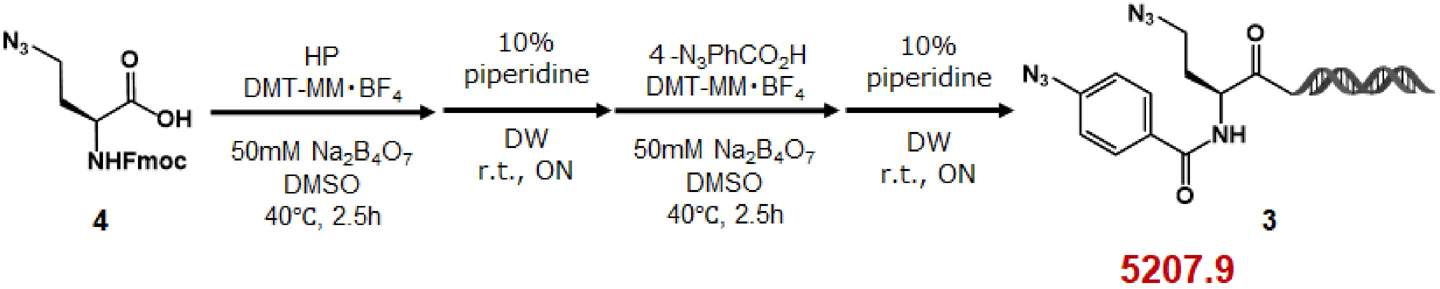

Conversion (Product%) = 91%
Base Peak Mass (Da): 5207.8

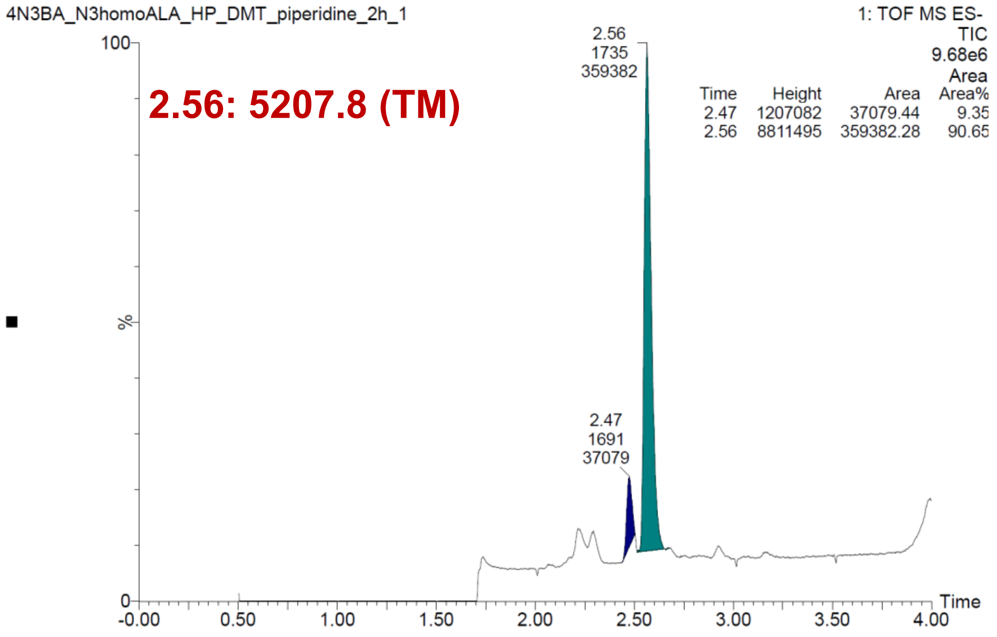

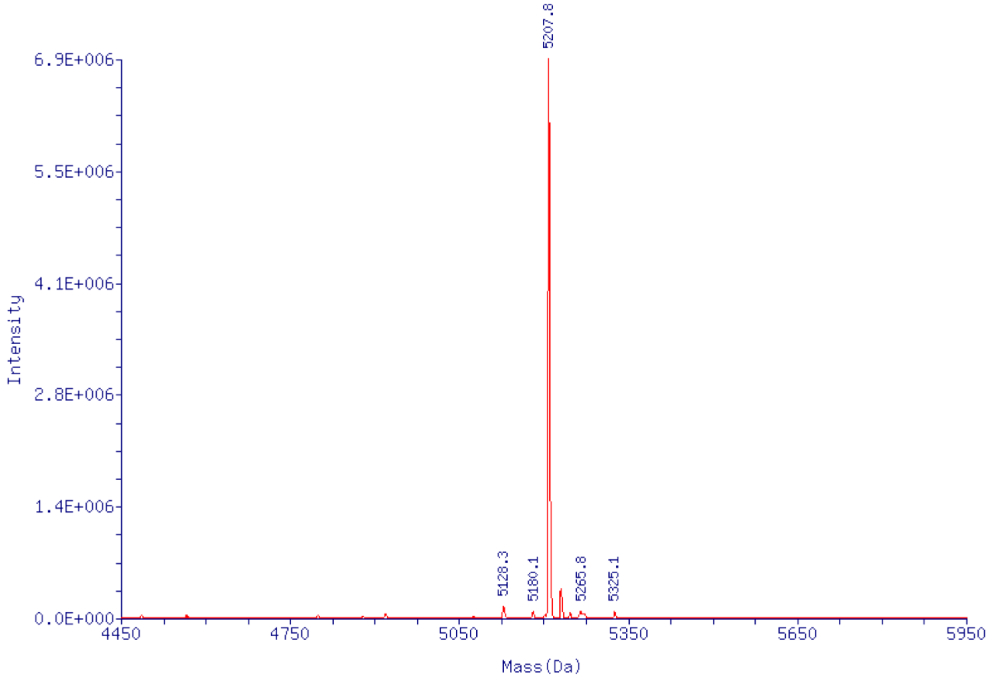

Organocatalyzed [3+2] Cycloaddition Reactions of 4N₃-BA-(*S*)-N₃-homoAla–HP

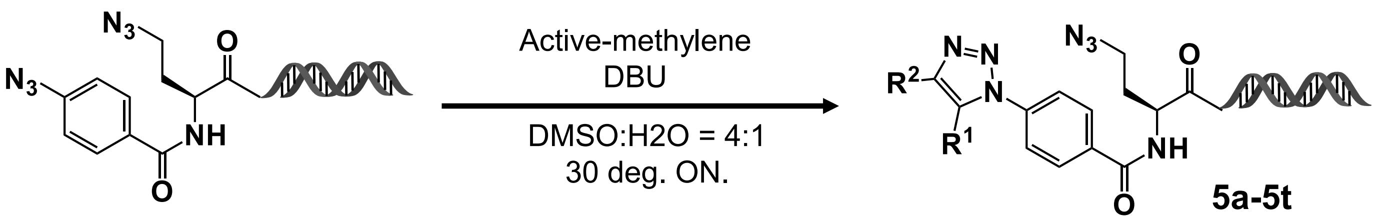

5a-5t

5a-TM

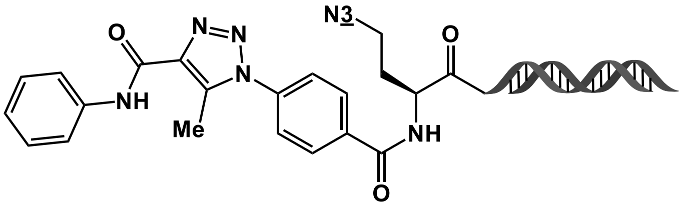

Conversion (Product%) = 92%
Base Peak Mass (Da): 5367.0

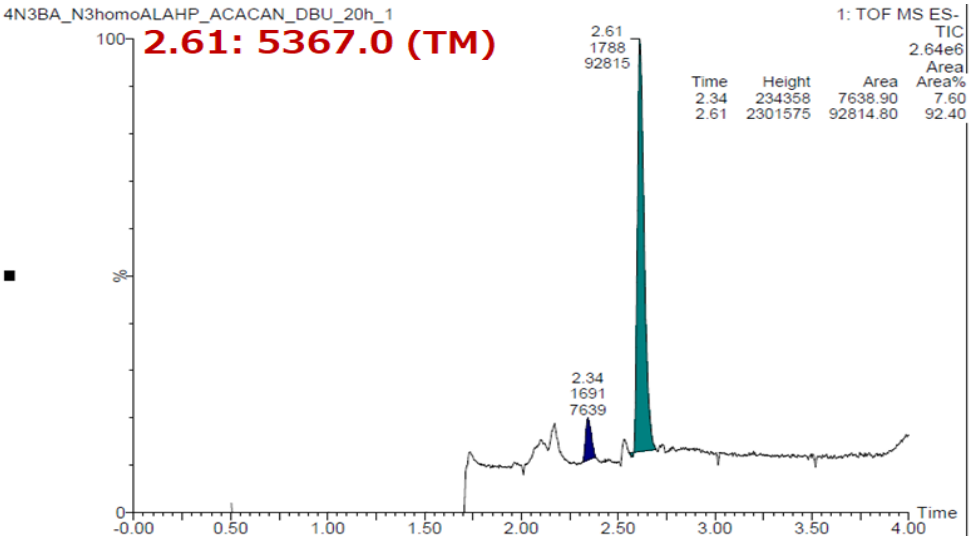

Base Peak Mass (Da)	Intensity	Spectral Quality
5367.0	1.49E+006	ok

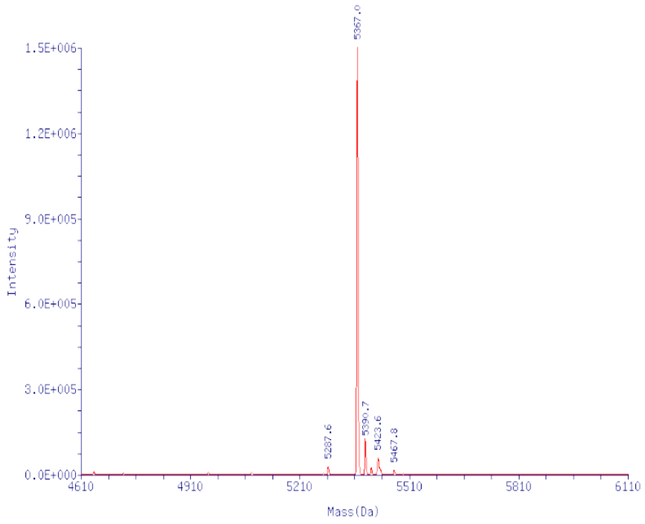

5b-TM

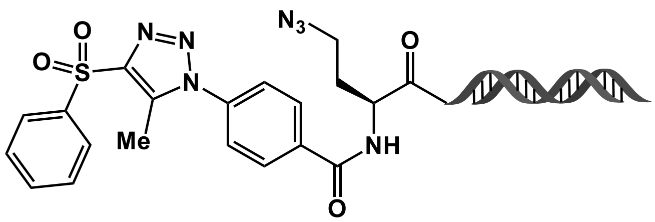

Conversion (Product%) = 92%
Base Peak Mass (Da): 5388.2

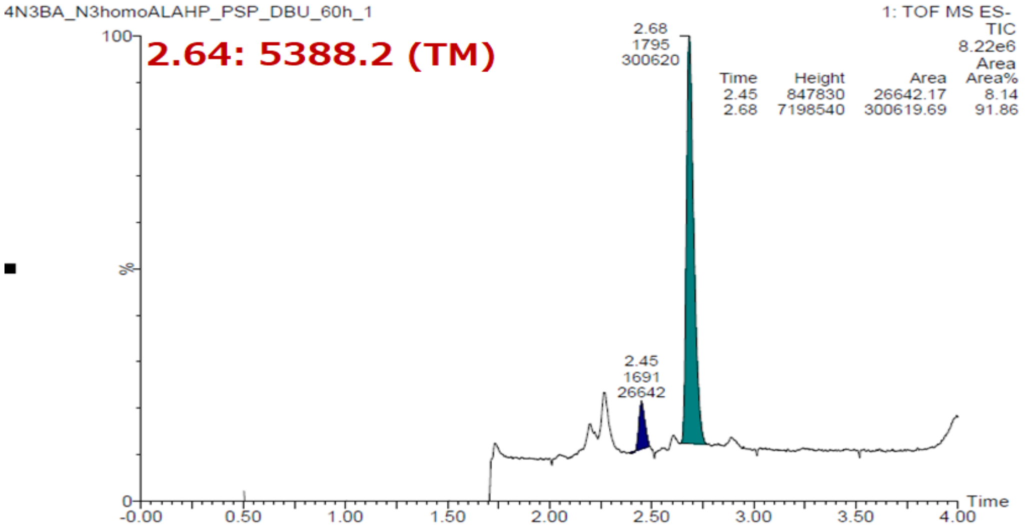

Base Peak Mass (Da)	Intensity	Spectral Quality
5388.2	3.74E+006	ok

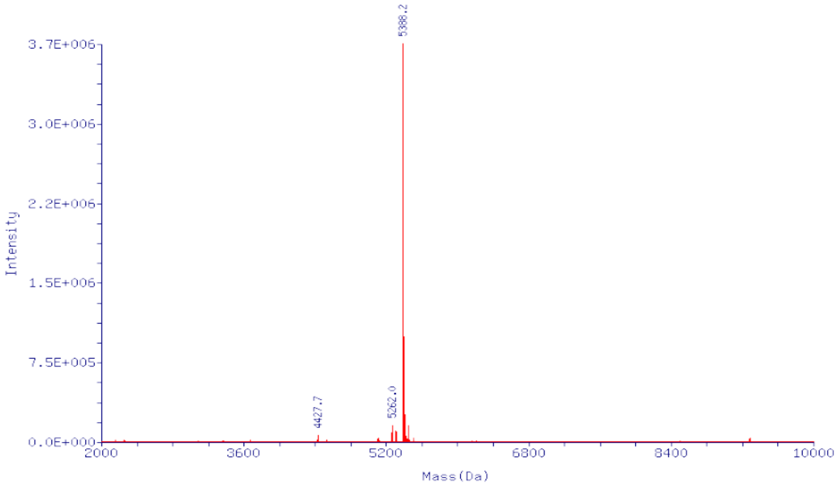

5c-TM

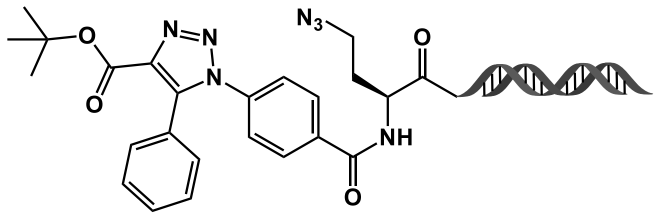

Conversion (Product%) = 94%
Base Peak Mass (Da): 5410.2

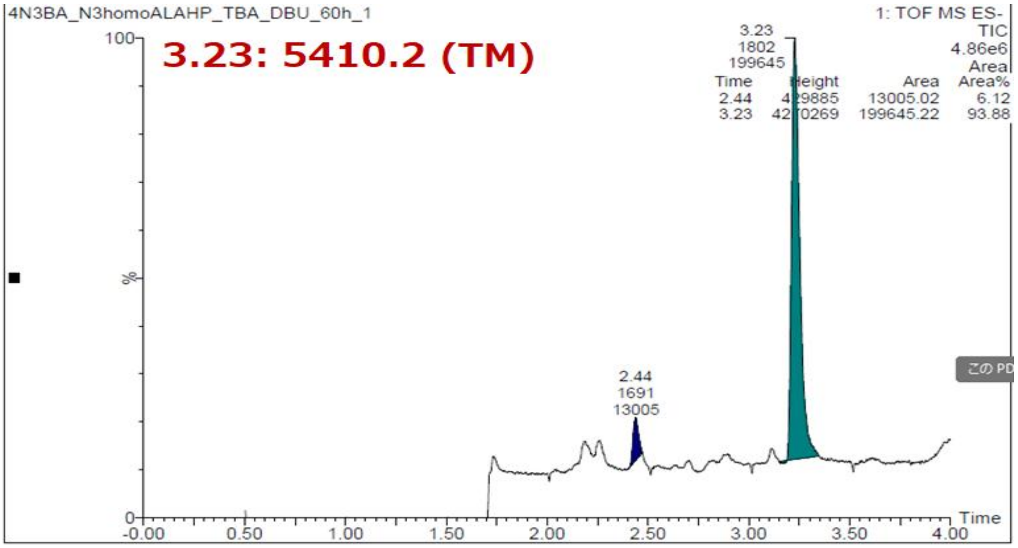

Base Peak Mass (Da)	Intensity	Spectral Quality
5410.2	3.09E+006	ok

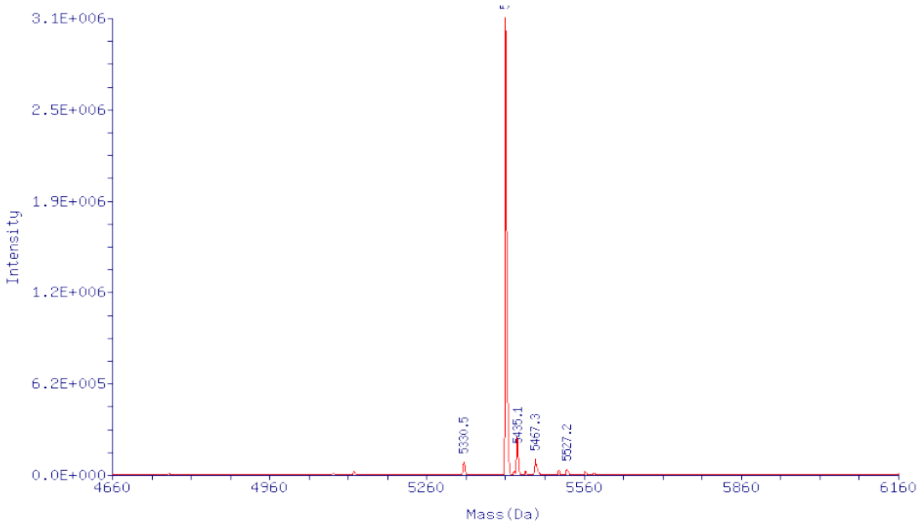

5d-TM

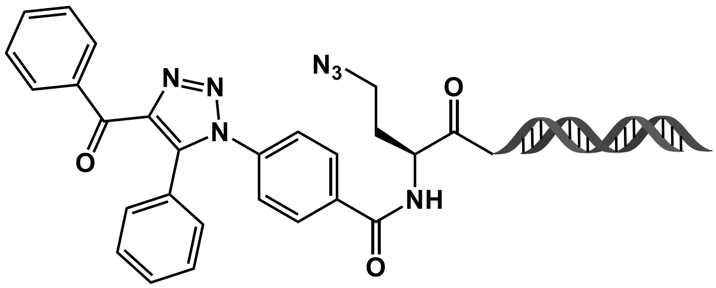

Conversion (Product%) = 90%

Base Peak Mass (Da): 5410.2

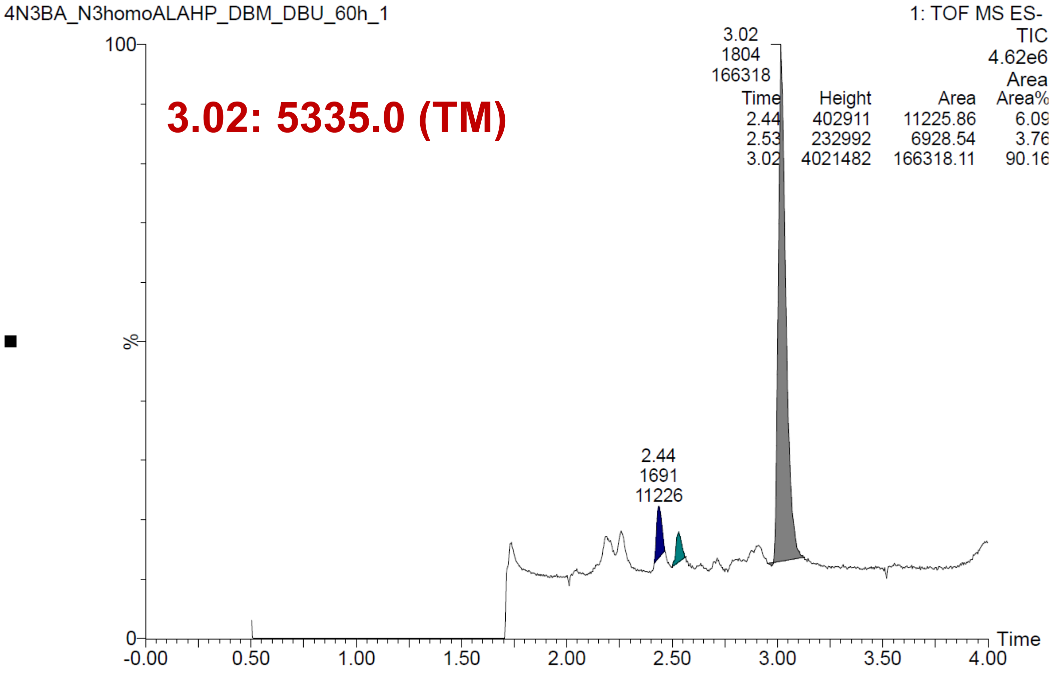

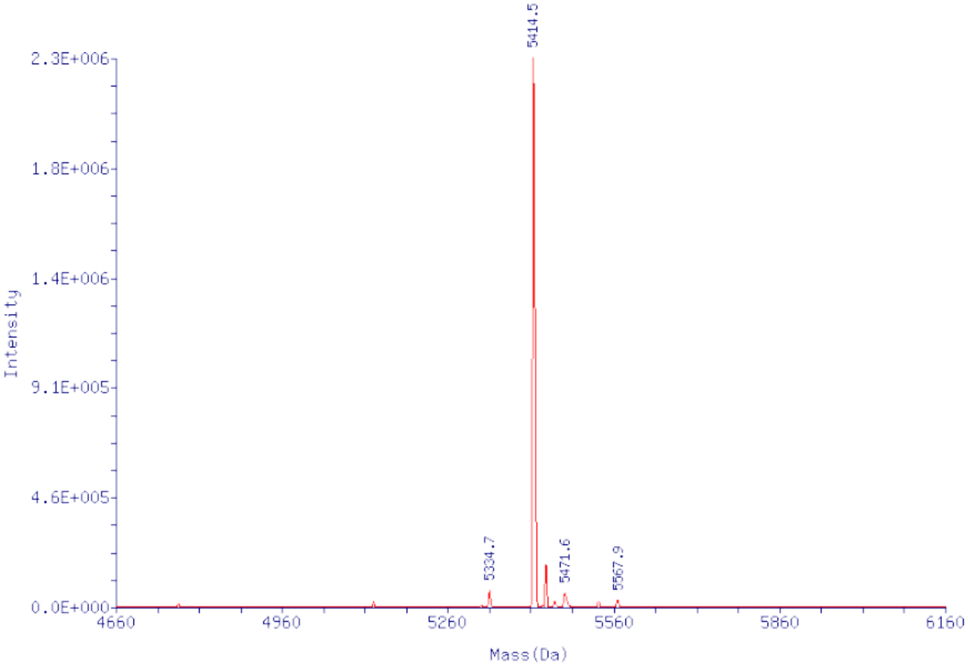

5e-TM

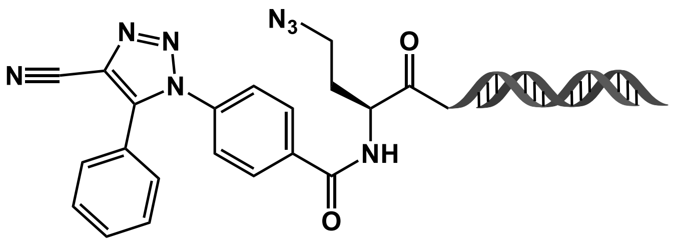

Conversion (Product%) = 92%
Base Peak Mass (Da): 5335.0

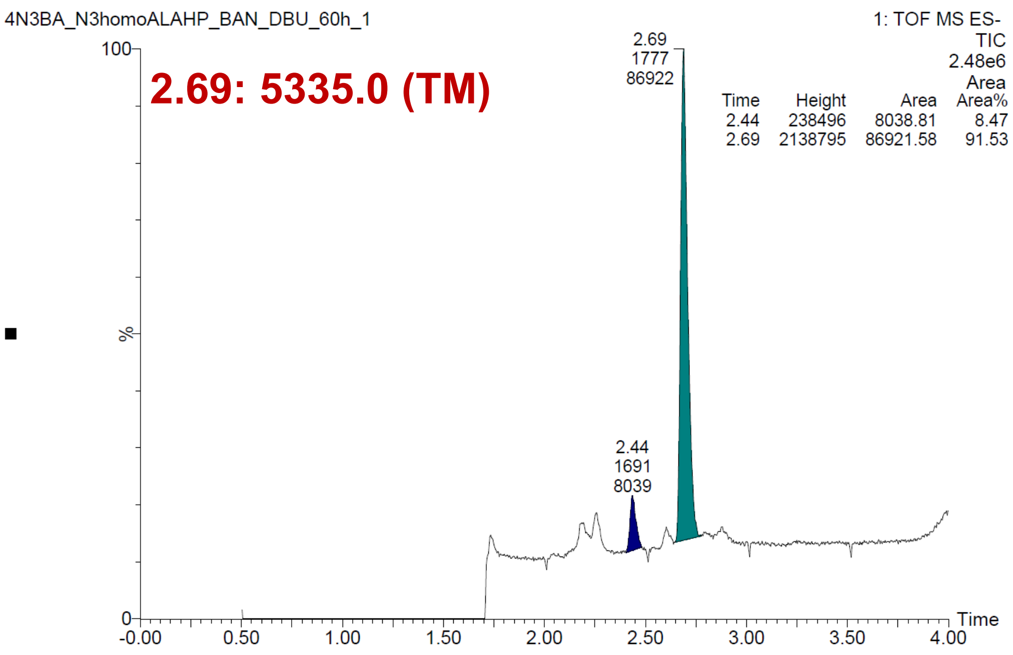

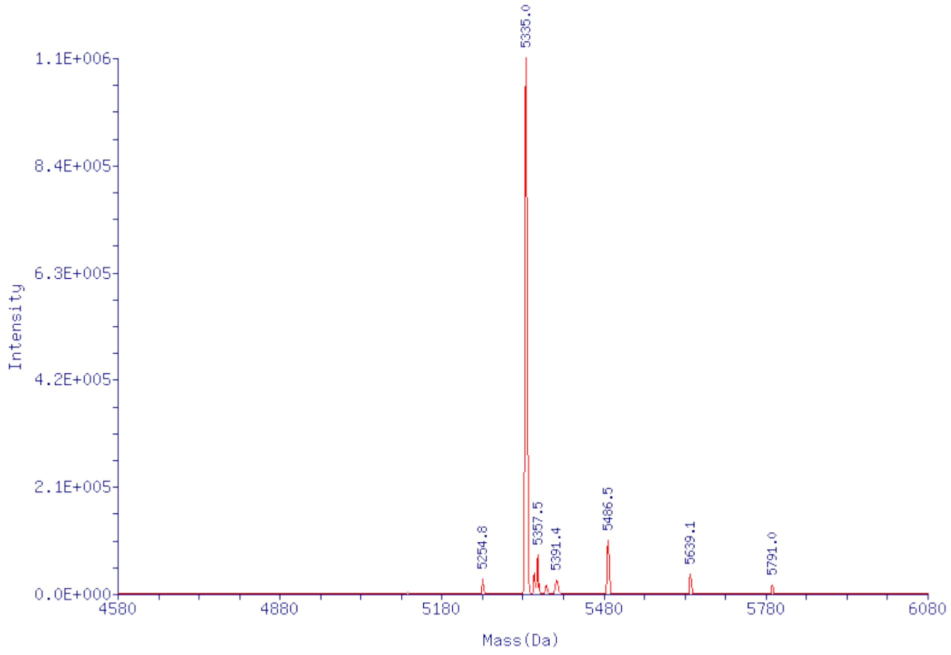

5f-TM

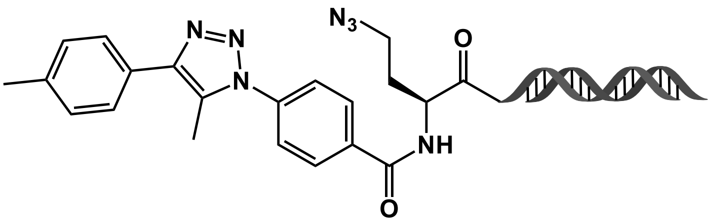

Conversion (Product%) = 88%
Base Peak Mass (Da): 5338.0

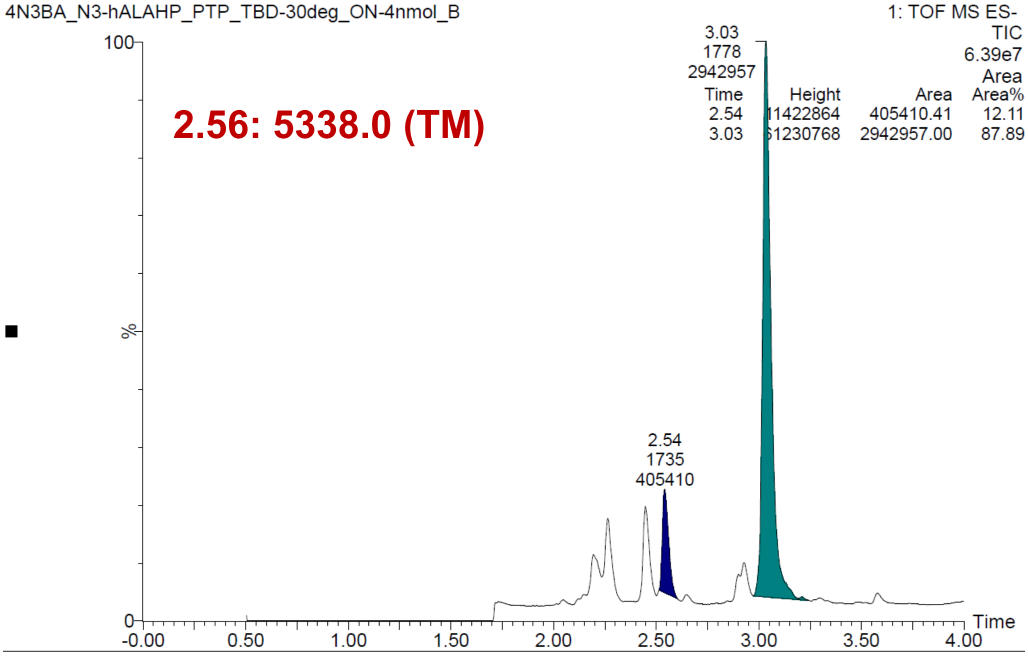

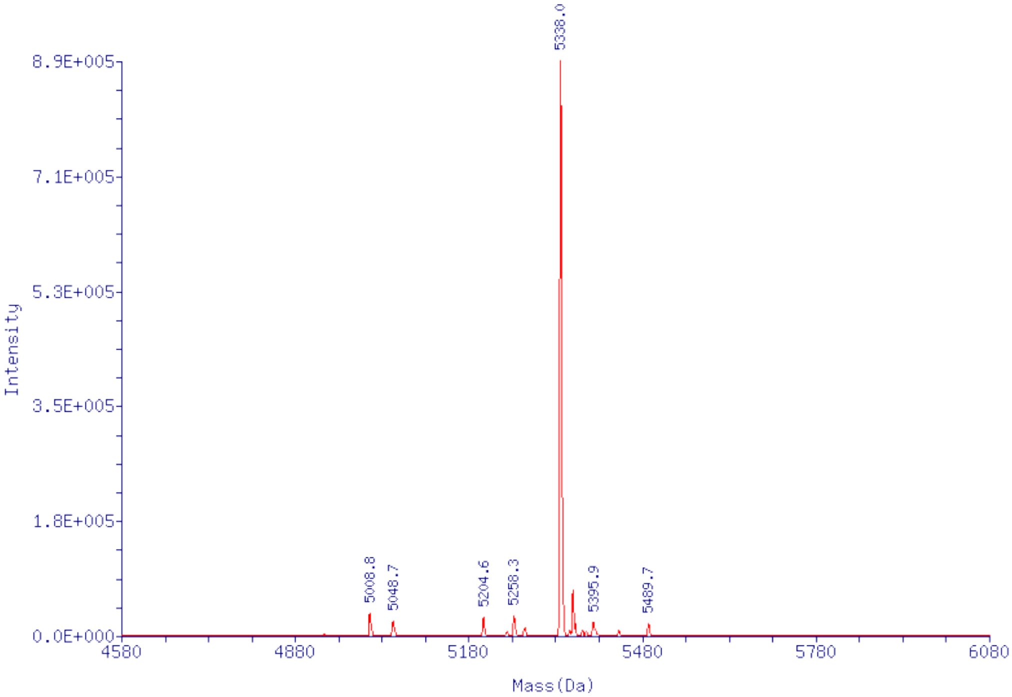

5g-TM

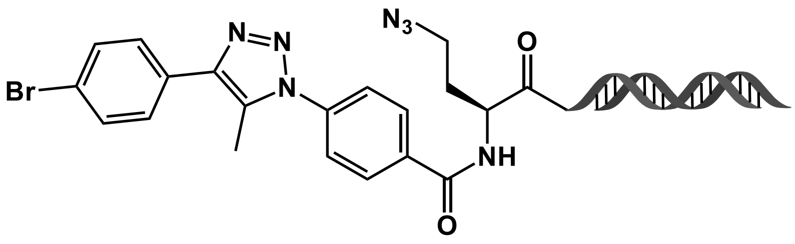

Conversion (Product%) = 86%
Base Peak Mass (Da): 5402.8

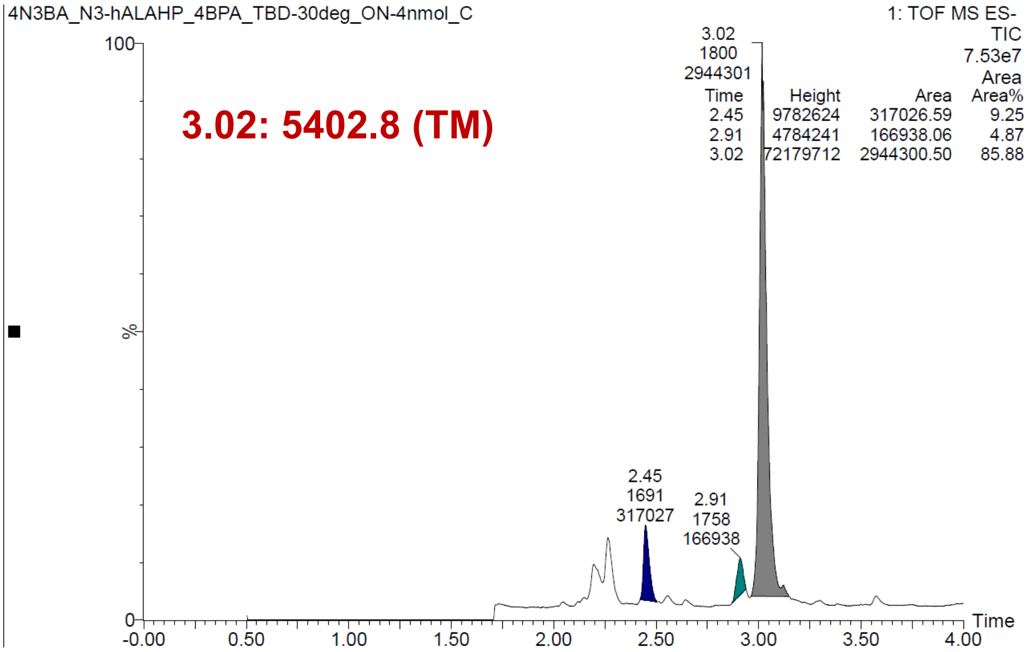

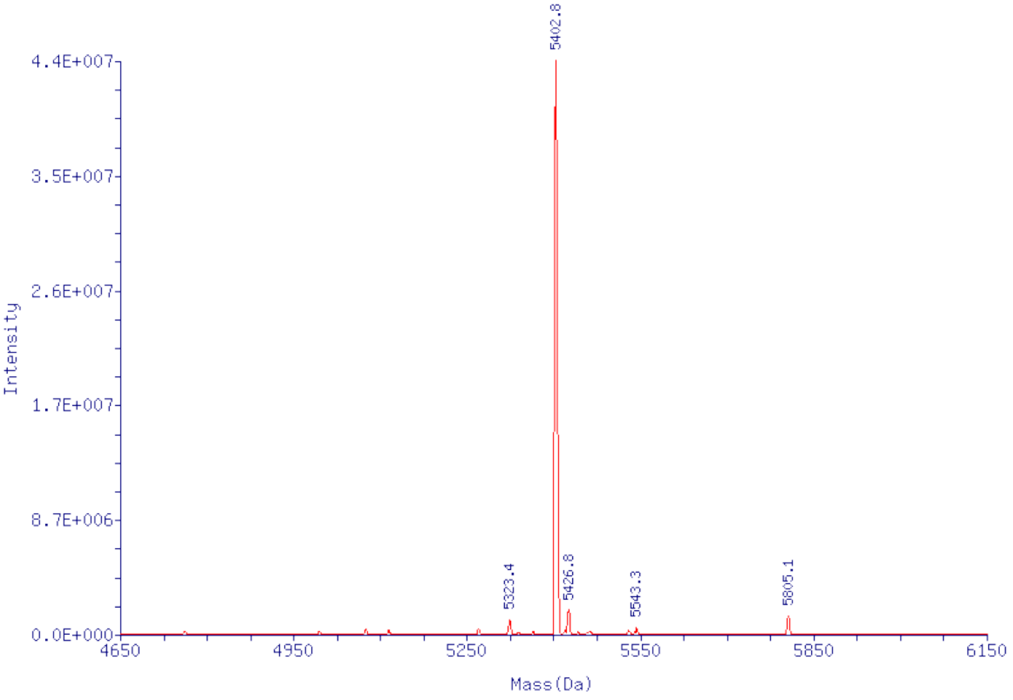

5h-TM

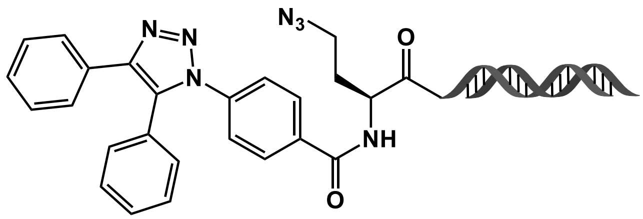

Conversion (Product%) = 89%
Base Peak Mass (Da): 5385.6

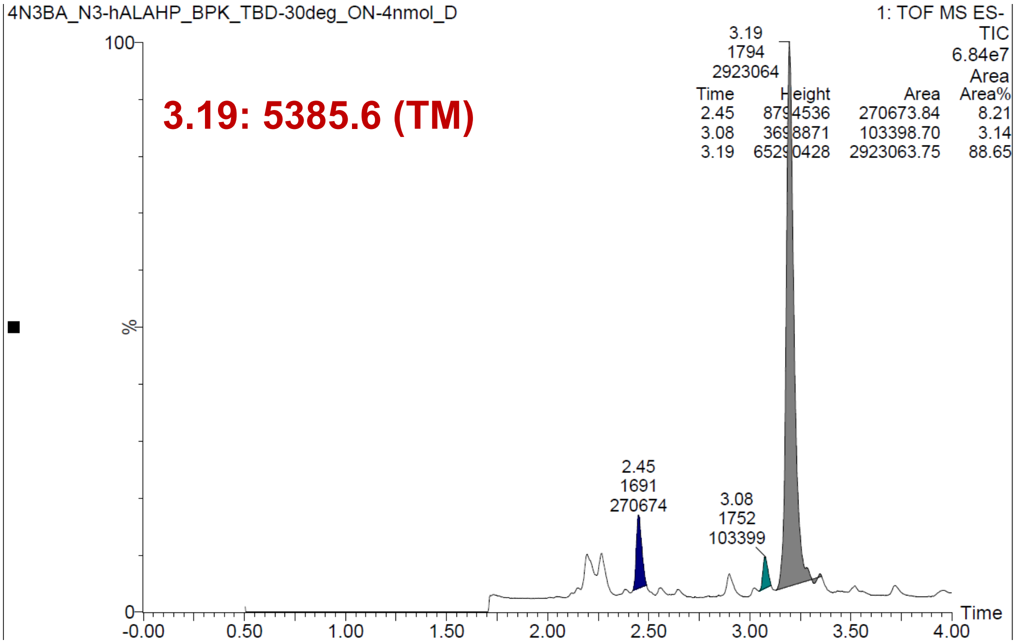

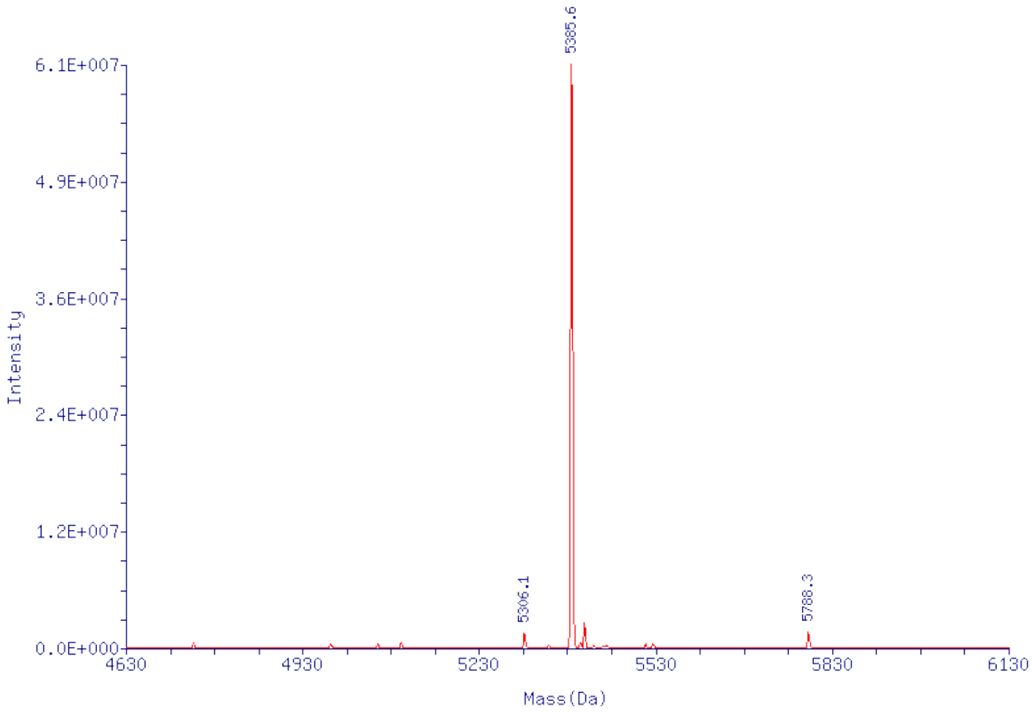

5j

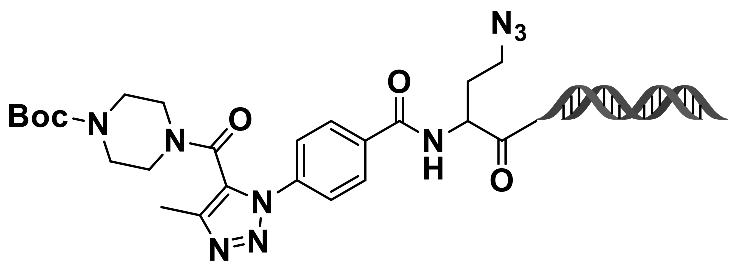

Conversion (Product%) = 97%
Base Peak Mass (Da): 5459.8

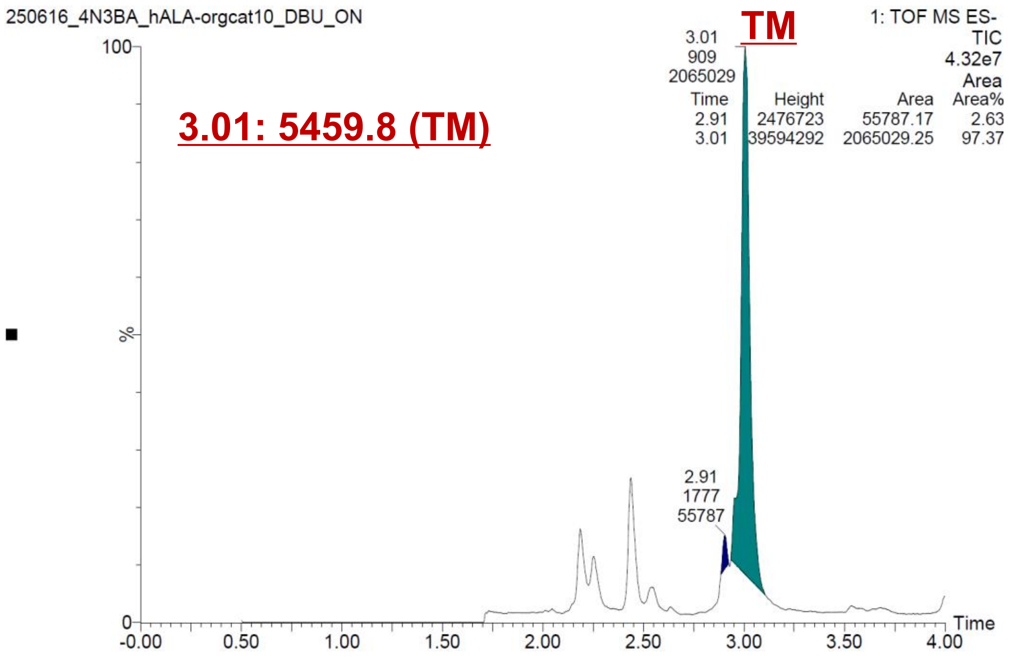

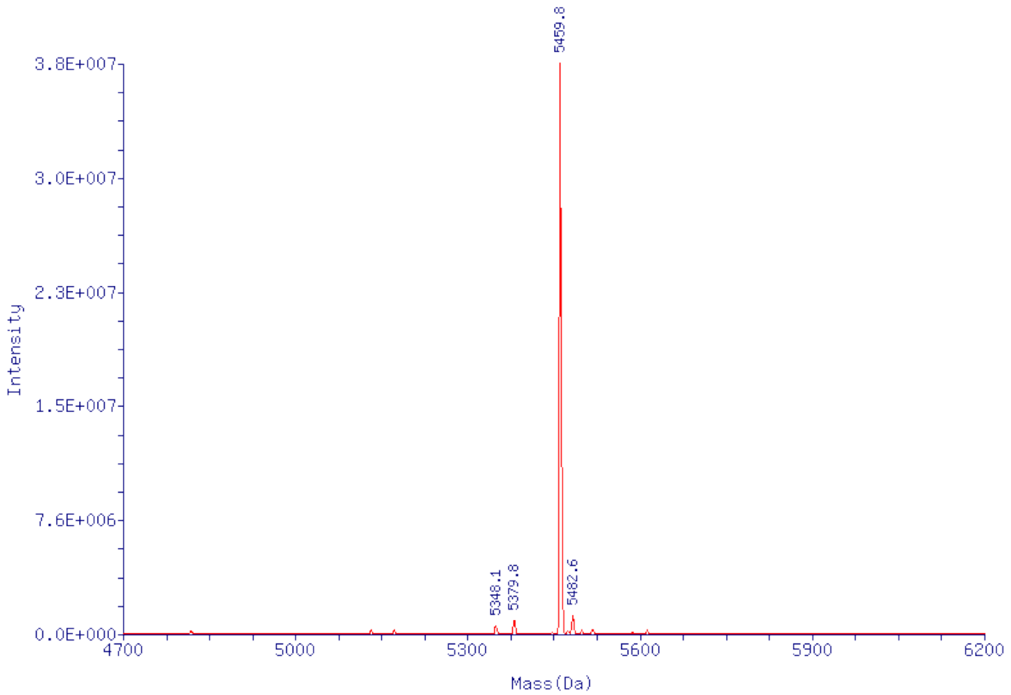

5k

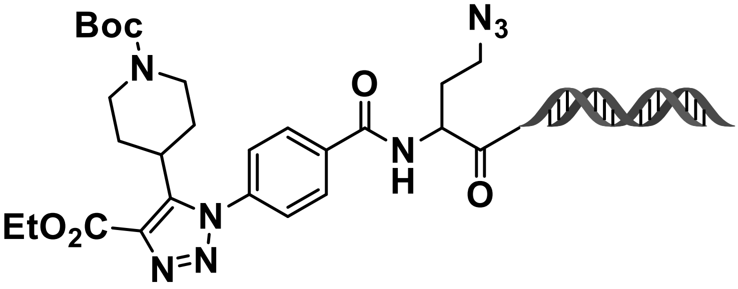

Conversion (Product%) = 93%
Base Peak Mass (Da): 5489.3

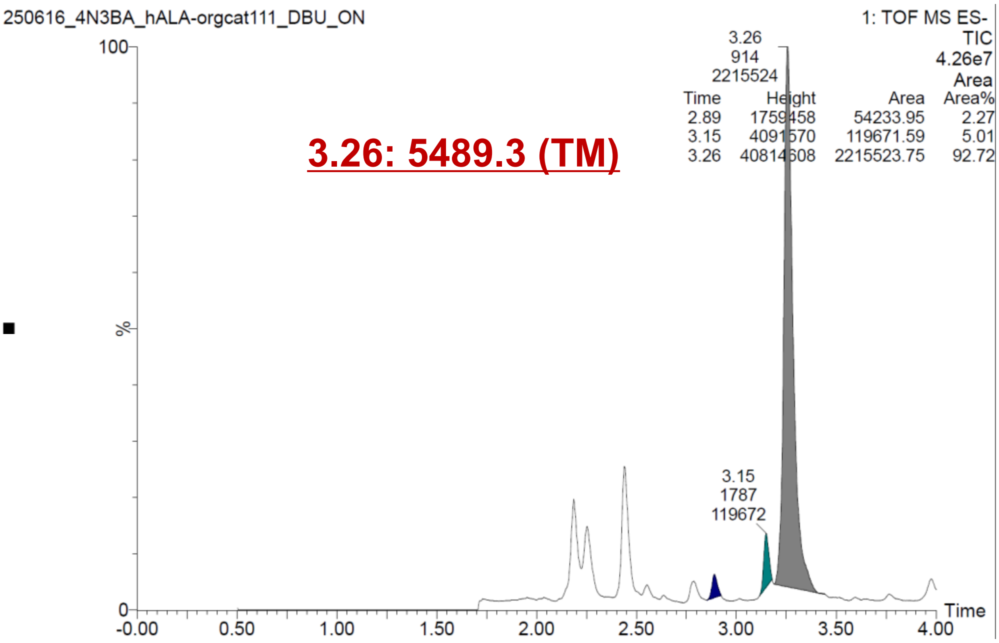

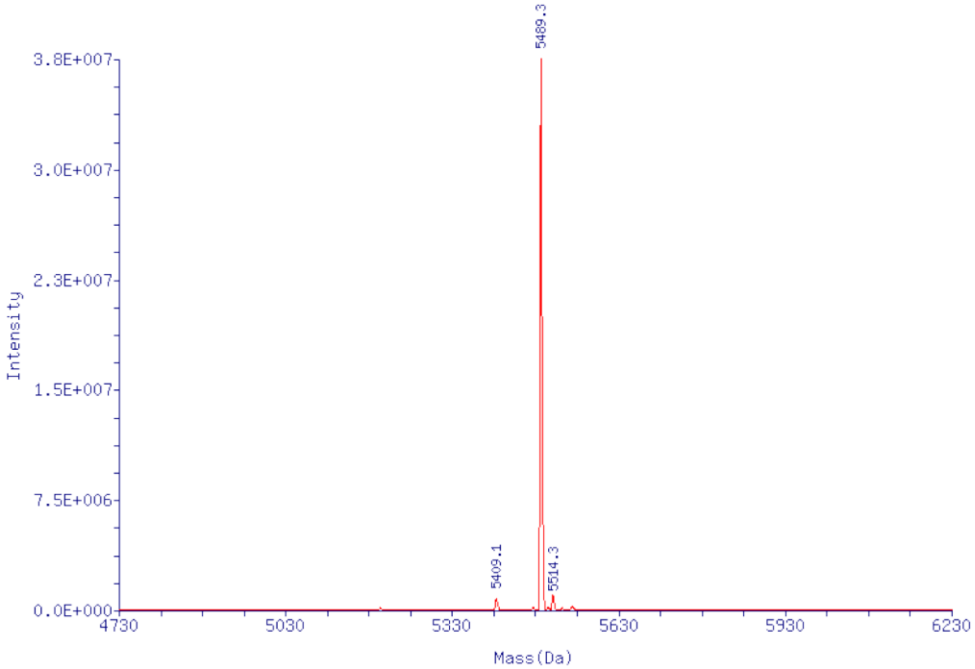

5l

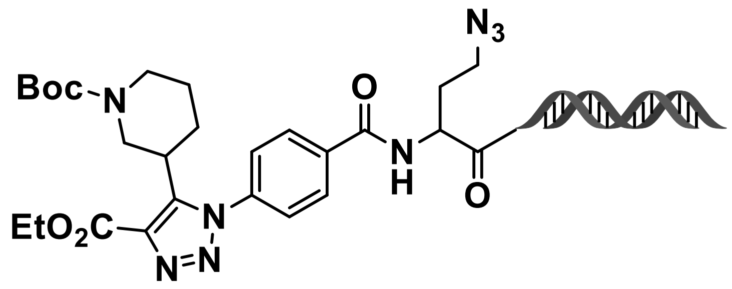

Conversion (Product%) = 94%
Base Peak Mass (Da): 5489.4

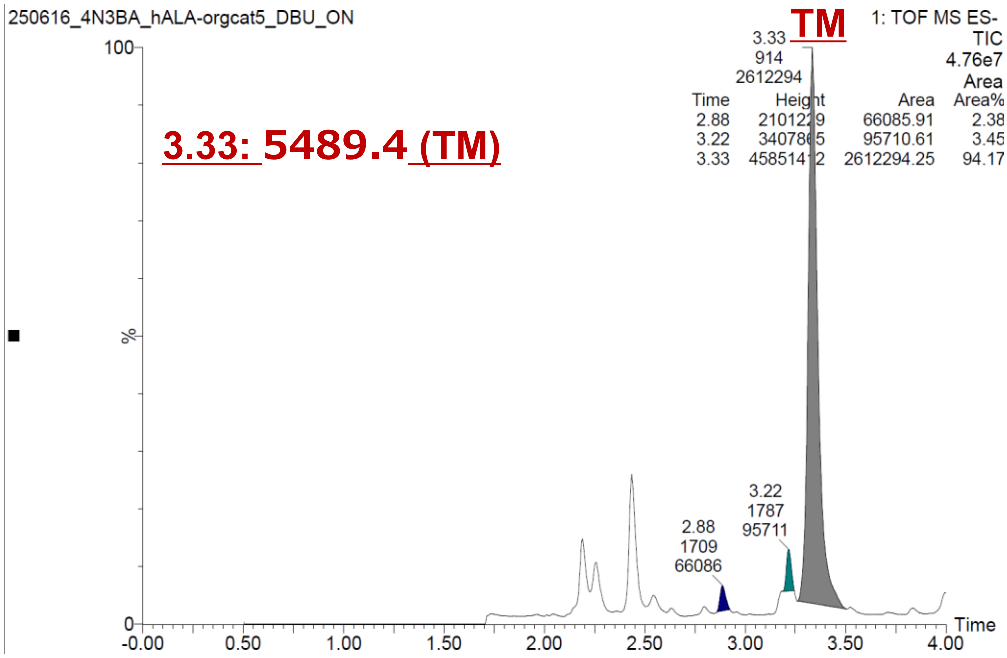

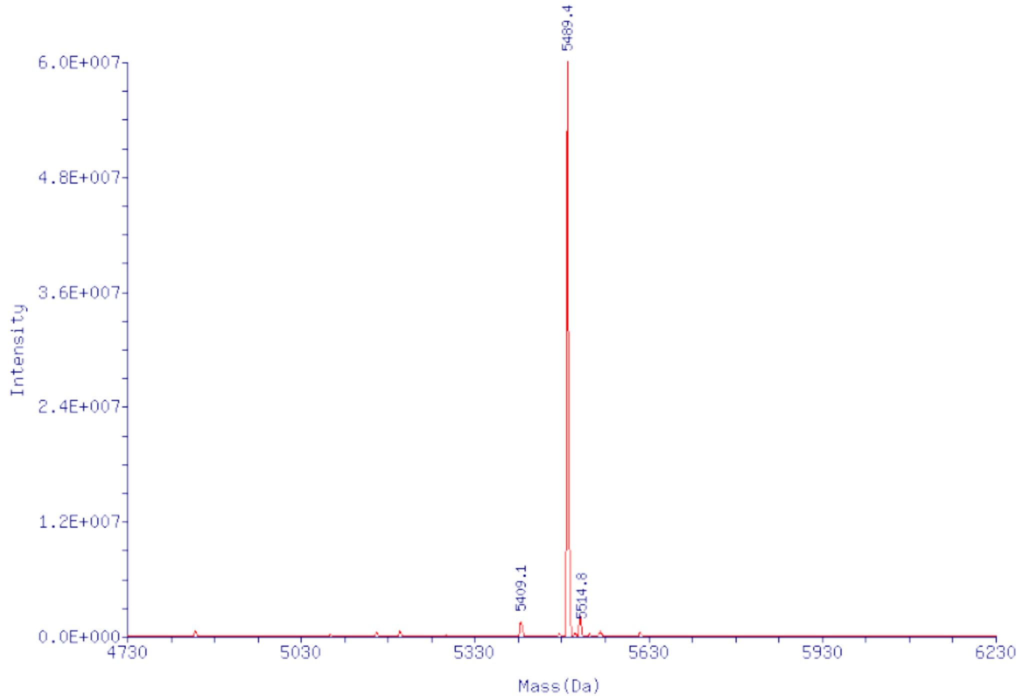

5m

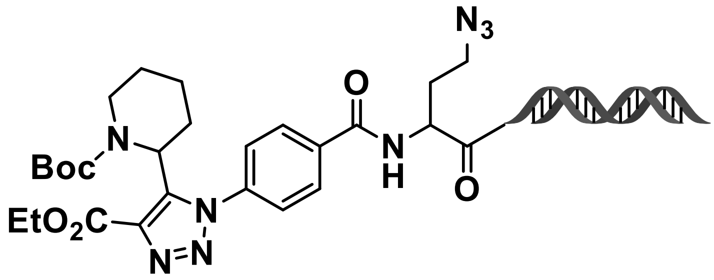

Conversion (Product%) = 88%
Base Peak Mass (Da): 5489.2

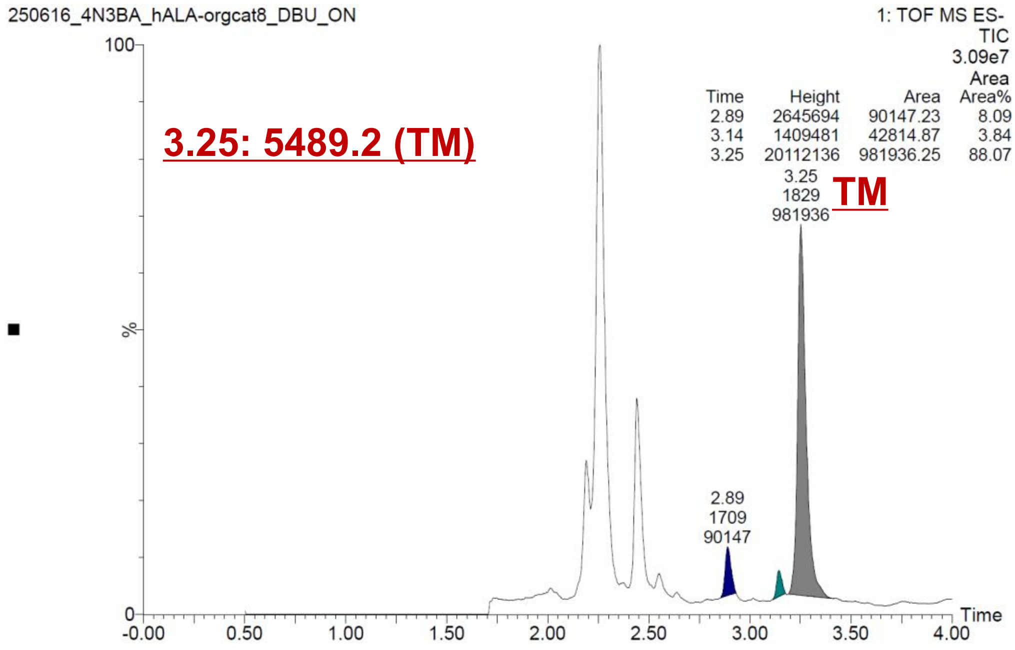

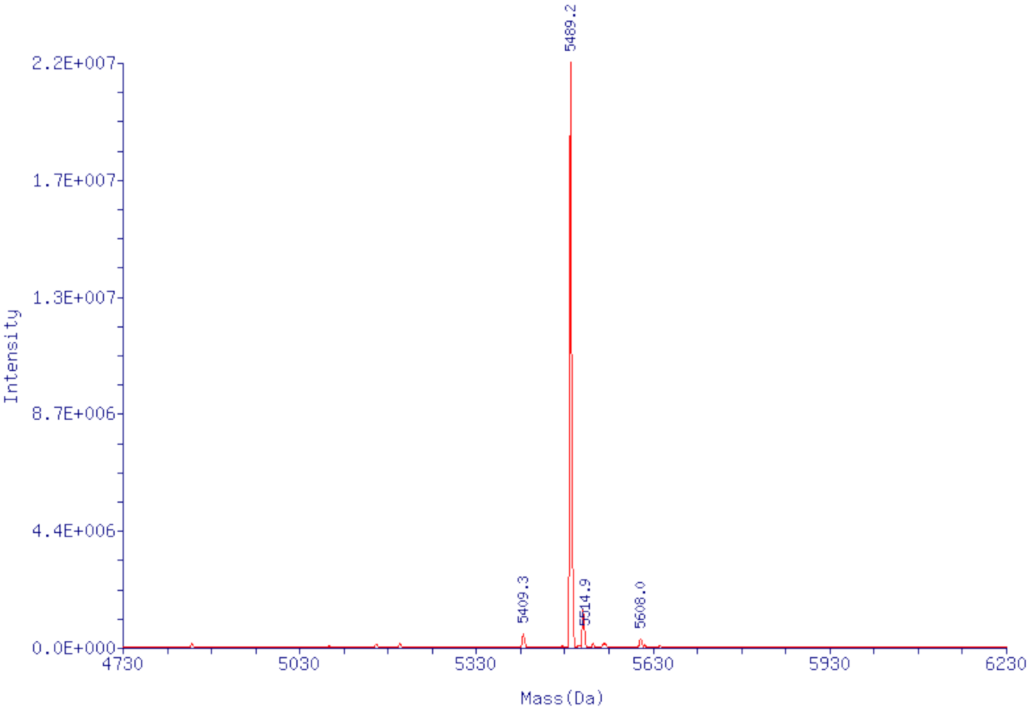

5n

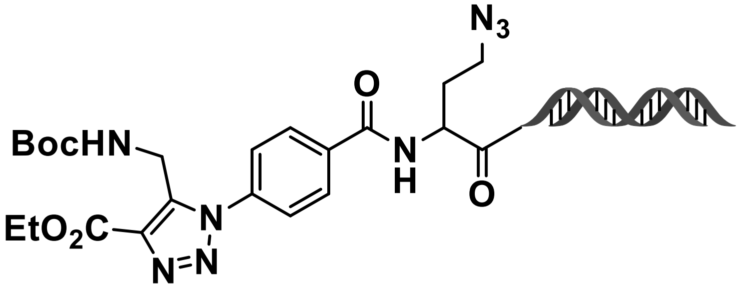

Conversion (Product%) = 92%
Base Peak Mass (Da): 5434.8

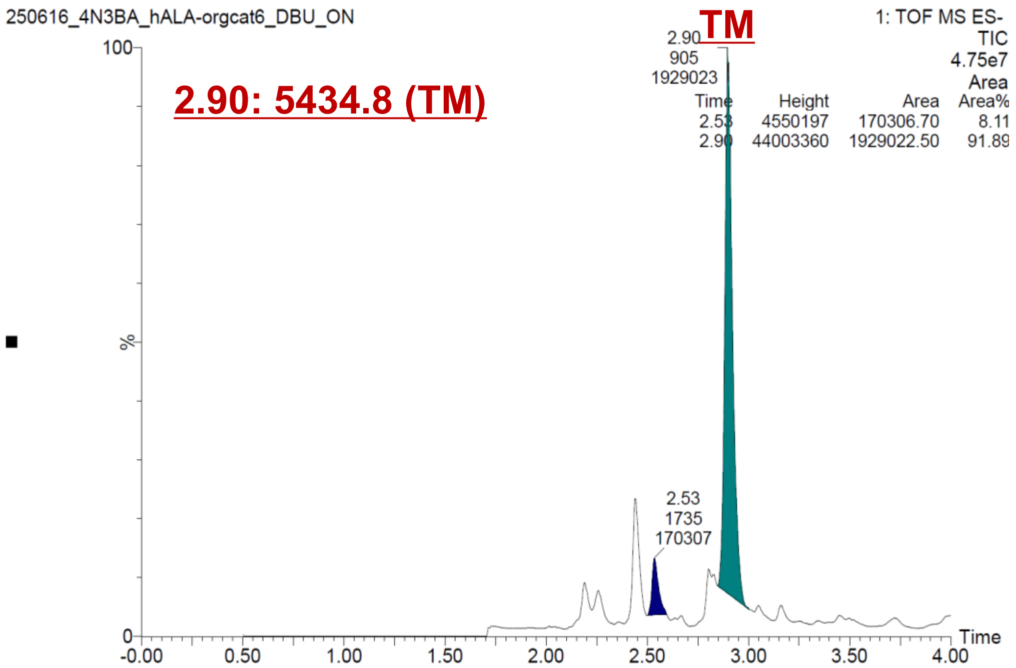

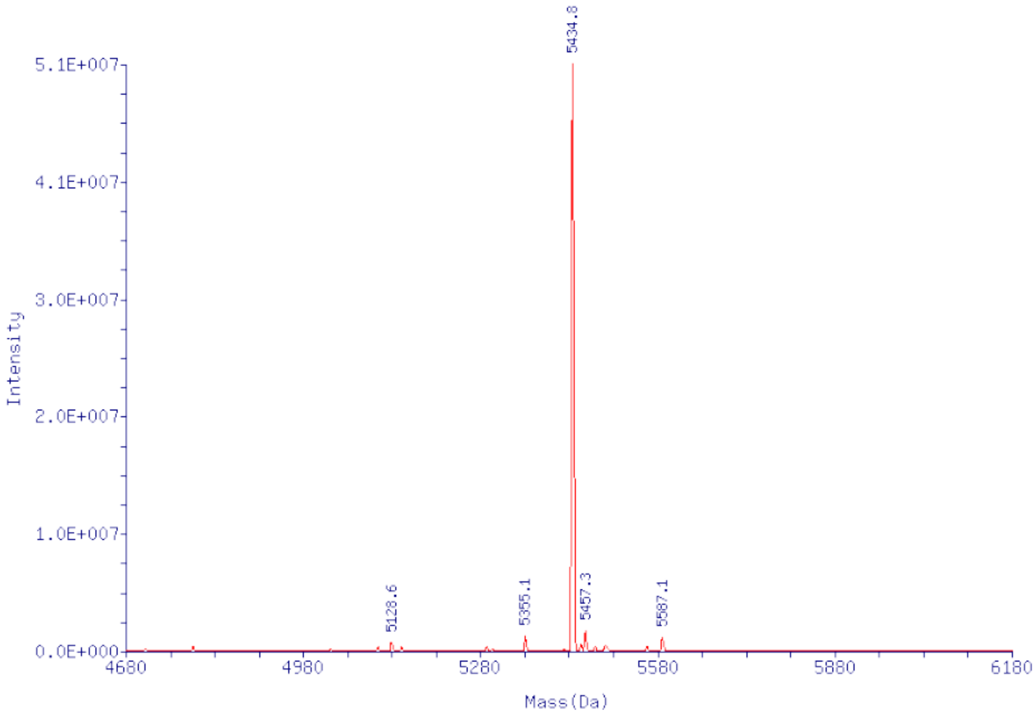

5o

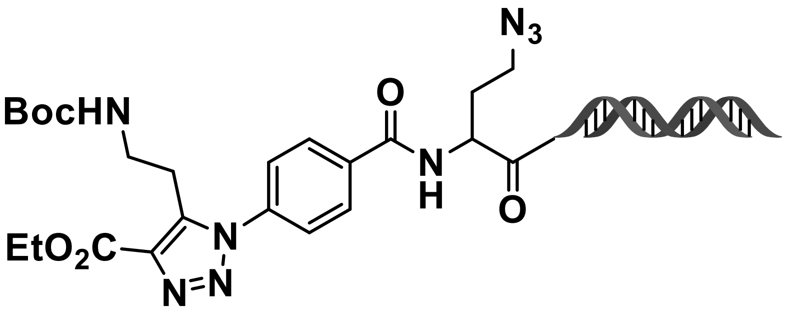

Conversion (Product%) = 86%
Base Peak Mass (Da): 5449.2

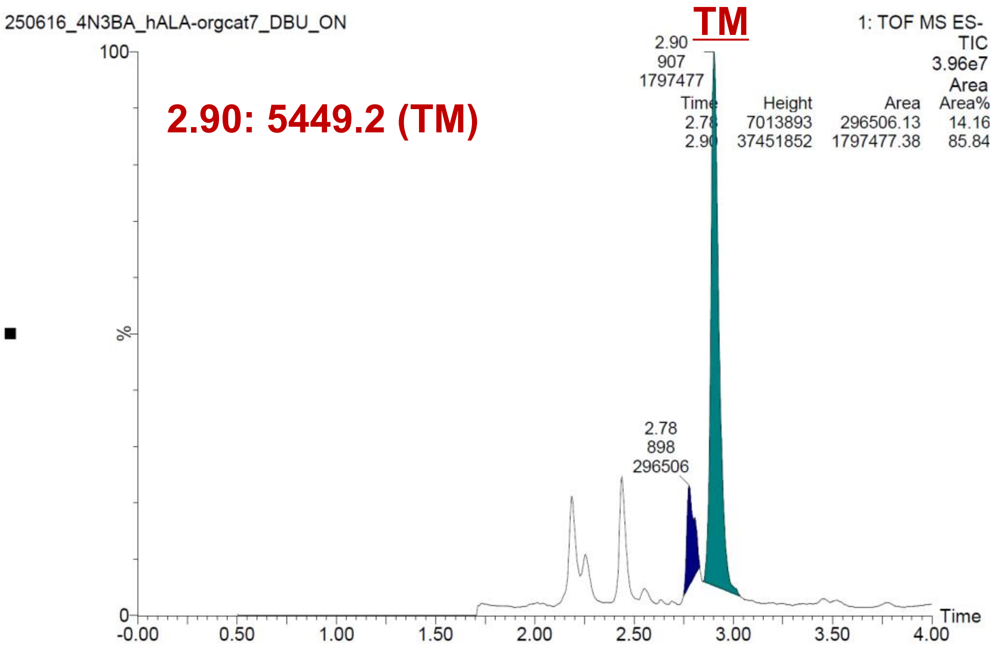

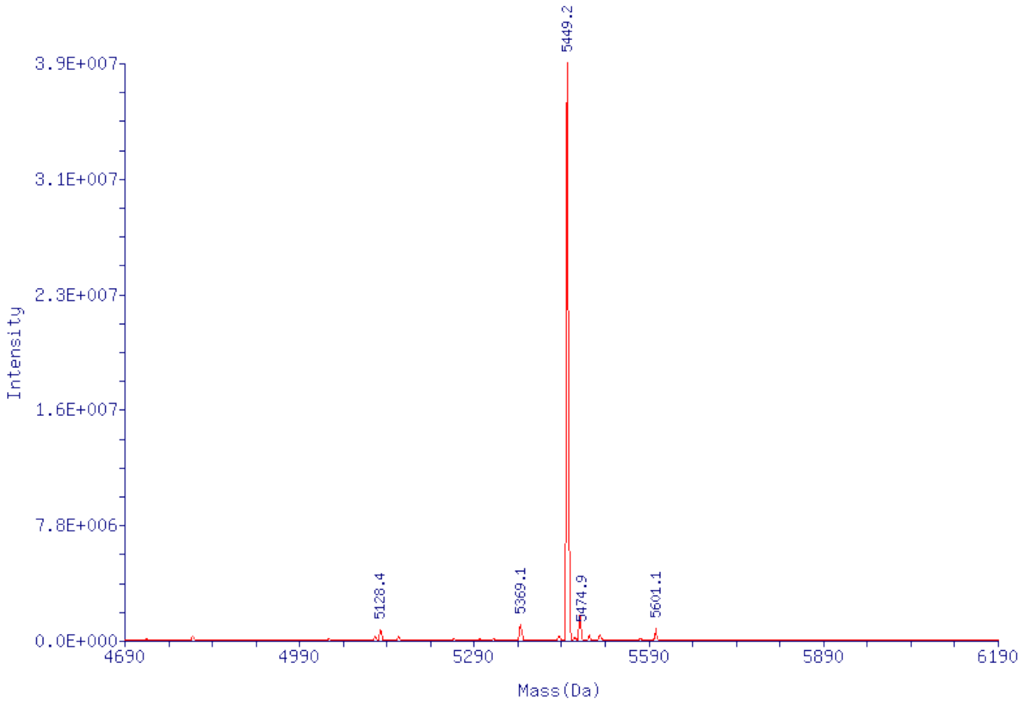

5p

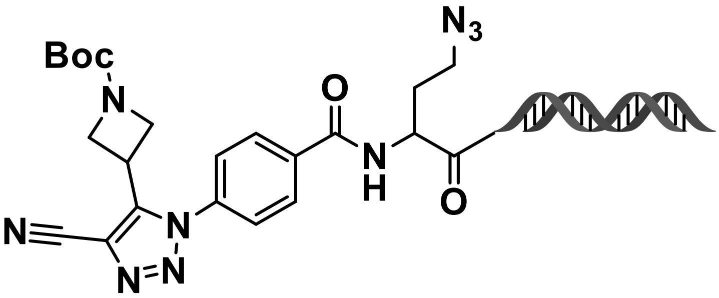

Conversion (Product%) = 83%
Base Peak Mass (Da): 5414.0

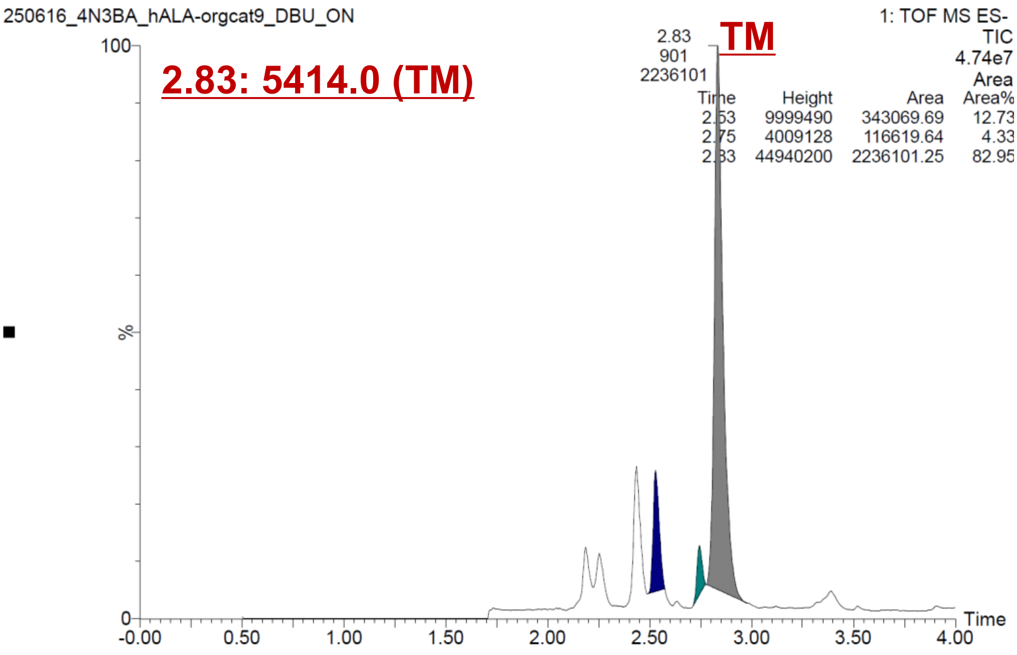

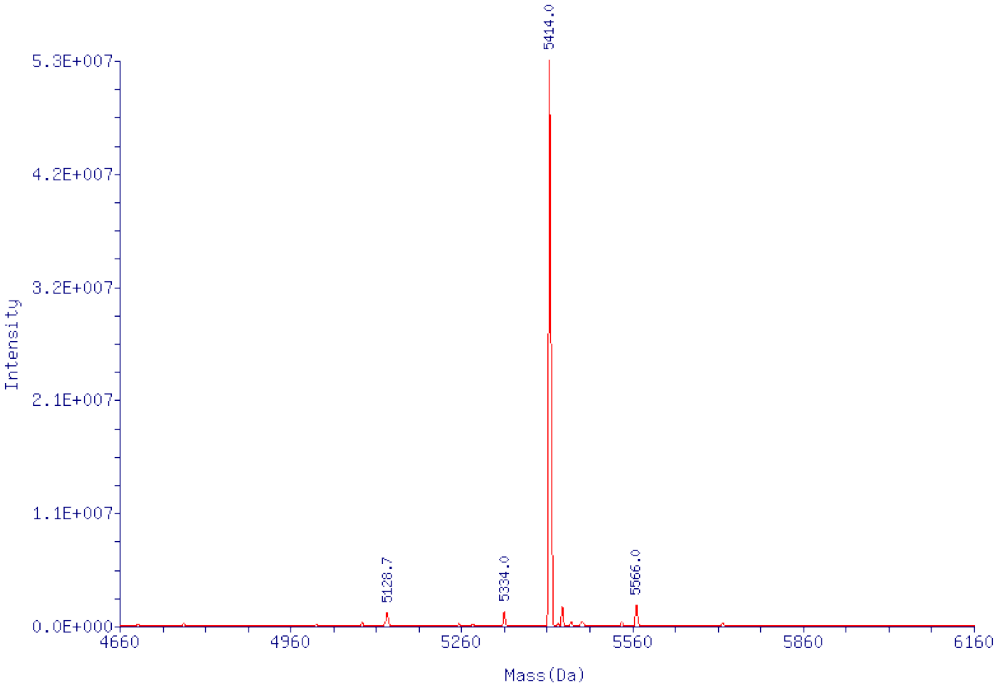

5q

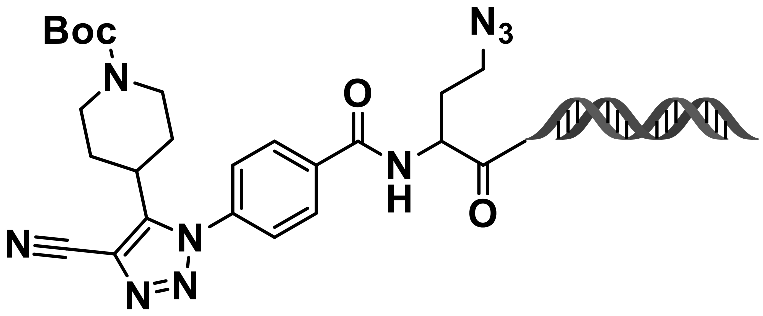

Conversion (Product%) = 93%
Base Peak Mass (Da): 5441.9

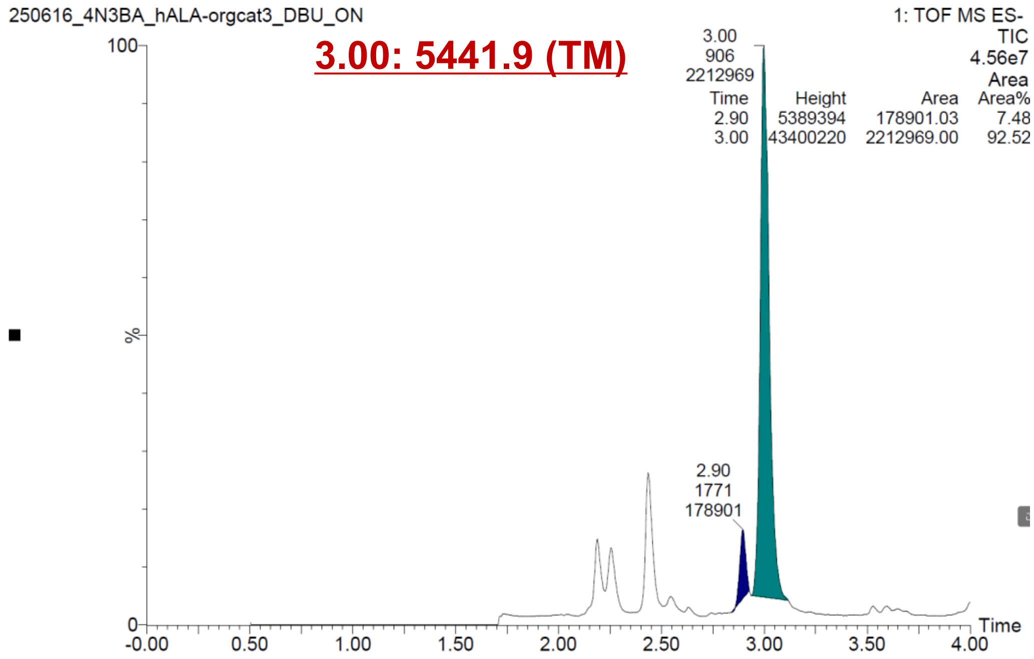

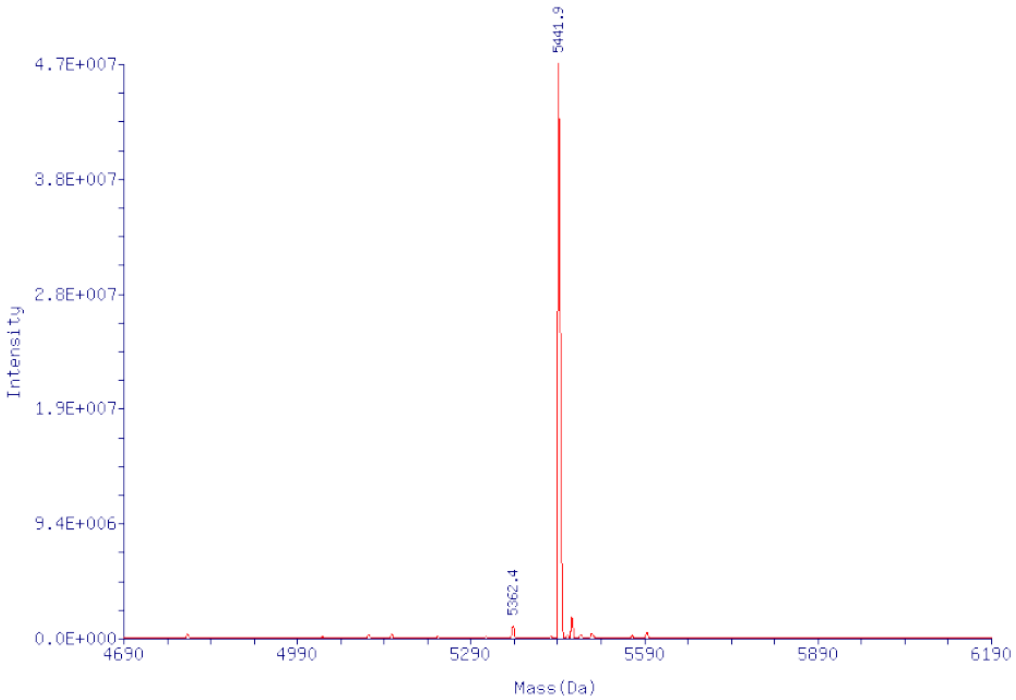

5r

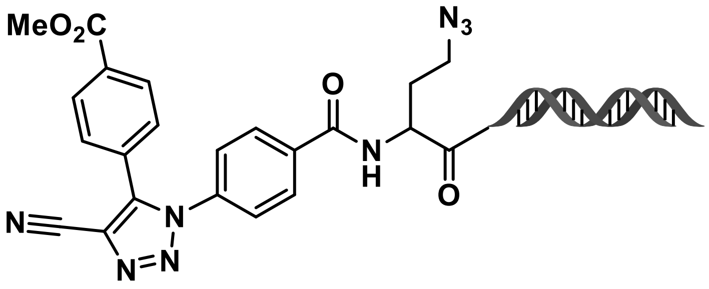

Conversion (Product%) = 93%
Base Peak Mass (Da): 5392.6

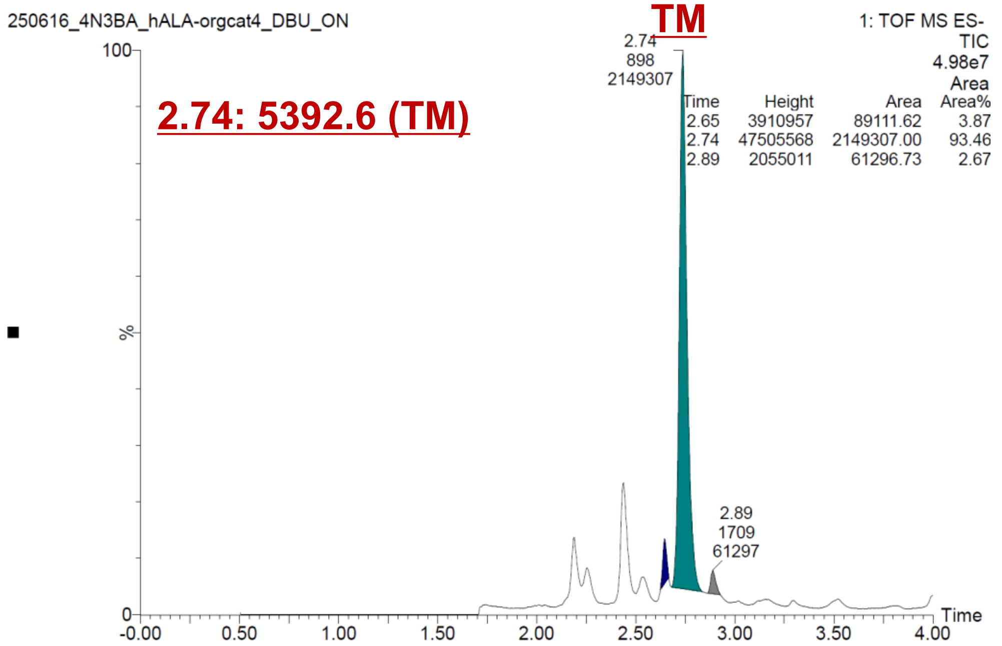

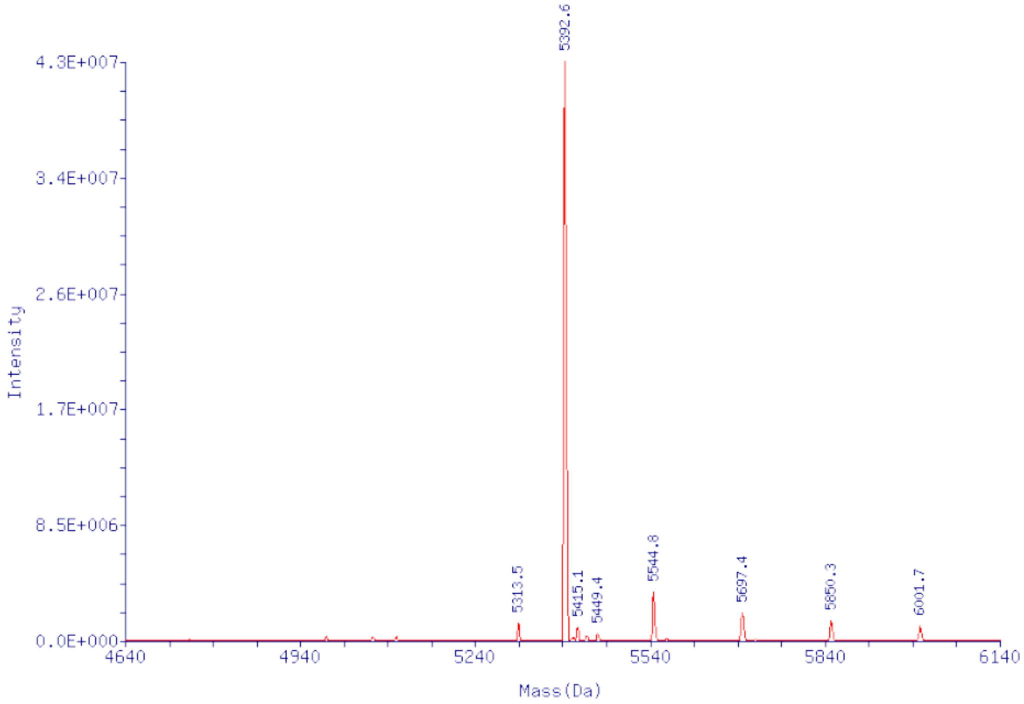

CuAAC reaction of 4N$_3$-BA-(*S*)-N$_3$-homoAla–HP

6-TM

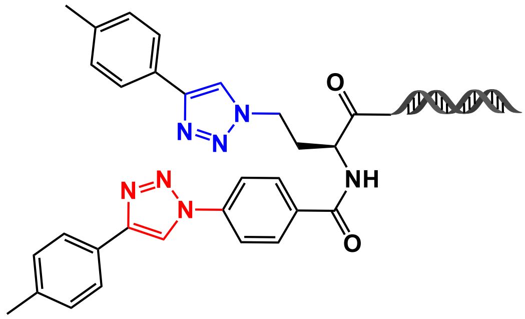

Conversion (Product%) = 59%
Base Peak Mass (Da): 5440.5

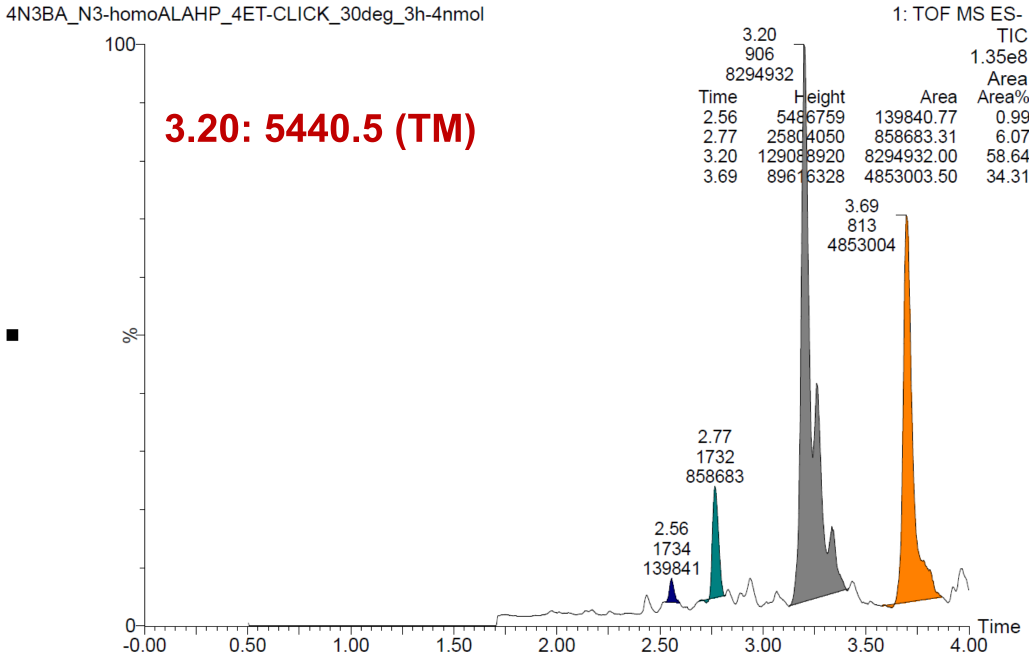

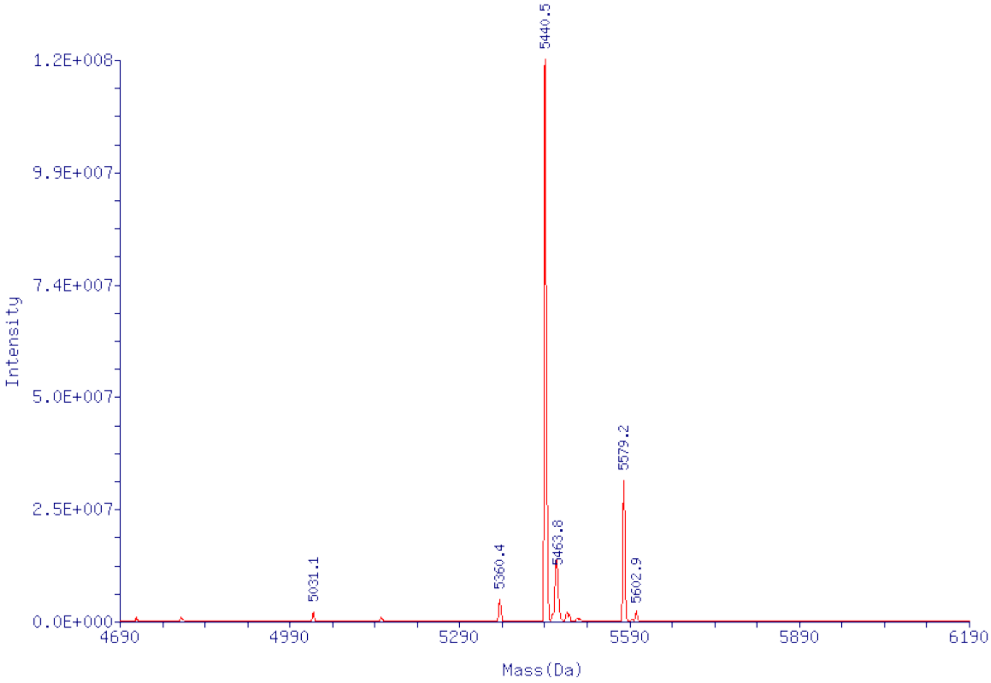

Azide reduction of 4N_3-BA-(*S*)-N_3-homoAla–HP

8-TM

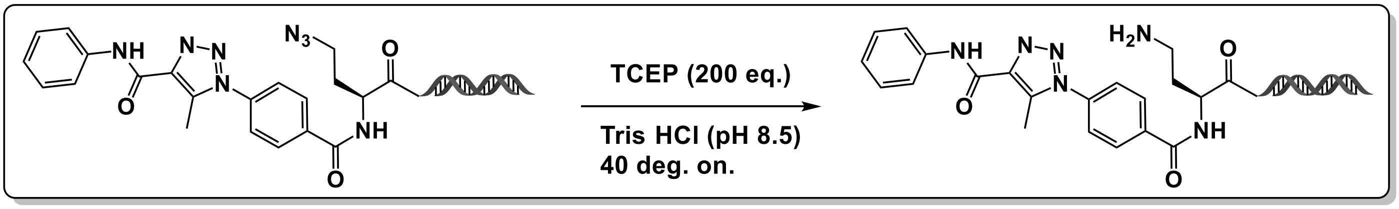

Conversion (Product%) = 75%
Base Peak Mass (Da): 5341.2

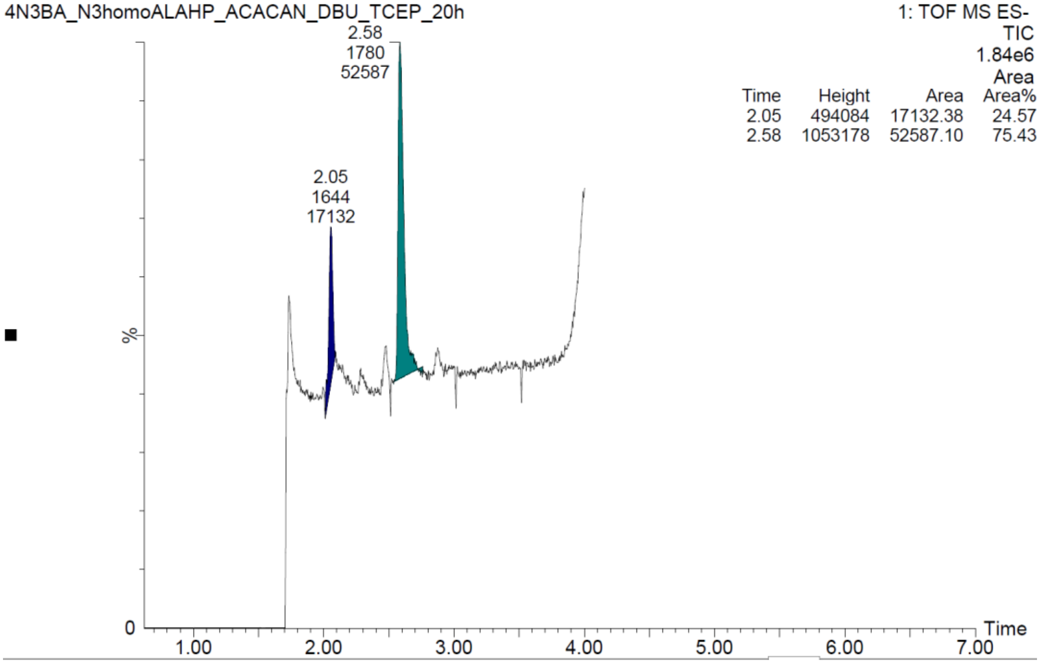

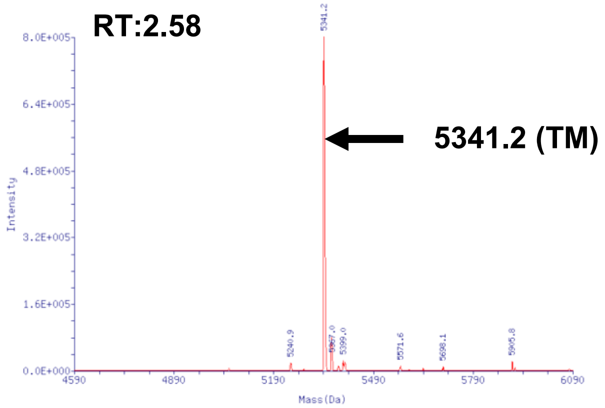

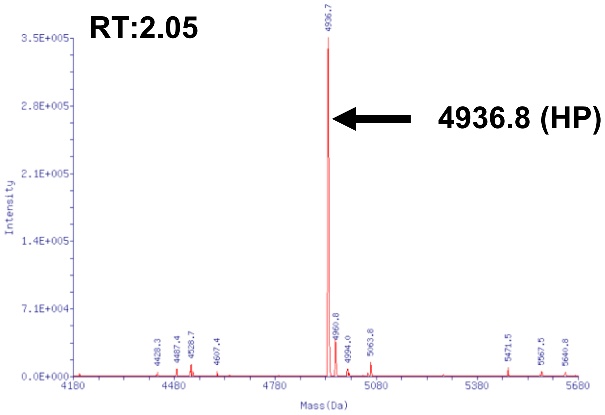

8a-TM

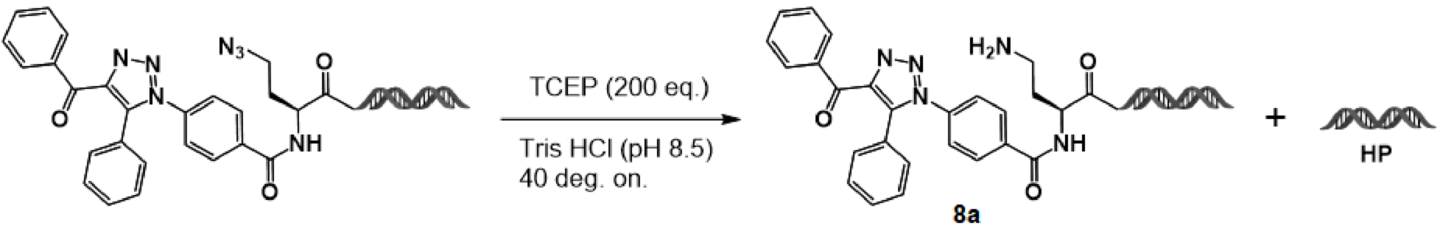

Conversion (Product%) = 82%
Base Peak Mass (Da): 5387.0

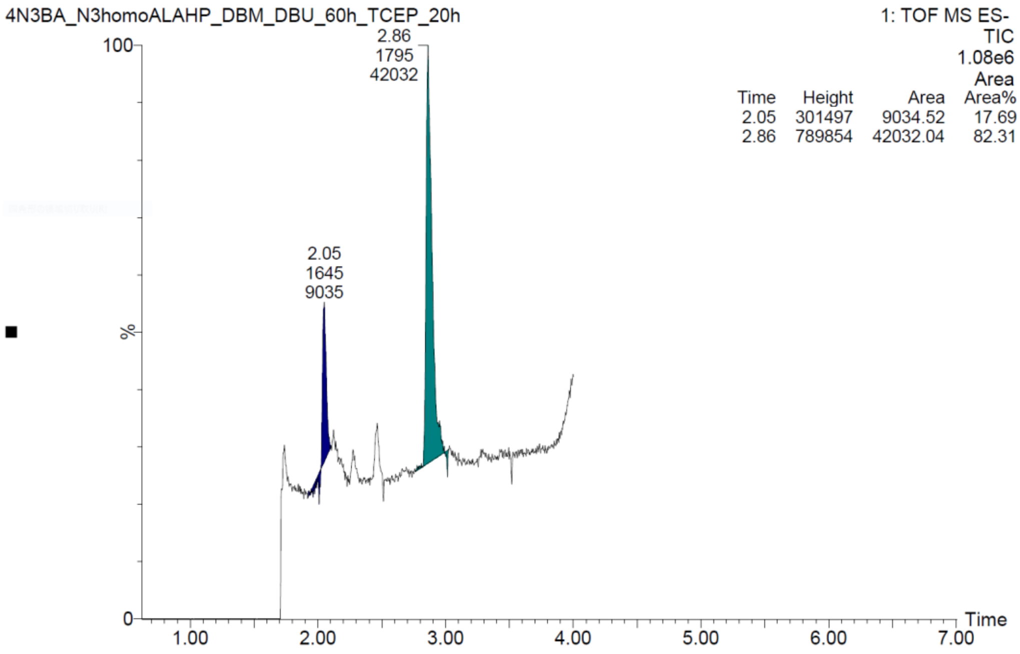

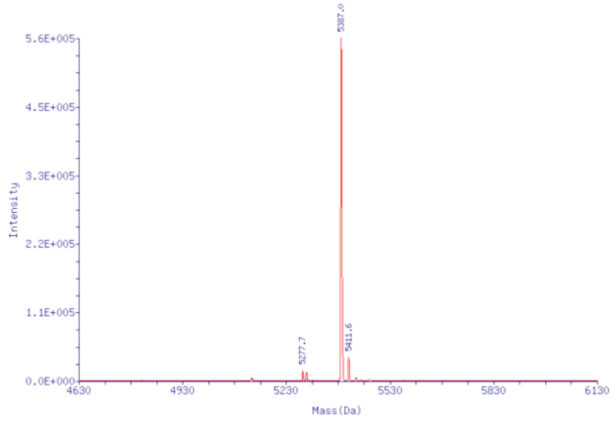

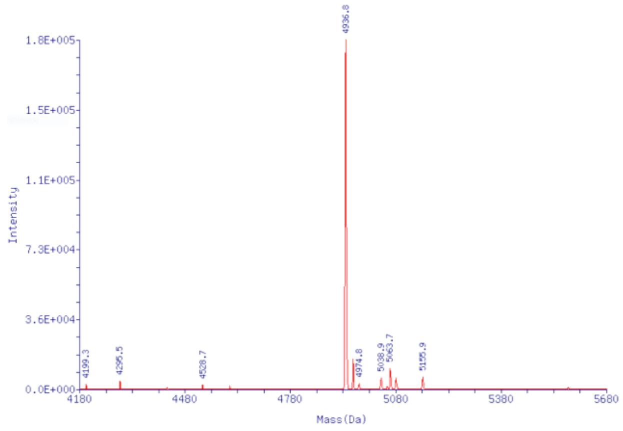

8b-TM

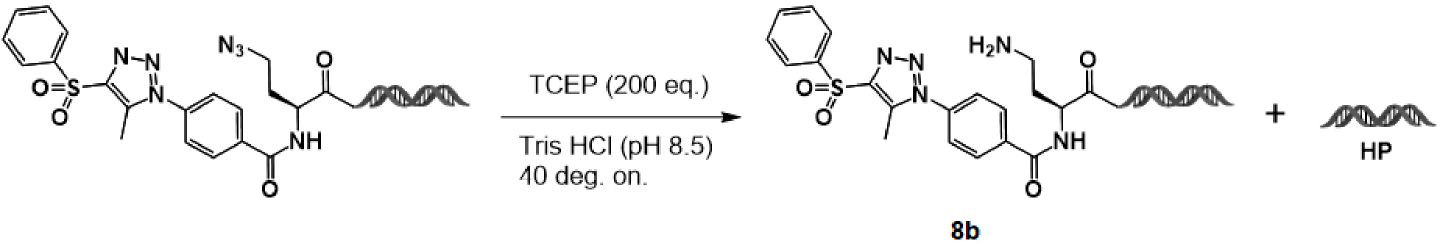

Conversion (Product%) = 63%
Base Peak Mass (Da): 5362.7

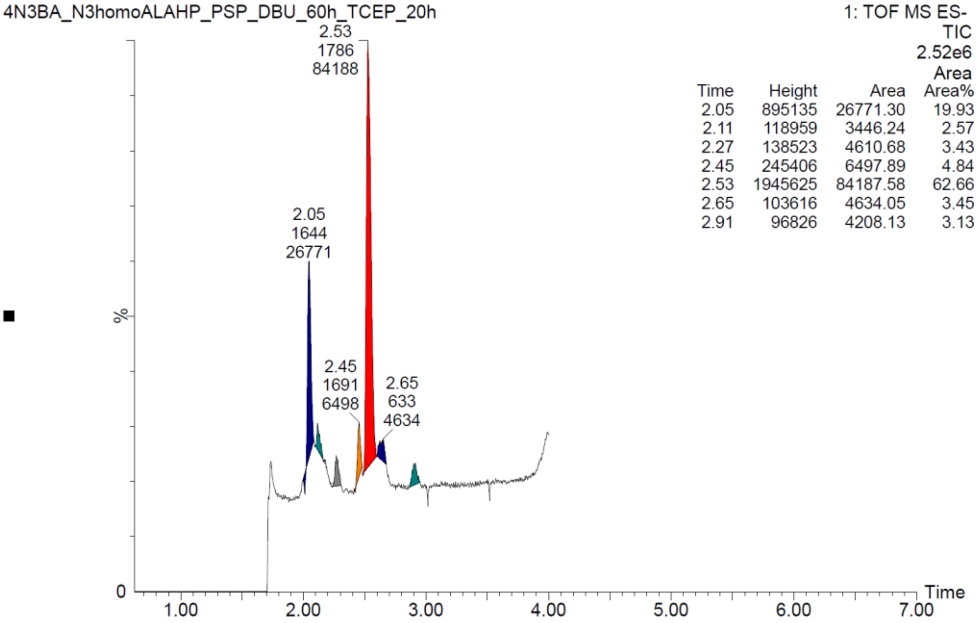

Time	Height	Area	Area%
2.05	895135	26771.30	19.93
2.11	118959	3446.24	2.57
2.27	138523	4610.68	3.43
2.45	245406	6497.89	4.84
2.53	1945625	84187.58	62.66
2.65	103616	4634.05	3.45
2.91	96826	4208.13	3.13

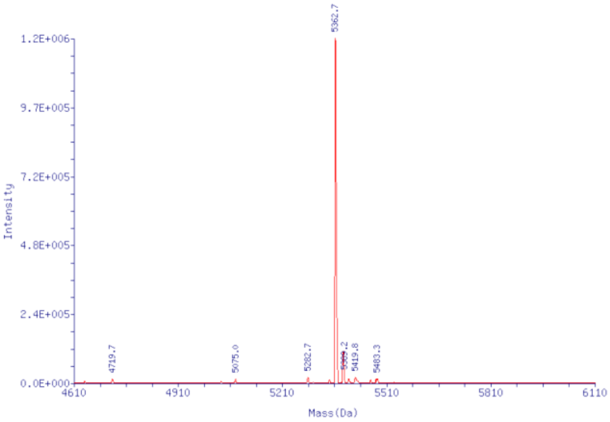

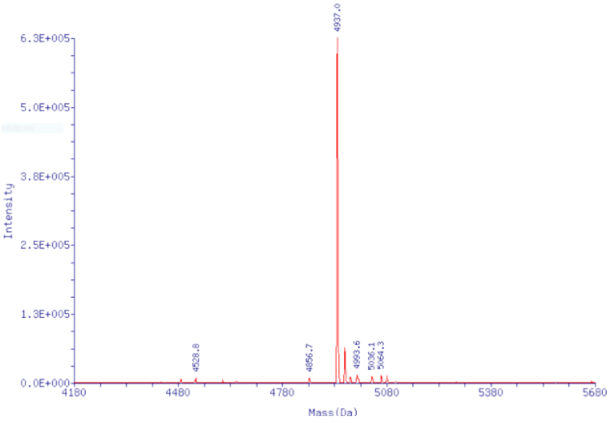

8c-TM

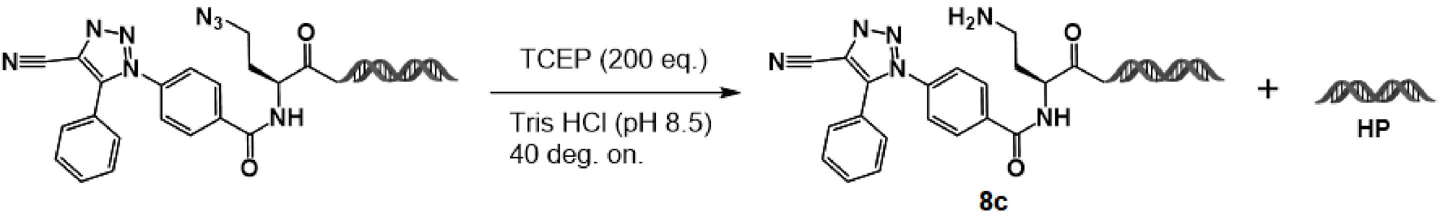

Conversion (Product%) = 75%
Base Peak Mass (Da): 5309.1

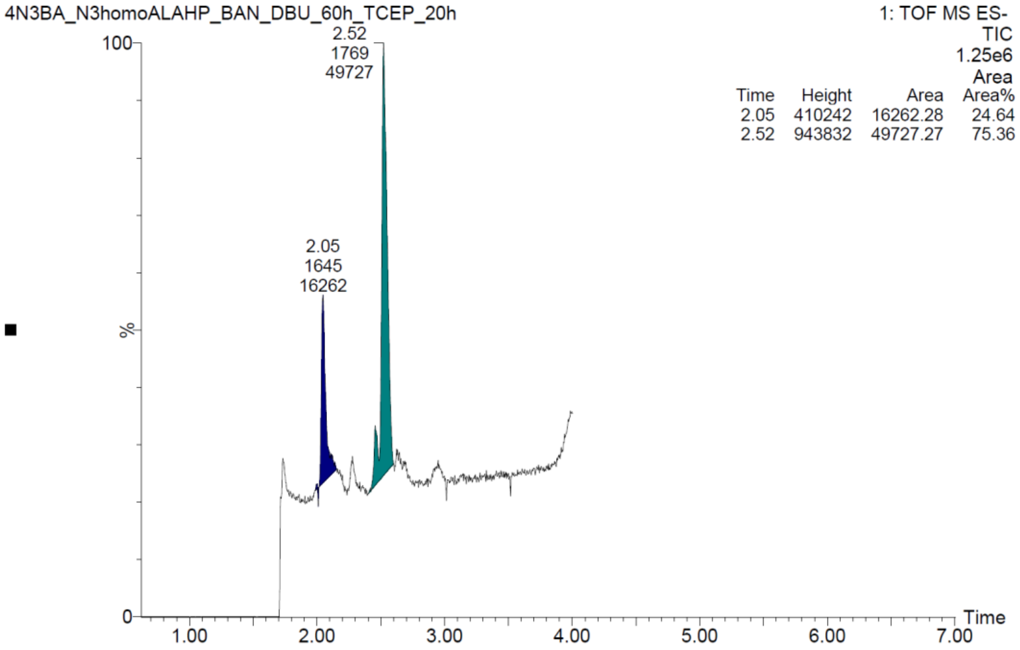

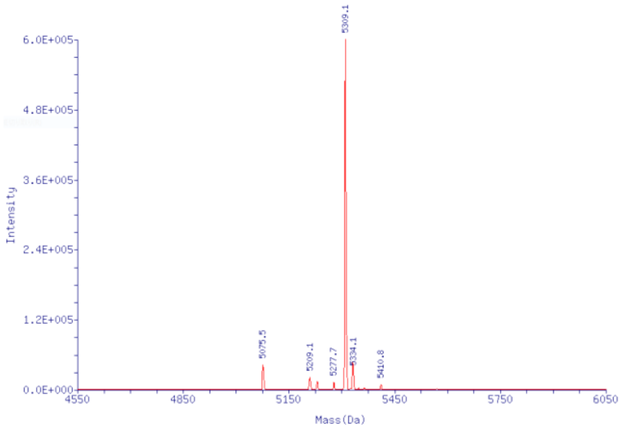

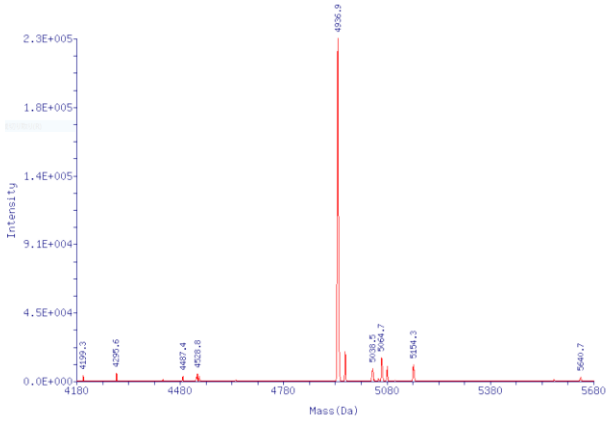

8d-TM

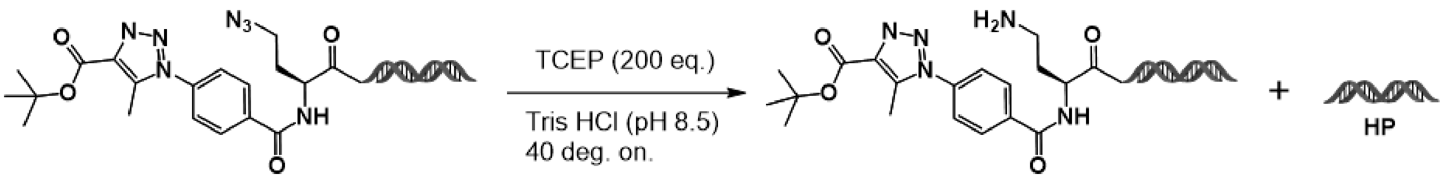

8d

Conversion (Product%) = 75%
Base Peak Mass (Da): 5384.8

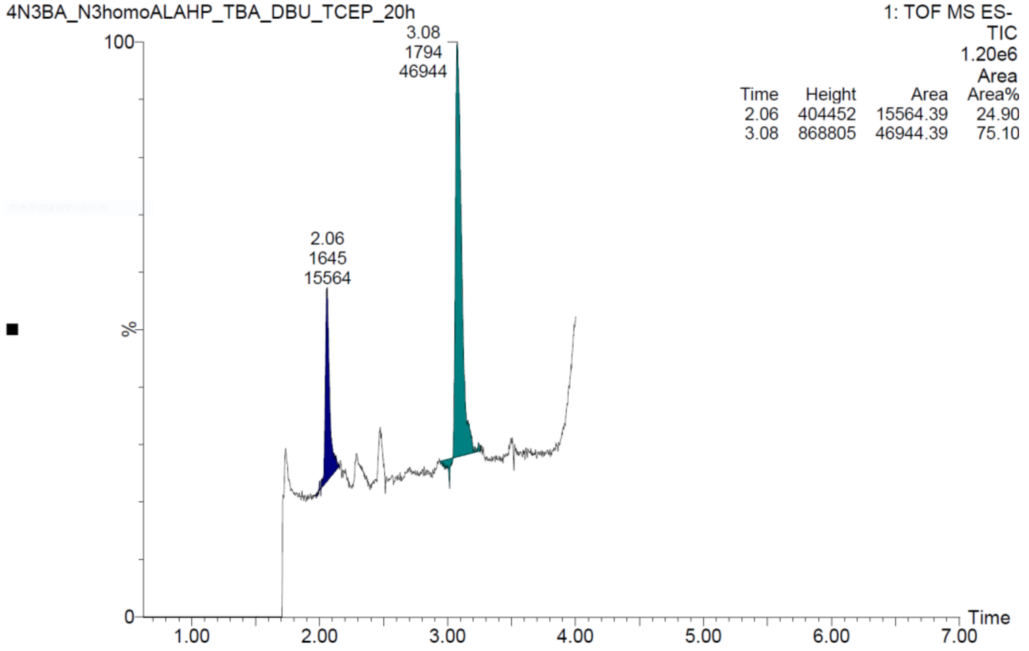

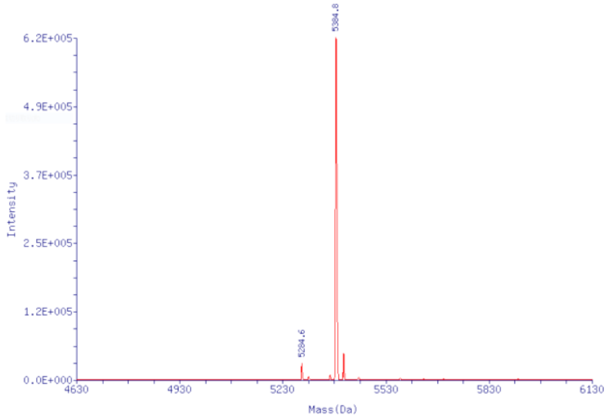

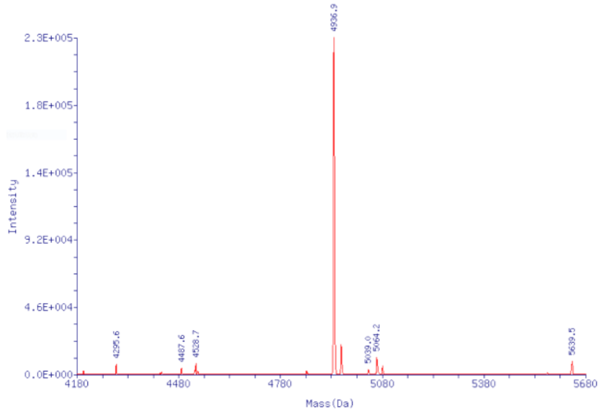

8e-TM

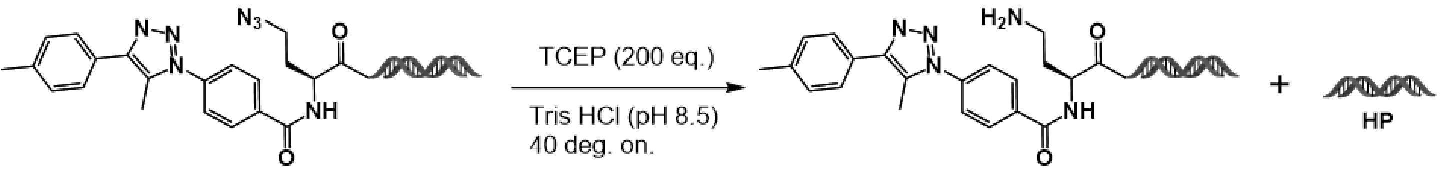

8e

Conversion (Product%) = 80%
Base Peak Mass (Da): 5312.2

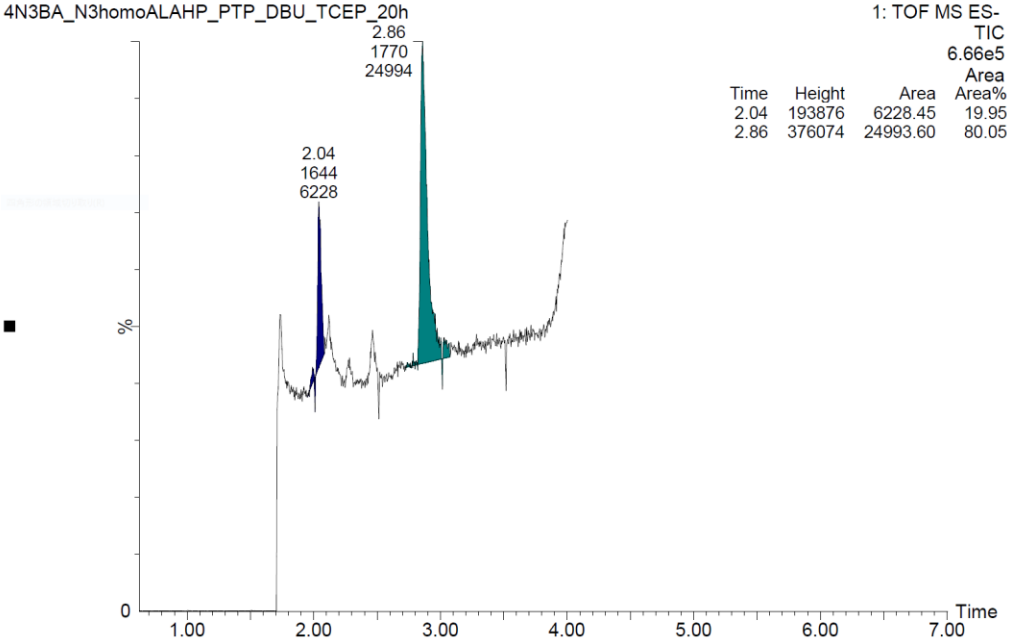

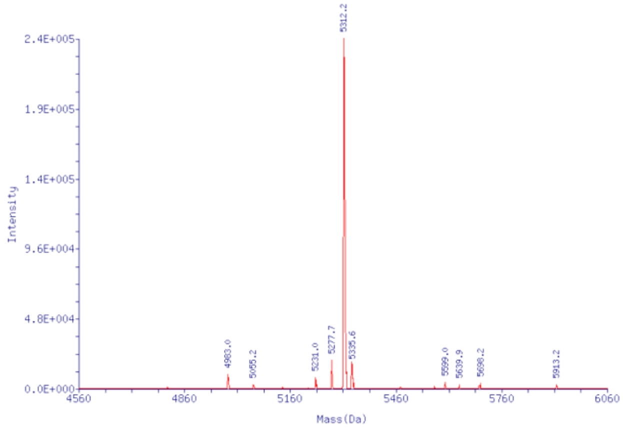

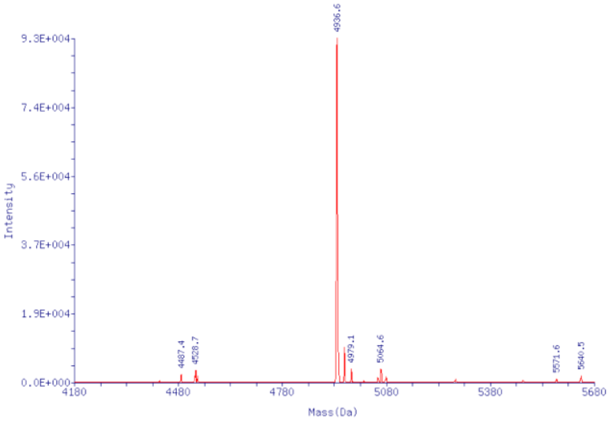

Preparation of a Novel On-DNA Platform Molecule: N-(4-Azidobenzoyl)-azide-proline-HP (Pro-based DAP)

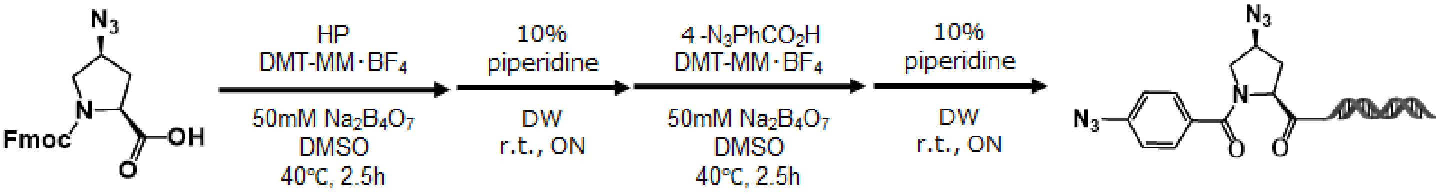

Conversion (Product%) = 78%
Base Peak Mass (Da): 5220.0

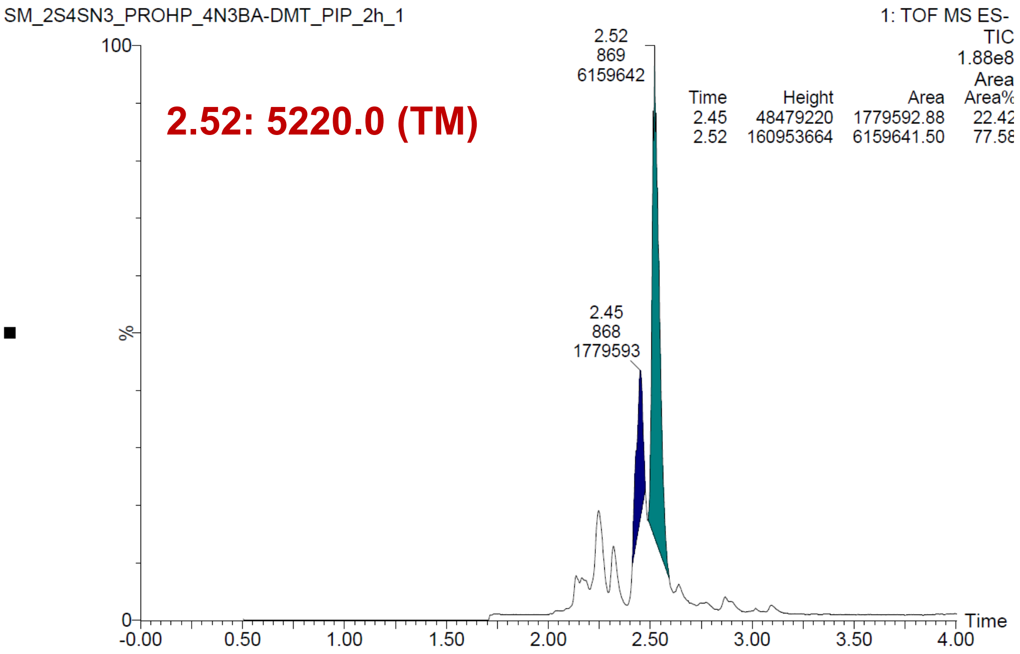

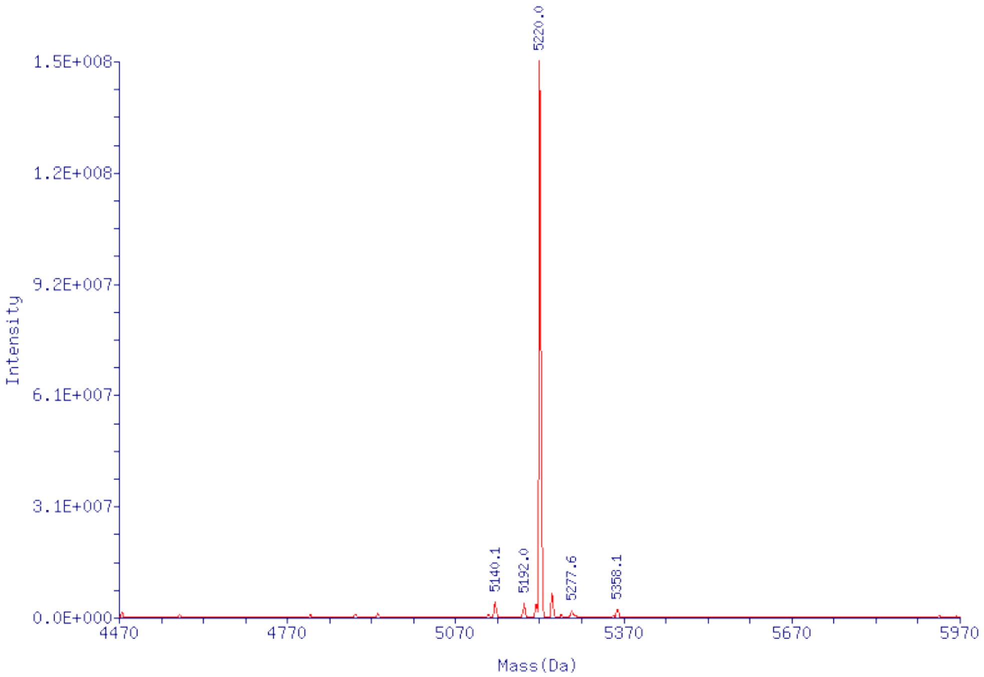

Reduction of the azide group of Pro-based DAP

10-TM

11-TM

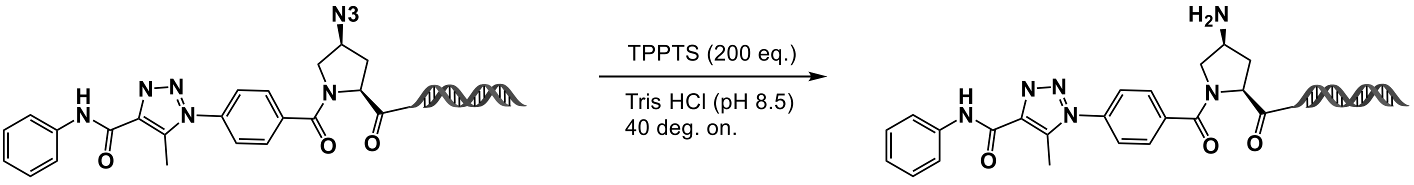

10

11

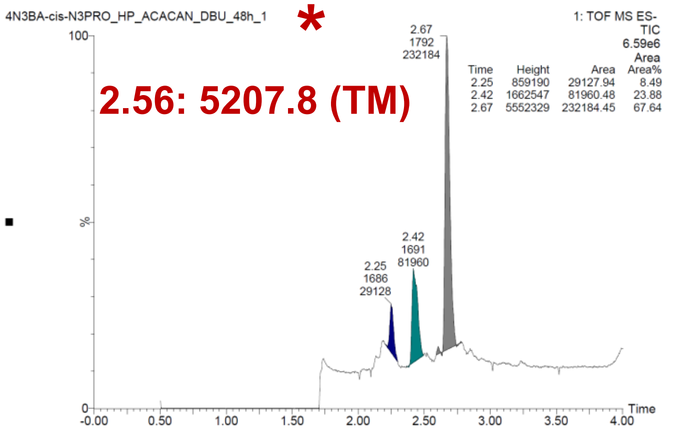

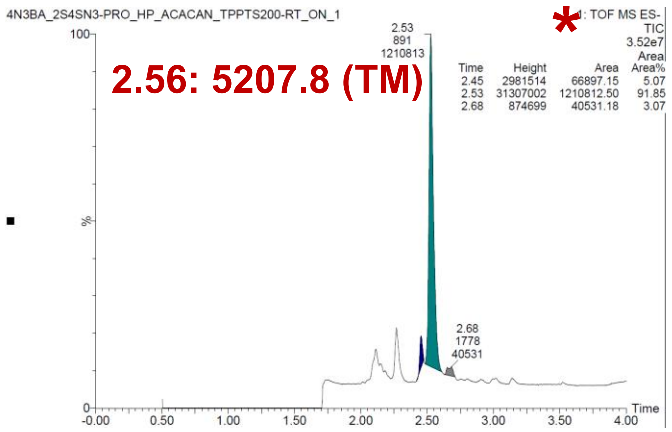

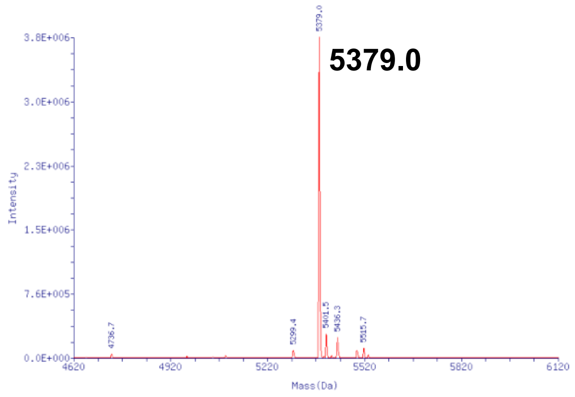

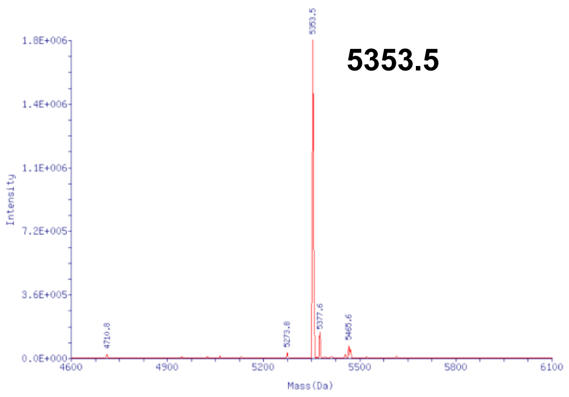

Stepwise Construction of On DNA compounds Using 4N_3-BA-(*S*)-N_3-homoAla–HP (Double-Click Strategy)

14a-TM

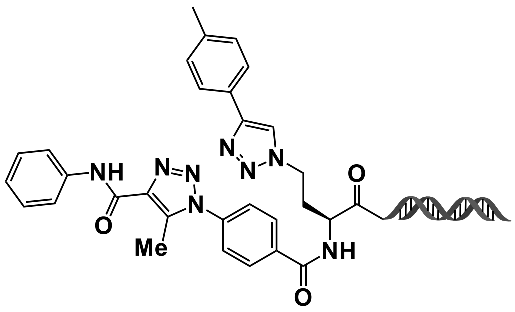

Conversion (Product%) = 89%
Base Peak Mass (Da): 5495.3

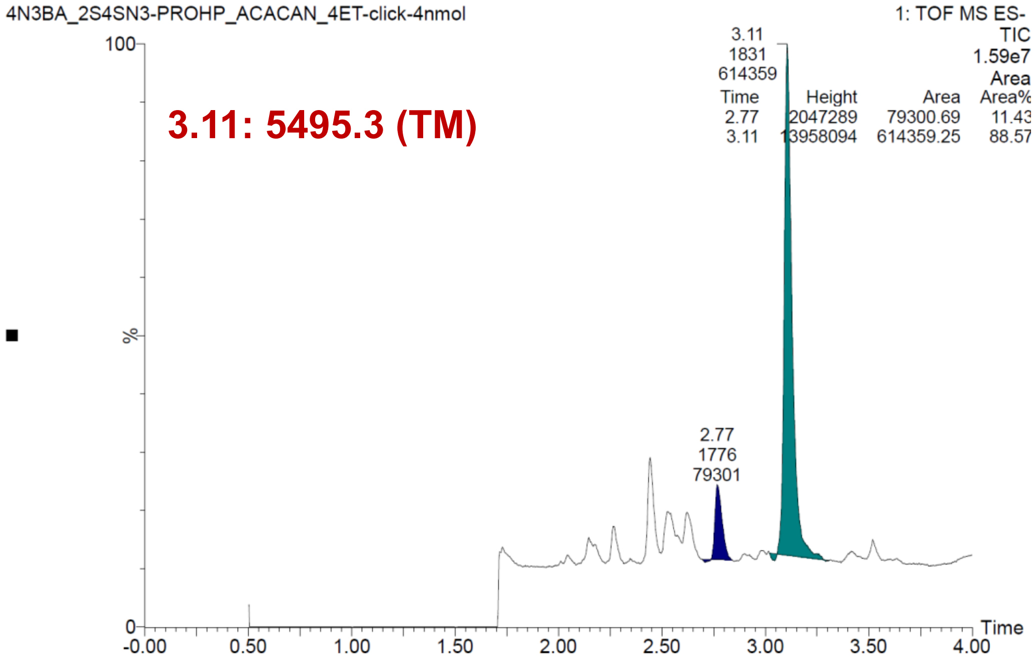

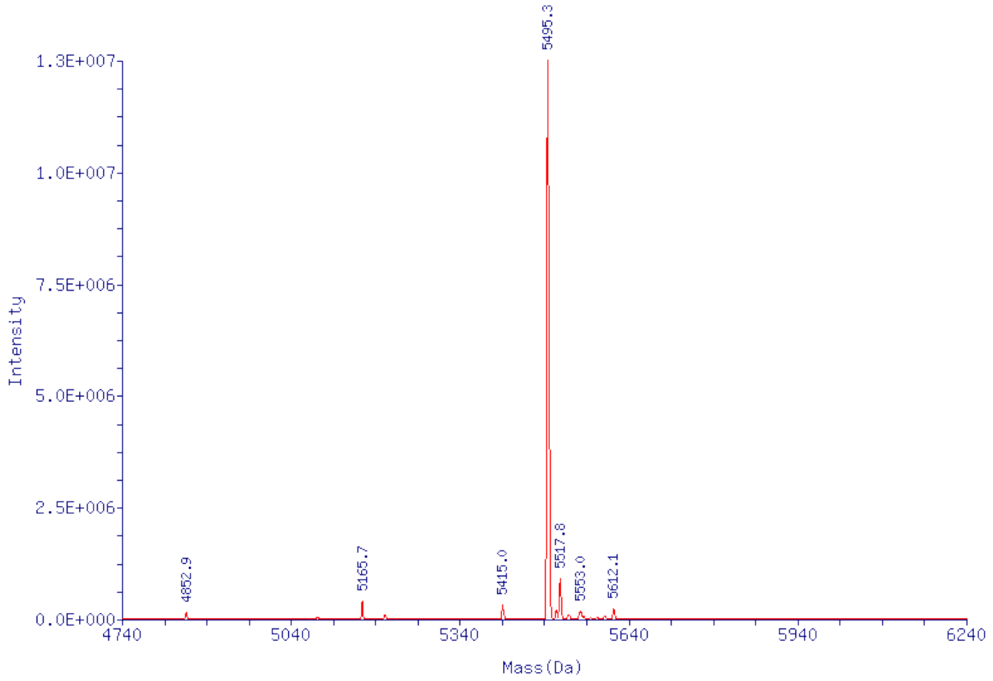

14b-TM

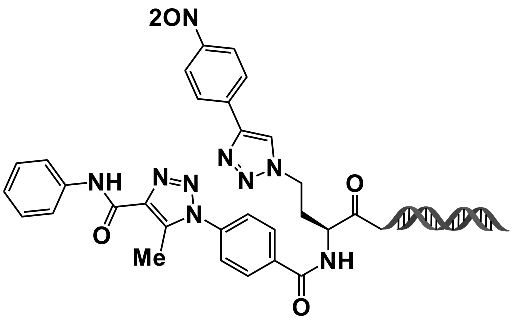

Conversion (Product%) = 92%
Base Peak Mass (Da): 5526.5

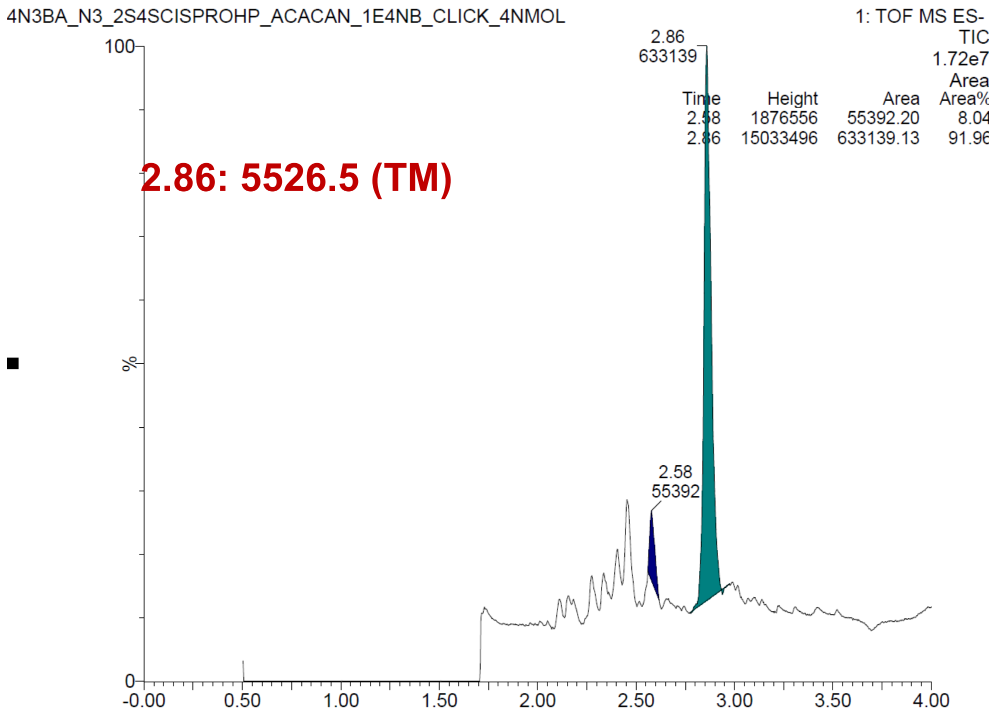

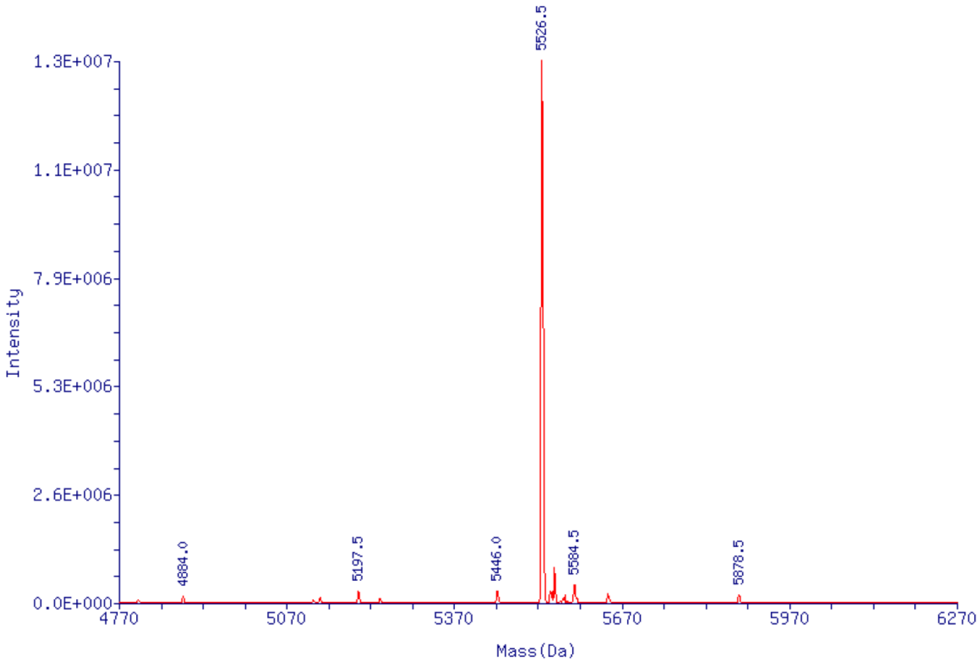

14c-TM

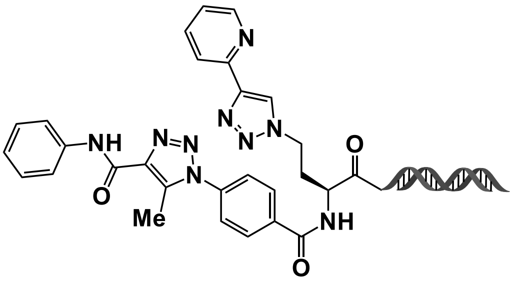

Conversion (Product%) = 90%
Base Peak Mass (Da): 5482.5

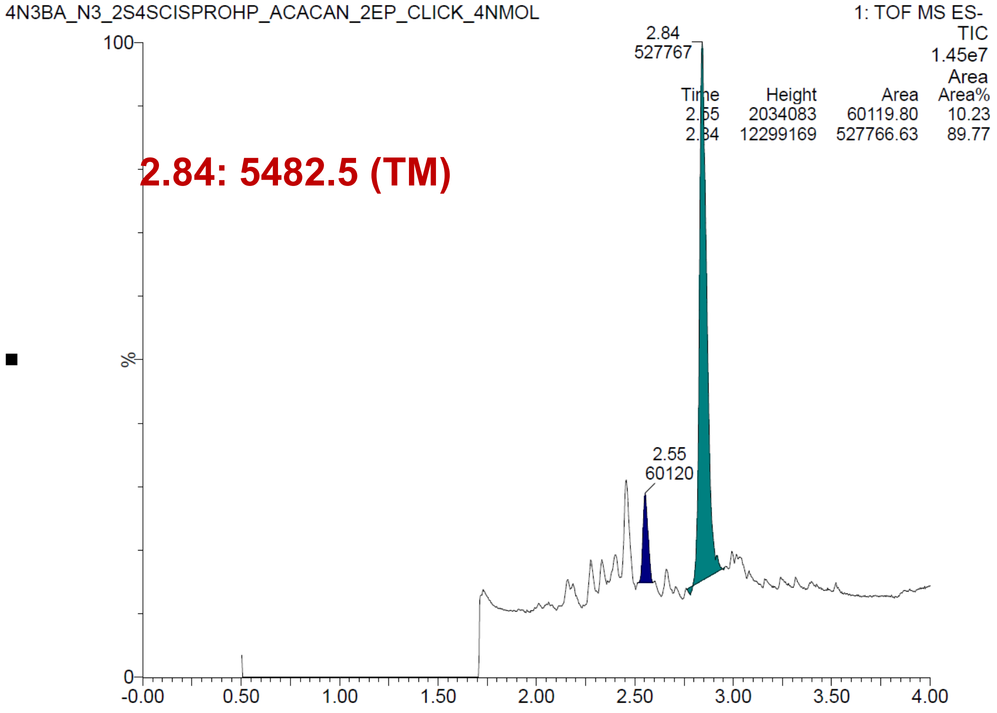

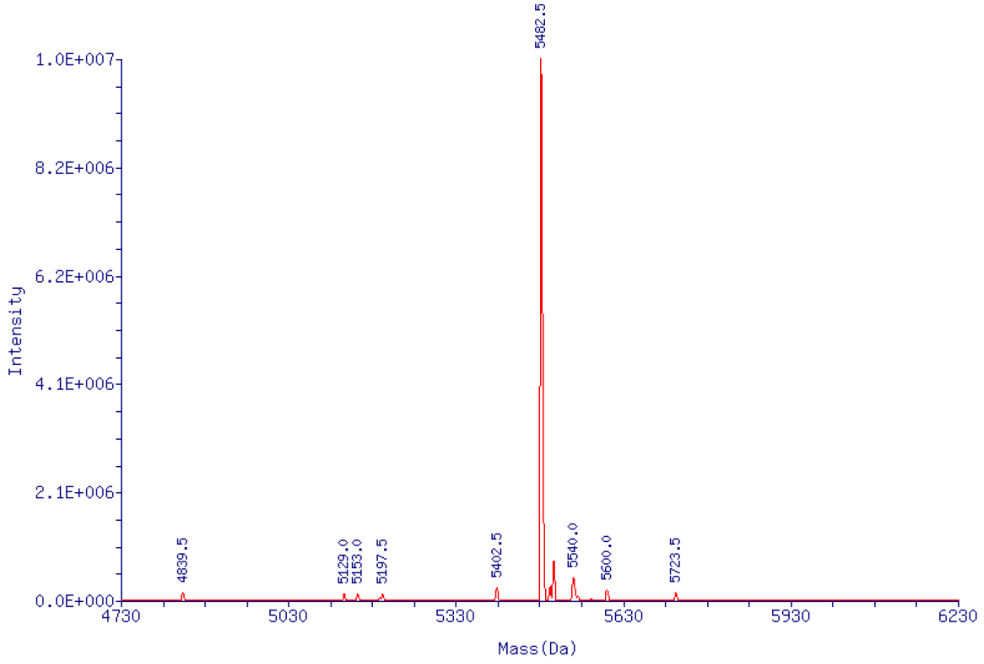

14d-TM

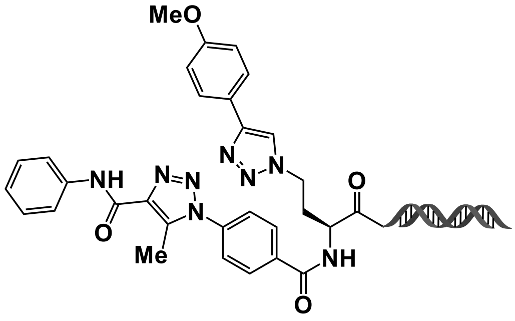

Conversion (Product%) = 89%
Base Peak Mass (Da): 5511.0

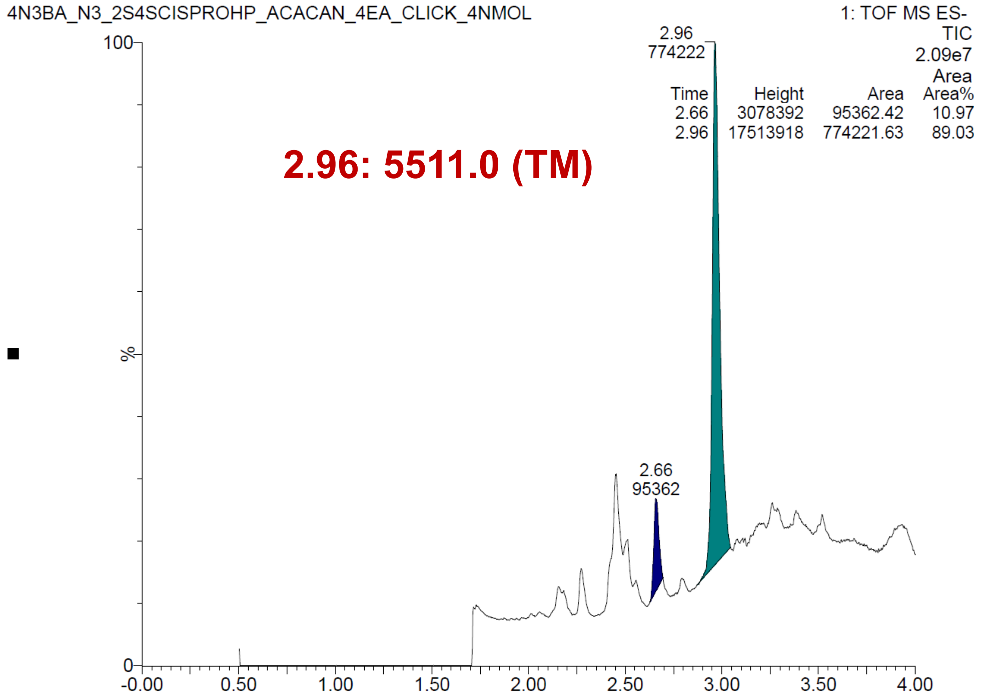

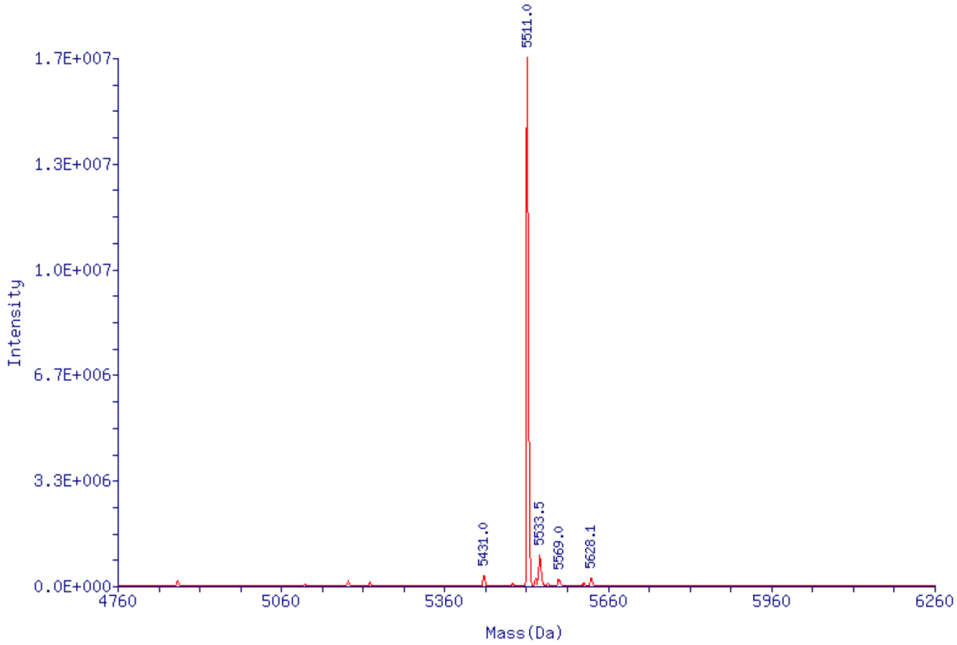

14e-TM

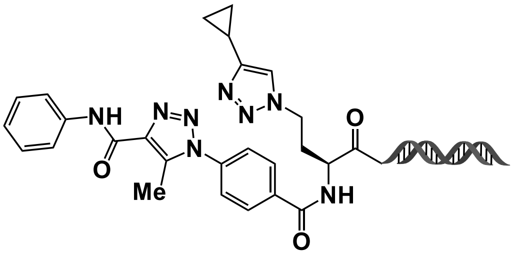

14e

Conversion (Product%) = 90%
Base Peak Mass (Da): 5445.5

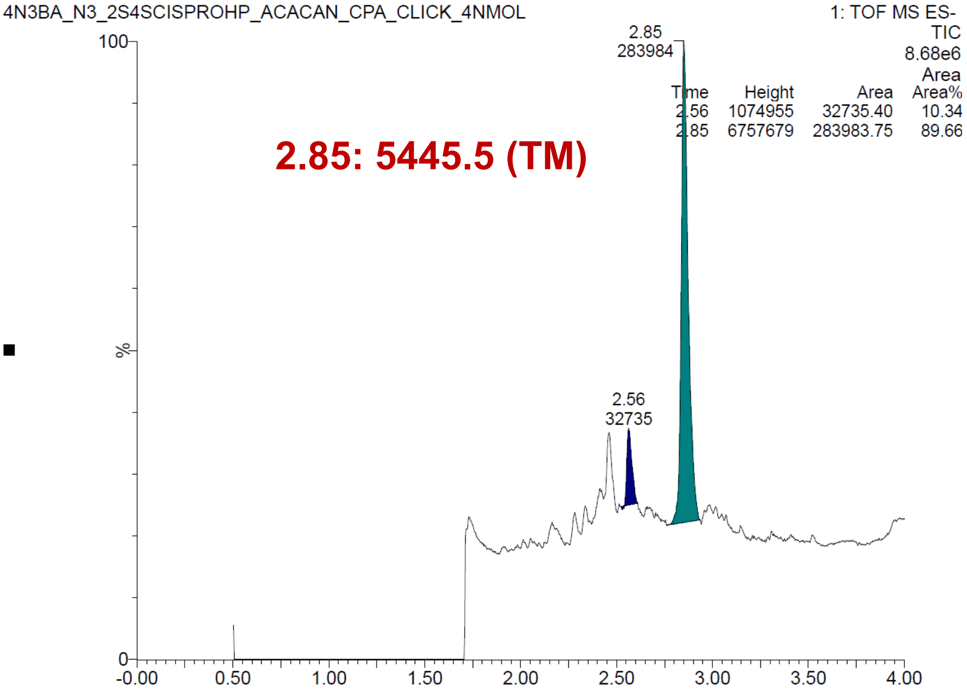

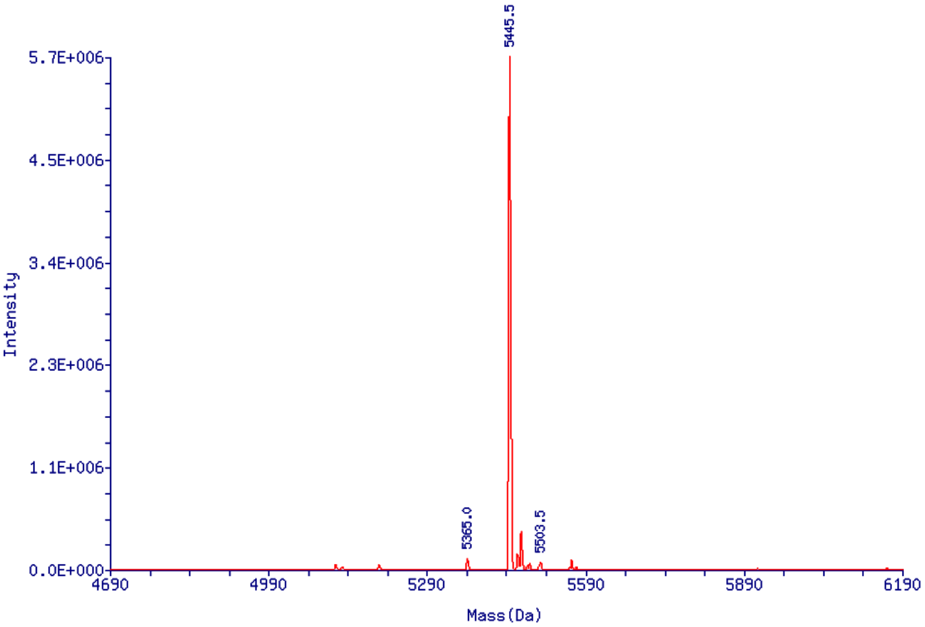

14f-TM

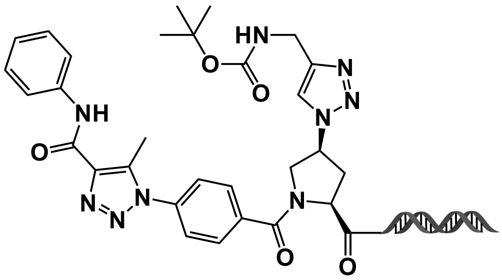

Conversion (Product%) = 92%
Base Peak Mass (Da): 5534.5

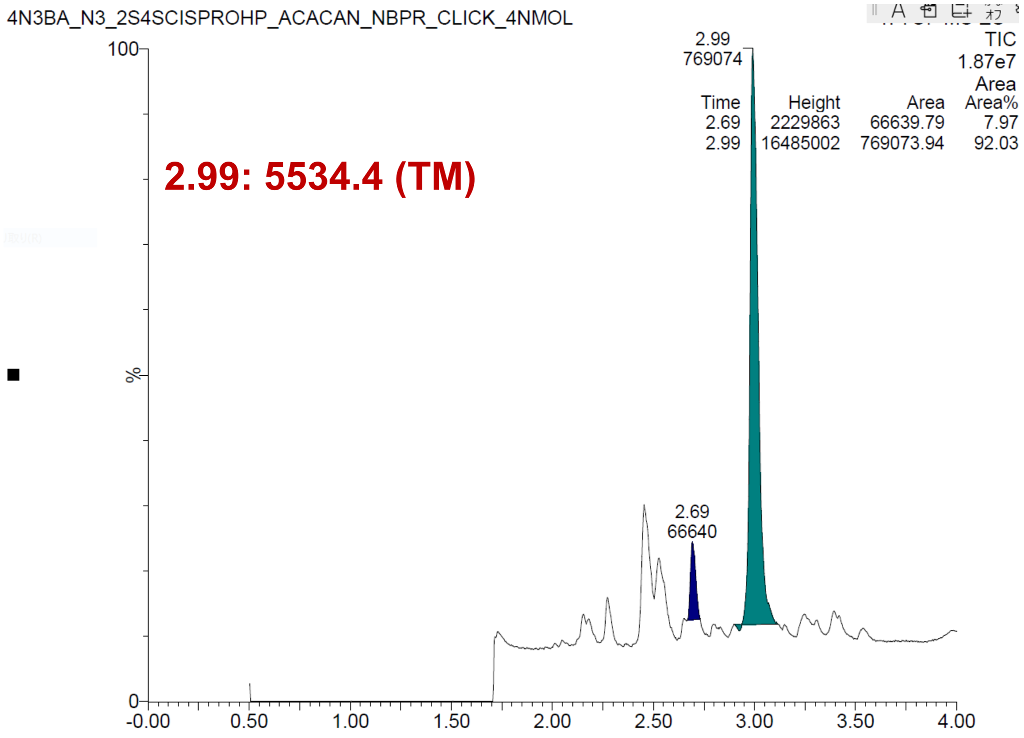

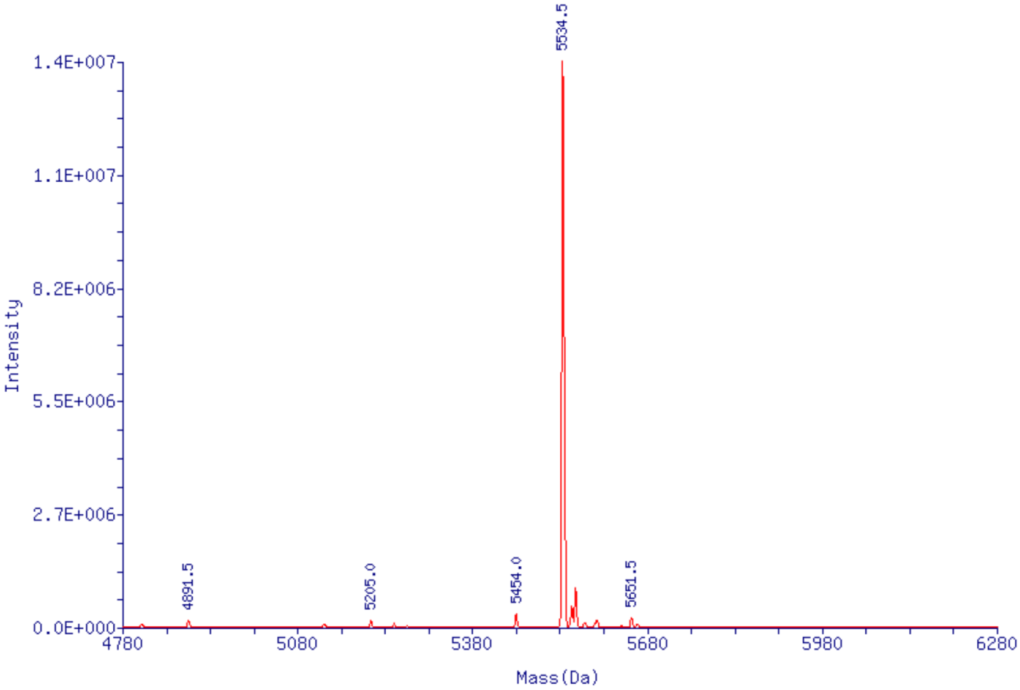

15a-TM

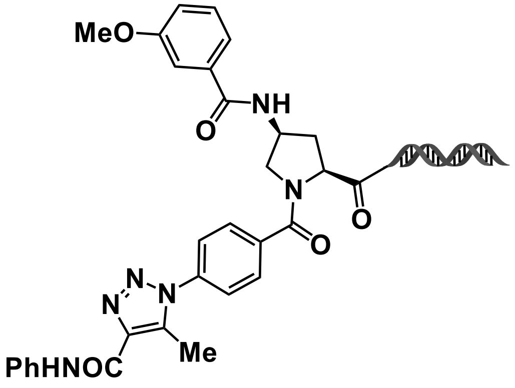

Conversion (Product%) = 55%
Base Peak Mass (Da): 5487.4

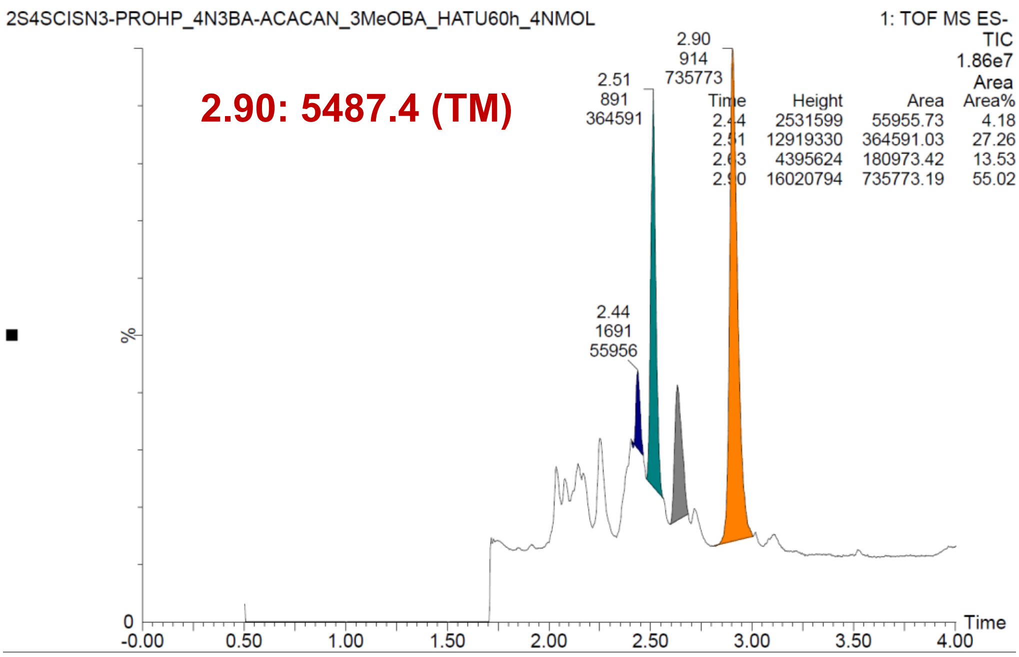

Time	Height	Area	Area%
2.44	2531599	55955.73	4.18
2.51	12919330	364591.03	27.26
2.63	4395624	180973.42	13.53
2.90	16020794	735773.19	55.02

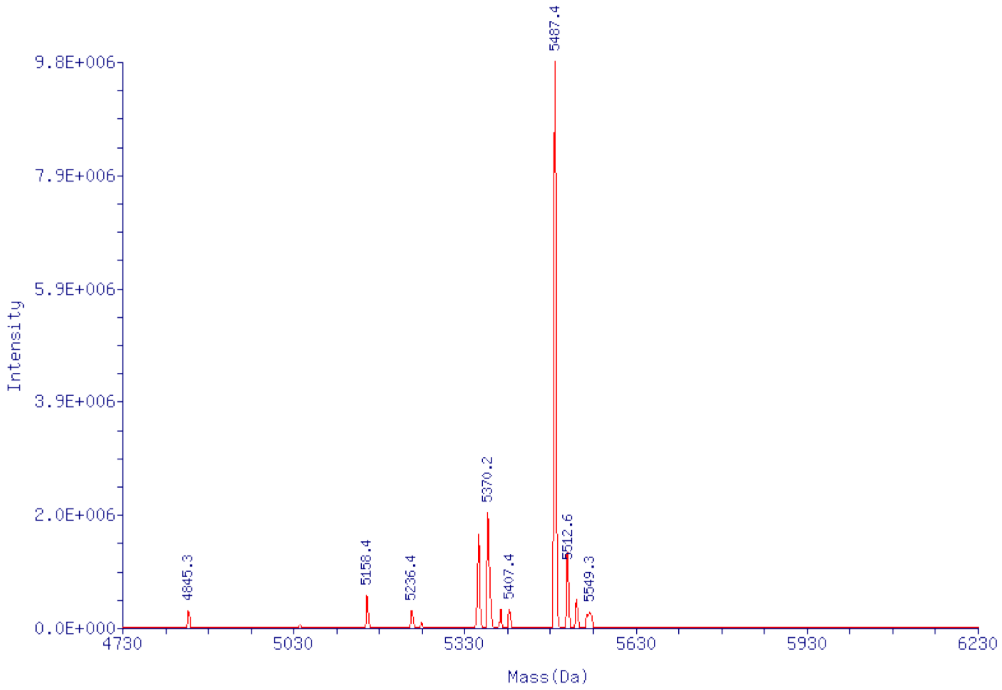

15b-TM

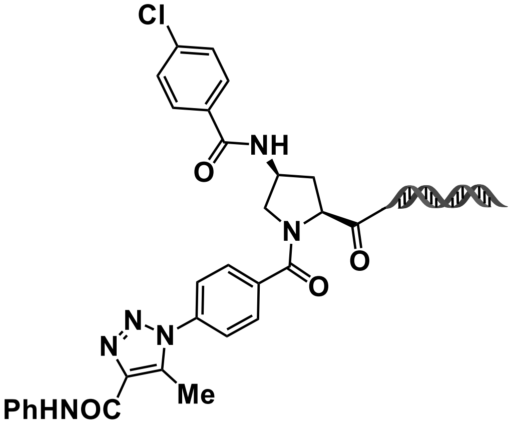

Conversion (Product%) = 83%
Base Peak Mass (Da): 5491.7

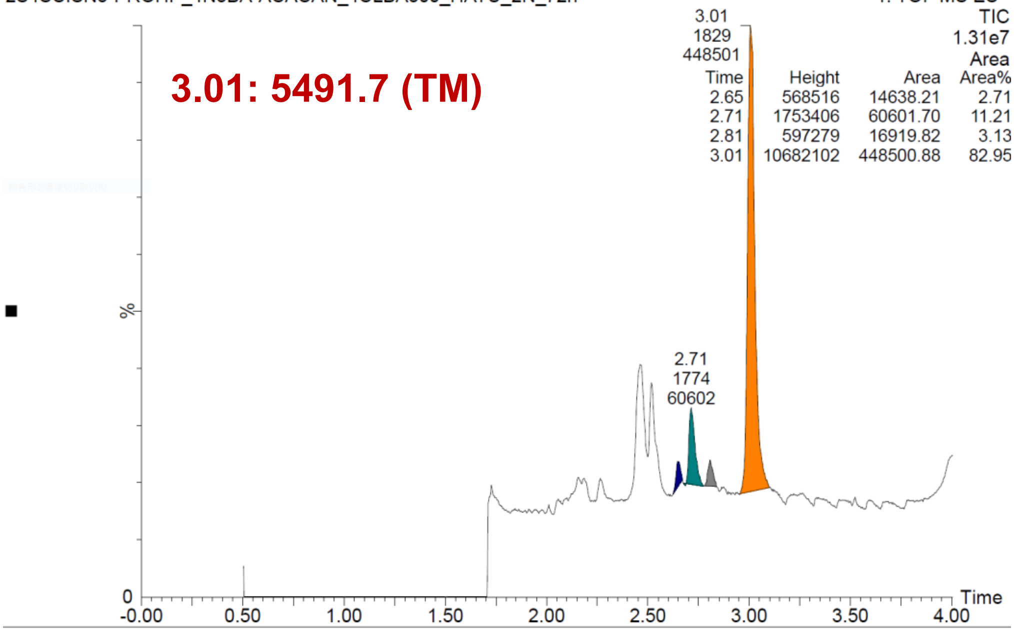

Time	Height	Area	Area%
2.65	568516	14638.21	2.71
2.71	1753406	60601.70	11.21
2.81	597279	16919.82	3.13
3.01	10682102	448500.88	82.95

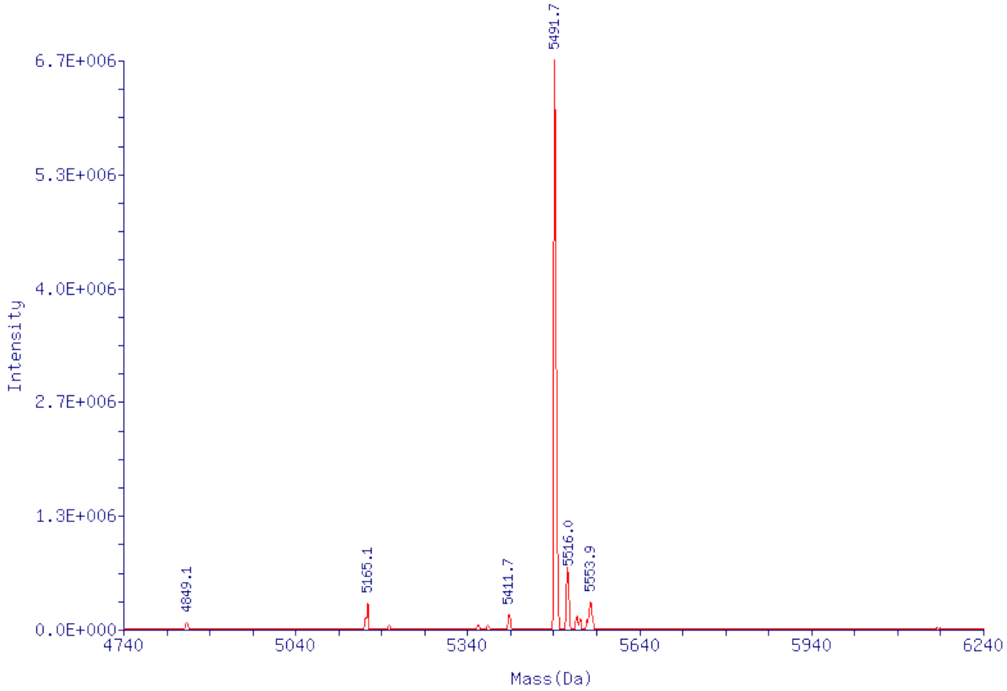

15c-TM

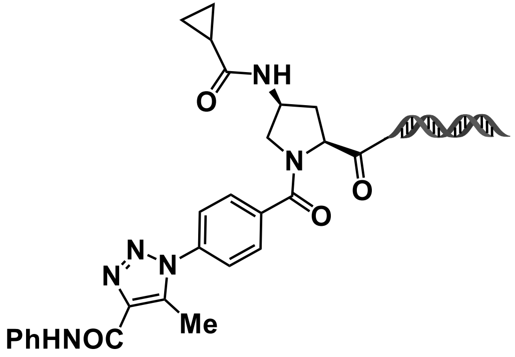

Conversion (Product%) = 97%
Base Peak Mass (Da): 5421.0

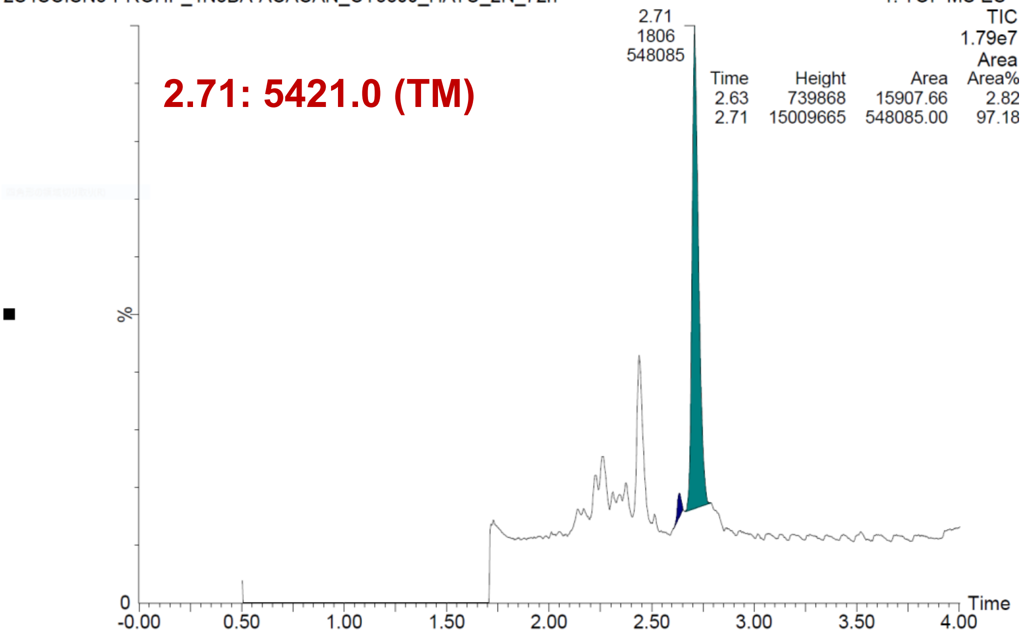

Time	Height	Area	Area%
2.63	739868	15907.66	2.82
2.71	15009665	548085.00	97.18

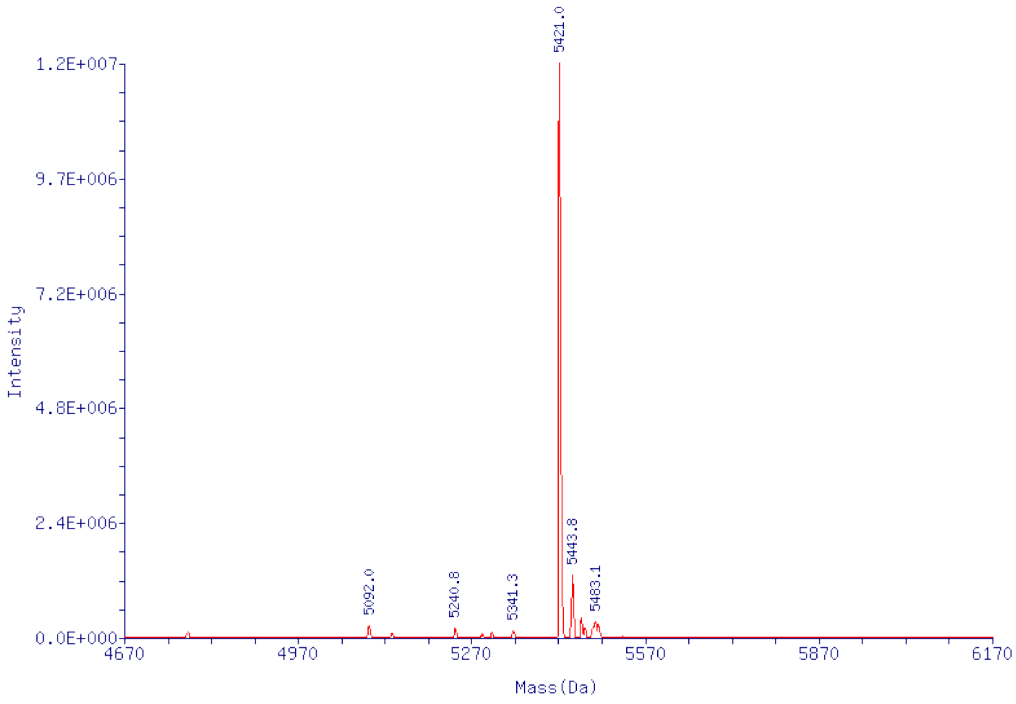

15d-TM

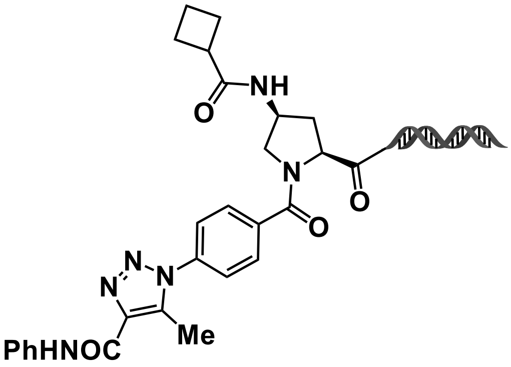

Conversion (Product%) = 73%
Base Peak Mass (Da): 5435.1

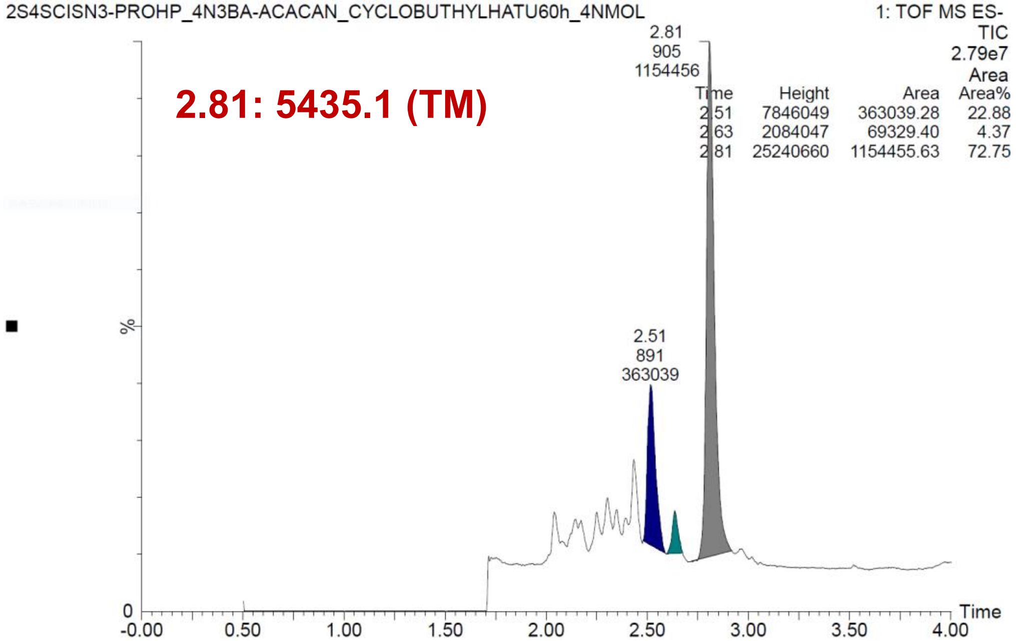

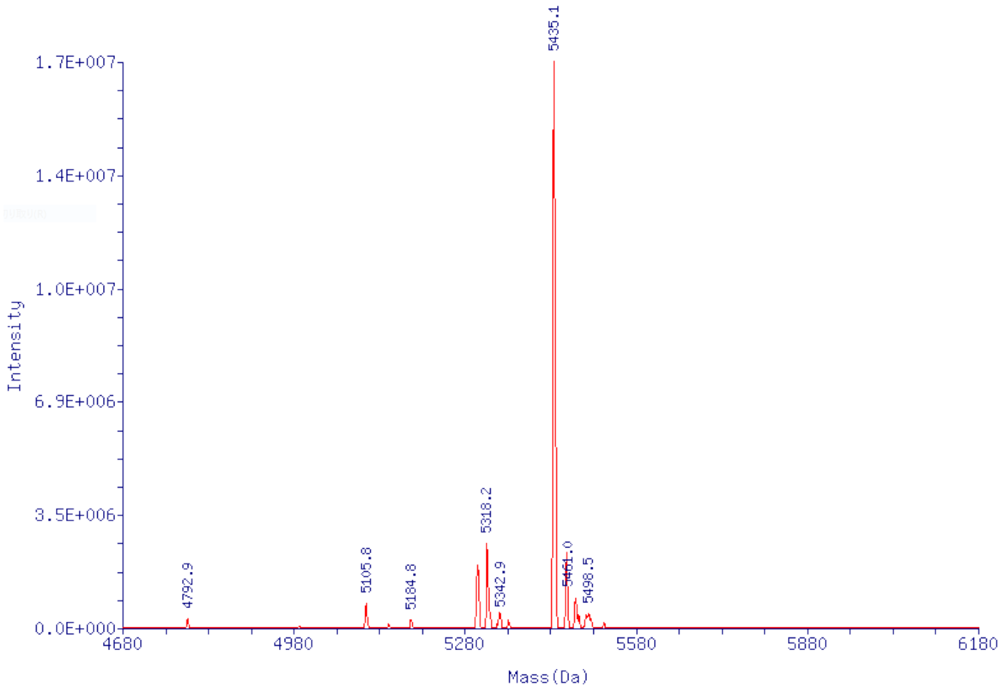

15e-TM

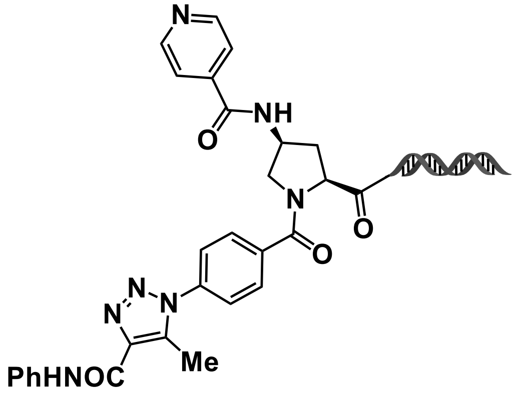

Conversion (Product%) = 45%
Base Peak Mass (Da): 5458.3

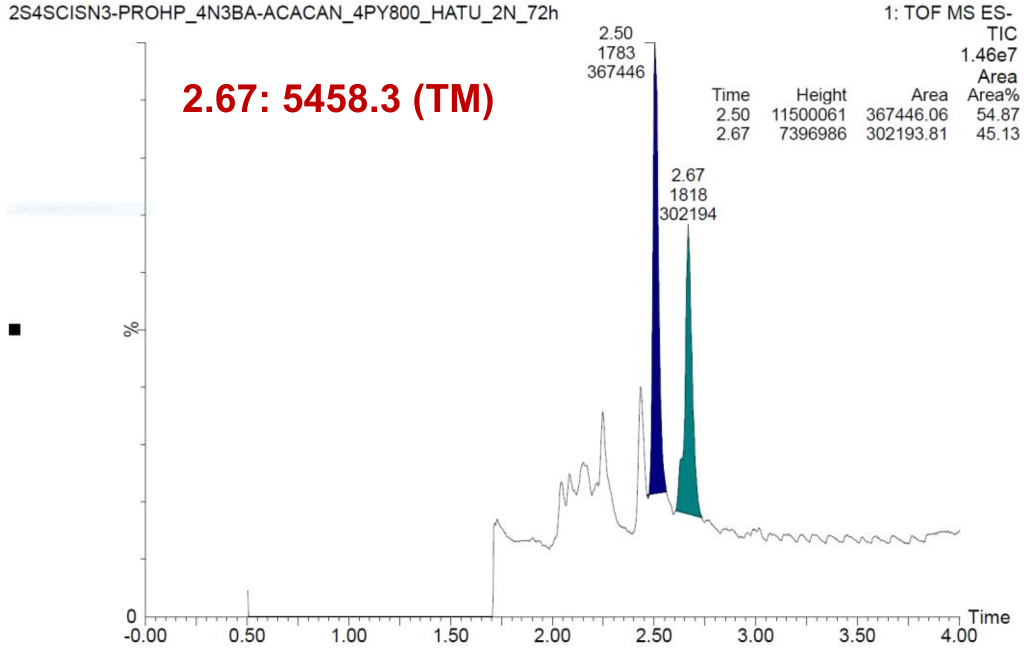

Time	Height	Area	Area%
2.50	11500061	367446.06	54.87
2.67	7396986	302193.81	45.13

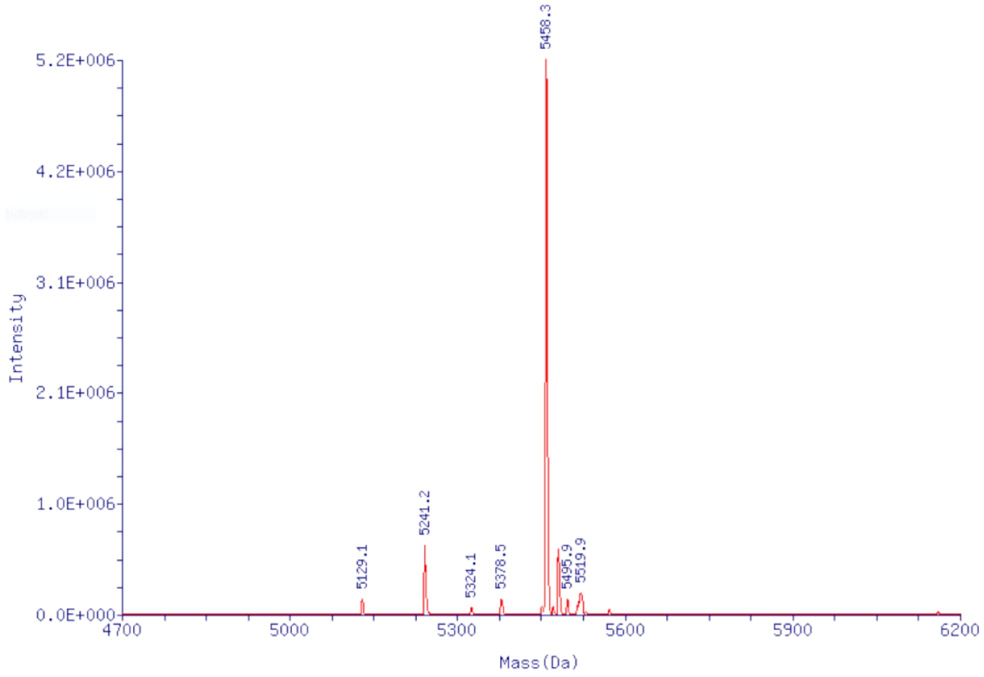

15g-TM

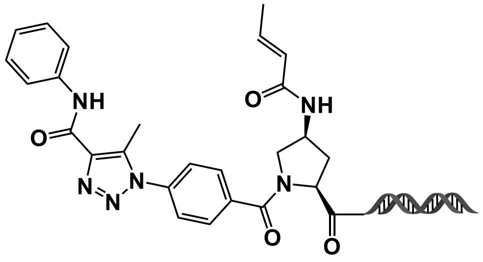

Conversion (Product%) = 97%
Base Peak Mass (Da): 5421.1

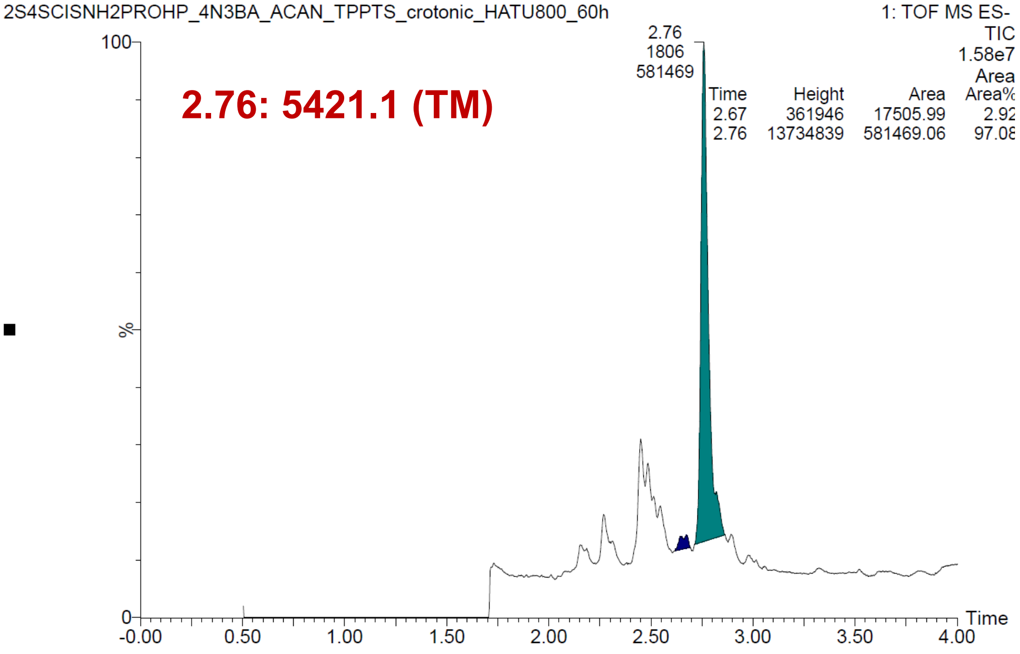

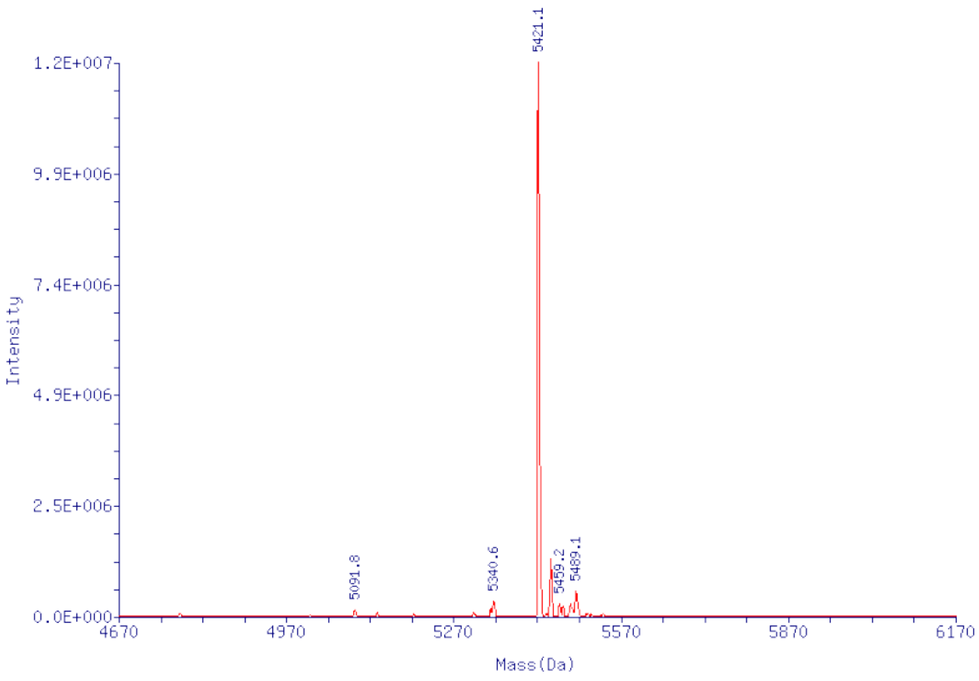

15h-TM

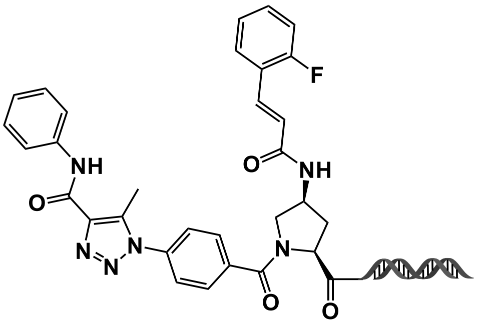

Conversion (Product%) = 88%
Base Peak Mass (Da): 5501.3

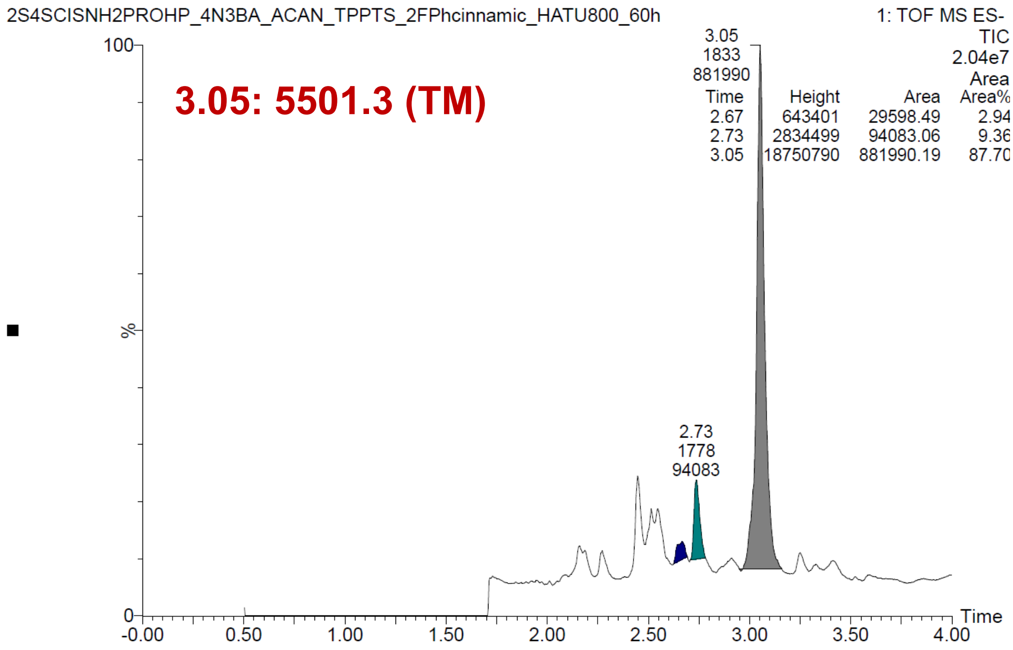

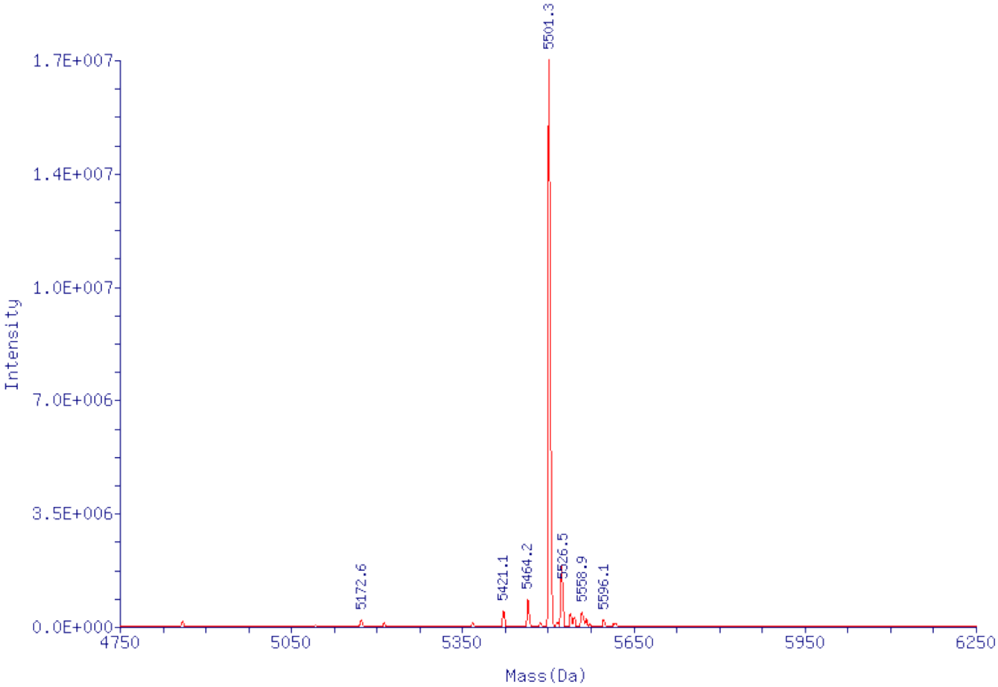

Validation of the Practical Utility of on-DNA DAP via Mock DNA-Encoded Pool Synthesis

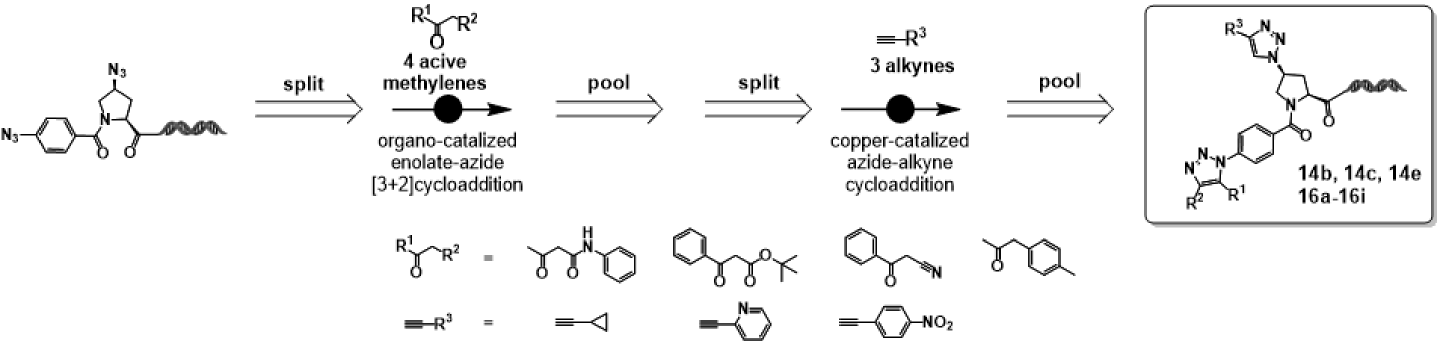

cpd.	R^1	R^2	R^3	Retention Time (min.)	MS. (calcd/found)
14e	Me	CONHPh	CYP	2.85	5445.1 / 5445.5
16a	Ph	CO_2tBu	CYP	3.33	5488.1 / 5487.6
16b	Ph	CN	CYP	2.78	5413.0 / 5413.4
16c	Me	4-Me-Ph	CYP	3.14	5416.1 / 5416.1
14c	Me	CONHPh	2-Py	2.84	5482.0 / 5482.2
16d	Ph	CO_2tBu	2-Py	3.32	5525.1 / 5525.4
16e	Ph	CN	2-Py	2.77	5450.0 / 5450.2
14b	Me	4-Me-Ph	2-Py	3.13	5453.0 / 5453.4
16f	Me	CONHPh	4-NO_2-Ph	2.86	5526.0 / 5526.3
16g	Ph	CO_2tBu	4-NO_2-Ph	3.36	5569.1 / 5569.4
16h	Ph	CN	4-NO_2-Ph	2.8	5492.0 / 5494.4
16i	Me	4-Me-Ph	4-NO_2-Ph	3.16	5481.0 / 5497.4

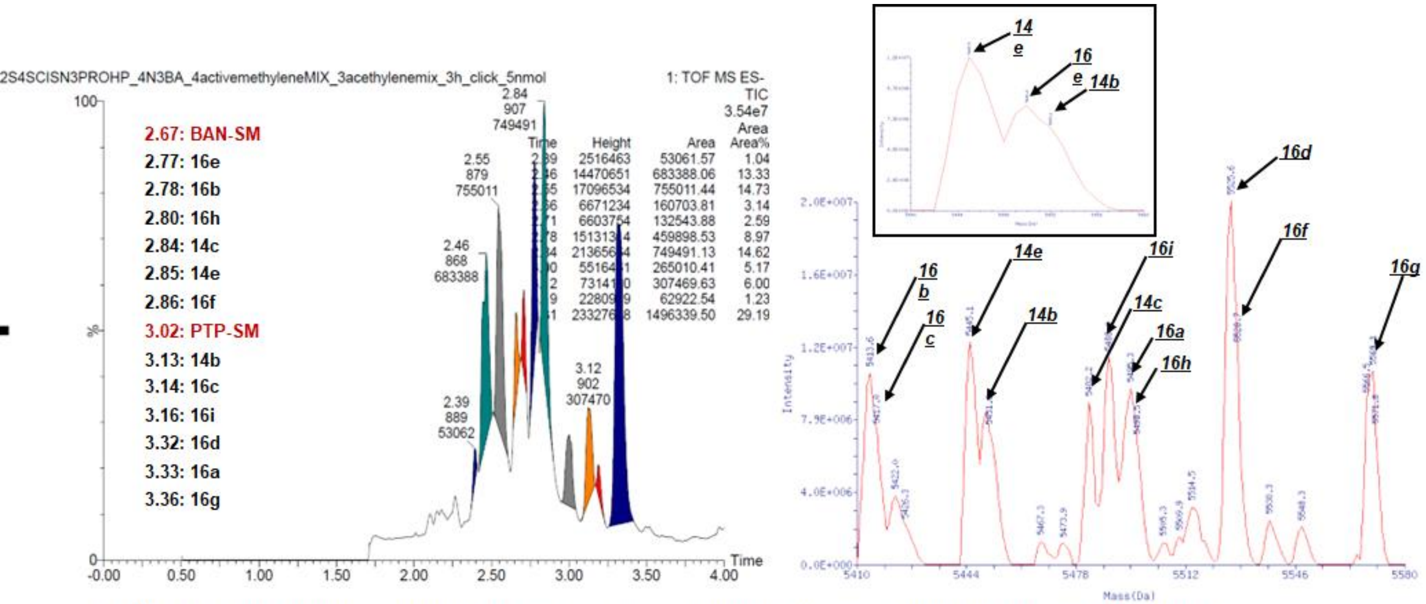

*BAN-SM: run 21 starting material. PTP-SM: run 22 starting material. The presence of both BAN-SM and PTP-SM clearly indicated that the reaction did not complete, and the optimization of the reaction conditions should be needed for future library construction.

Assessment of DNA Damage During the Full-Length on-DNA Synthesis of DAP Compounds

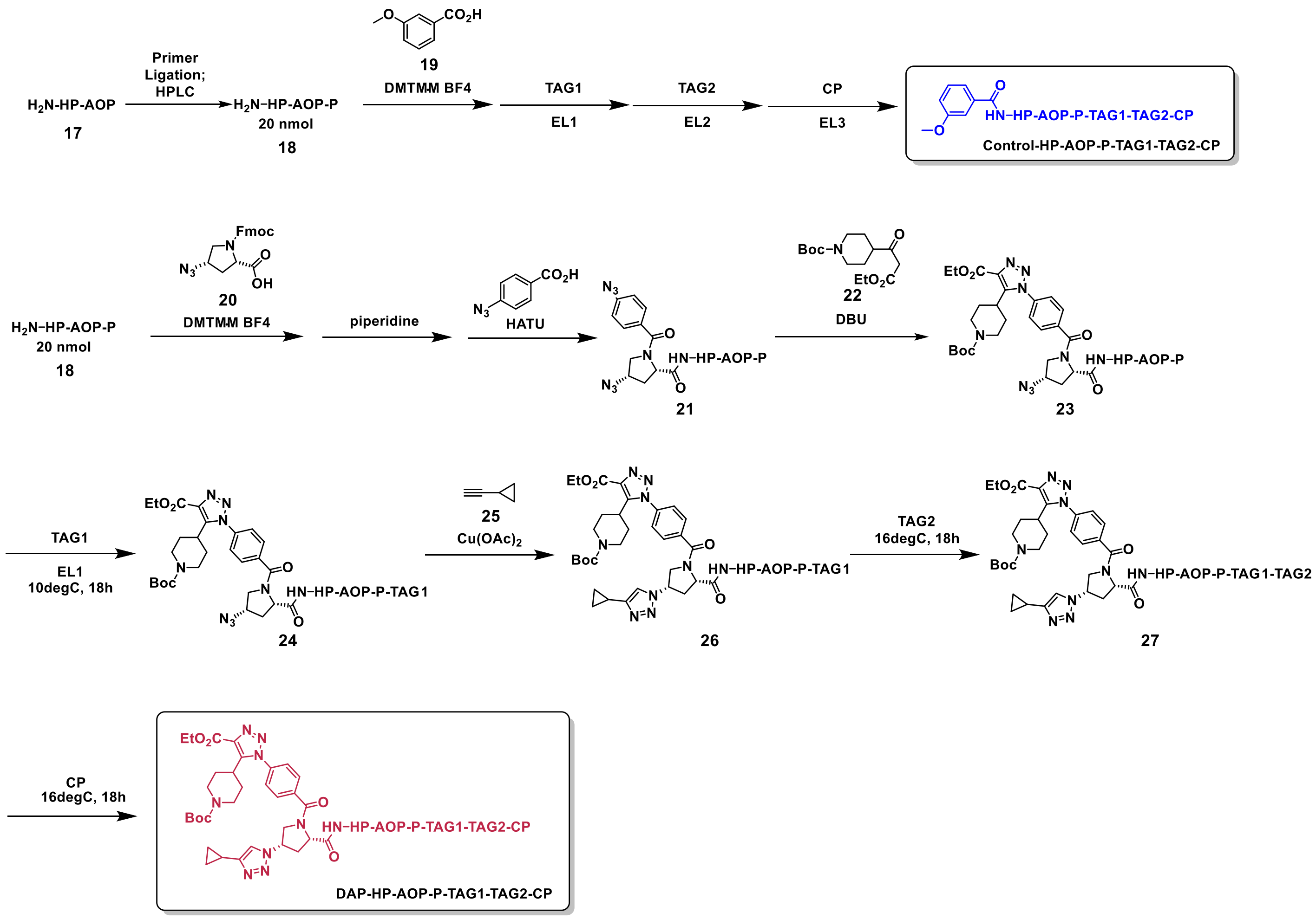

Control-HP-AOP-P-TAG1-TAG2-CP

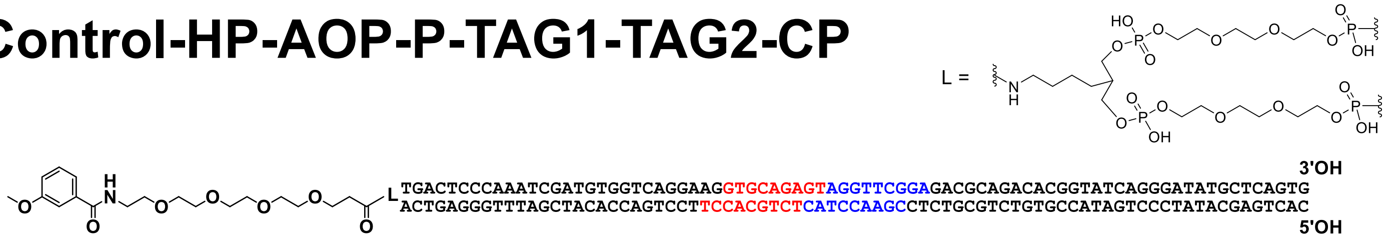

Calcud MS : 49849.4

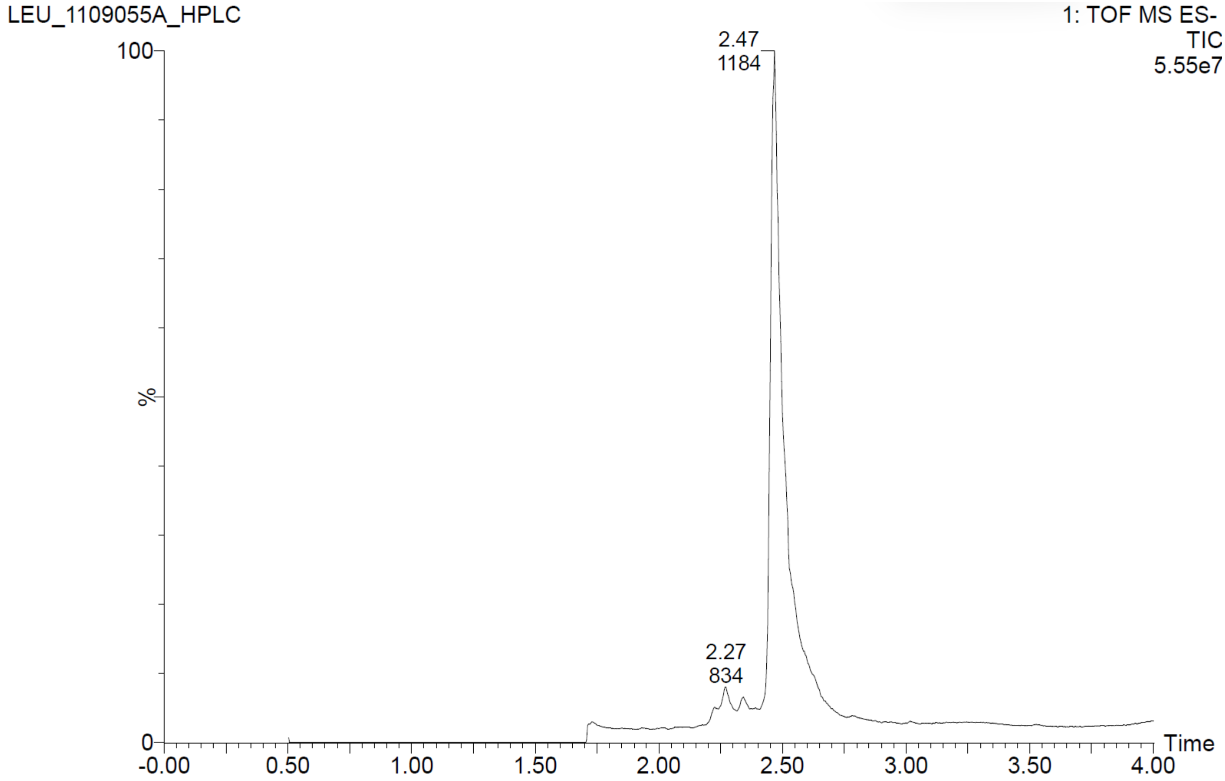

Deconvolued MS : 49871.3 (TM+Na adduct)

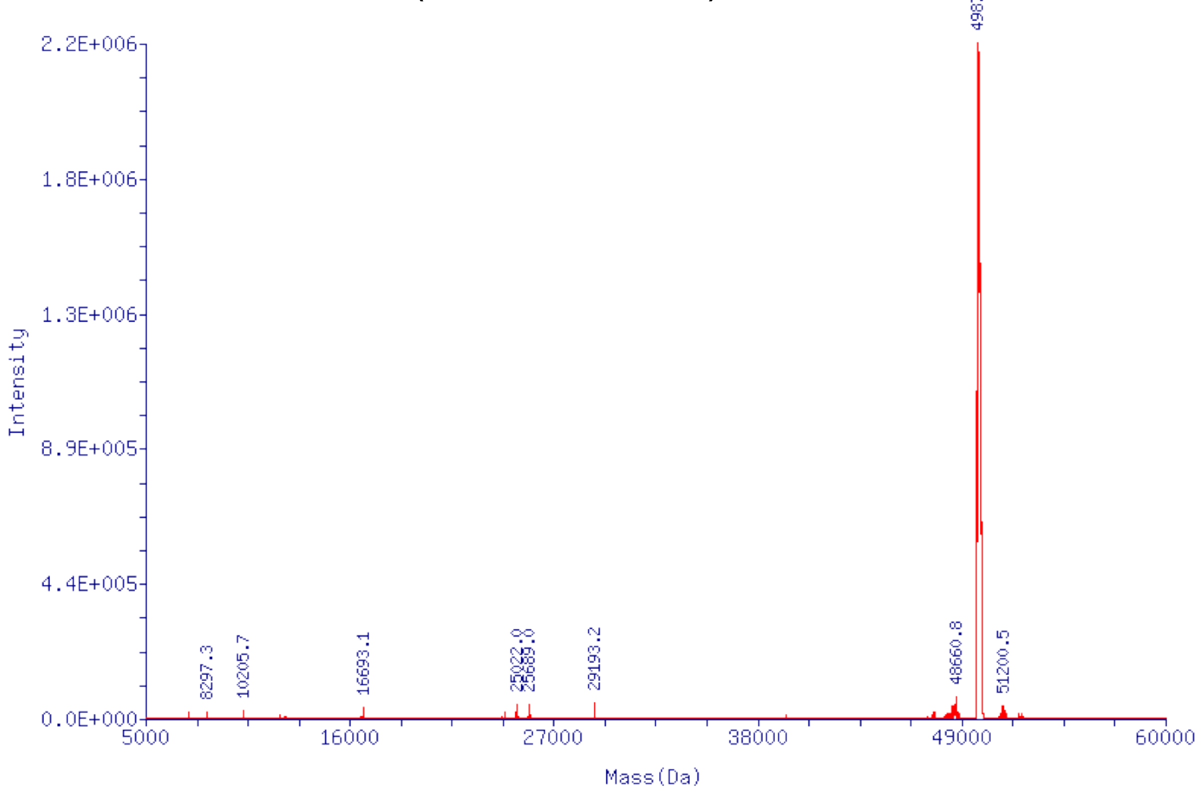

21

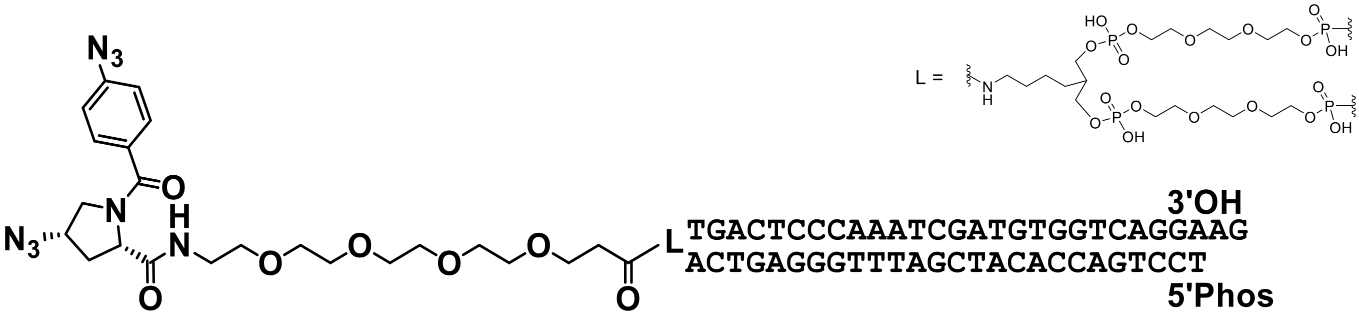

Calcud MS : 17889.8

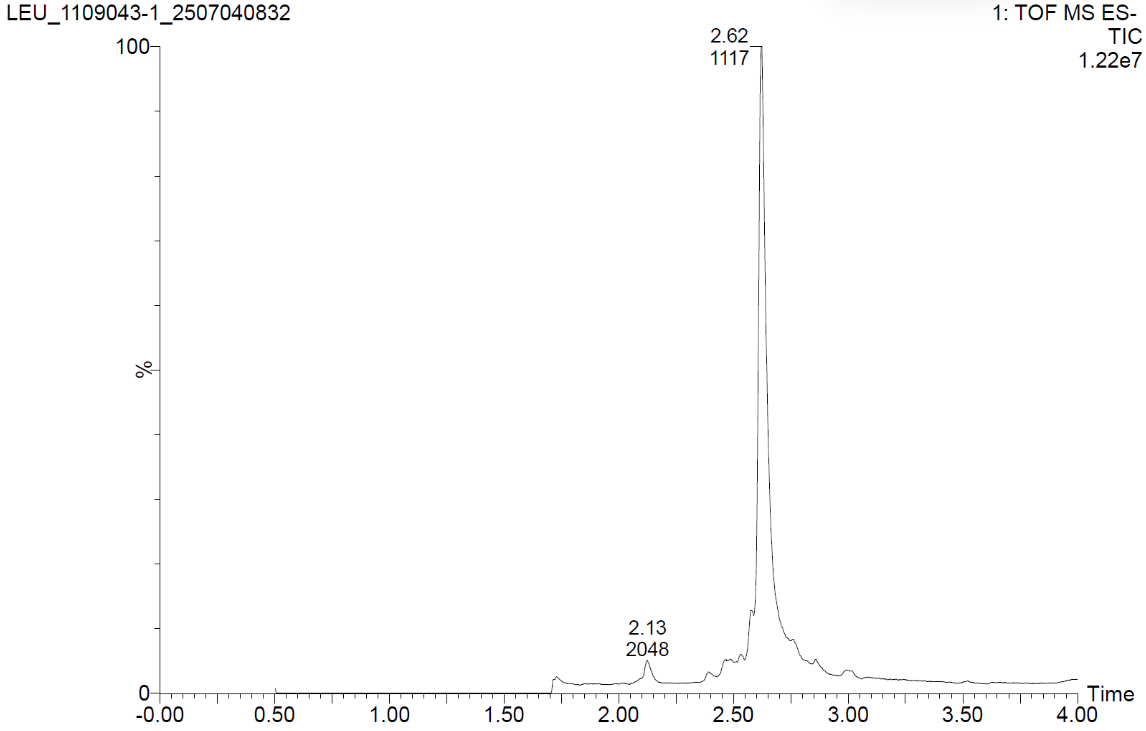

Deconvolued MS : 17889.6 (TM)

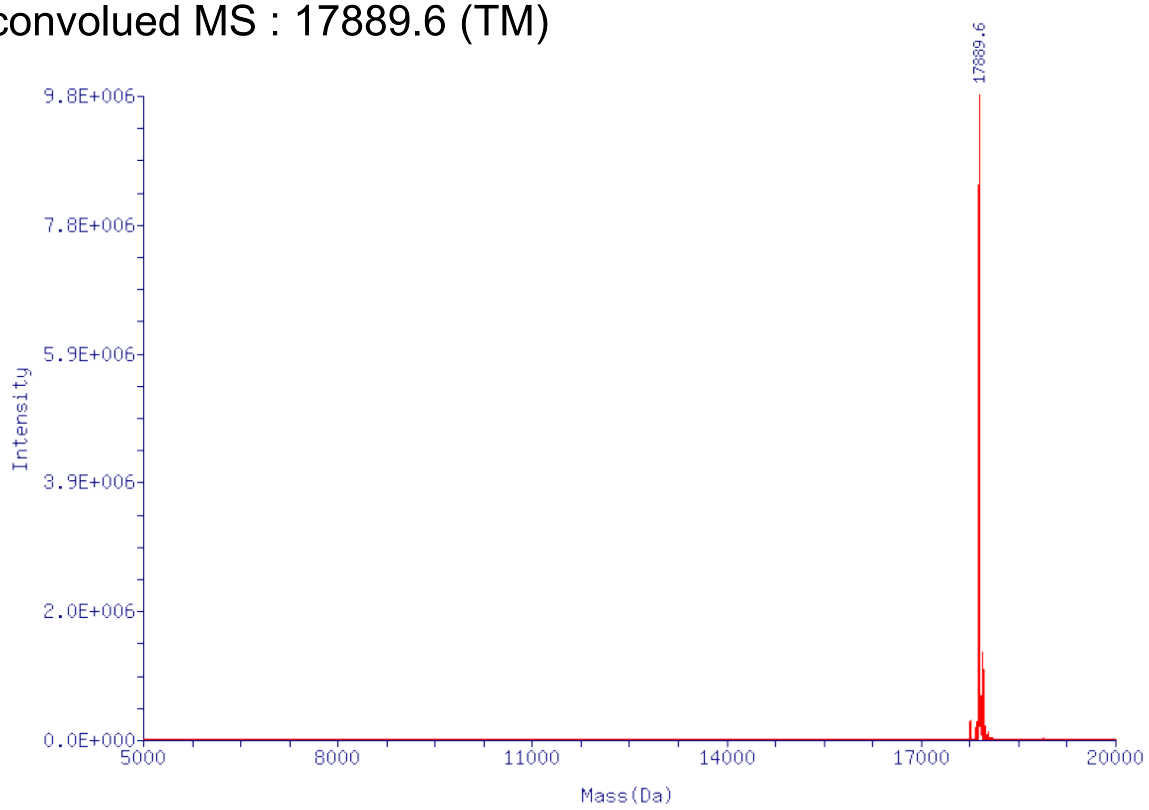

23

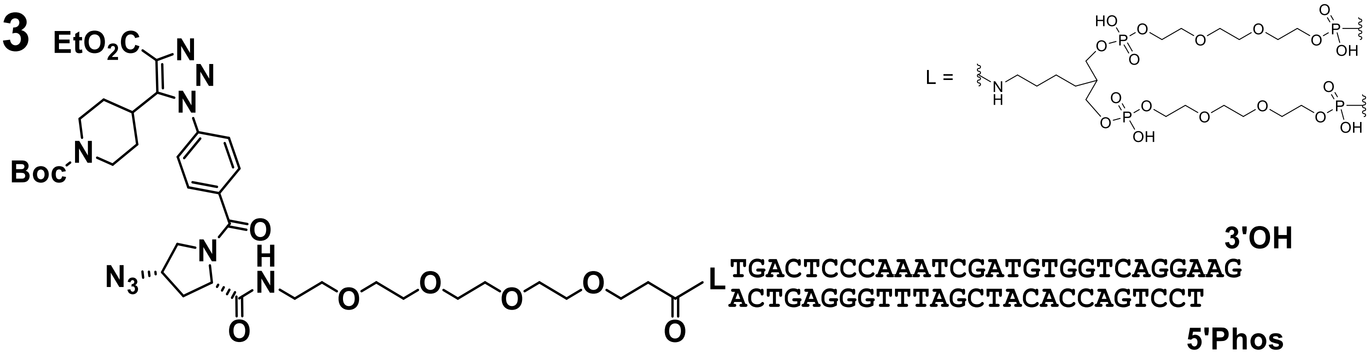

Calcud MS : 18171.1

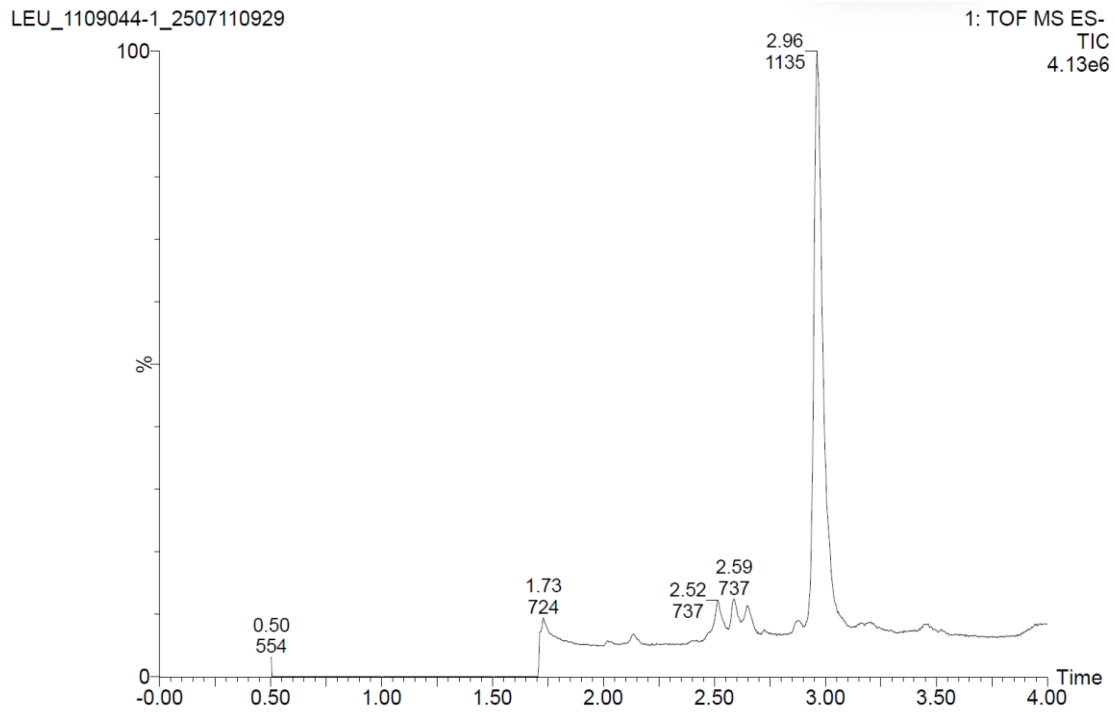

Deconvolued MS : 18171.5 (TM)

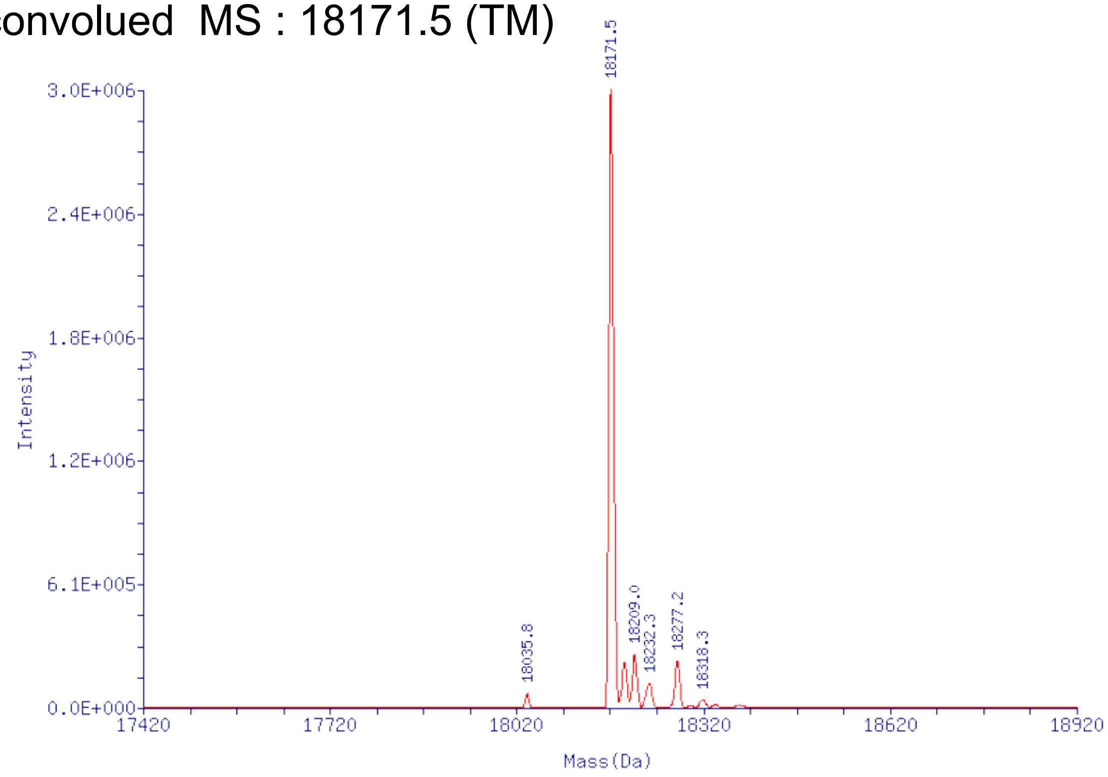

24

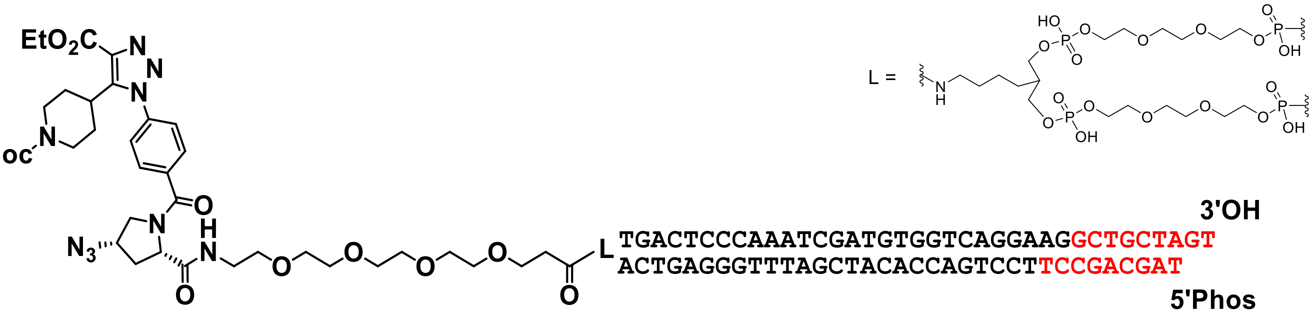

Calcud MS : 23723.7

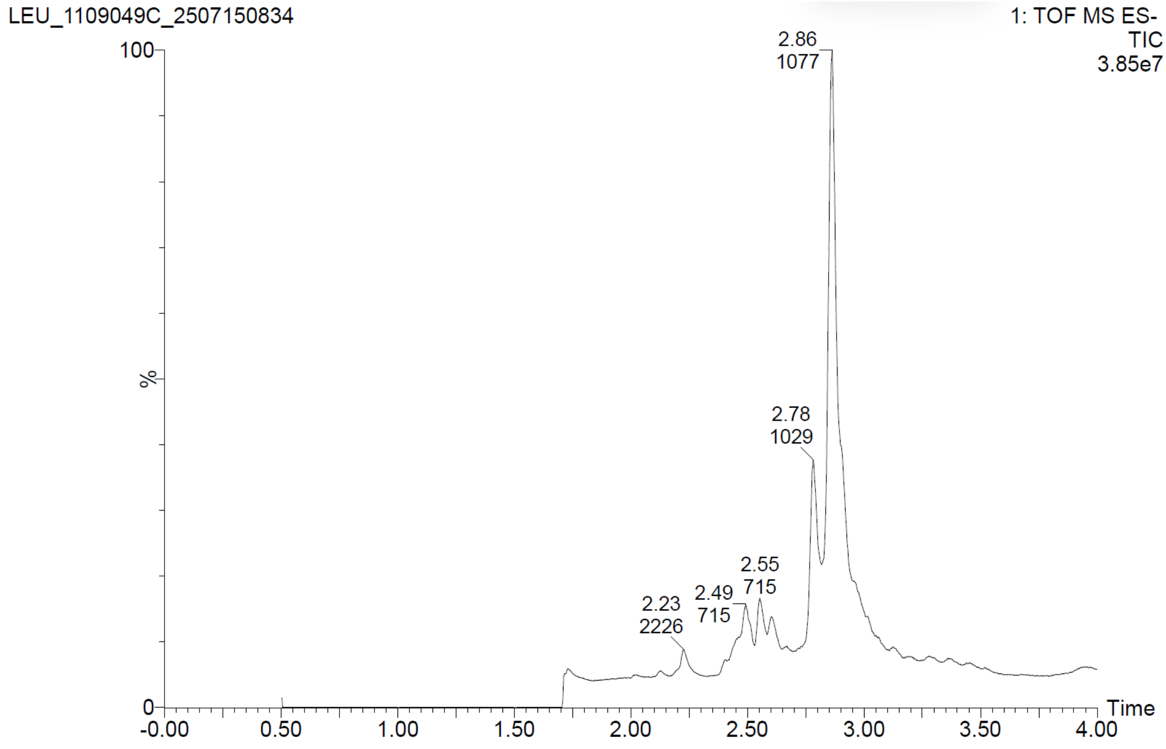

Deconvolued MS MS : 23724.2 (TM)

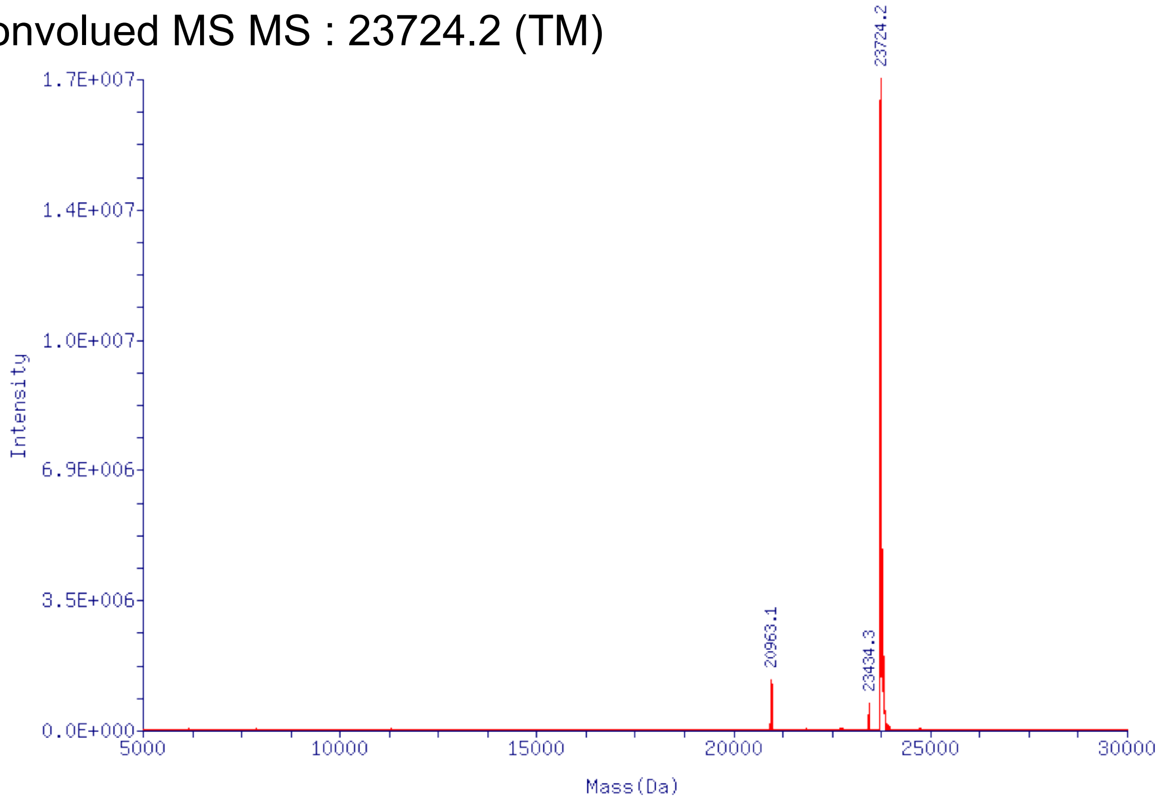

26

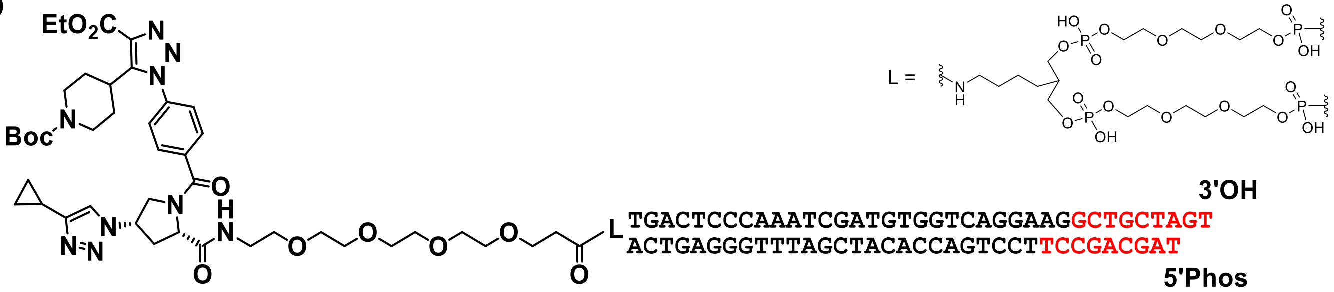

Calcud MS : 23789.8

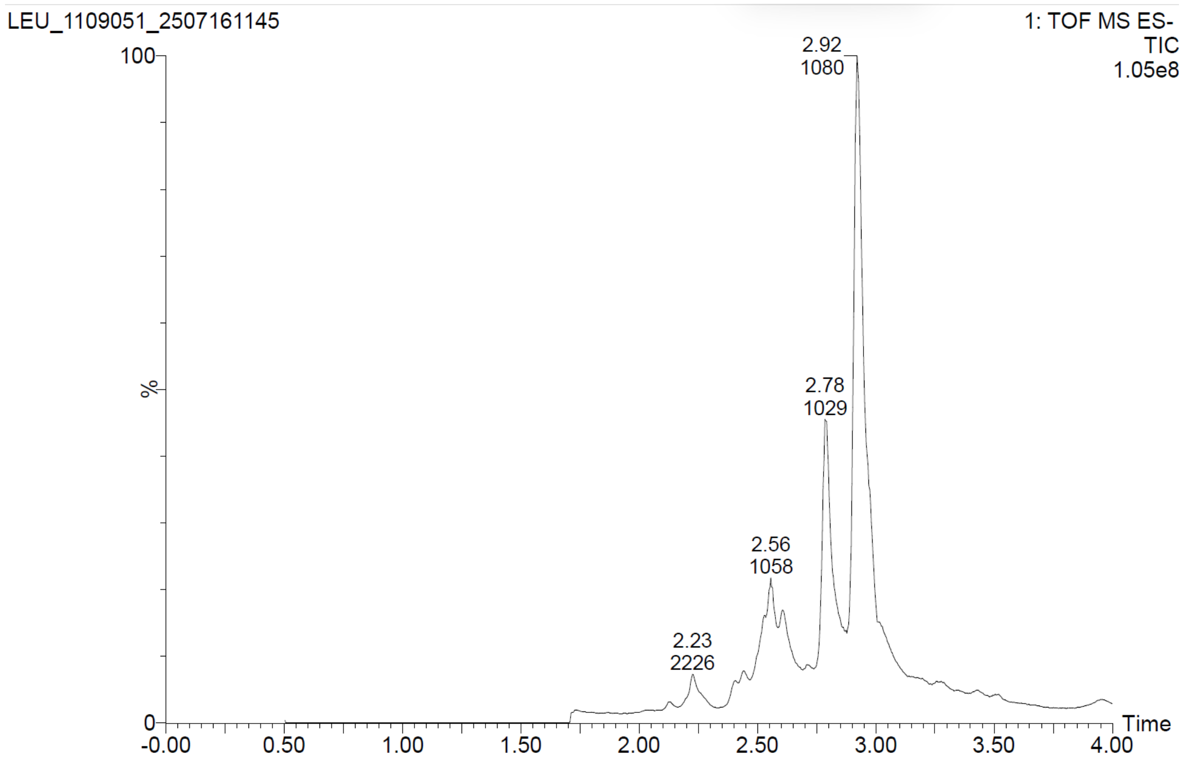

Deconvolued MS d MS : 23790.2 (TM)

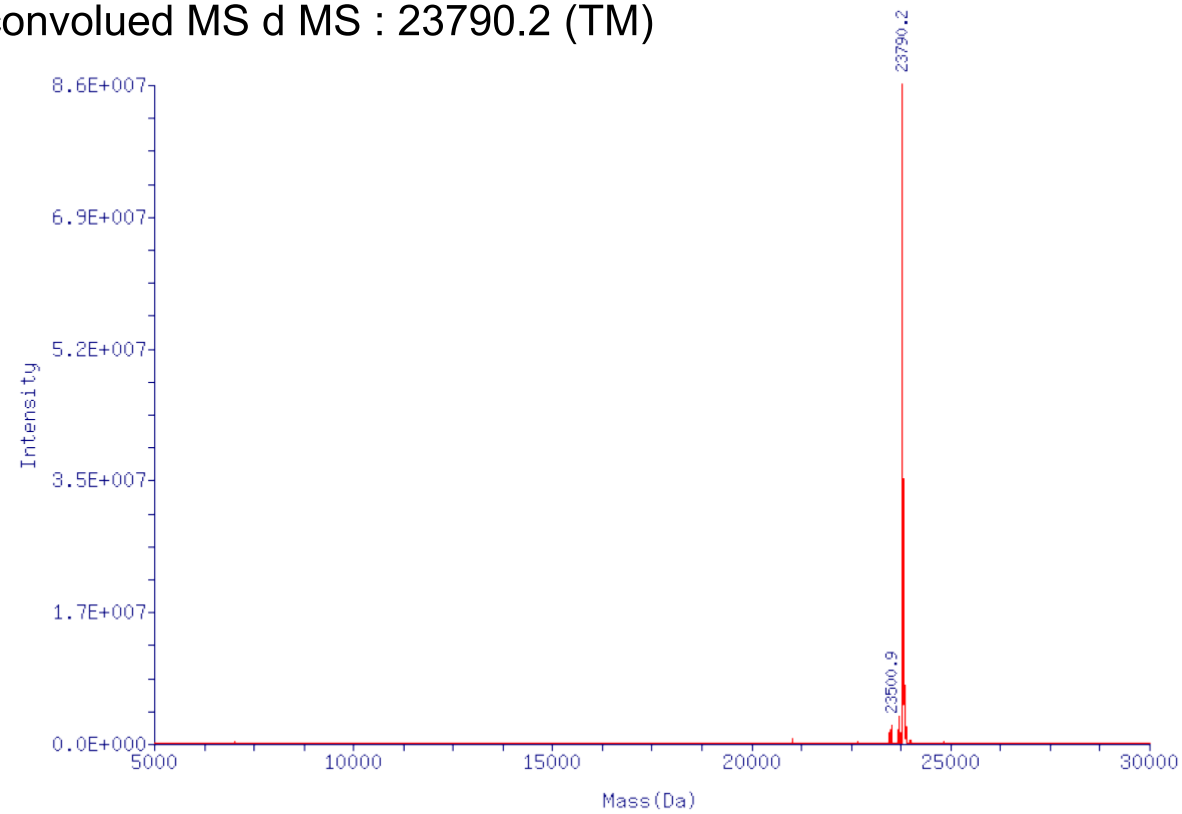

27

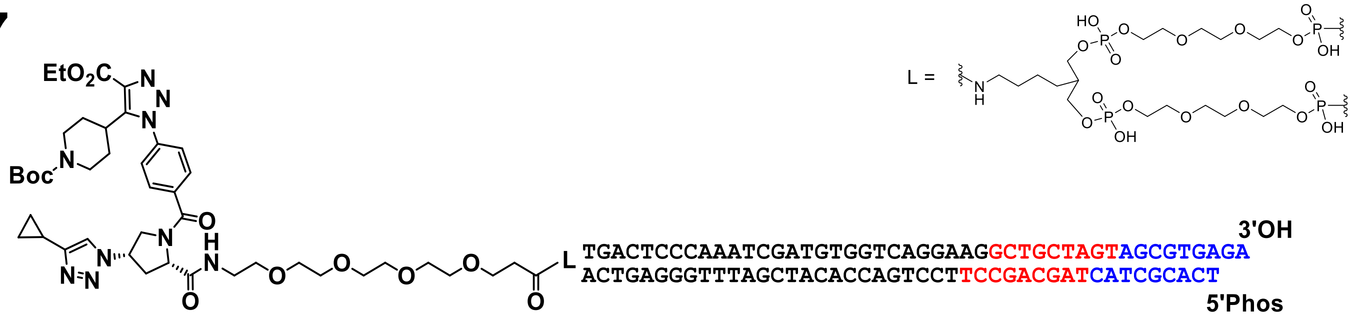

Calcud MS : 29360.4

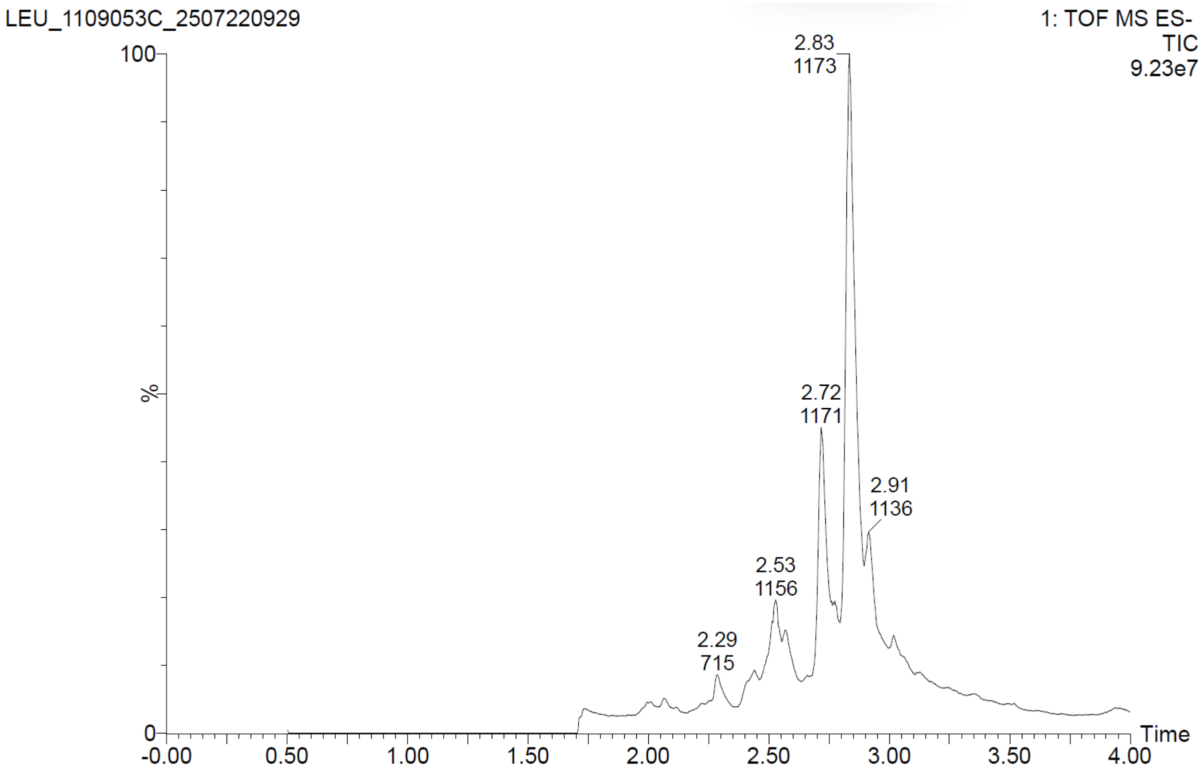

Deconvolued MS MS : 29360.9 (TM)

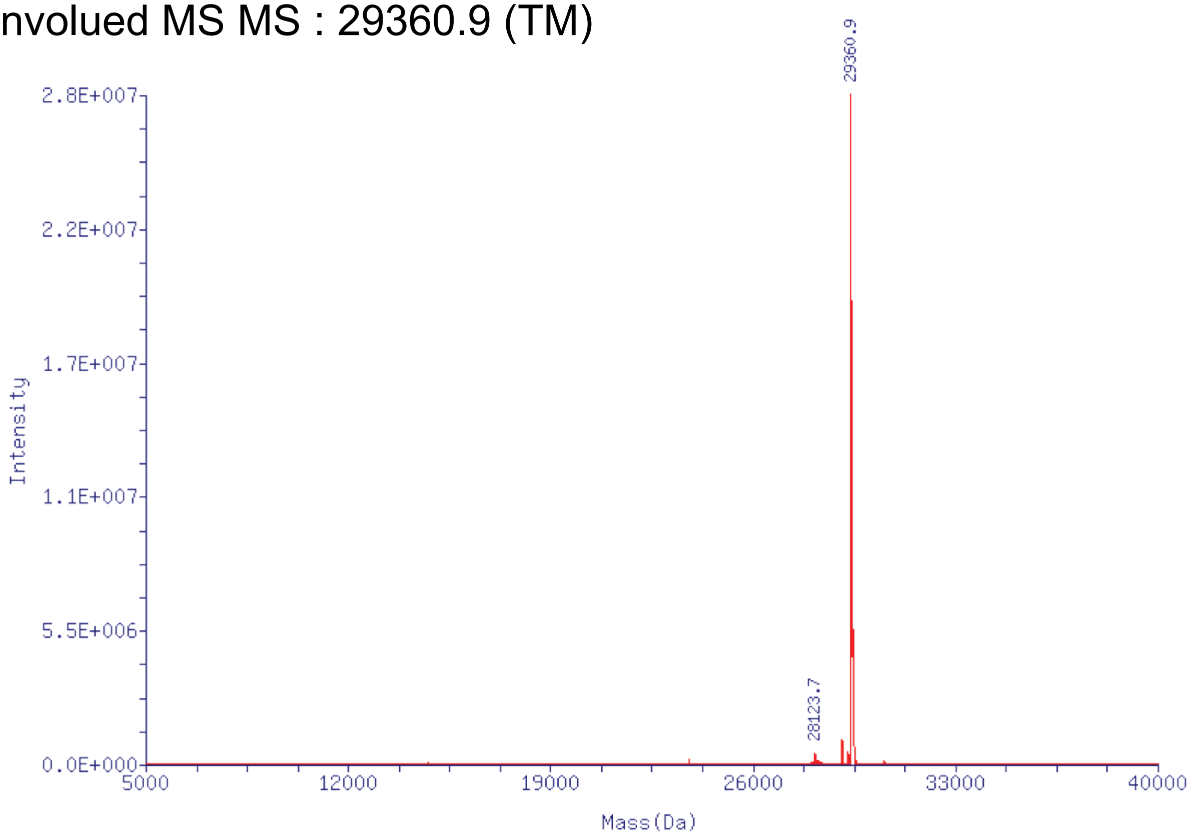

DAP-HP-AOP-P-TAG1-TAG2-CP

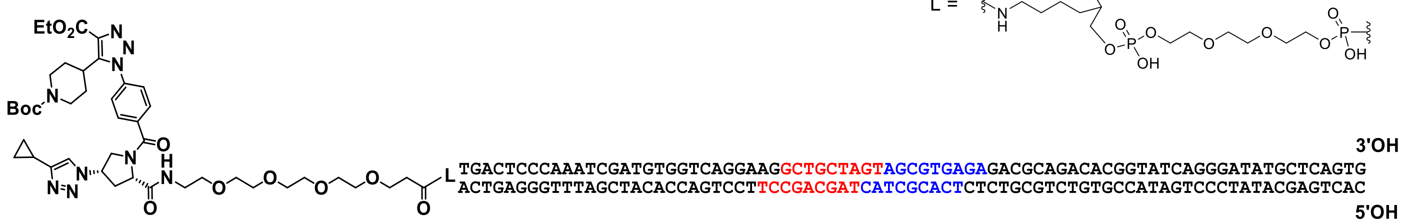

Calcud MS : 50345.9

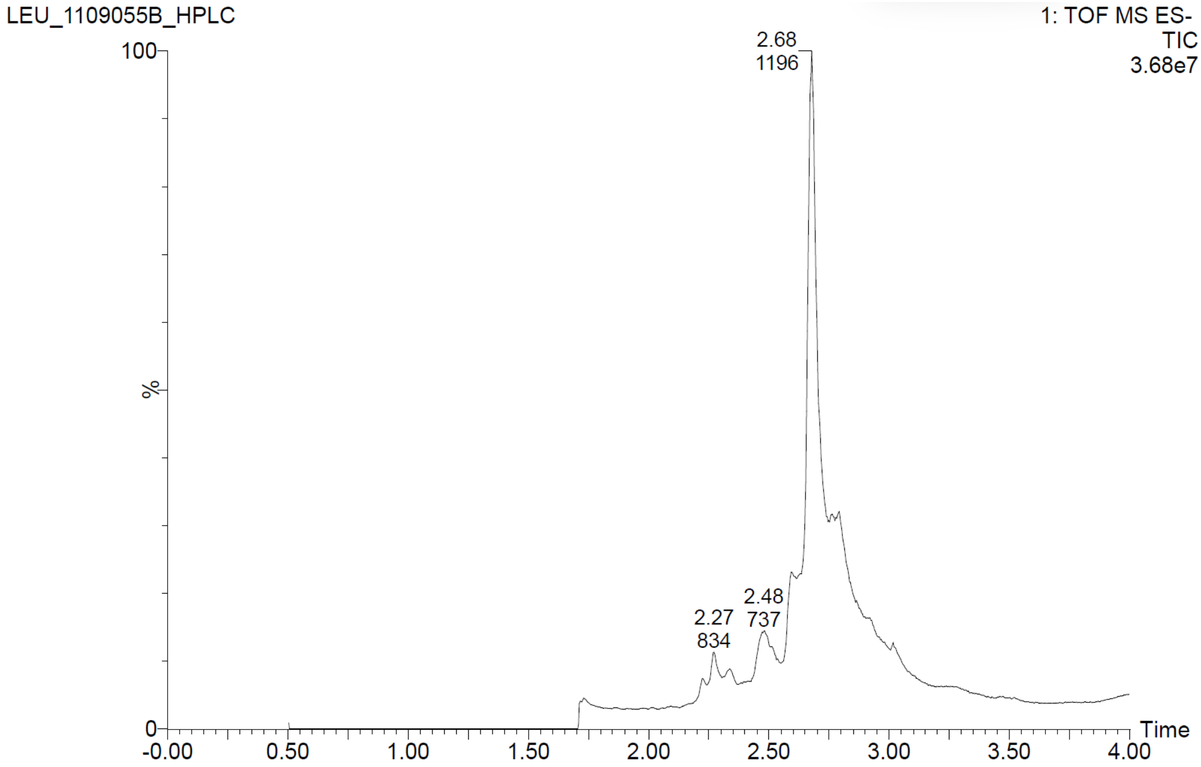

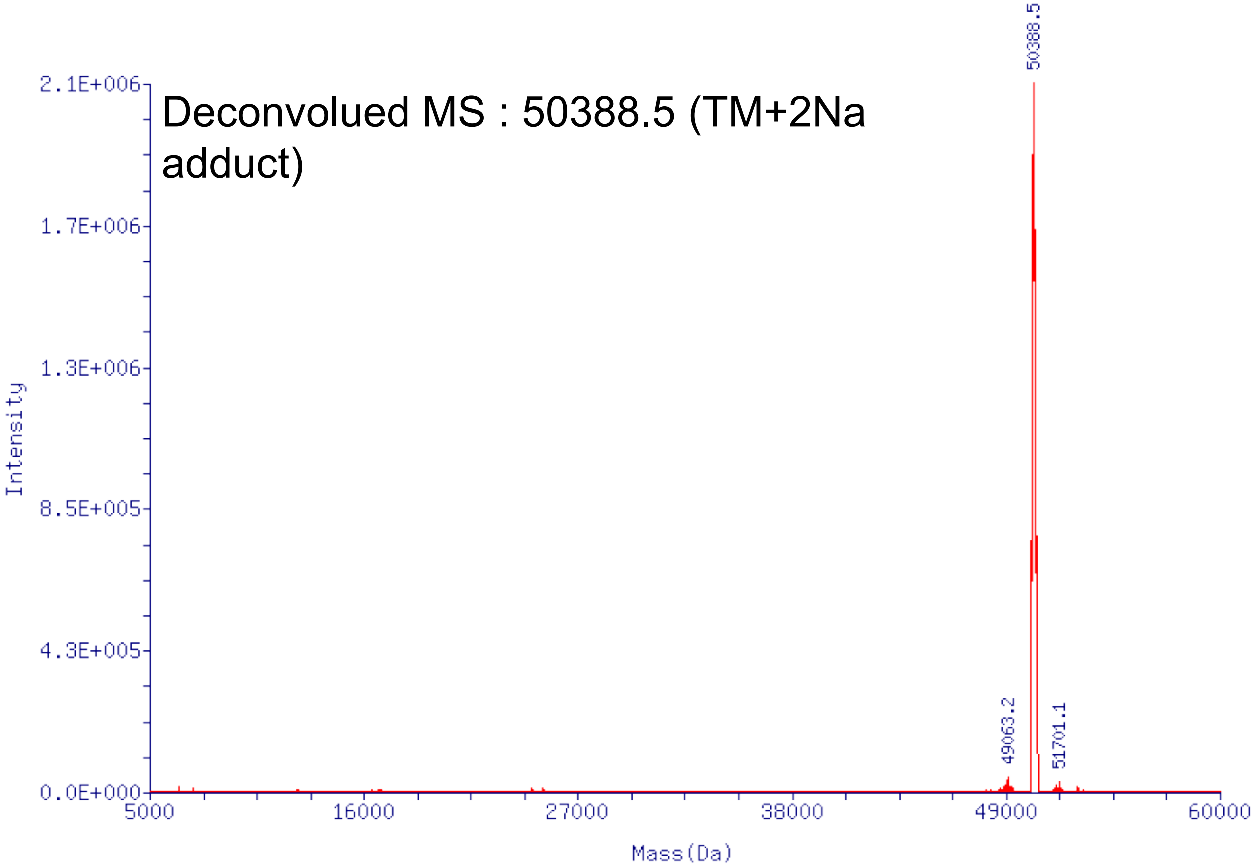

qPCR analysis of the Full-Length on-DNA Compounds

Experimental Conditions

Entry	Final DNA conc.
1	900
2	270
3	81
4	24.3
5	7.29
6	2.187
7	0.6561
8	water

※n=3

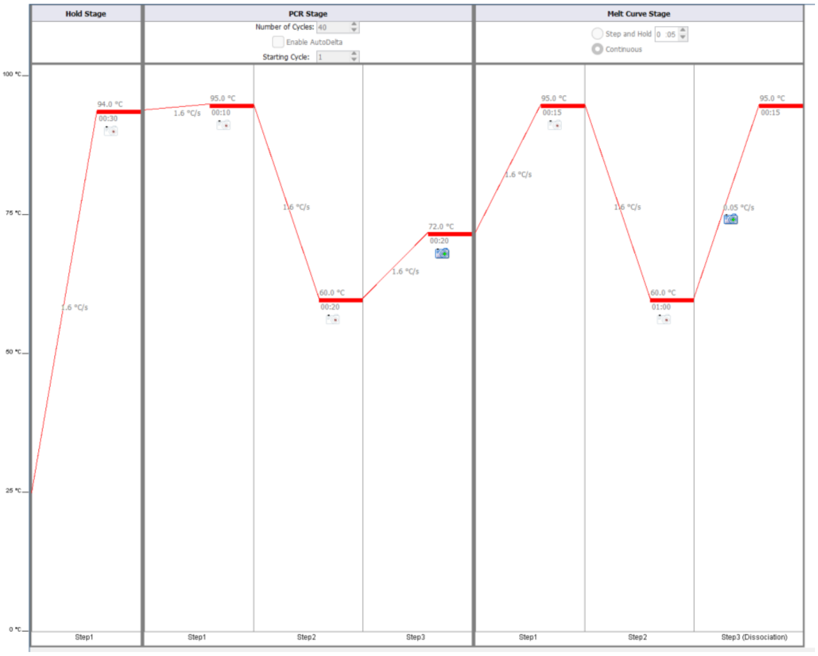

Each qPCR sample (10 µL)

- DNA: 1 µL
- TB Green Premix EX Taq II：5 µL
- Forward primer (50 µM)：0.08 µL
- Reverse primer (50 µM)：0.08 µL
- nuclease-free water：3.64 µL
- ROX Reference Dye II：0.2 µL

Melt Curve Plot (Control-HP-AOP-P-TAG1-TAG2-CP)

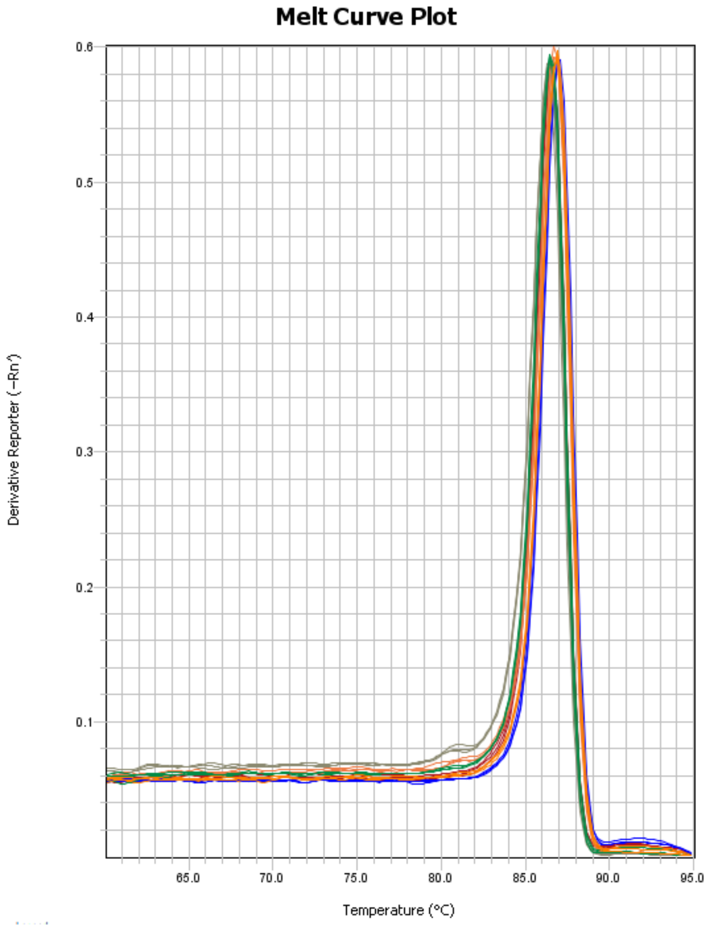

Result of Control-HP-AOP-P-TAG1-TAG2-CP

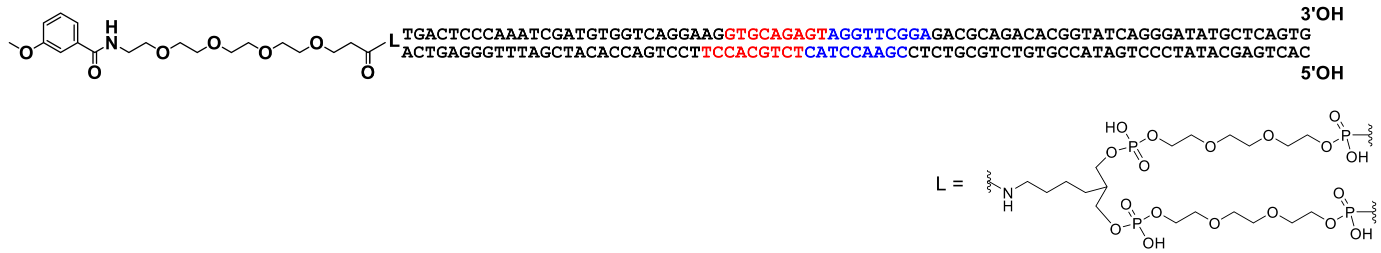

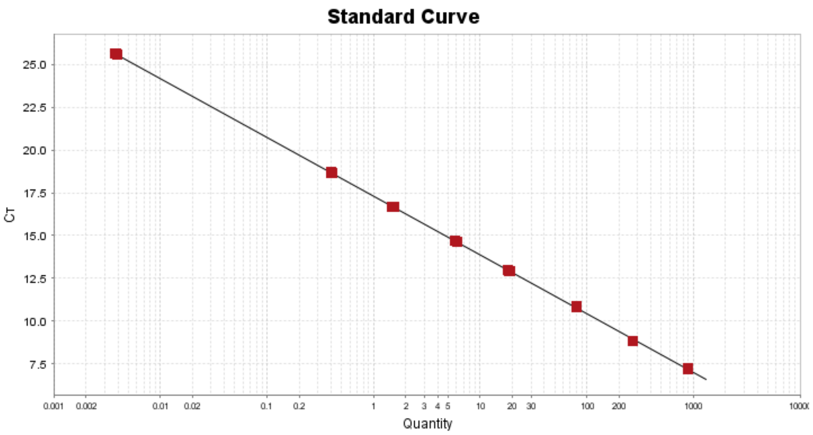

slope = -3.435, y-intercept = 17.315, R2 = 0.997, efficiency = 95.496%, Error = 0.074

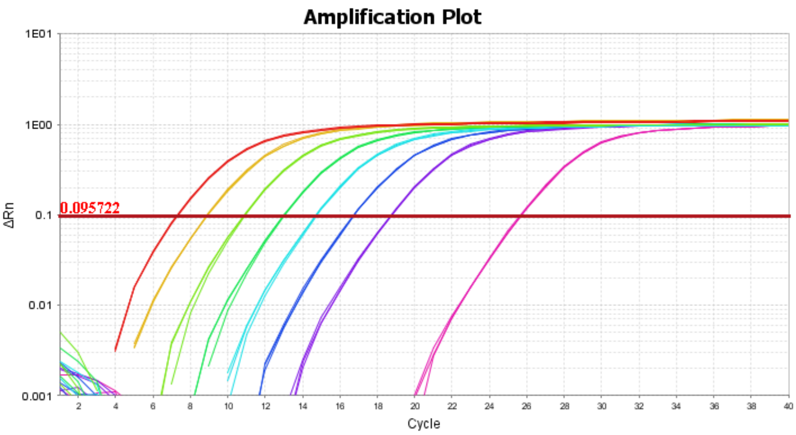

CONFIDENTIAL

Result of DAP-HP-AOP-P-TAG1-TAG2-CP

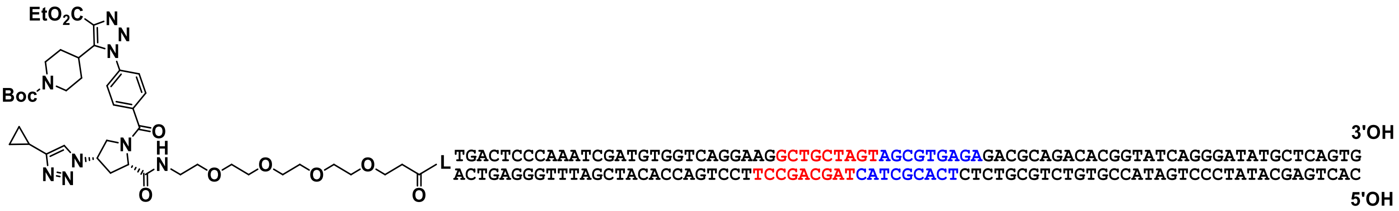

L =
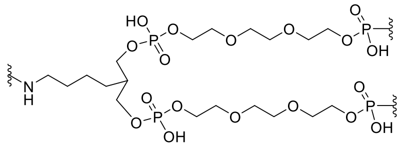

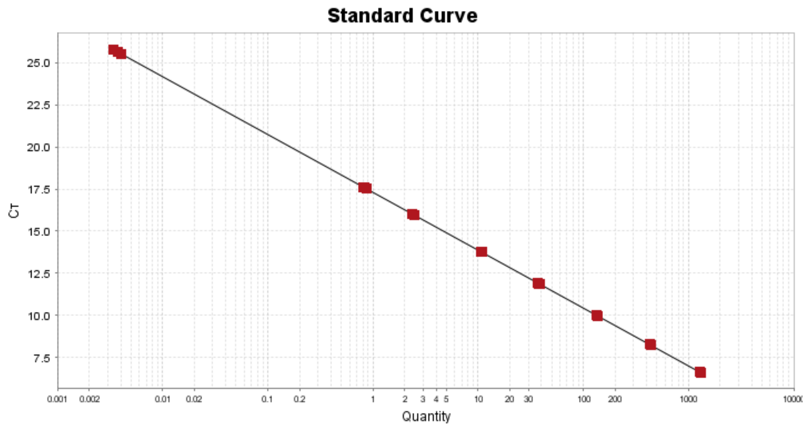

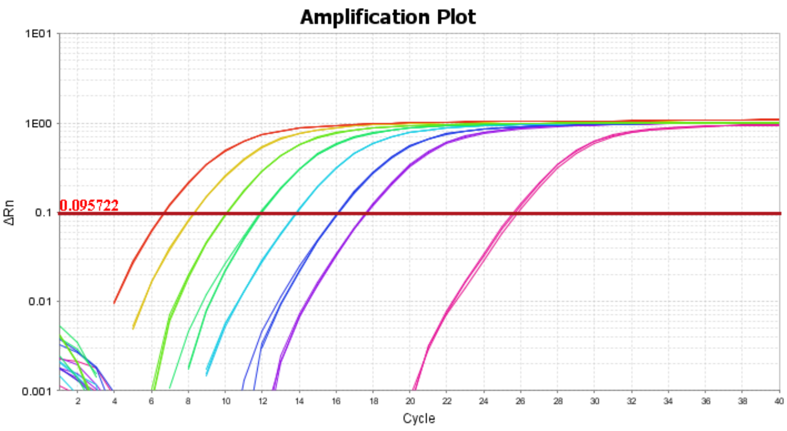

Chemical Space Analysis of a Virtual DEL Originating from $4N_3$-BA-(2S,4S)-N_3-PRO-HP

Chemical Space Analysis

Computational Environment

Chemical space analyses were performed using KNIME Analytics Platform (v5.4.3) in combination with RDKit (v2024.09.2), Marvin Extensions 4.7.0 (based on Marvin 25.1.3), and Python 3.11.6 within a custom Conda environment (Conda v24.11.3). Canonical SMILES were used as the molecular representation throughout the study.
"MarvinSketch was used for generating and editing chemical structures, primarily for SMARTS pattern creation within KNIME workflows, MarvinSketch 25.1.1, ChemAxon (https://www.chemaxon.com)."

Selection of Building Blocks

To ensure structural diversity in the virtual library, building blocks (BBs) used for compound generation were selected according to the following workflow. Active methylene compounds and terminal alkynes were sourced from the commercial catalog of Enamine Ltd., and processed through the steps below:
1. Desalting
2. Fingerprinting: Conversion to 1024-bit Morgan fingerprints (radius = 2)
3. Clustering: K-means clustering of the fingerprint space
4. Selection: The compound closest to each cluster centroid was selected

The number of selected BBs and the total number of compounds in the original catalog were as follows:
- Active methylene derivatives: 500 (from 1,194)
- Terminal alkyne derivatives: 500 (from 4,513)

Azide-containing building blocks used in the study included:
- Azidobenzoic acids (3 compounds)
  - 2-Azidobenzoic Acid (CAS: 31162-13-7)
  - 3-Azidobenzoic Acid (CAS: 1843-35-2)
  - 4-Azidobenzoic Acid (CAS: 6427-66-3)
- Azidoproline derivatives (4 compounds)
  - (2S,4R)-FMOC-4-azido-proline (CAS: 702679-55-8)
  - (2S,4S)-FMOC-4-azido-proline (CAS: 263847-08-1)
  - (2R,4S)-FMOC-4-azido-proline (CAS: 2137142-63-1)
  - (2R,4R)-FMOC-4-azido-proline (CAS: 1378847-51-8)

Virtual Library Generation

Virtual compound libraries were generated using the selected building blocks according to the following schemes:
- Scaffold A: Approximately 3 million compounds were generated by combinatorial reactions among 500 active methylenes, 3 azidobenzoic acids, 4 azidoprolines, and 500 terminal alkynes.
- Scaffold B: Approximately 3 million compounds were generated by reacting 3 azidobenzoic acids and 4 azidoprolines with 500 terminal alkynes (with reactions occurring at two positions).

To simplify the chemical structures, DNA-conjugation sites were mimicked by methyl groups, and carboxylic acid moieties were modeled as N-methylamides.

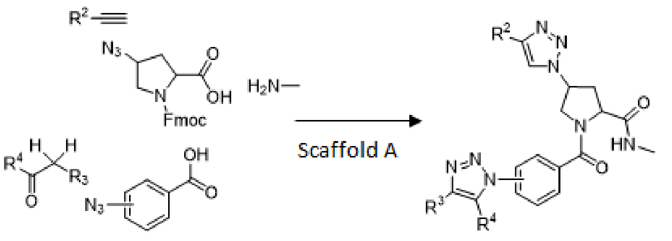
Scaffold A

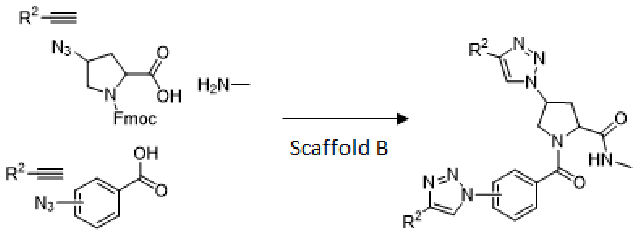
Scaffold B

Comparison of Chemical Space (UMAP + KDE)

Chemical spaces of the Scaffold A and B libraries were compared with the ChEMBL compound set using UMAP followed by kernel density estimation (KDE) for diversity quantification.

Representative Compound Selection

To reduce computational cost while retaining structural diversity, representative compounds were extracted from each library. The number of clusters (5,000) was empirically chosen to balance diversity and manageability.

• Clustering Method:
Morgan fingerprints (radius = 2, 512 bits) → MiniBatch K-means clustering
Parameters: n_clusters = 5000, batch_size = 10000, random_state = 42

• Scaffold A/B:
Each set of 3 million compounds was clustered, and the molecule closest to the centroid of each cluster was selected (5000 representatives each).

• ChEMBL Dataset:
Structures were extracted from ChEMBL_35, desalinated, deduplicated, and filtered by molecular weight (100–1000 Da), resulting in 2,324,649 compounds.
The same clustering procedure was applied, and 50 molecules were randomly selected from each cluster to obtain a total of 208,577 representative compounds.

Dimensionality Reduction Using UMAP

All 218,577 compounds (5000 from Scaffold A, 5000 from Scaffold B, and 208,577 from ChEMBL) were embedded into a 2D chemical space using UMAP under the following settings:

• Morgan Fingerprints (1024 bits, radius = 2)
• UMAP parameters: n_neighbors = 100, n_components = 2, random_state = 42

The resulting 2D coordinates were used to visualize and compare the chemical space coverage across libraries.

CPDS Parameter (Scaffold A)

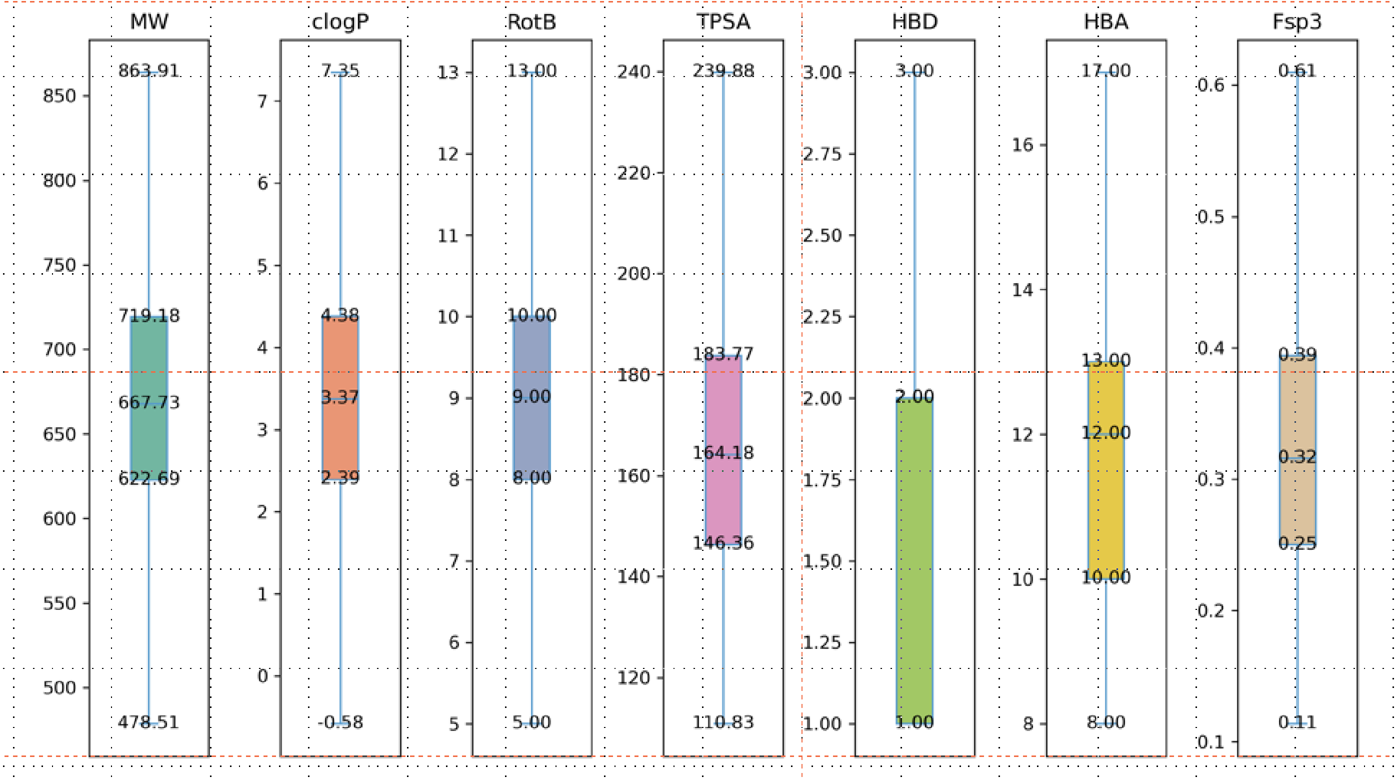

Supplement: Supplementary file 1 [file ijms-26-09501-s001.zip › ijms-3886408-supplementary.pdf]
